# Function and evolution of allelic variations of *Sr13* conferring resistance to stem rust in tetraploid wheat (*Triticum turgidum* L.)

**DOI:** 10.1111/tpj.15263

**Published:** 2021-05-29

**Authors:** Baljeet K. Gill, Daryl L. Klindworth, Matthew N. Rouse, Jinglun Zhang, Qijun Zhang, Jyoti S. Sharma, Chenggen Chu, Yunming Long, Shiaoman Chao, Pablo D. Olivera, Timothy L. Friesen, Shaobin Zhong, Yue Jin, Justin D. Faris, Jason D. Fiedler, Elias M. Elias, Shuyu Liu, Xiwen Cai, Steven S. Xu

**Affiliations:** ^1^ Department of Plant Sciences North Dakota State University Fargo ND 58108 USA; ^2^ USDA‐ARS Cereal Crops Research Unit Edward T. Schafer Agricultural Research Center Fargo ND 58102 USA; ^3^ USDA‐ARS Cereal Disease Laboratory St Paul MN 55108 USA; ^4^ Texas A&M AgriLife Research Amarillo TX 79106 USA; ^5^ Department of Plant Pathology University of Minnesota St Paul MN 55108 USA; ^6^ Department of Plant Pathology North Dakota State University Fargo ND 58108 USA

**Keywords:** *Triticum turgidum*, tetraploid wheat, *Puccinia graminis* f. sp. *tritici*, stem rust, Ug99, *Sr13* alleles

## Abstract

The resistance gene *Sr13* is one of the most important genes in durum wheat for controlling stem rust caused by *Puccinia graminis* f. sp. *tritici* (*Pgt*). The *Sr13* functional gene *CNL13* has haplotypes R1, R2 and R3. The R1/R3 and R2 haplotypes were originally designated as alleles *Sr13a* and *Sr13b*, respectively. To detect additional *Sr13* alleles, we developed Kompetitive allele specific PCR (KASP™) marker *KASPSr13* and four semi‐thermal asymmetric reverse PCR markers, *rwgsnp37–rwgsnp40*, based on the *CNL13* sequence. These markers were shown to detect R1, R2 and R3 haplotypes in a panel of diverse tetraploid wheat accessions. We also observed the presence of *Sr13* in durum line CAT‐A1, although it lacked any of the known haplotypes. Sequence analysis revealed that *CNL13* of CAT‐A1 differed from the susceptible haplotype S1 by a single nucleotide (C2200T) in the leucine‐rich repeat region and differed from the other three R haplotypes by one or two additional nucleotides, confirming that CAT‐A1 carries a new (R4) haplotype. Stem rust tests on the monogenic, transgenic and mutant lines showed that R1 differed from R3 in its susceptibility to races TCMJC and THTSC, whereas R4 differed from all other haplotypes for susceptibility to TTKSK, TPPKC and TCCJC. Based on these differences, we designate the R1, R3 and R4 haplotypes as alleles *Sr13a*, *Sr13c* and *Sr13d*, respectively. This study indicates that *Sr13d* may be the primitive functional allele originating from the S1 haplotype via a point mutation, with the other three R alleles probably being derived from *Sr13d* through one or two additional point mutations.

## Significance Statement

A new resistant haplotype (R4) of *Sr13*, designated as *Sr13d*, was identified in the genotype CAT‐A1, and based on sequencing studies this allele is hypothesized to be a primitive functional resistant allele of *Sr13*. We established from extensive rust tests that haplotypes R1 and R3 should be separated, and R3 was designated *Sr13c* and determined to be preferable to *Sr13a* for breeding, while *Sr13d* was undesirable for breeding due to susceptibility to stem rust Ug99.

## INTRODUCTION

Stem rust, caused by the pathogen *Puccinia graminis* Pers. f. sp. *tritici* Eriks and E. Henn (*Pgt*), is historically the most damaging disease of hexaploid (*Triticum aestivum* L., 2*n* = 6*x* = 42, AABBDD) and tetraploid (*T. turgidum* L., 2*n* = 4*x* = 28, AABB) wheat. The occurrence of new virulent races, and disease outbreaks and epidemics in East Africa (Olivera *et al*., 2015), Europe (Bhattacharya, 2017; Olivera Firpo *et al*., 2017) and Central Asia (Shamanin *et al*., 2018) in the last two decades indicate that wheat stem rust is an emerging disease, posing a threat to wheat production worldwide. Since the discovery of *Sr31*‐virulent race TTKSK in East Africa in 1998 (Pretorius *et al*., 2000; Jin *et al*., 2008), 12 additional *Pgt* races have evolved which are now commonly referred to as the Ug99 race group (Fetch *et al*., 2016). There are additional *Pgt* races unrelated to the Ug99 race group that are of concern. Race TRTTF was detected in Yemen in 2006 (Singh *et al*., 2011), and is the first known race with virulence to *Sr1RS^Amigo^* (Olivera, Badebo, *et al*., 2012; Singh *et al*., 2015). Race TKTTF caused a severe epidemic in Ethiopia in 2013 on the cultivar Digalu that carried the *SrTmp* gene (Olivera *et al*., 2015). This race has been detected across the Middle East (Singh *et al*., 2015), East Africa and Europe (Olivera Firpo *et al*., 2017; Lewis *et al*., 2018). A variant of race TKTTF was identified in Germany having added virulence to genes *Sr7a*, *Sr33*, *Sr45* and *SrTt‐*3 (Olivera Firpo *et al*., 2017). A severe stem rust epidemic on durum wheat occurred in Sicily, Italy in 2016 (Bhattacharya, 2017), and the race responsible for the severe damage was identified as TTRTF (Patpour *et al*., 2017). This race was first reported in Georgia in 2014 and has also been detected in Ethiopia (Olivera *et al*., 2019).

Because many known *Sr* genes have been defeated by the Ug99 race group and new races have emerged, the various world core collections of wheat and its relatives have been surveyed for stem rust resistance, and numerous accessions with resistance to the Ug99 race group and other new races have been identified (Jin and Singh, 2006; Jin *et al*., 2007; Xu *et al*., 2009; Olivera *et al*., 2011; Rouse and Jin, 2011; Rouse, Olson, *et al*., 2011; Olivera, Jin, *et al*., 2012; Chao *et al*., 2017; Olivera, 2018; Arora *et al*., 2019). Recent advances in genome sequencing, gene cloning and DNA marker technology have provided enormous resources and tools for rapid and precise identification of genes of interest in wheat and its relatives. In wheat, the chip‐based 9K (Cavanagh *et al*., 2013) and 90K single nucleotide polymorphism (SNP) arrays (Wang *et al*., 2014) have been widely used in the past decade for the identification of *Sr* genes through high‐density linkage and association mapping (Chao *et al*., 2017; Nirmala *et al*., 2017; Chen *et al*., 2018). The new PCR‐based SNP genotyping technologies such as Kompetitive allele specific PCR (KASP™; https://www.lgcgroup.com) and semi‐thermal asymmetric reverse PCR (STARP) (Long *et al*., 2017) have been used to identify *Sr* genes using high‐throughput genotyping technologies (Nirmala *et al*., 2017; Saini *et al*., 2018). The reference genomes of common wheat (IWGSC, 2018), durum wheat (*T. turgidum* subsp. *durum* (Desf.) Husn.) (Maccaferri *et al*., 2019) and their progenitors (Avni *et al*., 2017; Luo *et al*., 2017; Ling *et al*., 2018) that have recently become available have not only allowed localization of genetically mapped genes to specific genomic regions (Chen *et al*., 2018) but also, along with sequences of cloned genes, they have been used to develop gene‐specific DNA markers such as the derived cleaved amplified polymorphic sequence (dCAPS) and STARP markers that are diagnostic for *Sr13* (Zhang *et al*., 2017; Saini *et al*., 2018). Such markers are especially useful in determining the presence of the target genes and alleles in the uncharacterized germplasm collections and breeding populations.

In the primary gene pool of wheat, tetraploid wheat is a good source of stem rust resistance. Several *Sr* genes, such as *Sr2* (Knott, 1968), *Sr9e* (Stakman *et al*., 1962), *Sr9g* (Stakman *et al*., 1962), *Sr11* (Knott and Anderson, 1956), *Sr12* (Knott, 1984), *Sr13* (Knott, 1962; Simons *et al*., 2011), *Sr14* (Knott, 1962) and *Sr8155B1* (Nirmala *et al*., 2017), originated from tetraploid wheat. The *Sr13* gene is widely present in tetraploid wheat (Zhang *et al*., 2017), originating from Khapli emmer (*T. turgidum* subsp. *dicoccum* (Schrank) Schübl.) accession CItr 4013 (Knott, 1962) and the Ethiopian durum landrace ST464 (PI 191365) (Klindworth *et al*., 2007). The *Sr13* gene was recently identified as a coiled‐coil nucleotide‐binding leucine‐rich repeat (NLR) gene, with 13 DNA polymorphisms (haplotypes) being detected mostly in the LRR region (Zhang *et al*., 2017). The haplotypes were classified as resistant (R) or susceptible (S) based on their reaction to TTKSK, and three R (R1, R2 and R3) and 10 S (S1–S10) haplotypes were observed. When tested with JRCQC, the R1 and R3 haplotypes were resistant, while the R2 haplotypes were susceptible. The R1/R3 and R2 haplotypes were designated *Sr13a* and *Sr13b*, respectively (Zhang *et al*., 2017).

Several sets of monogenic lines resistant to stem rust were developed and are maintained by the United States Department of Agriculture Agricultural Research Service (USDA‐ARS) at Fargo, ND (Table S1 in the online Supporting Information). Included among the lines are 70 tetraploids comprising 12 sets developed from crosses to the stem rust susceptible parent Marruecos 9623 (PI 192334). The 12 parents of the stem rust resistant monogenics include historically important sources of stem rust resistance developed in the mid‐20th century. For example, Vernal was the source of *Sr9e*, Khapli was the source of *Sr13* and *Sr14*, ST464 was also a source of *Sr13* and Iumillo was the source of *Sr12* (McIntosh *et al*., 1995). Klindworth *et al*. (2007) reported that the tetraploid monogenic line ST464‐C1, a derivative of ST464, carried a gene on chromosome 6A that was postulated to be *Sr13*. Four monogenic lines were derived from Khapli, including KL‐A, KL‐B, KL‐C and KL‐D (Williams and Miller, 1982). Of these four lines, KL‐A and KL‐D were excluded from carrying *Sr13* based on infection types (ITs) and avirulence/virulence tests over a range of *Pgt* races. Williams and Gough (1965) observed a gene conditioning IT 2^=^ in Khapli crosses, and they concluded that this gene was probably *Sr13*. The KL‐B line has this trait (Williams and Miller, 1982), indicating it probably carries *Sr13*, but KL‐C was susceptible to isolate 111‐SS2 (LBBLB), indicating it was unlikely to be *Sr13*. Mapping studies have never confirmed the genes in KL‐B and ST464‐C1 to be *Sr13* alleles; however, sequencing has provided genetic confirmation that the gene in ST464‐C1 belongs to the R3 haplotype of *Sr13* (Zhang *et al*., 2017).

In addition to cultivated emmer and durum wheat, *T. turgidum* subsp. *carthlicum* (Neyski) Á. Löve & D. Löve and *T*. *turgidum* subsp. *polonicum* (L.) Thell. (hereafter abbreviated as *T. carthlicum* and *T*. *polonicum*, respectively) are also cultivated forms of tetraploid wheat. They were reported to possess genes for many useful agronomic traits, including resistance to stem rust (Raut *et al*., 1984; Laidò *et al*., 2015). Investigations of tetraploid wheat accessions for seedling resistance to stem rust found that *T*. *carthlicum* accession PI 387696 and *T*. *polonicum* accession CItr 14803 were resistant to Ethiopian races of *Pgt* (Olivera *et al*., 2011; Zhuang *et al*., 2011). To date, no *Sr* genes have been identified from either of these subspecies. Initially, the objective of our study was to determine the inheritance of resistance in these lines, determine the chromosomal locations of the resistance genes and identify closely linked markers. However, as the genes were identified it became clear that we were conducting a study of *Sr13*. We therefore expanded the study to include the USDA monogenic lines which led to the discovery of a new resistant haplotype (R4) of *Sr13*. Thus, the objective of the overall experiment was to conduct inheritance, chromosomal location, linkage and sequencing studies of the *Sr13* region with multi‐pathotype testing to support the conclusions.

## RESULTS

### Inheritance and mapping of stem rust resistance in *T. carthlicum* PI 387696

**Figure 1:**
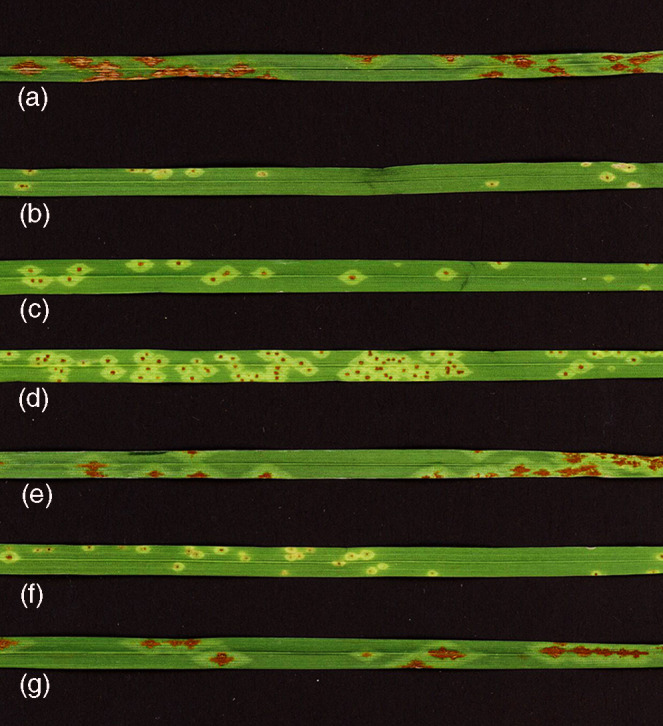
Infection types (ITs) of F_1_, F_2_ and recombinant inbred line (RIL) plants from the cross Rusty × PI 387696 after inoculation with *Puccinia graminis* f. sp. *tritici* (*Pgt*) race TMLKC. (a) Rusty (IT 34), (b) PI 387696 (IT 1), (c) F_1_ plant with (IT 1;), (d) F_2_ resistant plant (IT 1), (e) F_2_ susceptible plant (IT 34), (f) resistant RIL plant (IT 1;), and (g) susceptible RIL plant (IT 34).

Rusty and PI 387696 had susceptible and resistant reactions, respectively, to *Pgt* races TTKSK, TRTTF and TMLKC (Figure 1). To identify the *Sr* gene(s) in PI 387696, the F_1_ hybrid and F_2_ and recombinant inbred line (RIL) (F_7_) populations were developed from the cross Rusty × PI 387696. Sixteen F_1_ plants were evaluated with *Pgt* race TMLKC and four F_1_ plants with races TTKSK, and TRTTF. In addition, 83, 90, and 167 F_2_ plants were tested with *Pgt* races TTKSK, TRTTF, and TMLKC, respectively. All F_1_ plants had a level of resistance similar to PI 387696. In the F_2_ populations, the plants tested with TTKSK, TRTTF and TMLKC segregated for resistant and susceptible phenotypes in ratios of 63:20, 68:22 and 128:39, respectively, fitting the expected 3:1 ratio for a single dominant gene (Table S2). Among the 190 RILs tested with TTKSK, TRTTF and TMLKC, 85 had resistant (ITs fleck (;), 1, ;2, and 22^+^) and 98 had susceptible (ITs 3^+^, 4) reactions to the three *Pgt* races, whereas seven lines had ambiguous or intermediate (ITs 2/3^+^, 2^+^/3^+^, 3^+^/2 and 22^+^/3^+^) reactions and were excluded from further linkage analysis. The segregation of 85 resistant versus 98 susceptible fitted an expected 1:1 ratio for a single gene (Table S2). Therefore, the stem rust tests all substantiated that stem rust resistance against the three *Pgt* races in PI 387696 was controlled by a single dominant gene.

**Figure 2:**
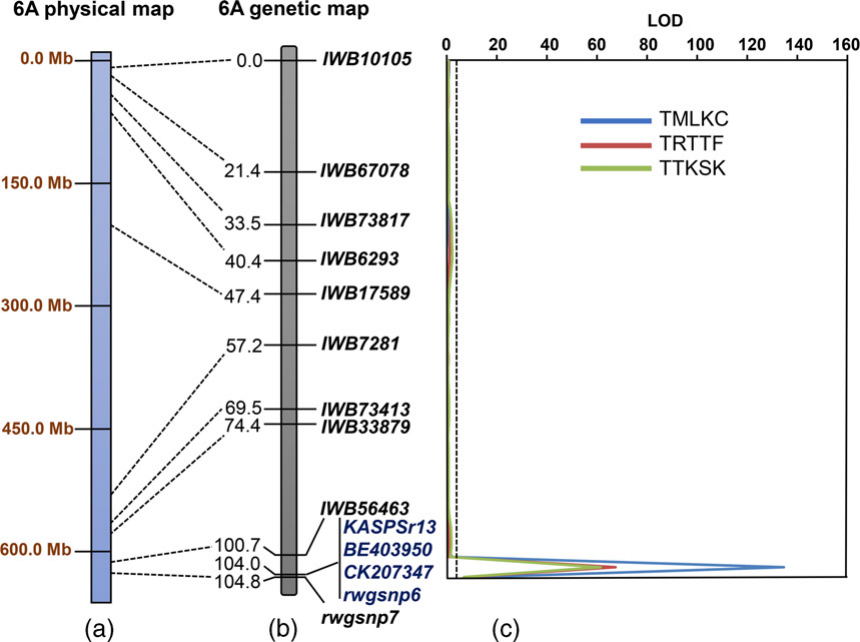
(a) Coordinate positions (bp) of the markers associated with *Sr13* on the chromosome arm 6AL physical map in the reference genome RefSeq v1.0 of common wheat (IWGSC, 2018). (b) Regions of the genetic linkage map for chromosome arm 6AL harboring quantitative trait loci for stem rust resistance to *Puccinia graminis* f. sp. *tritici* races (TMLKC, TRTTF and TTKSK) in a Rusty × PI 387696 recombinant inbred line population. The centimorgan (cM) distances between the marker loci are indicated to the left‐hand side of the linkage map and marker loci are on the right‐hand side of the linkage map. (c) The critical logarithm of the odds (LOD) threshold is 3.0 and the LOD score scale is represented at the top.

A total of 9996 polymorphic SNP markers polymorphic between Rusty and PI 387696 were identified by analyzing 190 RILs (Rusty × PI 387696) with the 90K iSelect SNP array. Because many of these polymorphic markers were co‐segregating (Table S3), only 1542 of the polymorphic SNP markers were used for genetic map construction by choosing one marker from each set of co‐segregating markers (Table S4). In addition to the 90K SNP markers, 74 simple sequence repeats (SSRs), two expressed sequence tag sequence‐tagged site (EST‐STS) markers, and two STARP markers (*rwgsnp6* and *rwgsnp7*) were also included in linkage map construction (Tables 1 and S3, Figures 2 and S1). A total of 1621 polymorphic markers were finally used to construct the genetic linkage groups.

**Table 1:** Semi‐thermal asymmetric reverse PCR (STARP) and Kompetitive allele specific PCR (KASP™) markers developed in this study

Marker	SNP source	Primer type	Primer sequence (5ʹ–3ʹ)	*T*_m_ (°C)
*rwgsnp6*	IWB71956	F ‐ primer 1	[Tail 2]GGAAAACGACGACGACT	54.45
		F ‐ primer 2	[Tail 1]GGAAAACGACGACGCTC	56.07
		R ‐ primer	TGGAAAATCAGCGCTCGACAG	61.27
*rwgsnp7*	IWB34398	F ‐ primer 1	[Tail 2]AGCACACTACTACGAGAACAT	56.50
		F ‐ primer 2	[Tail 1]AGCACACTACTACGAGACAAG	57.17
		R ‐ primer	CGACCCATACTCAAGACCATCTG	60.49
*rwgsnp37.1*	CNL13	F ‐ primer 1	[Tail2]AAACCTTTGTTCTCTAACTCTGC	56.99
		F ‐ primer 2	[Tail1]AAACCTTTGTTCTCTAACTACGT	55.83
		R ‐ primer	GCGTCAGCAAGAAGTCATCATCA	61.47
*rwgsnp37.2*	CNL13	R ‐ primer	CACCATGTATTCAGCAAGAAGTCA	59.30
*rwgsnp38*	CNL13	F ‐ primer 1	[Tail1]GAATGTATATGTCATGTCCAACG	55.57
		F ‐ primer 2	[Tail2]GAATGTATATGTCATGTCCGCCT	58.43
		R ‐ primer	GCGACTGTAATCTTCAGTTATCCTC	59.31
*rwgsnp39*	CNL13	F ‐ primer 1	[Tail1]GCCTGAGGAAGTTTAAATATTGG	57.18
		F ‐ primer 2	[Tail2]GCCTGAGGAAGTTTAAATACTAT	56.52
		R ‐ primer	CGTACGCAGAGGATAACTGAAGA	59.94
*rwgsnp40*	CNL13	F ‐ primer 1	[Tail1] GGAATATACAACCCCATACGAC	56.12
		F ‐ primer 2	[Tail2] GGAATATACAACCCCATATGCG	56.77
		R ‐ primer	CACTTACTTCTTGGCTCAGAAGACA	60.74
*KASPSr13*	CNL13	F ‐ primer FAM	CACAAAACCTTTGTTCTCTAACTATGC	59.41
		F ‐ primer HEX	CACACAAAACCTTTGTTCTCTAACTATGT	60.63
		R ‐ primer	CCATGTATTCAGCAAGAAGTCATCATCAT	61.99

SNP, single nucleotide polymorphism; *T*_m_, melting temperature.

The primer type in STARP markers: F = forward, R = reverse. STARP markers *rwgsnp37.1* and *rwgsnp37.2* have the same F primers. For KASP marker *KASPSr13*, F primer 1 is coupling with FAM for resistant allele C, whereas F primer 2 is coupling with HEX for susceptible allele either T or no amplification.

Primer sequence: Tail1, GCAACAGGAACCAGCTATGAC‐3ʹ; Tail2, GACGCAAGTGAGCAGTATGAC‐3ʹ (Long *et al*., 2017).

For linkage analysis, 1621 polymorphic markers were assigned to 14 linkage groups representing 14 (A and B genomes) chromosomes with a total map length of 2356.5 cM and an average marker density of 1.5 cM/marker (Table S4). For quantitative trait locus (QTL) analysis, a permutation test consisting of 1000 iterations revealed that the critical logarithm of the odds (LOD) threshold value ranged from 2.7 to 3.0 (*P* = 0.01). Therefore, a critical LOD threshold value of 3.0 was used to declare a significant QTL. A major QTL, designated as *QSr.rwg‐6A.3*, for resistance to the three *Pgt* races was mapped to the distal region on chromosome arm 6AL, which is associated with *Sr13* (Tables 2 and S5). To verify if the *Sr* gene was the same as or different from *Sr13*, we used the *Sr13* gene‐specific KASP marker *KASPSr13* to genotype the RIL population. The linkage analysis showed that *KASPSr13* co‐segregated with the *Sr13* markers *BE403950* and *CK20734* (Simons *et al*., 2011) and the STARP marker *rwgsnp6* (Figure 2), confirming that the *Sr* gene in PI 387696 was *Sr13*.

**Table 2:** Single‐trait multiple interval mapping (SMIM) analysis of the quantitative trait locus (QTL) for resistance to stem rust caused by *Puccinia graminis* f. sp. *tritici* races TTKSK, TRTTF and TMLKC

QTL	Chromosome arm	Marker interval	Marker position	TTKSK			TRTTF			TMLKC		
				LOD	*R* ^2^	Add.	LOD	*R* ^2^	Add.	LOD	*R* *^2^*	Add.
*Qsr.rwg‐6A.3*	6AL	*IWB6825* ‐*rwgsnp7*	94.7–123.2 cM	60.97	0.76	2.51	66.95	0.81	2.48	134.00	0.79	3.16

Add. is the additive effects of the QTL.

### Identification of the stem rust resistance gene from *T. polonicum* CItr 14803

One‐hundred and eighty BC_1_F_2_ families (CItr 14803/2* Rusty) were tested with TMLKC and TTKSK to determine the genotype of each BC_1_F_2_ plant. Approximately 25 plants in each family were tested with TMLKC. We observed 53 homozygous susceptible, 82 segregating and 45 homozygous resistant families in the TMLKC test. A chi‐square test indicated that this result was an acceptable fit to a 1:2:1 ratio (χ^2^ = 2.133, *P* = 0.344). Following testing with TMLKC, each family was also tested with TTKSK. Only five plants per family were tested with TTKSK, but by comparing the data with the results from the TMLKC test a satisfactory classification could be determined. As a result, all families scored as either homozygous resistant or homozygous susceptible to TMLKC could be detected and confirmed in the TTKSK test. Due to the small family size there was some incorrect classification of heterozygous families in the TMLKC test. However, the results of the two tests were sufficiently consistent to conclude that the same gene conditioned resistance to both races, and the inconsistencies were due to the small family size of the TTKSK test.

**Figure 3:**
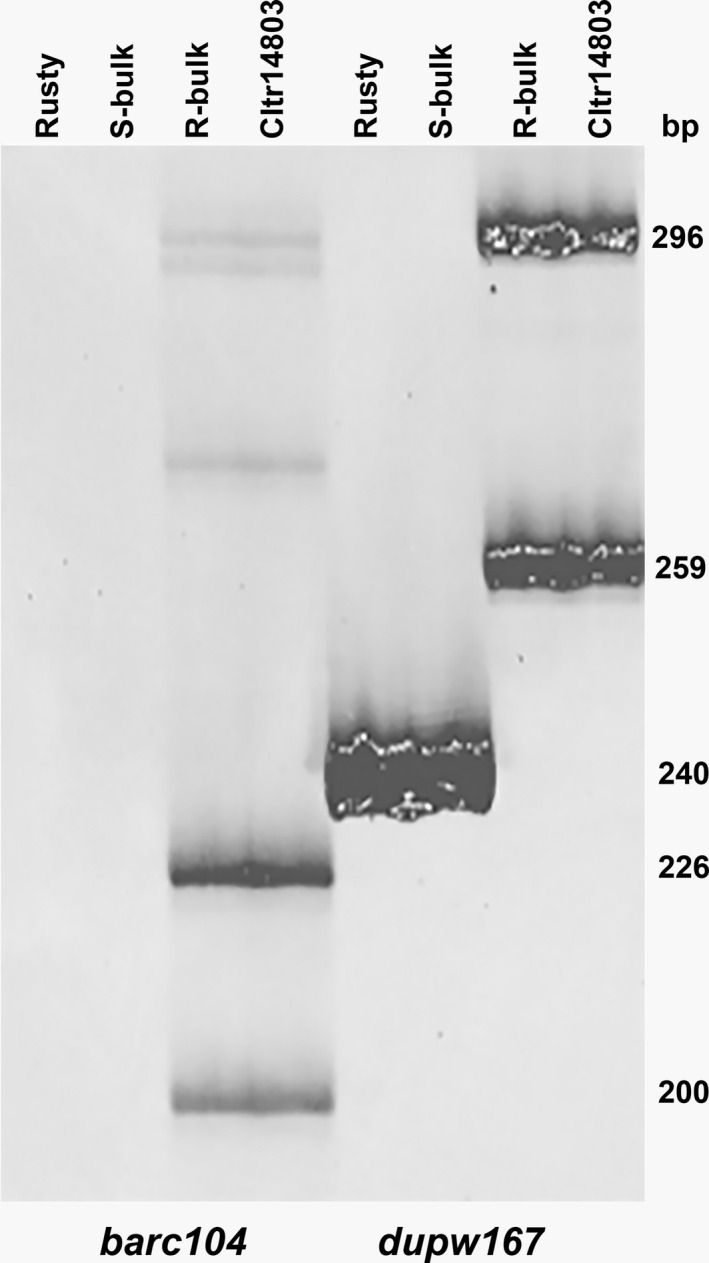
Bulk segregant analysis of stem rust resistance in *Triticum polonicum* CItr 14803/2*Rusty families using the markers *barc104* and *dupw167*. Each bulk was composed of DNA from 10 plants classified as homozygous‐susceptible (S‐bulk) or homozygous‐resistant (R‐bulk) to *Puccinia graminis* f. sp. *tritici* race TMLKC based on progeny testing. Failure of *barc104* to amplify the 200 and 226 bp bands in Rusty and the S‐bulk indicated that *barc104* was a dominant‐coupling phase marker.

Bulk segregant analysis using 67 SSR markers (Table S6) revealed that 65 markers were independent of the rust resistance gene but two markers, *barc104* and *dupw167*, were closely linked to the IT (Figure 3). Marker *barc104* was a dominant marker linked in coupling phase with the resistance gene, and *dupw167* was co‐dominant. Both marker loci are located near the 6AL telomere and are tightly linked to *Sr13* (Simons *et al*., 2011). This result suggested that additional examination of markers linked to *Sr13* was warranted. Marker *dupw167* was then tested on all 180 BC_1_F_2_ plants. Symbols R and S were assigned to the resistant and susceptible alleles, respectively. For the *dupw167* locus, the 240 bp amplicon from Rusty (Figure 3) was assigned allele A, and the 259 and 296 bp amplicons from CItr 14803 were assigned allele B. When the *dupw167* and stem rust data were combined, there were 53 AASS, 2 AARS, 80 ABRS and 45 BBRR plants. Thus, only two of the 180 BC_1_F_2_ plants showed recombination between the rust resistance and *dupw167* loci. Using the Kosambi function, the distance from the resistance gene to *dupw167* was 1.11 ± 0.78 cM. When compared with the results of Simons *et al*. (2011), our results strongly suggest that the resistance gene in CItr 14803 was located at the *Sr13* locus.

The marker *KASPSr13* was then tested on the 180 BC_1_F_2_ plants. In the analysis, we found that the AB and BB classes for *KASPSr13* did not form distinct clusters. The AB and BB plants were analyzed as a single cluster (B_), which could be separated into RS or RR classes by progeny testing. Based on these classifications, we observed a total of 53 AASS, 82 B_RS and 45 B_RR plants. Despite pooling of the AB and BB classes, there were no apparent recombinants in the *KASPSr13* analysis. In addition, the same 53 plants were classified as AASS in the analysis of *KASPSr13* and *dupw167*. These results indicate that the *Sr* gene in CItr 14803 was located at the *Sr13* locus on chromosome arm 6AL.

### Haplotype and sequence analysis of *Sr13* genotypes

**Figure 4:**
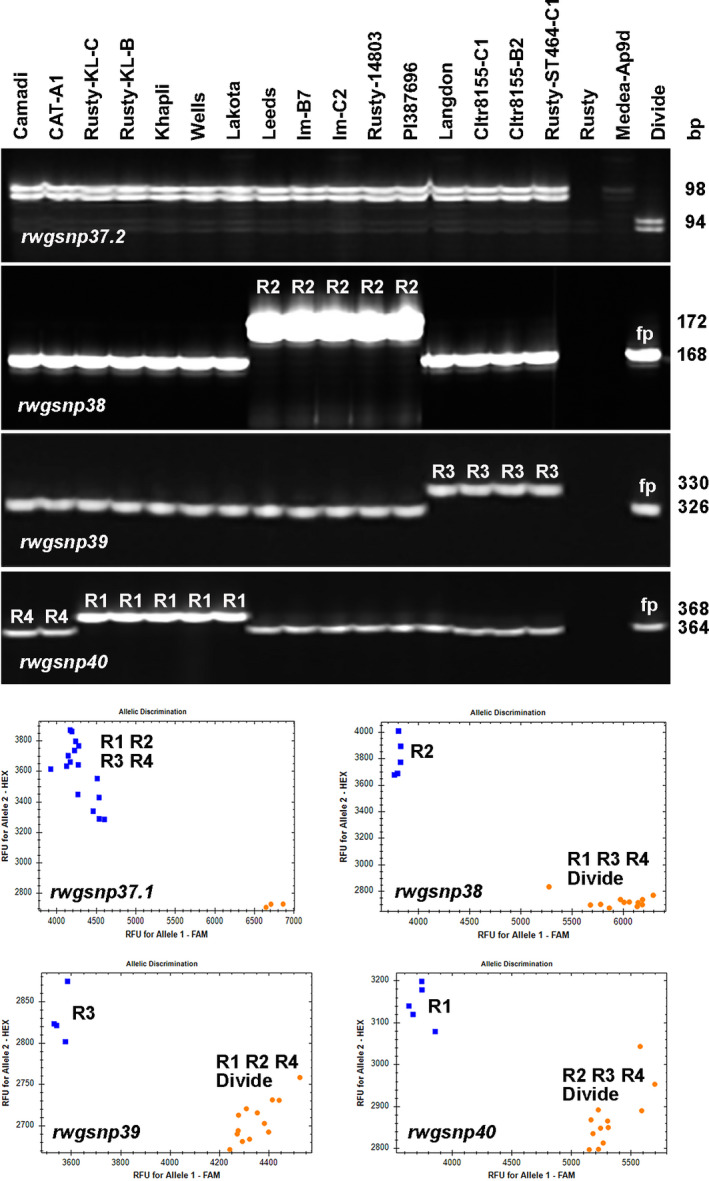
Analysis of markers *rwgsnp37*, *rwgsnp38*, *rwgsnp39* and *rwgsnp40* in tetraploid wheat cultivars and lines. Semi‐thermal asymmetric reverse PCR marker analysis of 19 cultivars and lines is shown on polyacrylamide gels for the four markers. For marker *rwgsnp37.2*, a 98 bp amplicon indicated a positive result for *Sr13* (Saini *et al*., 2018), while a 94 bp amplicon as seen in Divide or a null result indicated absence of *Sr13*. For marker *rwgsnp38*, genotypes having the R1/R3/or R4 haplotype produce a 168 bp amplicon while genotypes carrying the R2 haplotype produce a 172 bp amplicon. For marker *rwgsnp39*, genotypes having the R1/R2/or R4 haplotype produce a 326 bp amplicon while genotypes carrying the R3 haplotype produce a 330 bp amplicon. For marker *rwgsnp40*, genotypes having the R2/R3/or R4 haplotype produce a 364 bp amplicon while genotypes carrying the R1 haplotype produce a 368 bp amplicon. Any genotypes such as Camadi or CAT‐A1, which are positive for *Sr13* by *rwgsnp37.2* but which are not classified as R1, R2 or R3, are classified as R4 haplotype. The figure also includes three genotypes that are negative for *Sr13* by *rwgsnp37.2*, and one of these, Divide, produces false positives (fp) when tested with *rwgsnp38*, *rwgsnp39,* and *rwgsnp40*. These are easily detected by *rwgsnp37.2*. For the real‐time images, a clear separation of groups was observed, the number of genotypes and identity of the genotypes in each group was observed to match the corresponding gel. In the real‐time image of *rwgsnp37.1*, the blue cluster is *Sr13*‐positive lines; in the *rwgsnp38* image, the blue cluster is R2‐positive lines; in the *rwgsnp39* image, the blue cluster is R3‐positive lines; and in the *rwgsnp40* image, the blue cluster is R1‐positive lines. R4‐positive lines appear within the blue cluster in the *rwgsnp37.1* image, but appear in the orange clusters of the three other markers.

To detect haplotypes, the presence of *Sr13* must first be ascertained followed by the use of markers to detect either the R1, R2 or R3 haplotypes. Marker *rwgsnp37.2* was used to test for the presence of *Sr13*. Any line carrying *Sr13* should be positive for a 98 bp band amplified by *rwgsnp37.2* (Figures 4 and S2a). The markers *rwgsnp38*, *rwgsnp39* and *rwgsnp40* were developed to detect plants carrying haplotypes R2, R3 and R1, respectively (Figures 4 and S2). Khapli, Rusty‐KL‐B, Rusty‐KL‐C, Wells and Lakota all carried the R1 haplotype (Figure 4). Rusty‐14803, PI 387696 and Leeds all carried the R2 haplotype. Langdon and Rusty‐ST464‐C1 carried the R3 haplotype. Both Rusty and Medea Ap9d (*Srdp2*) were null for *rwgsnp37.2*. This result for Medea Ap9d agreed with the conclusion of Zhang *et al*. (2017) that *Srdp2* is not an allele of *Sr13*. Cultivar Divide is shown in Figure 4 to illustrate false positives produced by markers *rwgsnp38*, ‐*39* and ‐*40*. These false positives are easily detected by rejecting any genotype not carrying the 98 bp amplicon from *rwgsnp37.2*.

Results from the tests with *KASPSr13* indicated that eight tetraploid lines carried *Sr13*. Three of these, ST464‐C1, KL‐B and KL‐C, derived from ST464 and Khapli, were expected based on previous results. The five additional lines were derived from three sources: 8155‐B2 and 8155‐C1 were derived from CItr 8155, Im‐B7 and Im‐C2 were derived from Iumillo and CAT‐A1 was derived from Camadi Abdu tipo #103 (PI 192168; and henceforth referred to as Camadi). For analysis of the *CNL13* sequences of these monogenic lines (Tables 3 and S7), we removed Im‐B7 and 8155‐C1 and added PI 387696 and the newly derived near‐isogenic line (NIL) Rusty‐14803.

**Table 3:** Variation in the DNA and amino acid sequences of the leucine‐rich repeat region (LRR) of CNL13 observed in eight tetraploid genotypes

Genotypes	Haplotype	Virulent *Pgt* races	bp of the LRR region of the CNL13 sequence (amino acid at position)				
			1963 (655)	2200 (734)	2272 (758)	2517 (839^a^)	2798 (933^a^)
Rusty‐KL‐B, Rusty‐KL‐C	R1	THTSC, TCMJC, TTTTF?	C (Arg)	C (Arg)	C (Leu)	G (Trp)	C (Thr)
Rusty‐14803, PI 387696, Im‐C2	R2	JRCQC, QFCSC, QCCJC	G (Gly)	C (Arg)	G (Val)	G (Trp)	A (Lys)
Rusty‐ST464, 8155‐B2	R3		G (Gly)	C (Arg)	G (Val)	T (Cys)	C (Thr)
CAT‐A1	R4	TTKSK, TCCJC,^b^	G (Gly)	C (Arg)	G (Val)	G (Trp)	C (Thr)
	S1^c^		G (Gly)	T (Trp)	G (Val)	G (Trp)	C (Thr)

*Pgt*, *Puccinia graminis* f. sp. *tritici*

^a^ Consistent with Zhang *et al*. (2017); beyond position 763 the number of each amino acid has been increased by one to adjust for a 3 bp insertion found in haplotype S10.

^b^ TPPKC, TCMJC and THTSC were also reported in Table S4 as virulent on CAT‐A1, but THTSC and TCMJC do not differentiate R1 from R4.

^c^ Published with permission.

The DNA and amino acid sequences of the four R haplotypes were compared with those reported by Zhang *et al*. (2017) and are summarized in Table 3. Rusty‐KL‐B and Rusty KL‐C had the R1 haplotype, Rusty‐14803, PI 387396 and Im‐C2 had the R2 haplotype, and Rusty‐ST464 and 8155‐B2 had the R3 haplotype (Table S7). However, monogenic line CAT‐A1 was found to carry a new haplotype, named R4 (NCBI Nucleotide Accession MW033594). CAT‐A1 carried the T2200C polymorphism which resulted in amino acid substitution W734R (Tables 3 and S7), which was the single polymorphism that discriminated R from S haplotypes of *CNL13* in Zhang *et al*. (2017). Other than this polymorphism, haplotype R4 was identical to haplotype S1 and possessed polymorphisms at SNPs distinguishing it from the other three R haplotypes (Table 3). Since the R4 haplotype differed from the S1 haplotype only by this single polymorphism (Table 3), that polymorphism appears to be responsible for the *Sr13* gene gaining its resistance function from this T to C point mutation. The R4 haplotype differed from R1 by only 2 bp at positions G1963C/G655R and G2272C/V758L, from R2 by only 1 bp at C2798A/T933K and from R3 by only 1 bp at G2517T/W839C. The sequencing results agreed with the results from haplotyping (Figure 4). Marker *rwgsnp38* showed that lines Im‐B7 and Im‐C2 carried the R2 haplotype, whereas *rwgsnp39* showed that 8155‐C1 and 8155‐B2 carried the R3 haplotype. At the same time, Camadi and CAT‐A1 were assigned to haplotype R4 (Figure 4d) because they were positive for *Sr13* by *rwgsnp37.2*, but were not positive for R1, R2 or R3. These data confirmed that the *CNL13* sequence of CAT‐A1 is correctly categorized as a new R haplotype named R4.

As a check that monogenic lines ST464‐C1, KL‐B, KL‐C, 8155‐B2, 8155‐C1, Im‐B7, Im‐C2 and CAT‐A1 carried *Sr13*, we examined the CItr or PI lines from which they were derived (Table S8). In most instances, these landraces were heterogeneous for the presence of *Sr13*, but *Sr13* was present and the haplotype matched the haplotype of the monogenic lines. For example, only 6 of 15 Camadi plants carried the R4 haplotype, and 13 of 29 ST464 plants carried the R3 haplotype (Table S8). The only exception to this heterogeneity was Iumillo, where 138 plants were examined and no plants having *Sr13* were detected. Although this result is consistent with prior studies of Iumillo, in the present study the failure to detect *Sr13* in Iumillo was notable because it calls into question how was it possible that the monogenic lines Im‐B7 and Im‐C2 carried *Sr13*.

### Comparison of three *Sr13* haplotypes (R1, R2 and R3) for reactions to multiple *Pgt* races

Two experiments were initially conducted to compare the *Sr* genes in *T. carthlicum* PI 387696 and *T. polonicum* CItr 14803 with the established *Sr13* genotypes. Because Medea Ap9d did not carry *Sr13* it functioned only as a check for these experiments. Generally, results from the second experiment were more consistent than results from the first experiment (Table 4). For example, when comparing genotypes carrying haplotypes R1 or R2 in Experiment 2, genotypes were closely similar. Langdon, which carried genes in addition to *Sr13*, was resistant to all races in both experiments. Rusty‐KL‐B and Rusty‐KL‐C, both carrying haplotype R1, exhibited nearly identical ITs in both experiments. Across both experiments, no significant differences were observed among the *Sr13* genotypes for six races, namely RTQQC, RKQQC, RHFSC, TMLKC, TPMKC and TPPKC. The remaining four races fell into three types. When the first type was tested with THTSC and TCMJC the R1 genotypes (Rusty‐KL‐B and Rusty‐KL‐C) were susceptible, whereas the R2 (Rusty‐14803, CItr 14803, and PI 387696) and R3 (Rusty‐ST464‐C1) genotypes were resistant (Table 4, Figure S3). When the second type was tested with QFCSC, the R2 genotypes were susceptible while R1 and R3 genotypes were resistant (Figure S3). When the third type was tested with QTHJC, the R1, R2 and R3 haplotypes were resistant; however, the R3 genotype (Rusty‐ST464‐C1) exhibited the lowest IT (Table 4, Figure S3). Rusty‐ST464‐C1 was the only genotype resistant to all 10 races. Based on this difference, we redesignated the *Sr13a* (R1/R3) allele (Zhang *et al*., 2017) as *Sr13a* (R1) and *Sr13c* (R3), respectively.

**Table 4:** Infection types (ITs) observed on nine genotypes when inoculated with 10 races of *Puccinia graminis* f. sp. *tritici* and incubated at 25°C

Line	*Sr* gene (haplotype)	QTHJC		QFCSC		RTQQC		RKQQC		RHFSC		TMLKC		TPMKC		TPPKC		THTSC		TCMJC	
		1	2	1	2	1	2	1	2	1	2	1	2	1	2	1	2	1	2	1	2
Rusty		43	4	4	4	4	34	–	34	34	4	34	4	43	4	–	34	34	4	34	34
Langdon	*Sr13* (R3) +	1^+^2^−^	2	2^−^cn	1^+^2^−^	2^−^c	2^−^	–	2^−^	1^−^n	1^−^	12^−^c	1^+^	2^−^	2^−^	–	1^+^	0;	1^−^	13c	1^−^1/1^−^0;/22^+^
Rusty‐KL‐B	*Sr13* (R1)	22^+^	32^−^	2^+^3^−^	2^+^3^−^	2	2	–	2^−^	2/2^++^	2^+^3^−^	2^−^c	1^−^	2^−^	2	–	2^+^	3^−^2^+^	34	3^−^2^+^	34
Rusty‐KL‐C	*Sr13* (R1)	22^+^	32^−^	3^−^2^+^	2^+^3^−^	2	2	–	2^−^	2^+^	2^+^3^−^/32	2^−^c	1^+^	2^−^c	2	–	2^+^	3^−^2^+^	33^+^	34	34
Rusty‐ST464‐C1	*Sr13* (R3)	2	22^+^	2^−^	2	2	2	–	2^−^	2	2^+^3^−^	2^−^c	2^−^	2^−^	2^−^	–	2	2^−^2^+^	2^+^	2	22^+^
Rusty‐14803	*Sr13* (R2)	–	3	–	34	–	2	–	2^−^	–	2^+^	–	1^+^	–	2^−^	–	2^+^	–	2^+^	–	22^+^
CItr 14803	*Sr13* (R2)	22^+^	32^−^	43	3^−^2	2	22^+^	–	2	2	2^+^	2^−^	1^+^2^−^	2	2^−^	–	2^−^	2^−^	2^+^	2^−^	2^−^
PI 387696	*Sr13* (R2)	2	2^+^	32^+^	3^−^2	2	2	–	2	2^−^2c	22^+^	2^−^c	1^+^	2^−^	2^−^	–	2	0;1^−^c	2^−^	2^−^c	22^+^
MedeaAp9d	*Srdp2*	32	34c	3^−^2^+^	34	2^+^	23^−^	–	23^−^	2	2^+^3^−^	2	2^+^	22^−^	2	–	2^+^	2	2^+^3^−^	32^+^	34

1, experiment 1; 2, experiment 2. ITs: 0, fleck (;), 1 and 2 are resistant reactions, while 3 and 4 are susceptible reactions. Some plants have combinations of ITs, such as IT 32 which is primarily IT 3 with smaller amount of IT 2. Plus (^+^) and minus (^–^) signs indicate larger and smaller pustules than normally seen for that IT (Stakman *et al*., 1962). C = more chlorosis than normal, n = necrosis. A slash indicates a mixture of infection types observed on different plants in the population.

**Figure 5:**
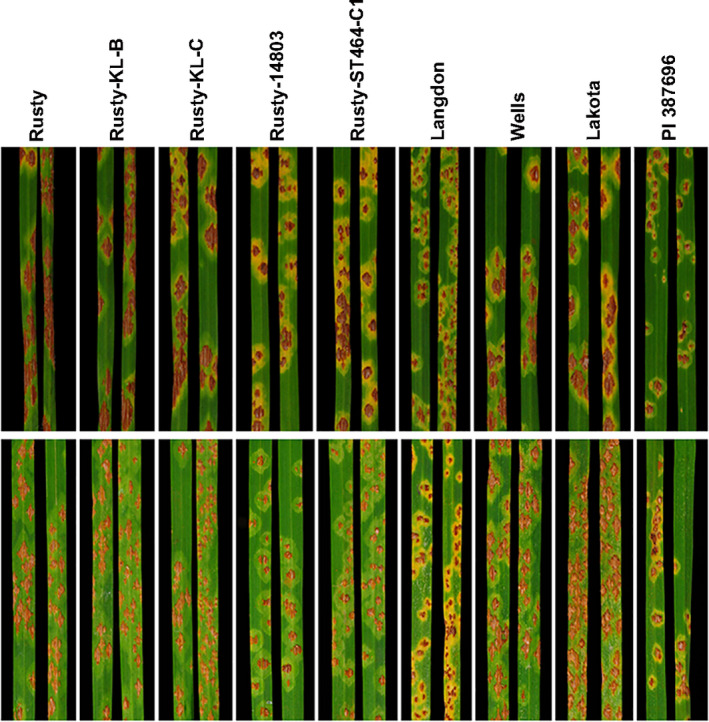
Infection types (ITs) observed on nine durum genotypes inoculated with *Puccinia graminis* f. sp. *tritici* race TCMJC and incubated at 22℃ in two replications with two leaves per replication shown per genotype. In both replications, genotypes carrying the R1 haplotype (Rusty‐KL‐B, Rusty‐KL‐C, Wells and Lakota) exhibit similar susceptibility to TCMJC as Rusty. Genotypes carrying the R2 and R3 haplotype (Rusty‐14803, Rusty‐ST464‐C1, Langdon, and PI 387696) exhibit resistant ITs.

To compare Wells/Lakota virulence on *Sr13* genotypes, races TCMJC and THTSC, which were considered as possibly identical to 15‐WL, were tested at Fargo, ND in a 25°C growth chamber and in a 21°C greenhouse (Table 5). Race TCMJC was also tested at 22°C and 25°C in the growth chambers at St Paul. On Wells and Lakota, THTSC produced IT X (mesothetic) in the 25°C test (Figure S4). In addition, in the greenhouse test at Fargo, Wells and Lakota were resistant to THTSC, indicating that THTSC was unlikely to be the race described as 15‐WL. In contrast, with the exception of a single off‐type plant (IT 23) observed in the greenhouse test, TCMJC produced susceptible ITs on Wells and Lakota, indicating it was probably the 15‐WL race. In addition to being virulent on Wells and Lakota, TCMJC produced similar ITs on Rusty‐KL‐B and Rusty‐KL‐C as on Rusty (Table 5) at both Fargo and St Paul, but was avirulent on Rusty‐ST464‐C1, Rusty‐14803 and PI 387696. This was true regardless of temperature (Figures 5 and S5). These results indicated that TCMJC was virulent on R1 lines but avirulent on the R3 (Rusty‐ST464‐C1) and R2 (*T*. *carthlicum* PI 387696 and Rusty‐14803) haplotypes.

**Table 5:** Infection types (ITs) observed on nine tetraploid genetic stocks with races THTSC and TCMJC of *Puccinia graminis* f. sp. *tritici* at Fargo, ND and St Paul, MN, USA

Line	*Sr13* haplotype	THTSC^a^		TCMJC^a^		TCMJC^b^		TCMJC^c^	
		25℃	GH	25℃	GH	25℃	22℃	25℃	22℃
Rusty		4	4	43	43	3^+^	3^+^	3^+^	3^+^
Wells	R1	14X	12	34	34/23	32^+^	3	33^+^	33^+^
Lakota	R1	123X	12^−^	34	34	2^+^	3	3^+^	3^+^
Rusty‐KL‐B	R1	33^−^	33^−^	34	34	3^+^	3^+^	3^+^	3^+^
Rusty‐KL‐C	R1	33^−^	33^−^	34	34	3^+^3	3^+^	3^+^	3^+^
Rusty‐14803	R2	2	23^−^	2^+^	2^+^3^−^	2^+^	2^+^	2^+^	2^+^
PI 387696	R2	2	2	2	2^+^	2	2	2^+^	2^+^
Rusty‐ST464‐C1	R3	22^−^	2	2^+^	3^−^2	2^+^	2^+^	2^+^	2^+^
Langdon	R3 +	1^−^	1^=^	2^+^	2	22^+^	22^+^	22^+^	22^+^

22℃ and 25℃ are growth chamber trials conducted with daylight temperatures as indicated. GH is a greenhouse trial set at 21℃ but where temperatures are not stringently maintained. ITs: 0, fleck (;), 1, and 2 are resistant reactions, while 3 and 4 are susceptible reactions. Plants may have combinations of ITs, for example IT 32 is primarily IT 3 with smaller amount of IT 2. Plus (^+^), minus (^−^), and double minus (^=^) signs indicate larger, smaller and much smaller pustules than normally seen for that IT (Stakman *et al*., 1962). C = more chlorosis than normal, n = necrosis. A slash indicates heterogeneous infection types on different plants of a population while X indicates a highly heterogeneous infection type on a single leaf.

^a^ Fargo, ND.

^b^ Trial 1 at St Paul, MN.

^c^ Trial 2 at St Paul.

Additional tests of THTSC and TCMJC were conducted at St Paul, along with tests of TTKSK, TRTTF and JRCQC (Table 6, Figure S6). In these tests, the Rusty monogenic lines were compared with Kronos (R1 haplotype), Kronos ethyl methanesulfonate (EMS) mutant (T_4_ lines), Fielder and a Fielder transgenic (T_2_) line with R3 previously reported by Zhang *et al*. (2017). Over three replications, THTSC produced resistant ITs on Kronos, possibly indicating that a second gene in Kronos conferring resistance to TRTTF (Zhang *et al*., 2017) is also effective against THTSC. The Rusty‐KL‐B and Rusty‐KL‐C monogenics also showed a higher level of resistance to THTSC than to TCMJC. TCMJC consistently produced susceptible ITs of 33^+^ or 3^+^ on R1 genotypes Kronos, Rusty‐KL‐B and Rusty‐KL‐C, whereas R2 and R3 genotypes were resistant except for some higher readings on the third replication of T_2_Sr13‐2. Excluding the Kronos T_4_ mutants, for TTKSK and TRTTF all R1, R2 and R3 genotypes had resistant ITs, consistent with previous studies. For JRCQC, we observed that the Rusty‐14803 line (R2 genotype) was susceptible, whereas the R1 and R3 genotypes were resistant regardless of background (Table 6), although there were once again some higher readings with Rusty‐KL‐B and Rusty‐KL‐C.

**Table 6:** Infection types (ITs) observed on 12 genetic stocks when tested with five races of *Puccinia graminis* f. sp. *tritici* at St Paul, MN, USA

Genotype	Haplotype	THTSC			TCMJC			TTKSK			TRTTF			JRCQC		
		1	2	3	1	2	3	1	2	3	1	2	3	1	2	3
Fielder		3^+^	3^+^	33^+^	3^+^	3^+^	3^+^	3^+^	3^+^	3^+^	3^+^	3^+^	3^+^	0;3	0;3	0;
T_2_Sr13‐2^a^	R3	22^+^	22^+^	2/22^+^/3^+^	2^+^3	2^+^3	32^+^/3^+^	2^+^	2^+^	2^+^3	2	22^+^	2^+^3	0;3	0;3	0;
Kronos	R1	22^+^	2	12	33^+^	33^+^	33^+^	22^+^	2	2^−^	2	2	12^−^	2	2	22^+^
T_4_‐3102^a^	R1 mutant	33^+^	33^+^	33^+^	33^+^	33^+^	3^+^	3^+^	3^+^	3^+^	22^+^	2	2^−^	33^+^	33^+^	3^+^
T_4_‐771^a^	R1 mutant	33^+^	33^+^	–	3^+^	33^+^	–	3^+^	3^+^	–	22^+^	22^+^	–	33^+^	33^+^	–
Langdon	R3	;1	;1	–	12	12	12^−^	22^+^	22^+^	–	12	12	12^−^	22^+^	2^+^	32^+^
Rusty‐KL‐B	R1	2^+^3	2^+^3	2^+^3	33^+^	33^+^	3^+^	22^+^	22^+^	22^−^	2	2	2	22^+^	2^+^	32^+^
Rusty‐KL‐C	R1	32^+^	32^+^	2^+^3	33^+^	3^+^	3^+^	22^+^	22^+^	22^−^	2	22^+^	2	32^+^	2^+^3	32^+^
Rusty‐ST464‐C1	R3	2	2	–	2	2	–	22^+^	22^+^	–	2	2	–	22^+^	22^+^	–
Rusty‐14803	R2	2	22^+^	–	22^+^	22^+^	2^+^	2	2	12^−^	2	22^+^	12^−^	3^+^	3^+^	3^+^
Rusty		3+	3^+^	3^+^	33^+^	3^+^	3^+^	33^+^	3^+^	33^+^	33^+^	33^+^	3^+^	3^+^	3^+^	3^+^

Infection types were scored by following Stakman *et al*. (1962) as described in the footnotes in Tables 4 and 5.

^a^ The T_2_ transgenic has *Sr13* transformed into a Fielder background. The T_4_‐3102 and T_4_‐771 lines are ethyl methanesulfonate mutants of Kronos carrying premature stop codons in CNL13 (Zhang *et al*., 2017) and thus loss of function of *Sr13*.

We evaluated three races (TCMJC, THTSC and TTKSK) for pustule size on four genotypes (Table S9, Figure S6). Fielder was compared with the T_2_‐Sr13 Fielder‐background transgenic line (R3 genotype), and Kronos (R1 genotype) was compared with the Kronos mutant T_4_‐3102 genotype. For TTKSK, significantly smaller pustule sizes were observed on both Kronos and T_2_‐Sr13, indicating that both the R1 and R3 haplotypes were effective. For THTSC, pustules on Kronos were significantly smaller than those observed on T_4_‐3102, opposite of what would be expected for an R1‐virulent race. This might also indicate that a second gene in Kronos (Zhang *et al*., 2017) was effective against THTSC. Addition of *Sr13* to Fielder in T_2_‐Sr13 resulted in a decrease in average pustule size with THTSC, although the *P*‐value was 0.015. The most significant comparison of the pustule measurements was Kronos versus T_4_‐3102 for TCMJC, where Kronos produced a higher average pustule size of 3.16 mm^2^ compared with 2.75 mm^2^, but the result was not significantly different. At the same time, average pustule size was reduced on the T_2_‐Sr13 transgenic line compared with Fielder (3.92 versus 5.68 mm^2^; *P* = 0.049). These results clearly indicate that TCMJC was virulent on R1 genotypes and avirulent on R3 genotypes, supporting the results from screening of the Rusty monogenic lines.

### Comparison of stem rust resistance in CAT‐A1 and *Sr13* monogenic lines

Nine monogenic lines were tested at Fargo in 1995 and St Paul in 2008 (Table S10) with 22 North American and four African races. Based on the 1995 test, CAT‐A1 was not identified as a line of interest and was not included in the 2008 test. The significance of the 1995 test was that the R1 genotypes were susceptible to THTSC and TCMJC, the R2 genotypes were susceptible to RCCNC, QCCJC, QFCSC and perhaps one isolate of QTHJC, the R4 haplotype was susceptible to THTSC, TCMJC, TCCJC and TPPKC (T group) and the R3 genotypes were resistant to all the races. In addition to testing these lines over a wide range of races, these tests were used as the basis for selection of races to test whether R4 belonged to the *Sr13a*, *Sr13b* or *Sr13c* alleles, or whether it should be assigned a new allele. While the data in Table S10 indicate that R4 is neither *Sr13b* nor *Sr13c*, additional data were needed for the R1 versus R4 comparison.

Three trials were conducted at Fargo comparing CAT‐A1 with eight monogenic lines using nine *Pgt* races at two temperatures (Table S11). All of the monogenic lines except CAT‐B1 carried a *Sr13* haplotype. CAT‐B1 was included in the test to more fully account for the resistance in the parental line Camadi, which was also included in the trial. Resistance in CAT‐B1 was similar to CAT‐A1 with the exception that CAT‐B1 was susceptible to all races in the T group. In each trial, the R3 genotypes were resistant to all nine races, the R2 genotypes were susceptible to QFCSC and QCCJC and showed variable results to QTHJC, and the R1 genotype was susceptible to TCMJC and also showed high ITs in two trials to TCCJC. A summary of these trials is shown in Table 7. These results were identical or similar to results shown in Table S10.

**Table 7:** Infection types (ITs) observed when CAT‐A1 and 10 other genotypes were tested with nine races of *Puccinia graminis* f. sp. *tritici* at 25℃ and 20℃ at Fargo, ND, USA

Genotype	Haplotype	QFCSC		QCCJC		TCCJC		TCMJC		JRCQC		TTKSK	
		20°C	25°C	20°C	25°C	20°C	25°C	20°C	25°C	22°C	25°C	22°C	25°C
Rusty	–	4	34	4	34	4	34	34	34/23	4	4	4	3^+^
KL‐B	R1	32	2^+^	2^+^	23	32	23	34	34	2^+^	2^+^	22^+^	2
Rusty‐14803	R2	34	34	34	34	22^+^	12	2^+^	21	3^+^	33^+^	2	2
Im‐C2	R2	4	34	34	34	2^–^/2^+^	12c	2^+^	21	4	4	2	2
Im‐B7	R2	4	34	34	34	2^−^	12	2	1^+^c	3^+^	33^+^	2	2^−^
8155‐B2	R3	2^−^	2c	2^−^	1^+^c	2^−^	2^−^	2^+^	21	2	2	2	22^−^
8155‐C1	R3	2	2c	2^−^c	2^−^/0;1^−^	2^−^/2	2^−^	2/2^+^	21	2^−^	22^−^	2^−^	2^−^
ST464‐C1	R3	2^+^	22^+^c	2^−^c	1^+^c	22^+^/2	2/23	2^+^	21	2^+^	2^+^	2^+^	2^+^
CAT‐A1	R4	2^+^	23/34	2^−^/2^+^	2	34	34	34/2^+^3	32	2^+^	2^+^	3	3
CAT‐B1	non‐*Sr13*	1^−^	2^−^c	0;1/1	1cn	34	32	34	23/34	3	3^+^	3^+^	3^+^
Camadi‐1^a^	R4	0;1^−^	1^+^c^+^	0;	2	2^−^	34/23	34/2^+^3	1/34	33^+^	3^+^	33^+^c	3

Infection types were scored by following Stakman *et al*. (1962) as described in the footnotes of Tables 4 and 5.

^a^ Camadi Abdu tipo #103.

Parental line Camadi had not been identified as having resistance to race TTKSK. Thus, we questioned whether CAT‐A1 would be susceptible to TTKSK. We grew individual plants of CAT‐A1 and Camadi for testing with marker *rwgsnp37.2*. All three plants of CAT‐A1 tested were positive for *Sr13* (Table S12). Of 15 Camadi plants, only six were positive for *Sr13*. Progeny from four of these plants were tested along with the monogenic lines in St Paul to determine if R4 differed from the other *Sr13* haplotypes for resistance to TTKSK and JRCQC. As expected, the monogenic lines carrying R1 and R3 were resistant to JRCQC while the R2 monogenic lines were susceptible (Table S12). The progeny of the R4 genotype (CAT‐A1) were generally resistant, although the progeny of plant #3 were susceptible in the 25°C test (Table S12). Camadi plants #1 and #3 were both positive for the *Sr13* marker *rwgsnp37.2*, yet their progeny were susceptible and resistant, respectively, to both TTKSK and JRCQC (Table S12). This suggests that a gene other than an allele at the *Sr13* locus was conditioning resistance in Camadi #3. CAT‐A1 also showed some variability in response to TTKSK, though not as variable as Camadi (Table S12). Two of the three CAT‐A1 sources (plants #1 and #3) were susceptible to TTKSK, while plant #2 had an intermediate (2^+^3) IT. In contrast, all monogenic lines having R1, R2 or R3 genotypes had resistant infection types. These results indicated that the R4 haplotype differed from the R1, R2 and R3 haplotypes in its reaction to TTKSK; it was therefore designated as a new allele, *Sr13d*.

### Validation of STARP markers

Two STARP markers *rwgsnp6* and *rwgsnp7*, which are closely linked to the *Pgt* resistance locus (*QSr.rwg‐6A.3*) in PI 387696, were analyzed on a diverse set of 16 durum and 30 common wheat cultivars. The complete results for all cultivars/genotypes and markers are shown in Table S13 and a summary of tetraploid genotypes is shown in Table 8. Marker *rwgsnp6* amplified the Rusty allele (A1) in three durum and one common wheat cultivars/lines and the remaining 41 cultivars/lines had the PI 387696 allele (A2) (Table S13 and Figure S7). For marker *rwgsnp7*, the Rusty allele (A1) was present in only one durum cultivar, and 19 cultivars/lines had the PI 387696 allele (A2) (Table S13 and Figure S7). In addition, 25 common wheat cultivars/lines had both Rusty and PI 387696 alleles (A1A2), which might be the homeoalleles from different chromosomes detected by *rwgsnp7*. Based on these validation data, 98% of durum and common wheat cultivars/lines carried the PI 387696 allele (A2). When tested with *rwgsnp37.2*, none of the hexaploid wheats in the validation set carried *Sr13* (Table S13, Figure S2). Therefore, for validation of *rwgsnp38, rwgsnp39* and *rwgsnp40*, 22 additional durum wheats were added to the analysis. Based on the results with *rwgsnp37.2*, 10 durum cultivars and lines in the tests did not carry *Sr13* (Table 8). The importance of *Sr13* in North Dakota durum production is illustrated by the fact that since 1960 there have been 32 durum cultivars released, and *Sr13* was absent in only six of those cultivars, namely, Crosby, Edmore, Monroe, Belzer, Dilse and Divide. Among the North Dakota durum cultivars, when considering the *rwgsnp38*, ‐*39* and ‐*40* data, the most striking observation was that the cultivars tended to have the R2 haplotype (*Sr13b)*, and this may reflect the influence of Wells/Lakota virulence because both the R2 and R3 genotypes were resistant to TCMJC and THTSC.

**Table 8:** Summary of the haplotype/alleles at the *Sr13* locus observed among the tetraploid wheat cultivars and genotypes tested during this study^a^

Haplotype	Allele	Cultivar/line
R1	*Sr13a*	Khapli (CItr 4013), Rusty‐KL‐B, Rusty‐KL‐C, Strongfield, Transcend, Grenora, Wells, Lakota, Cando, Mountrail, Durox, Kronos
R2	*Sr13b*	Im‐B7, Im‐C2, Leeds, Svevo, D151343, ND Grano, ND Riveland, Joppa, Lebsock, Carpio, Ben, Pierce, Ward, Rugby, Botno, Munich, Calvin, Vic, Plaza, Lloyd, Tioga, Sceptre, Medora, CItr 7777, PI 387696
R3	*Sr13c*	CItr 8155, 8155‐B2, 8155‐C1, ST464, Rusty‐ST464‐C1, Altar 84, PI 352548, Langdon, D101073, Alkabo, CItr 7771
R4	*Sr13d*	Camadi Abdu tipo #103, CAT‐A1
S	Null	Carleton, Stewart, Ramsey, Cappelli, Rusty
S	False positive	Divide, Monroe, Dilse, Edmore, Belzer, Crosby, CItr 7780

^a^ Detailed results including common wheat cultivars are shown in Table S13.

## DISCUSSION

In North American and CIMMYT (International Maize and Wheat Improvement Center) durum cultivars/lines, *Sr13* alleles are the main component of resistance effective against the Ug99 race group and other prevailing *Pgt* races. They have been found in many cultivars including Kronos, Kofa, Medora, Sceptre, Langdon, Wells, Leeds, Lebsock, Joppa and Carpio (Klindworth *et al*., 2007; Simons *et al*., 2011; Zhang *et al*., 2017; Saini *et al*., 2018). Zhang *et al*. (2017) showed that the *Sr13* functional gene *CNL13* had three resistant haplotypes R1, R2 and R3. Based on the reaction to *Pgt* race JRCQC, the resistant (R1/R3) and susceptible (R2) haplotypes were designated as *Sr13a* and *Sr13b*, respectively (Zhang *et al*., 2017). Here we further identified *Sr13b* (R2) in *T. carthlicum* PI 387696 and *T*. *polonicum* CItr 14803. We also confirmed that several durum monogenic lines carry R1, R2 and R3 haplotypes. Based on the SNPs in the LRR region, we developed three robust functional STARP markers diagnostic for the three haplotypes. We differentiated the R3 haplotype from R1 based on the reaction to *Pgt* race TCMJC (Tables 4, 5, 6, 7), thereby identifying allele *Sr13c*. By marker and sequence analysis, we identified a new haplotype R4 in a monogenic durum line CAT‐A1.

We had previously developed the NILs Rusty‐KL‐B (R1), Rusty‐KL‐C (R1) and Rusty‐ST464‐C1 (R3). During this study, we also developed the NIL Rusty‐14803 (R2) derived from CItr 14803. In all four NILs, *Sr13* had been transferred to Rusty through six backcrosses. These lines served as checks for each of the three *Sr13* haplotypes in multi‐pathotype tests. Because we have only recently discovered the R4 haplotype, backcrossing of CAT‐A1 to Rusty to introduce the R4 haplotype into a Rusty background is currently ongoing to generate a near‐isogenic R4 line with a uniform genetic background to eliminate some of the variation observed in the rust tests (Tables 8 and S11).

Two races maintained at Fargo, TCMJC and THTSC, were known to be virulent to lines designated as carriers of *Sr13*, although the specific *Sr13* allele ineffective to these races was not known. Although past records of isolate numbers going back to the 1960s are incomplete, the race reported as 15‐WL by Gough *et al*. (1964) is likely to be TCMJC, based on its virulence on Wells and Lakota. Most importantly, all R1 genotypes were susceptible to TCMJC (Table 5) similar to 15‐WL, but Wells and Lakota expressed a resistant reaction to THTSC, indicating that it was not 15‐WL. In addition, TCMJC produced a larger average pustule size (Table S9) than THTSC. Comparisons of the IT data on Rusty‐KL‐B and Rusty‐KL‐C also supported this conclusion (Tables 5 and 6). The two genotypes carrying *Sr13b*, PI 387696 and CItr 14803, were resistant to TCMJC, as was Rusty‐ST464‐C1, which carried the R3 haplotype. Therefore, TCMJC is important in separating the R3 haplotype of ST464 from the R1 haplotype of Khapli. Wells/Lakota virulence is also important because both TCMJC and THTSC are still used today as part of the bulk of isolates inoculated for selection of cultivars in North Dakota, and thus may select against genotypes carrying the *Sr13a* allele.

Khapli emmer and landrace ST464 were the sources of the *Sr13* gene in tetraploid wheat (Knott, 1962; Klindworth *et al*., 2007). Zhang *et al*. (2017) reported that Khapli, Langdon, ST464 and Leeds, had *Sr13* haplotypes R1, R3, R3 and R2, respectively. We confirmed this result, but this presented a problem in that Langdon was reported to derive stem rust resistance from Khapli (Heyne, 1959) and should therefore have the R1 haplotype. Meanwhile, based on its pedigree, Leeds could have derived *Sr13* from either ST464 or Wells (Lebsock *et al*., 1967) and should therefore have either the R1 or R3 haplotype, but instead has the R2 haplotype. Our haplotyping included Langdon parents Carleton and Stewart (Table 8), and they clearly did not contribute *Sr13* to Langdon, a result that agreed with the data of Gough *et al*. (1964). Therefore, we agree that Langdon could only have obtained *Sr13* from Khapli. An explanation for the inconsistency between pedigrees and haplotypes could be due to several factors during breeding, including genotype heterogeneity, seed/pollen mixtures, planting/harvesting errors or record keeping errors. We examined the pedigrees of all North Dakota durum cultivars to try to determine if any other cultivars exhibited inconsistencies (Table S14). To do this, we used published pedigrees and haplotyping of cultivars from this study or from Zhang *et al*. (2017). Comparing haplotypes with pedigrees, other than Langdon and Leeds the only other cultivar with an inconsistent R haplotype was Lloyd. Lloyd had the R2 haplotype, and its parents, Cando and Edmore, had the R1 and S haplotype, respectively.

In addition to investigating the haplotypes of current cultivars, we were interested in determining the origin of the R2 haplotype in durum cultivars. The discovery that Im‐C2 and Im‐B7 carried the R2 haplotype suggested that perhaps Iumillo was the source of R2; however, we were unable to confirm the presence of the R2 haplotype in Iumillo. Furthermore, we know of no one who has suggested that *Sr13* was among the several putative *Sr* genes present in Iumillo. CItr 7780 is found in the pedigrees of Ward, Rugby and Botno (Table S14) and reportedly has stem rust resistance (Quick *et al*., 1975). We found CItr 7780 did not carry *Sr13* (Tables 8, S13 and S14), therefore this line was not the source of R2 in durum.

In this study, we identified six new molecular markers including one KASP marker and five STARP markers in the *Sr13* LRR region. Because the STARP markers *rwgsnp6* and *rwgsnp7* were not developed from the *CNL13* sequence they are not perfect markers and their utility may be limited to mapping of the *Sr13* region. In contrast, both *KASPSr13* and *rwgsnp37* are perfect markers for *Sr13* and should be preferred markers for geneticists and breeders. We also developed STARP markers *rwgsnp38*, *rwgsnp39* and *rwgsnp40* to discriminate the *Sr13* haplotypes. *Rwgsnp38* differentiates R2 from the R1/R3/R3 haplotypes, *rwgsnp39* differentiates R3 from the R1/R2/R4 haplotypes and *rwgsnp40* differentiates R1 from the R2/R3/R4 haplotypes. All three of these markers detect some false positives which must be identified via either *rwgsnp37* or *KASPSr13*.

Zhang *et al*. (2017) concluded that haplotypes R1 and R3 formed a single allele, *Sr13a*. Our data establish that R1 and R3 are clearly different alleles. When TCMJC was tested on NILs Rusty‐KL‐B (R1), Rusty‐KL‐C (R1) and Rusty‐ST464‐C1 (R3) (Table 5, Figure 5), the R1 genotypes were susceptible but the R3 genotype was resistant, with the exception of one test. When comparing the Fielder transformation line T_2_Sr13‐2 with Fielder in tests with TCMJC (Table 6), the transgenic line had lower ITs and consistently resistant ITs in two of the three replications. In the same test, Kronos and all other R1 genotypes were susceptible, whereas the R3 Rusty‐ST464‐C1 was resistant. Transformation of Fielder with R3 also resulted in decreased pustule size when plants were inoculated with TCMJC (Table S9). However, when Kronos (R1+) and the mutant T_4_‐3102 (R1−) were inoculated with TCMJC, no differences in pustule size were observed. This suggested that, contrary to the results obtained with R3, the presence/absence of R1 did not influence pustule size in tests with TCMJC. In addition to these observations, other stem rust data are also consistent with these results. In our efforts to concentrate on Wells/Lakota virulence, we neglected to study a more recent race which may differentiate R3 from R1. The data from the test in 2008 show that the KL‐B (R1) and KL‐C (R1) monogenic lines were susceptible to TTTTF, whereas R2 and R3 genotypes were resistant (Table S10).

Zhang *et al*. (2017) noted that a polymorphism in the *CNL13* sequence of *Sr13* at position T2200C resulting in the amino acid change W734R was perfectly associated with resistance to TTKSK. The discovery of the R4 haplotype and its apparent failure to express resistance to TTKSK despite carrying T2200C/W734R indicated that while the T2200C/W734R polymorphism was crucial for stem rust resistance it was not the only determinant of TTKSK resistance within the *CNL13* sequence. The R1, R2 and R3 polymorphisms at G1963C/G655R, G2272C/V758L, G2517T/W839C and C2798A/T933K also have a positive influence on stem rust response. Because the R4 haplotype carries only the T2200C/W734R polymorphism compared with the susceptible haplotype S1, it is probably the primitive resistant haplotype. R4 might have acquired its resistance function via a point mutation in the S1 haplotype and the other three resistant haplotypes (R1, R2 and R3) were probably derived from R4 through one or two additional point mutations.

Assignment of an allele name to the R4 haplotype is dependent upon stem rust response data clearly differentiating R4 from the other three haplotypes. The data from Miller *et al*. (Table S10) showed that CAT‐A1 (R4) differed from R2 genotypes with QFCSC, QCCJC, QTHJC and RCCNC, whereas CAT‐A1 differed from R3 genotypes with four of six T‐races. Our tests confirm these observations (Tables 7 and S11) and clearly establish that R4 is different from R2 and R3. Differentiating R4 from R1 is a bit more difficult, primarily because it relies on fewer races. Among the two differential races reported by Miller *et al*. (Table S10), we confirmed differences from TCCJC (Tables 7 and S11), but differences from TPPKC were less clear. In addition, we observed susceptible ITs on CAT‐A1 when inoculated with TTKSK (Table S13). Based on these results, the R4 haplotype is designated as allele *Sr13d*.

In this study, we first identified accessions of *T*. *carthlicum* and *T*. *polonicum* that carried the *Sr13* R2 haplotype using gene mapping and markers diagnostic for specific *Sr13* haplotypes. In rust tests involving these two lines with check tetraploid lines we showed that the R2 genotypes were susceptible to QFCSC whereas the R1/R3 genotypes were resistant. We then developed STARP markers that could differentiate the R3 haplotype and clearly demonstrated that the R3 haplotypes were resistant to TCMJC whereas R1 haplotypes were susceptible. Therefore, the R1 and R3 haplotypes were separated and designated as *Sr13a* (Khapli source) and *Sr13c* (ST464 source), respectively. As the R2 haplotype retains the original allele symbol *Sr13b* (Zhang *et al*., 2017), a total of four *Sr13* alleles (*Sr13a*, *Sr13b*, *Sr13c* and *Sr13d*) have been identified. Extensive stem rust testing showed that *Sr13c* is the strongest allele with no virulent races detected so far and should be a preferred allele for breeding resistance to stem rust, whereas *Sr13d* should be avoided in breeding due its susceptibility to TTKSK. The allele‐specific functional markers developed in this study are useful not only for detecting these alleles in the base germplasm and cultivars but also for facilitating the pyramiding of a specific allele with other *Sr* genes in durum and bread wheat breeding.

## EXPERIMENTAL PROCEDURES

### Plant materials

*Triticum carthlicum* accession PI 387696 and *T*. *polonicum* accession CItr 14803 were obtained from the USDA‐ARS National Small Grain Collection (Aberdeen, ID, USA). For PI 387696, a population of 190 RILs was developed by crossing PI 387696 to durum Rusty (PI 639869) (Klindworth *et al*., 2006), followed by single‐seed descent through the F_7_ generation. The *T*. *polonicum* accession was also crossed to Rusty but the F_1_ plants were completely sterile. Rusty was therefore used as the male parent in a backcross to the F_1_ plants. Nineteen BC_1_F_1_ plants were tested for reaction to stem rust, and 10 resistant plants were grown to maturity. Seedset on the BC_1_F_1_ plants ranged from 1 to 132 seeds, and the three plants having the highest seedset, 99 seeds or more, were selected for genetic analysis.

Additional genetic stocks used included KL‐B, KL‐C, ST464‐C1, ‘Langdon’ (CItr 13165), ‘Wells’ (CItr 13333), ‘Lakota’ (CItr 13335) and Medea Ap9d. KL‐B, KL‐C and ST464‐C1 are durum lines monogenic for stem rust resistance. Langdon and Medea Ap9d carry multiple *Sr* genes (Knott, 2000). Langdon derives stem rust resistance from Khapli emmer (Heyne, 1959) and therefore carries *Sr13*. Medea Ap9d carries *Srdp2* (Roelfs and McVey, 1979), which was thought to be allelic to *Sr13* (McIntosh *et al*., 1995), but it was later concluded (Zhang *et al*., 2017) that *Srdp2* could not be an allele of *Sr13*. Stem rust resistant lines, mostly monogenic, which were developed by the USDA‐ARS at Fargo, ND (Table S1) were also available for screening. Durum cultivars used for haplotyping were mostly available from local USDA‐ARS stocks; however, in a few instances, cultivars were obtained either from the USDA‐ARS National Small Grain Collection or North Dakota State University (Fargo, ND).

### Pathotypes of stem rust pathogen

Multiple *Pgt* races were used in the course of this study. The main races used were TMLKC, THTSC, TCMJC, TRTTF and TTKSK. The isolate number, origin and avirulence/virulence formula for these races, and 26 other races, are shown in Table S15. Some of the races listed were used only for screening of monogenic lines and three (TKTTF (Eth), TKTTF (Ger) and TTRTF) were not part of this study but their avirulence/virulence formulas are shown because these races are mentioned in the text.

### Development of NILs in the Rusty background

The potential tetraploid sources of *Sr13* including ST464‐C1, KL‐B and KL‐C were included in a backcrossing program to introduce these single genes into the Rusty background. In addition, because a BC_1_ population (CItr 14803/2*Rusty) had already been produced, we opted to include the gene from *T*. *polonicum* in the backcrossing program. Each line was crossed and backcrossed to Rusty to produce the BC_1_ generation. Beginning in the BC_1_ generation, we tested 10–15 hybrids in each generation with *Pgt* race TMLKC, selecting resistant hybrids for backcrossing to Rusty until we reached the BC_6_ generation. We selfed the BC_6_F_1_ and BC_6_F_2_ generations, and progeny tested approximately 25 BC_6_F_3_ plants to identify homozygous families. From the four tetraploid sources, we selected and named four NIL lines: Rusty‐ST464‐C1, Rusty‐KL‐B, Rusty‐KL‐C and Rusty‐14803.

### Identification of the *Sr* gene in *T. carthlicum* PI 387696

To identify the *Sr* gene in PI 387696, the F_1_ hybrid, the F_2_ and the RIL (F_7_) populations derived from the cross Rusty × PI 387696, along with their parents, were first evaluated for reactions to *Pgt* races TMLKC, TTKSK and TRTTF. Stem rust evaluation with races TTKSK and TRTTF were conducted at the USDA‐ARS at St Paul, Minnesota, USA using the protocol reported by Rouse, Wanyera *et al*. (2011). Testing of race TMLKC was conducted at the USDA‐ARS at Fargo, ND using the procedure described in Klindworth *et al*. (2012). The stem rust disease was evaluated using the 0–4 scale reported by Stakman *et al*. (1962). Plants with IT 2 or lower were considered resistant while plants with IT 3 or greater were considered susceptible. For linkage analysis, the original seedling IT scores were converted to a 0–9 linear single‐value disease scale using the method described by Zhang *et al*. (2014). The linearized scale values for the RIL population and parents were further utilized for QTL analysis.

For genotyping analysis, the RIL population and parents were genotyped using the wheat Infinium 90K SNP iSelect array (Illumina Inc., https://www.illumina.com/). The SNP genotyping assay was carried out on the iScan instrument following the manufacturer’s protocols (Illumina Inc.). The SNP markers were designated as ‘IWA’ and ‘IWB’ followed by their index number, as suggested in Wang *et al*. (2014). The SNP genotype calling was performed using the polyploidy clustering module of GenomeStudio v.2011.1 software (Illumina Inc.) and the accuracy of SNP clustering was visually inspected. Incorrect clustering was manually manipulated for accuracy of SNP genotype calling. Approximately 74 SSR primer sets including 23 WMC (Somers *et al*., 2004), 2 CFA and CFD (Sourdille *et al*., 2004), 25 BARC (Song *et al*., 2005), 1 CFB, 1 GPW (Sourdille *et al*., 2004) and 19 GWM (Röder *et al*. 1998), that detected markers with known chromosome locations, were selected to confirm genetic linkage groups representing particular chromosomes. The selected SSR set was checked for polymorphism between Rusty and PI 387696 using the PCR conditions and protocol by Sharma *et al*. (2019). Molecular markers that showed the polymorphism between the two parents were used to genotype the 190 RILs and used for linkage map construction.

The polymorphic SNP and SSR markers identified above were used to construct a high‐density linkage map covering all 14 chromosomes of tetraploid wheat using the computer program MapDisto v.1.8.2.1 (Lorieux, 2012) as described by Sharma *et al*. (2019). The genotypic and phenotypic data of the RIL population were used in QTL analysis. The critical LOD threshold was calculated using a permutation test with 1000 iterations. For QTL analysis, single‐trait multiple interval mapping (SMIM) was carried out using the computer program QGene 4.3 (Joehanes and Nelson, 2008). After a QTL at the *Sr13* locus was identified, the 190 RILs and their parents were further genotyped with the *Sr13* gene‐specific KASP marker *KASPSr13* (Table 1) by following the procedure described in Nirmala *et al*. (2017). The primers of *KASPSr13* were designed by LGC Genomics LLC (https://www.lgcgroup.com/) based on the cloned *Sr13* *CNL13* (Zhang *et al*. 2017).

### Identification of the *Sr* gene in *T*. *polonicum* CItr 14803

Prior to initiating bulk segregant analysis, Rusty and CItr 14803 were screened for polymorphism against a set of 1037 SSR markers. The publicly available markers included SSR primer sets described above (WMC, CFA, CFD, BARC, GDM and GWM markers) and the DUPW set (Eujayl *et al*., 2002). Because the F_1_ plants from the cross CItr 14803 × Rusty were sterile, we used 180 BC_1_F_2_ progeny from the three most fertile BC_1_F_1_ plants (CItr 14803/2*Rusty) for bulk segregant analysis. The BC_1_F_2_ progeny were screened for resistance to race TMLKC, and DNA from each plant was extracted and stored for future use. BC_1_F_3_ seed was harvested from each plant and BC_1_F_2_ families were then screened with races TMLKC and TTKSK to determine the genotype of each BC_1_F_2_ plant. For this rust test, we tested approximately 25 and 5 BC_1_F_3_ plants per family with TMLKC and TTKSK, respectively. Based on the segregation observed among BC_1_F_3_ plants within BC_1_F_2_ families, the original BC_1_F_2_ plant could then be classified as homozygous resistant, heterozygous or homozygous susceptible. A bulk segregant analysis was then prepared by going back to the DNA saved from BC_1_F_2_ plants and pooling DNA from 10 homozygous‐susceptible and 10 homozygous‐resistant plants to form a susceptible and resistant bulks, respectively.

From the list of polymorphic SSR markers, we selected a relatively small set of 67 markers (Table S6). In selecting markers, we used a minimum of two markers from each chromosome arm, with one marker being relatively close to the telomere and one located in a sub‐metacentric area. In addition, we selected markers based on linkage to known *Sr* genes. In particular, markers linked to the *Sr9* and *Sr13* loci were chosen due to the widespread occurrence of these genes in tetraploid wheat. DNA samples of Rusty, CItr 14803 and the susceptible and resistant bulks were used in the bulk segregant analysis. A PCR was conducted, and products were separated and scanned as described in Sharma *et al*. (2019). From the bulk segregant analysis, a marker was identified that was potentially very closely linked to the resistance gene. This marker was tested on the complete set of 180 BC_1_F_2_ plants. The distance of the marker from the rust resistance gene was calculated using the Kosambi function (Kosambi, 1944). Finally, the *Sr13* gene‐specific KASP marker *KASPSr13* was tested on the set of 180 BC_1_F_2_ plants and compared with the rust data.

### Stem rust testing of *Sr13* genotypes

Nine genotypes, Rusty, Rusty‐KL‐B, Rusty‐KL‐C, Rusty‐ST464‐C1, Langdon, Medea Ap9d, PI 387696, Rusty‐14803 and CItr 14803, were tested with 10 local *Pgt* races including QTHJC, QFCSC, RTQQC, RKQQC, RHFSC, TMLKC, TPMKC, TPPKC, THTSC and TCMJC. RTQQC and RKQQC are similar races, differing only by virulence to *Sr11*. TPMKC and TPPKC are also similar races, differing by virulence to *Sr30*. Two of these races, THTSC and TCMJC, are notable because in tests conducted with 21 North American *Pgt* races (Table S10) these were the only two races virulent to KL‐B; and hence virulent to lines thought to carry *Sr13*. One of these two races was originally reported by Gough *et al*. (1964) as race 15‐WL due to its virulence on the cultivars Wells and Lakota; however, because records of isolate numbers are incomplete back to the 1960s, we are unable to positively identify TCMJC or THTSC as 15‐WL. To resolve this, and to generate additional comparisons of the primary *Sr13* monogenic lines, we prepared additional stem rust tests at Fargo and St Paul in which Wells and Lakota were added to the entries. TCMJC and THTSC were tested at Fargo in a growth chamber at a constant 25°C and in a greenhouse maintained at 21°C, while at St Paul, tests were conducted in a growth chamber at a daytime temperature of 25°C and a nighttime temperature reduced by 3°C. All planting and scoring followed the same or very similar procedures to those described above.

An additional trial was conducted at St Paul to compare reactions of the Rusty NILs versus *CNL13* transgenic and mutant lines (Zhang *et al*., 2017). Kronos, Fielder and three CNL lines, T_2_Sr13‐2, T_4_‐771 and T_4_‐3102, were obtained from Dr Jorge Dubcovsky (University of California, Davis). Kronos carries *Sr13* (R1) and probably a second gene (Zhang *et al*., 2017). Two T_4_ lines are EMS‐derived mutants of Kronos that carry a truncated version of *CNL13* conferring stem rust susceptibility. T_2_Sr13‐2 is a transgenic line carrying the *Sr13*‐R3 haplotype from Langdon transformed into a Fielder background. Three replications of these lines and the Rusty NILs were planted and inoculated with races TCMJC, THTSC, TTKSK, TRTTF and JRCQC. Following inoculation, plants were grown in growth chambers at 25/22°C day/night temperatures. To quantify disease development, the sporulation area was determined from images analyzed using the ASSESS V2 software (American Phytopathology Society; Lamari, 2008). Average pustule sizes were calculated and analyzed by *t*‐tests.

### Development and validation of STARP markers

Genotyping with the STARP marker assay was performed using two‐universal priming element‐adjustable primers (PEA‐primers), two asymmetrically modified allele‐specific primers (AMAS‐primers) and one common reverse primer (Long *et al*., 2017). The PEA‐primer sequences used during this study were the same as those reported in Long *et al*. (2017) and AMAS‐primers and the common reverse primer were designed using the method described by Long *et al*. (2017). During this study one new KASP marker, five new STARP markers and the STARP markers *rwgsnp37.1* and *rwgsnp37.2* reported by Saini *et al*. (2018) were used to map and/or determine the identity of the *Sr* genes in PI 387696, CItr 14803 and CAT‐A1. After the resistance locus in PI 387696 was identified as *Sr13* based on the 90K SNP and *KASPSr13* genotyping analysis of the Rusty × PI 387696 RIL population, two 90K SNP markers *IWB34398* and *IWB71956* that were closely linked to *Sr13* were converted to STARP markers *rwgsnp6* and *rwgsnp7*, respectively (Table 1). Three additional STARP markers, *rwgsnp38*, *rwgsnp39* and *rwgsnp40* (Table 1), were then developed to differentiate among R1, R2, R3 and new haplotypes. The primers of these STARP markers were designed by following previous descriptions (Klindworth *et al*., 2017; Long *et al*., 2017; Saini *et al*., 2018). The STARP genotyping assays were performed using 6% non‐denatured polyacrylamide gels and the CFX384^TM^ Real‐Time System (Bio‐Rad Laboratories, Inc., https://www.bio‐rad.com/) following the protocol in Long *et al*. (2017). The five new STARP markers were validated using a diverse set of 16 durum and 32 common wheat cultivars or lines originating from different geographical regions.

To test monogenic lines for the presence of *Sr13*, leaf tissue was sampled from the monogenic stem rust resistant lines maintained at USDA‐ARS, Fargo, ND. Table S1 indicates the resistant parent of each set, the number of lines in each set and a designation for each set (e.g. KL for Khapli). The DNA was extracted and the monogenic lines were evaluated for the presence of *Sr13* using marker *KASPSr13* on a Roche Light Cycler 480II real time PCR system (Roche Diagnostics Corp., https://www.roche.com/). Those monogenic lines shown to be positive for *Sr13* were then tested to determine haplotype using STARP markers *rwgsnp37.2* (*or rwgsnp37.1*), *rwgsnp38*, *rwgsnp39* and *rwgsnp40*.

### *Sr13* sequence analysis

Sequence analysis of *Sr13* was performed in eight lines including Rusty‐KL‐B, Rusty‐KL‐C, Rusty‐14803, Rusty‐ST464‐C1, CAT‐A1, PI 387696, 8155‐B2 and Im‐C2. Four pairs of primers developed in Zhang *et al*. (2017), including 6ACNL13F7/R2, 6ACNL13F4/R7, 6ACNL13F3/R8 and 6ACNL13F5/R6, whose amplicons covered the complete coding and intron sequences of *Sr13*, were used to generate PCR products for DNA sequencing. The PCRs were conducted in a total volume of 15 µl that contained 0.3 µl each of forward and reverse primers (10 µm), 0.5 µl of dNTP mixture (10 mm), 1.2 µl MgCl_2_ (25 mm), 1 unit of Taq polymerase (New England Biolabs Inc., https://www.neb.com/) and 50–100 ng of template DNA. The amplification was performed according to Zhang *et al*. (2017) at 94°C for 3 min, followed by 38 cycles each consisting of 20 sec at 94°C, 20 sec at 58°C and 80 sec at 72°C. The PCR products were treated using an Exo‐CIP™ Rapid PCR Cleanup Kit (New England Biolabs Inc.) to degrade residual primers and dephosphorylate excess dNTPs, and then sequenced at the DNA Facility at Iowa State University, Ames, IA. The *Sr13* sequence in each line was then compared with that of Langdon using the BLASTn Suite (https://blast.ncbi.nlm.nih.gov) to identify SNPs among different *Sr13* haplotypes.

## AUTHOR CONTRIBUTIONS

SSX, DLK, YJ and MNR conceived the study and designed the experiments. DLK, QZ and SSX developed the mapping populations and new monogenic and near‐isogenic lines. SC genotyped the Rusty × PI 387696 population using the wheat 90K SNP iSelect assays and KASP marker. BKG, DLK, SZ, TLF and SSX conducted stem rust tests using North American races at Fargo, ND. MNR, PDO and YJ conducted the stem rust tests using TTKSK and other races at St Paul, MN. BKG, DLK, JSS, JDF, XC and SSX constructed linkage maps and conducted linkage analysis of stem rust resistance. CC and SL conducted sequencing analysis. JZ, DLK, BKG, YL, JDF and SSX developed STARP markers. SC developed marker *KASPSr13* and JDF screened monogenic lines with *KASPSr13*. EME provided plant materials. DLK, BKG and SSX wrote the first draft of the manuscript and all authors contributed to the final draft.

## CONFLICT OF INTEREST

None of the authors have any conflict of interest.

## ETHICAL STANDARDS

The experiments were performed in compliance with the current laws of United States of America.

## Supporting information

**Figure S1**. Analysis of two STARP markers (*rwgsnp6* and *rwgsnp7*) on a recombinant inbred line population and parents (Rusty, *Triticum* *turgidum* subsp. *carthlicum* PI 387696) using the CFX384^TM^ Real‐Time System.

**Figure S2**. Polyacrylamide gel‐electrophoresis of STARP markers *rwgsnp37.2* (a), *rwgsnp38* (b), and *rwgsnp39* (c) testing 16 tetraploid and 32 hexaploid wheat cultivars or genotypes.

**Figure S3**. Infection types observed on nine tetraploid genotypes inoculated with *Puccinia graminis* f. sp. *tritici* races QFCSC, QTHJC, THTSC and TCMJC and incubated at 25°C.

**Figure S4**. Infection types observed on 11 tetraploid genotypes inoculated with *Puccinia graminis* f. sp. *tritici* races THTSC and TCMJC and incubated at either 25°C or in a greenhouse maintained at 21°C.

**Figure S5**. Infection types observed on nine durum genotypes inoculated with stem rust (*Puccinia graminis* Pers. f. sp. *tritici*) race TCMJC and incubated at 25°C in two replications with two leaves per replication shown per genotype.

**Figure S6**. Infection types observed on four genotypes inoculated with races THTSC, TCMJC and TTKSK of *Puccinia graminis* Pers. f. sp. *tritici*.

**Figure S7**. Validation of two STARP markers (*rwgsnp6* and *rwgsnp7*) on 48 common and tetraploid wheat cultivars and lines.

**Table S1**. Stem rust resistant lines developed at Fargo, ND, USA by N. D. Williams and maintained by the USDA‐ARS and their status when tested for presence of *Sr13* using marker *KASPSr13*.
**Table S2**. Segregation of resistance to three races of *Puccinia graminis* f. sp. *tritici* in the F_2_ and recombinant inbred line populations derived from the cross Rusty × *T. turgidum* subsp. *carthlicum* PI 387696.

**Table S3**. Molecular markers mapped in the population of 190 recombinant inbred lines derived from cross durum (Rusty) × *T. turgidum* subsp. *carthlicum* (PI 387696).

**Table S4**. Details of markers mapped in each chromosome/genome in the Rusty × PI 387696 recombinant inbred line population.
**Table S5**. Coordinate positions (bp) of the markers associated with *Sr13* on chromosome 6A linkage map in the reference genome RefSeq v1.0 of common wheat (IWGSC, 2018).
**Table S6**. List of 67 simple sequence repeat markers used in bulked segregant analysis of a stem rust resistance gene derived from *Triticum turgidum* subsp. *polonicum* and size (bp) of the amplicon from each parent.

**Table S7**. Sequences of the *Sr13* coding regions in eight lines tested (sequence of Langdon was downloaded from Zhang *et al*. (2017)).

**Table S8**. Segregation and haplotyping for *Sr13* in historical landraces of tetraploid wheat.
**Table S9**. Analysis of pustule size when four genotypes where inoculated with three races of *Puccinia graminis* f. sp. *tritici* at St Paul, MN, USA.
**Table S10**. Stem rust infection types recorded on nine monogenic tetraploid wheat lines developed by N. D. Williams and tested on 21 stem rust races by Miller, Williams and Klindworth at Fargo, ND, in 1995 and on five races by Yue Jin at St Paul, MN in 2008.
**Table S11**. Infection types (ITs) observed when CAT‐A1 and 10 other genotypes were tested with nine races of *Puccinia graminis* f. sp. *tritici* at 25°C and 20°C at Fargo, ND.
**Table S12**. Infection types observed when CAT‐A1 and 10 other genotypes were tested with races JRCQC and TTKSK of *Puccinia graminis* f. sp. *tritici* at 25°C and 20°C at St Paul, MN.
**Table S13**. Analysis of durum and common wheat cultivars or lines with six STARP markers linked to the *Sr13* locus.
**Table S14**. Pedigrees and *Sr13* haplotypes of North Dakota durum cultivars.
**Table S15**. Avirulence/virulence formula for *Puccinia graminis* f. sp. *tritici* races used or mentioned in this study.

## Data Availability

The sequence of the *Sr13* haplotype R4 has been deposited in the GenBank database (accession no. MW033594; https://www.ncbi.nlm.nih.gov/nuccore/MW033594). All other relevant data can be found within the manuscript and its supporting materials.
