# Supplementary figures and images for: Function and evolution of allelic variations of Sr13 conferring resistance to stem rust in tetraploid wheat (Triticum turgidum L.)
Source: Plant J. 2021 May 29;106(6):1674–91. doi: 10.1111/tpj.15263 (PMC8362117; doi:10.1111/tpj.15263)

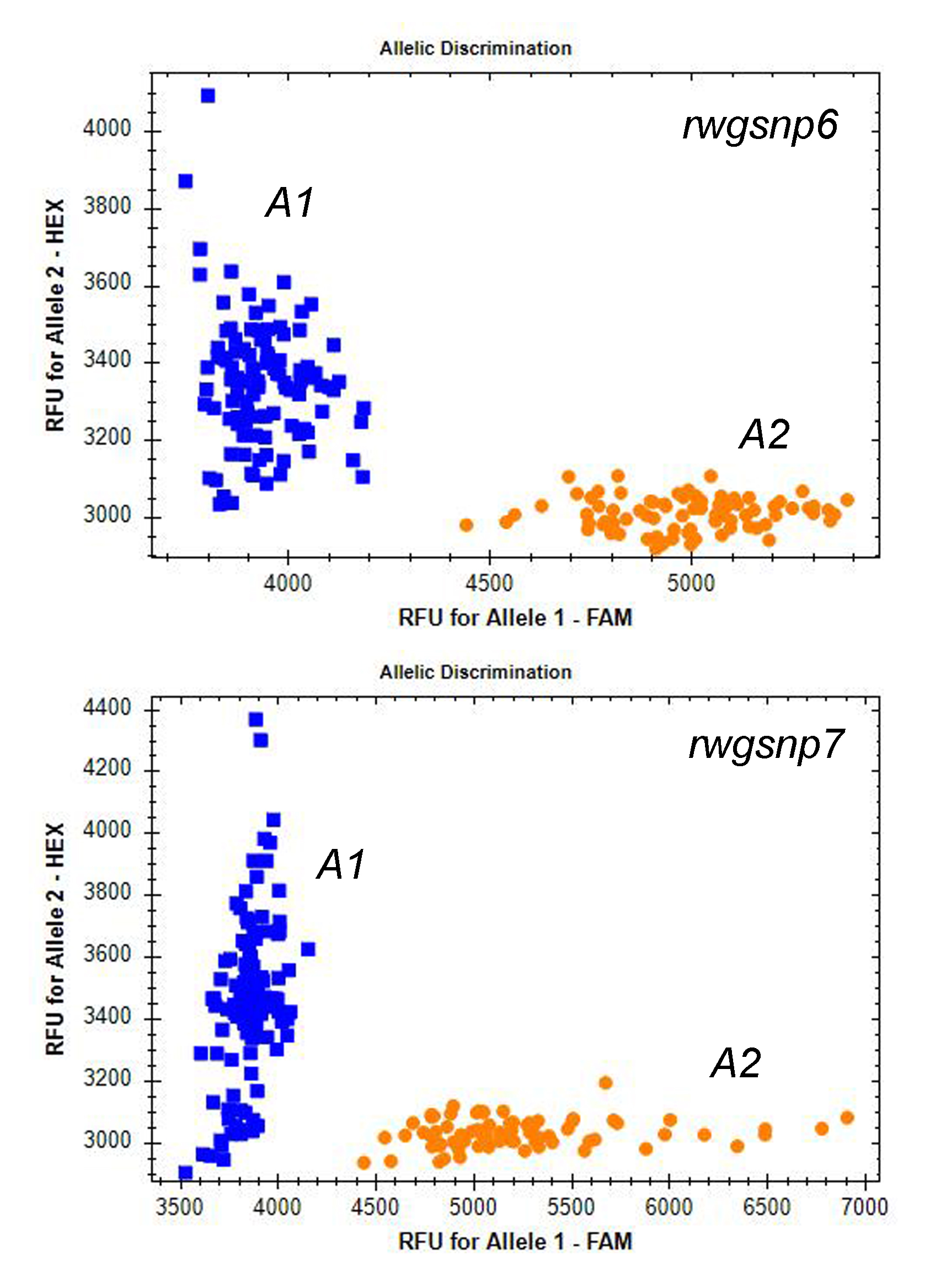

Supplement: Supplementary file 1 — Figure S1. Analysis of two STARP markers (rwgsnp6 and rwgsnp7) on a recombinant inbred line population and parents (Rusty, Triticum turgidum subsp. carthlicum PI 387696) using the CFX384TM Real‐Time System. [file TPJ-106-1674-s005.jpg]

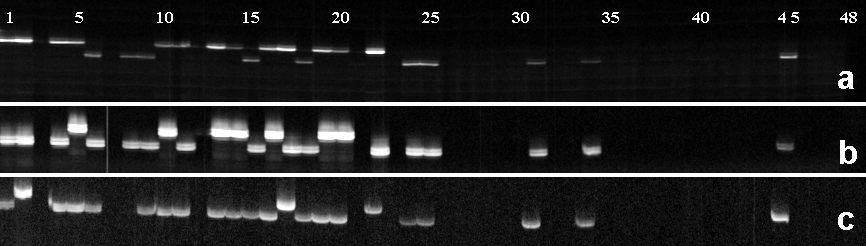

Supplement: Supplementary file 2 — Figure S2. Polyacrylamide gel‐electrophoresis of STARP markers rwgsnp37.2 (a), rwgsnp38 (b), and rwgsnp39 (c) testing 16 tetraploid and 32 hexaploid wheat cultivars or genotypes. [file TPJ-106-1674-s007.tif]

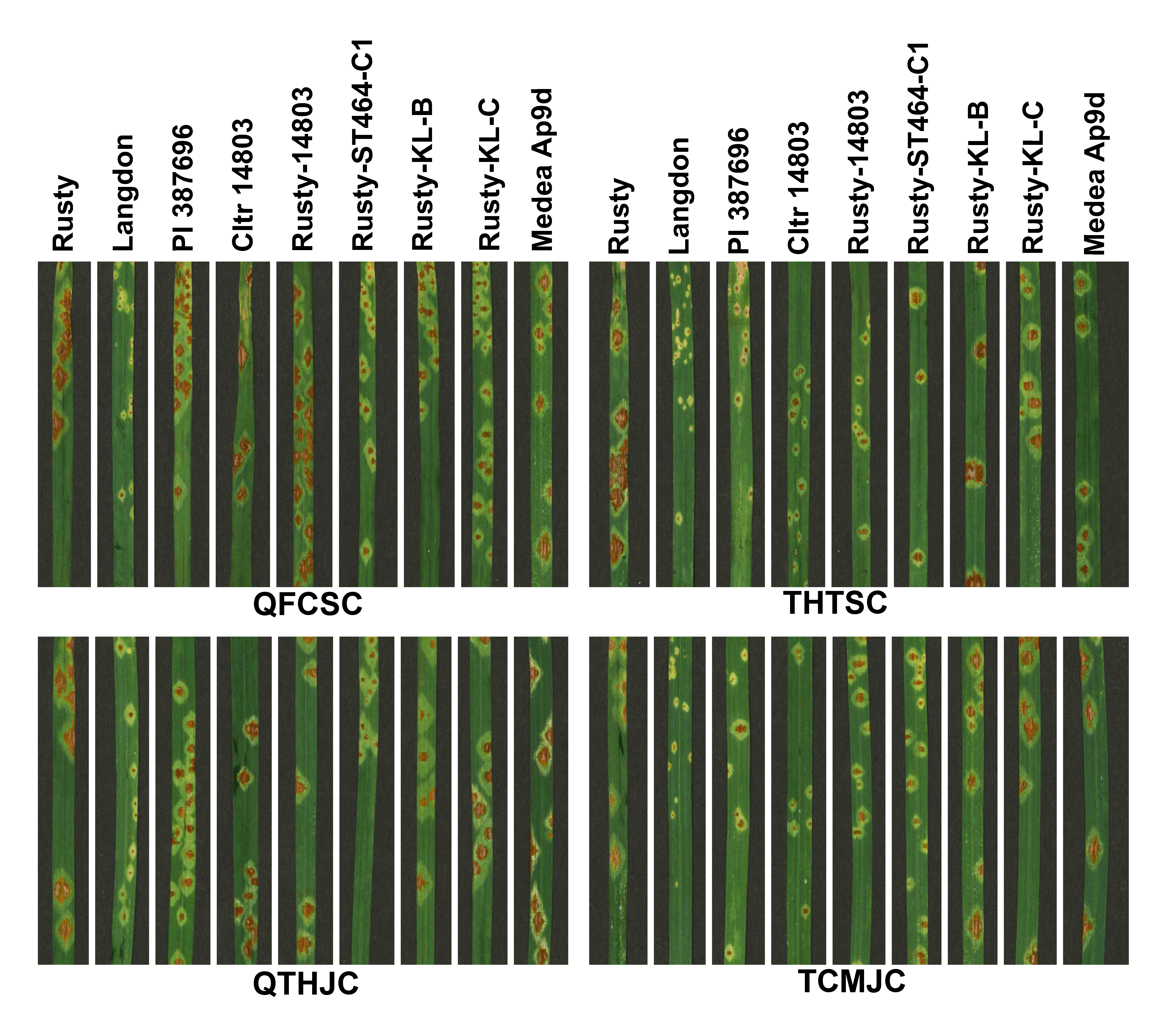

Supplement: Supplementary file 3 — Figure S3. Infection types observed on nine tetraploid genotypes inoculated with Puccinia graminis f. sp. tritici races QFCSC, QTHJC, THTSC and TCMJC and incubated at 25°C. [file TPJ-106-1674-s012.jpg]

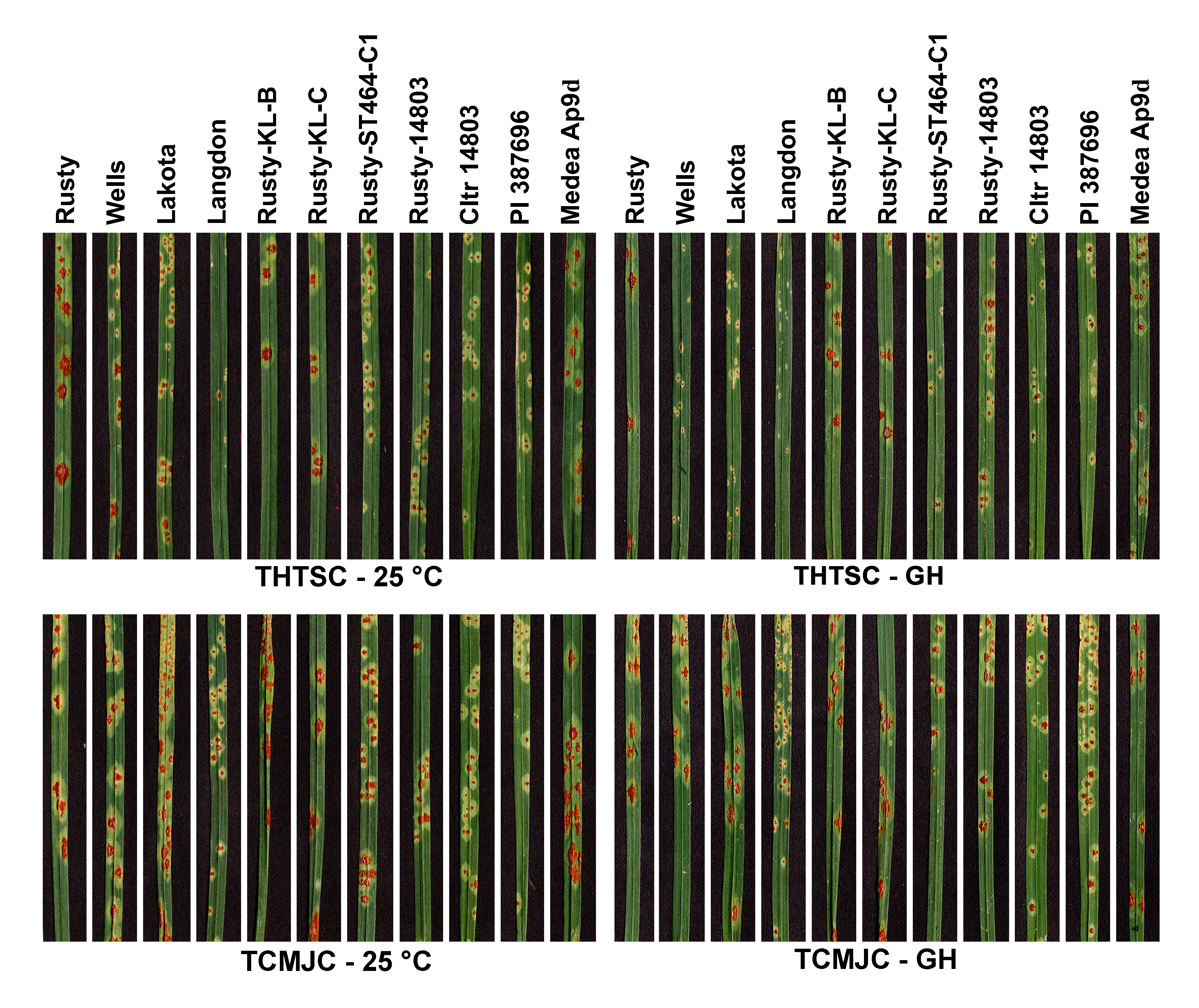

Supplement: Supplementary file 4 — Figure S4. Infection types observed on 11 tetraploid genotypes inoculated with Puccinia graminis f. sp. tritici races THTSC and TCMJC and incubated at either 25°C or in a greenhouse maintained at 21°C. [file TPJ-106-1674-s010.jpg]

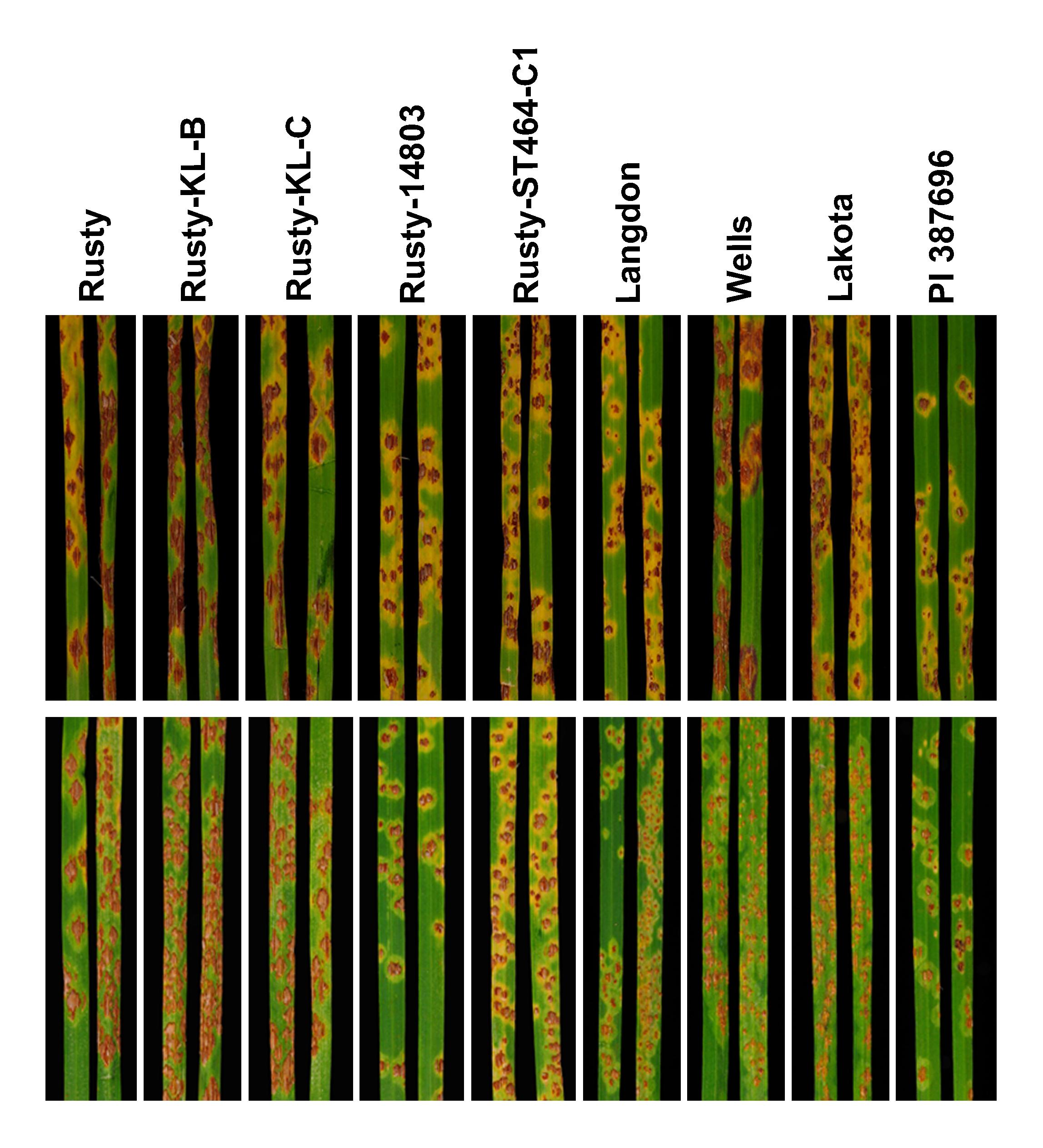

Supplement: Supplementary file 5 — Figure S5. Infection types observed on nine durum genotypes inoculated with stem rust (Puccinia graminis Pers. f. sp. tritici) race TCMJC and incubated at 25°C in two replications with two leaves per replication shown per genotype. [file TPJ-106-1674-s003.jpg]

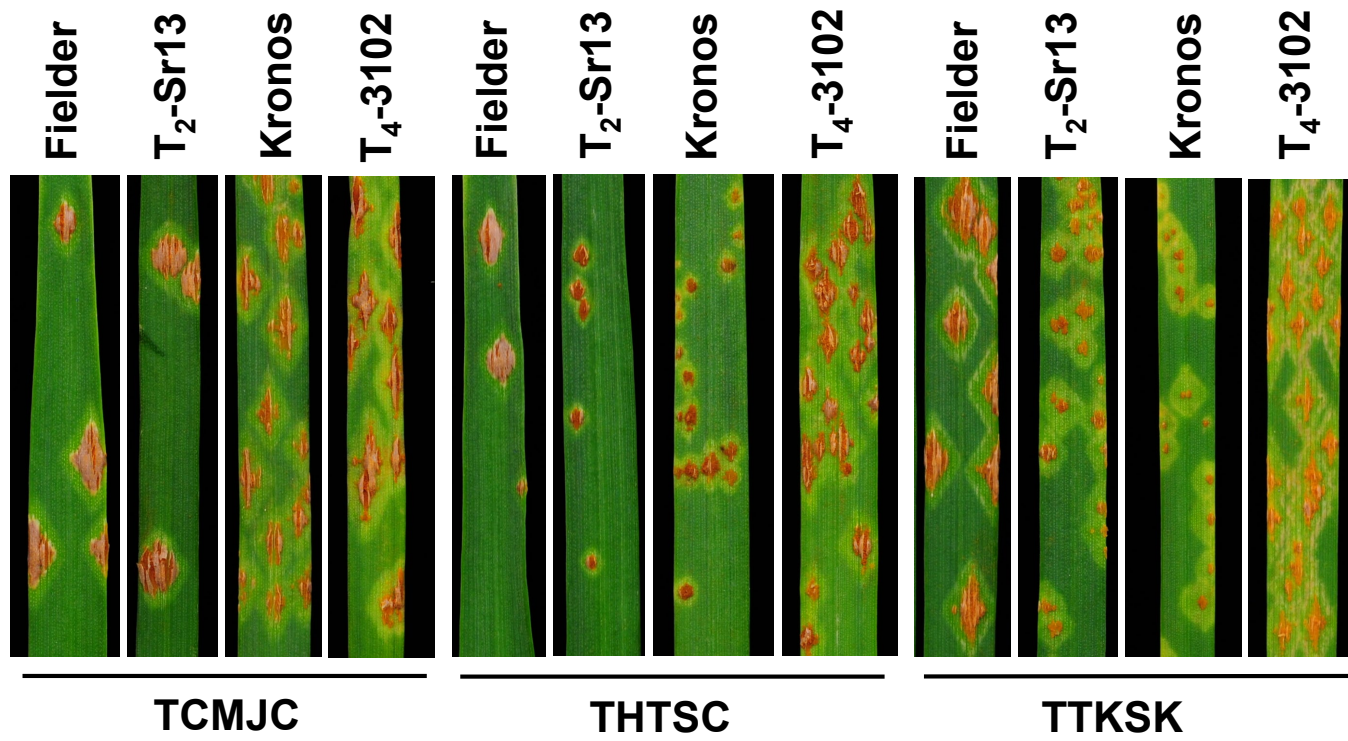

Supplement: Supplementary file 6 — Figure S6. Infection types observed on four genotypes inoculated with races THTSC, TCMJC and TTKSK of Puccinia graminis Pers. f. sp. tritici. [file TPJ-106-1674-s008.pdf]

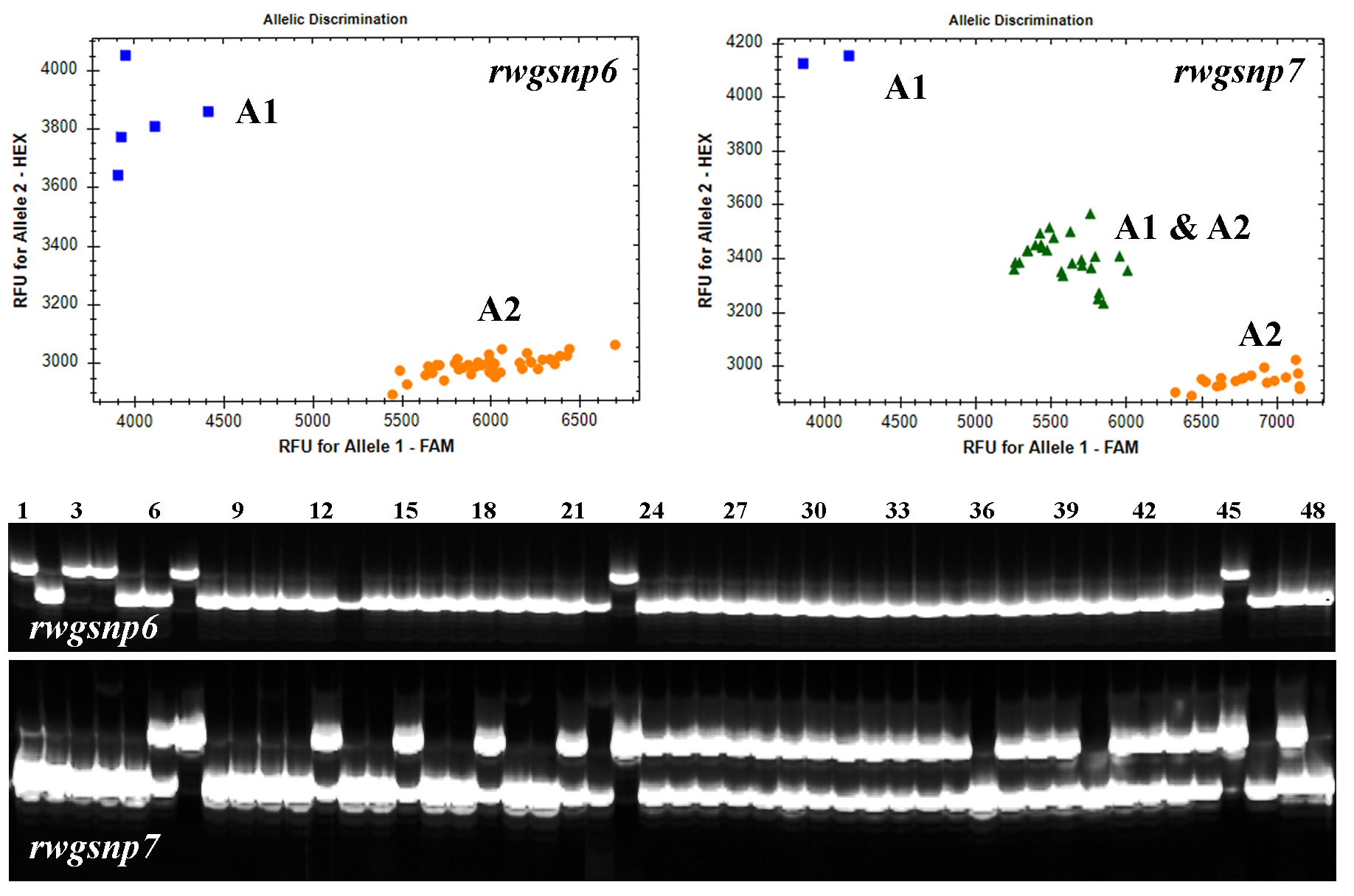

Supplement: Supplementary file 7 — Figure S7. Validation of two STARP markers (rwgsnp6 and rwgsnp7) on 48 common and tetraploid wheat cultivars and lines. [file TPJ-106-1674-s004.jpg]
