# Supplementary material for: Function and evolution of allelic variations of Sr13 conferring resistance to stem rust in tetraploid wheat (Triticum turgidum L.)
Source: Plant J. 2021 May 29;106(6):1674–91. doi: 10.1111/tpj.15263 (PMC8362117; doi:10.1111/tpj.15263)
Supplement: Supplementary file 8 — Table S1. Stem rust resistant lines developed at Fargo, ND, USA by N. D. Williams and maintained by the USDA‐ARS and their status when tested for presence of Sr13 using marker KASPSr13. Table S2. Segregation of resistance to three races of Puccinia graminis f. sp. tritici in the F2 and recombinant inbred line populations derived from the cross Rusty × T. turgidum subsp. carthlicum PI 387696. [file TPJ-106-1674-s009.docx]

| **Table S1**. Stem rust resistant lines developed at Fargo, North Dakota, USA by N.D. Williams and maintained by USDA-ARS and their status when tested for presence of *Sr13* using marker *KASPSr13*. | | | | | | | | | | | |
| --- | --- | --- | --- | --- | --- | --- | --- | --- | --- | --- | --- |
| Donor Parent | | | CI or PI No. of donor parent | Designation | 4X or 6X | Citations |  | Total lines | No. Sr13 pos | Name of Sr13+ lines | Comments |
|  |  |  |  |  |  |  |  |  |  |  |  |
| Monogenic tetraploid lines derived by crosses to Marruecos 9623 | | | | | | | | | | | |
| 1 | | Acme | CI 5284 | Srda | 4X | 1, 4, 8 |  | 21 | 0 |  | a Stakman differential |
| **2** | | **Beladi 116** | **PI 133457** | **BI 116** | **4X** | **1** |  | **8** | **0** |  |  |
| 3 | | Camadi Abdu tipo #103 | PI 192168 | CAT | 4X | 3 |  | 2 | 1 | CAT-A1 |  |
| 4 | | CI 8155 | CI 8155 | 8155 | 4X | 3 |  | 5 | 2 | 8155-B2, 8155-C1 | an East African durum wheat |
| 5 | | Iumillo |  | Im | 4X | 3 |  | 7 | 2 | Im-B7, Im-C2 | source of Thatcher resistance |
| 6 | | Khapli |  | KL | 4X | 6, 8 |  | 4 | 2 | KL-B, KL-C | a Stakman differential, important source of *Sr13*, *Sr14* |
| 7 | | Mindum |  | Srdm | 4X | 4, 8 |  | 3 | 0 |  | a Stakman differential |
| 8 | | Palestine | PI 94701 | Plt | 4X | 2, 8 |  | 3 | 0 |  | from ancient Palestine |
| 9 | | Spelmar | CI 9236 | Sm | 4X | 3 |  | 5 | 0 |  | a Stakman differential |
| 10 | | ST464 | CI 13160 | ST464 | 4X | 3, 16, 17 |  | 4 | 1 | ST464-C1 | an East African durum wheat, source of *Sr13* |
| 11 | | Tremez Rijo | CI 7066 | Tr | 4X | 1 |  | 6 | 0 |  |  |
| 12 | | Vernal | CI 3686 | Vn | 4X | 3 |  | 5 | 0 |  | a Stakman differential, *Sr9e* |
|  | |  |  |  |  |  |  |  |  |  |  |
| Monogenic hexaploid lines derived by crosses to Little Club | | | | | | | | | | | |
| 13 | | Kota |  | Kt | 6X | 5 |  | 4 | 0 |  | a Stakman differential |
| 14 | | Marquis |  | Mq | 6X | 5, 9, 10 |  | 3 | 0 |  | a Stakman differential *Sr18, 19*, and *20* |
| 15 | | Reliance |  | RL | 6X | 2, 9, 10 |  | 5 | 0 |  | a Stakman differential *Sr5, 16, 18, 20* |
| 16 | | Kota-B |  | LC/LC^8^-Kt-B | 6X | 5 |  | 2 | 0 |  | a derivative of Kota in a near-isogenic Little Club (LC) background |
| 17 | | Waldron |  | WDR | 6X | 7, 11, 12 |  | 6 | 0 |  | *Sr11, 41, SrWld1* |
|  | |  |  |  |  |  |  |  |  |  |  |
| Miscellaneous stem rust resistant lines, not necessarily monogenic and not in a Little Club or Marruecos 9623 background | | | | | | | | | | | |
| 18 | | Canthatch |  | Canthatch | 6X | 13 |  | 1 | 0 |  | parent to Dhanapala's M1 and Ctch Suppressor mutants |
| 19 | | Dhanapala's M1 | local line | Dhanapala's M1 | 6X | 14 |  | 1 | 0 |  | mutation on 5D, allelic to *Sr30*, could possibly be *Sr30* |
| 20 | | Canthatch Suppressor mutants | local lines | Ctch | 6X | 13, 18 |  | 15 | 0 |  | suppressor mutation on 7D, *SuSr-D1* |
| 21 | | McNair-Triumph64 |  | Mc-T64 | 6X | 15 |  | 2 | 0 |  |  |
| 22 | | T. durum 56-1 |  | T. durum 56-1 | 4X |  |  | 1 | 0 |  | probably not monogenic |
|  | |  |  |  |  |  |  |  |  |  |  |
| Citations | | |  |  |  |  |  |  |  |  |  |
|  | | 1. **Weeraratne H., and Williams, N.D**. (1971) Inheritance of resistance to stem rust in six durum wheats. | | | | | | | | |  |
|  | | Crop Sci. **11**:915-918. doi.org/10.2135/cropsci1971.0011183X001100060046x | | | | | | | | |  |
|  | |  |  |  |  |  |  |  |  |  |  |
|  | | 2. **Rondon, M.R., Gough, F.J., and Williams, N.D**. (1966) Inheritance of stem rust resistance in | | | | | | | |  |  |
|  | | *Triticum aestivum* ssp. *vulgare* 'Reliance' and P.I.94701 of *Triticum durum*. Crop Sci. **6**:177-179. | | | | | | | |  |  |
|  | | doi.org/10.2135/cropsci1966.0011183X000600020020x | | | | |  |  |  |  |  |
|  | |  |  |  |  |  |  |  |  |  |  |
|  | | 3. **Williams N.D. and Gough, F.J**. (1968) Inheritance of a stem rust resistance of tetraploid wheats. | | | | | | | |  |  |
|  | | 3rd Int. Wheat Genet. Symp. Pp. 239-244. | | | |  |  |  |  |  |  |
|  | |  |  |  |  |  |  |  |  |  |  |
|  | | 4. **Gough, F.J. and Williams, N.D.** (1963) Inheritance of stem rust reaction in two durum varieties, Acme and Mindum. | | | | | | | | |  |
|  | | Phytopath. **53**:295-299. |  |  |  |  |  |  |  |  |  |
|  | |  |  |  |  |  |  |  |  |  |  |
|  | | 5. **Berg, L.A., Gough, F.J., and Williams, N.D.** (1963) Inheritance of stem rust resistance in two wheat varieties, Marquis and Kota. | | | | | | | | | |
|  | | Phytopath. **53**:904-908. |  |  |  |  |  |  |  |  |  |
|  | |  |  |  |  |  |  |  |  |  |  |
|  | | 6. **Williams, N.D., and Gough, F.J.** (1965) Inheritance of stem rust reaction in a Khapli emmer cross. | | | | | | | |  |  |
|  | | Crop Sci. **5**:145-147. doi.org/10.2135/cropsci1965.0011183X000500020013x | | | | | | | |  |  |
|  | |  |  |  |  |  |  |  |  |  |  |
|  | | 7. **Williams, N.D., and Miller, J.D.** (1982) Inheritance of resistance to stem rust in a selection of the wheat cultivar 'Waldron'. | | | | | | | | | |
|  | | Crop Sci. **22**:1175-1179. doi.org/10.2135/cropsci1982.0011183X002200060021x | | | | | | | | |  |
|  | |  |  |  |  |  |  |  |  |  |  |
|  | | 8. **Williams, N.D., and Miller, J.D.** (1982) Allelic and linkage relations among genes for stem rust resistance from *Triticum turgidum*, | | | | | | | | | |
|  | | 'Mindum', 'Acme' selection, 'Palestine', and 'Khapli'. Crop Sci. **22**:1203-1207. doi.org/10.2135/cropsci1982.0011183X002200060027x | | | | | | | | | |
|  | |  | | | | | | | | | |
|  | \| 9. **Williams, N.D., and Kaveh, H.** (1976) Relationships of genes for reaction to stem rust from 'Marquis' and 'Reliance' wheat to other Sr genes.  Crop Sci. **16**:561-564. doi.org/10.2135/cropsci1976.0011183X001600040033x \| \| --- \| \|  \| \| 10. **Williams, N.D., Gough, F.J. and Rondon, M.R.** (1966) Interaction of pathogenicity in *Puccinia graminis* f. sp. *tritici* and reaction genes in \| \| *Triticum aestivum* ssp. *vulgare* 'Marquis' and 'Reliance'. Crop Sci. **6**:245-248. doi.org/10.2135/cropsci1966.0011183X000600030008x \| | | | | | | | | | | |
|  |  | | | | | | | | | | |
|  | \| 11. **Riede, C.R., Williams, N.D. and Miller, J.D.** (1995) Wheat lines monogenic for resistance to stem rust from the wheat cultivar 'Waldron'. \| \| \| \| \| \| --- \| --- \| --- \| --- \| --- \| \| Theor. Appl. Genet **90**:1164-1168. doi.org/10.1007/BF00222938 \| \| \| \| \|  \|  \|  \|  \| \| 12. **Riede, C.R., Williams, N.D., Miller, J.D., and Joppa, L.R.** (1995) Chromosomal location of genes for stem rust resistance derived from 'Waldron' wheat. \| \| \| \| \| \| Theor. Appl. Genet **90**:1158-1163. doi.org/10.1007/BF00222937 \| \| \| \| | | | | | | | | | | |
|  |  | | | | | | | | | | |
|  | \| 13. **Williams, N.D., Miller, J.D., and Klindworth, D.L.** (1992) Induced mutations of a genetic suppressor of resistance to wheat stem rust. \| \| \| \| \| \| \| \| \| \| \| --- \| --- \| --- \| --- \| --- \| --- \| --- \| --- \| --- \| --- \| \| Crop Sci. **32:**612-616. doi.org/10.2135/cropsci1992.0011183X003200030008x \| \| \| \| \| \| \| \|  \|  \| \|  \|  \|  \|  \|  \|  \|  \|  \|  \|  \| \| 14. **Dhanapala, M.P., Williams, N.D., Miller, J.D., and Klindworth, D.L.** (1991) Chromosomal location of a gene for resistance to wheat stem rust. \| \| \| \| \| \| \| \| \| \| \| 1991 Agronomy Abstracts, American Society of Agronomy, Madison, WI. p. 91. \| \| \| \| \| \| \| \|  \|  \| \|  \|  \|  \|  \|  \|  \|  \|  \|  \|  \| \| 15. **Williams, N.D., Miller, J.D., Klindworth, D.L., and Joppa, L.R.** (1998) Genes for wheat stem rust resistance from Triumph 64. \| \| \| \| \| \| \| \| \| \| \| *In*: Slinkard A.E. (ed) Proc 9th Int Wheat Genet Symp. University of Saskatchewan Extension Press, Saskatoon, pp 342–344 \| \| \| \| \| \| \| \| \| \| | | | | | | | | | | |
|  | |  | | | | | | | | | |
|  | \| 16. **Klindworth, D.L., Miller, J.D., Jin, Y., and Xu, S.S.** (2007) Chromosomal locations of genes for stem rust resistance in monogenic lines derived from \| \| \| \| \| \| \| \| \| \| \| --- \| --- \| --- \| --- \| --- \| --- \| --- \| --- \| --- \| --- \| \| tetraploid wheat accession ST464. Crop Sci.  **47**:1441-1450. doi:10.2135/cropsci2006.05.0345 \| \| \| \| \| \| \| \| \|  \| \|  \|  \|  \|  \|  \|  \|  \|  \|  \|  \| \| 17. **Zhang, W., Chen, S., Abate, Z., Nirmala, J., Rouse, M.N., and Dubcovsky, J.D.** (2017) Identification and characterization of *Sr13*, \| \| \| \| \| \| \| \| \| \| \| a tetraploid wheat gene that confers resistance to the Ug99 stem rust race group. Proc. Natl. Acad. Sci. **114**:E9483–E9492. doi:10.1073/pnas.1706277114 \| \| \| \| \| \| \| \| \| \| \|  \|  \|  \|  \|  \|  \|  \|  \|  \|  \| \| 18. **Hiebert, C.W, Moscou, M.J., Hewitt, T., Steuernagel, B**, et al. (2020) Stem rust resistance in wheat is suppressed by a subunit of the mediator complex. \| \| \| \| \| \| \| \| \| \| \| Nature Communications **11**:1123. doi:10.1038/s41467-020-14937-2 \| \| \| \| \|  \|  \|  \|  \|  \| | | | | | | | | | | |
|  | |  | | | | | | | | | |
|  | |  | | | | | | | | | |

| **Table S2.**  Segregation of resistance to three races of *Puccinia graminis* f. sp. *tritici* in the F_2_ and recombinant inbred line (RIL) populations derived from the cross Rusty × *T. turgidum* subsp. *carthlicum* PI 387696. | | | | | | | | | |
| --- | --- | --- | --- | --- | --- | --- | --- | --- | --- |
| Race | F_2_ plants | | | |  | RILs | | | |
|  | R | S | χ^2^ (3:1) | *p* value |  | R | S | χ^2^ (1:1) | *p* value |
| TTKSK | 63 | 20 | 0.031 | 0.860 |  | 85 | 98 | 0.922 | 0.336 |
| TRTTF | 68 | 22 | 0.015 | 0.903 |  | 85 | 98 | 0.922 | 0.336 |
| TMLKC | 128 | 39 | 0.241 | 0.623 |  | 85 | 98 | 0.922 | 0.336 |
|  |  |  |  |  |  |  |  |  |  |
|  |  |  |  |  |  |  |  |  |  |
