# Supplementary material for: Function and evolution of allelic variations of Sr13 conferring resistance to stem rust in tetraploid wheat (Triticum turgidum L.)
Source: Plant J. 2021 May 29;106(6):1674–91. doi: 10.1111/tpj.15263 (PMC8362117; doi:10.1111/tpj.15263)
Supplement: Supplementary file 9 — Table S3. Molecular markers mapped in the population of 190 recombinant inbred lines derived from cross durum (Rusty) × T. turgidum subsp. carthlicum (PI 387696). [file TPJ-106-1674-s002.docx]

| **Table S3.** Molecular markers mapped in the population of 190 recombinant inbred lines derived from cross durum (Rusty) × *T. turgidum* subsp. *carthlicum* (PI 387696). | | | | | |  |
| --- | --- | --- | --- | --- | --- | --- |
| **No.** | **Chromo-some** | **Marker shown in maps** | **Position (cM)** | **Segregation distortion** | **Co-located markers†** |  |
| 1 | 1A | *IWB7436* | 0.0 | ns |  |  |
| 2 | 1A | *IWB12616* | 2.0 | ns | *65373, 6058, 7208, 7944, 9222, 2454, 9223, 8776, 34801,12147, 25590, 16675, 6234, 6476, 74213, 71565, 57868, 47522, 29495.1, 7943, 74011.1, 13141, 8777, 34707* |  |
| 3 | 1A | *IWB71424.1* | 2.3 | ns | *29206* |  |
| 4 | 1A | *IWB57448* | 3.1 | ns | *8696* |  |
| 5 | 1A | *IWB6172* | 4.0 | ns | *29039, 29562, 35349, 33789, 44057, 3087, IWA8622, 17894, 44655, 8796, 28577, 11117, 31866, 31867.1, IWA4351, 11098* |  |
| 6 | 1A | *IWB7590* | 4.5 | ns |  |  |
| 7 | 1A | *IWB71687.1* | 4.8 | ns | *59361.1, 11162,71688.1* |  |
| 8 | 1A | *IWB61182* | 5.0 | ns | *12612, 7008, 10730.1, 46642* |  |
| 9 | 1A | *IWB10922* | 6.4 | ns | *10921, IWA4644, 11203, 12166, 13009, 9667, 11301, IWA4678, 12206, 11053, 71172.1, 73271, 71173, 7089, 36389, 73615, 59865.1* |  |
| 10 | 1A | *IWB63610* | 8.2 | ns |  |  |
| 11 | 1A | *IWB63712* | 10.3 | ns |  |  |
| 12 | 1A | *IWB36053* | 11.4 | ns | *72128, 5896, 10192.1, 72794, 7627, 12126, 7351, 72127, 10932, 72258, 35266.1* |  |
| 13 | 1A | *IWA414* | 11.4 | ns | *19101, 69805, 68393* |  |
| 14 | 1A | *IWB7727* | 12.2 | ns |  |  |
| 15 | 1A | *IWB7233* | 13.1 | ns | *31746, 67281, 67282, 7640, 35007.1* |  |
| 16 | 1A | *IWB3519* | 13.1 | ns | *50401, 57361, 31764.1, 59245, 9029, 31763.1, 9329, 51995.1, 57360.1, 12172.1, 9541, 31415, 8188, 31414.1, 59888.1* |  |
| 17 | 1A | *IWB11044* | 16.0 | ns |  |  |
| 18 | 1A | *IWB3334.1* | 18.6 | ns | *28549, IWA6217, 3682, 31933.1* |  |
| 19 | 1A | *IWB31932* | 23.3 | ns |  |  |
| 20 | 1A | *IWB31602* | 23.6 | ns |  |  |
| 21 | 1A | *IWB47933* | 25.0 | ns | *68107, 68106, 73417* |  |
| 22 | 1A | *IWA7048* | 25.6 | ns |  |  |
| 23 | 1A | *IWB72800* | 25.8 | ns | *66889, 26996, 72799, 66891* |  |
| 24 | 1A | *IWB7717* | 26.9 | ns |  |  |
| 25 | 1A | *IWB49926* | 27.5 | ns |  |  |
| 26 | 1A | *IWB47241* | 30.1 | ns | *72042, 6583, 73155, 10025, 12486, 7300, 59951, 72041, 7075.1, 43982* |  |
| 27 | 1A | *IWB52279* | 33.9 | ns | *57837, 57838* |  |
| 28 | 1A | *IWB35115* | 35.6 | ns |  |  |
| 29 | 1A | *IWB21167* | 35.8 | ns |  |  |
| 30 | 1A | *IWB57257.1* | 37.2 | ns | *IWA8307, 34853* |  |
| 31 | 1A | *Xgwm164* | 40.4 | ns |  |  |
| 32 | 1A | *Xbarc120* | 42.5 | ns |  |  |
| 33 | 1A | *IWA8506.1* | 43.4 | ns |  |  |
| 34 | 1A | *IWB6962* | 43.9 | ns | *14948, 12189.1, 28360, 45313, 6784, 8519, 31787, 6744, 65085, 50585, 65677, 25221, 44535, 44534, 6380,31380, 27755, 72311, 36350, 65824, 57485 43938, 6966, 59609* |  |
| 35 | 1A | *IWB71416* | 44.2 | ns | *71415, 65334, 8098, 6933* |  |
| 36 | 1A | *IWA3883* | 44.4 | ns | *11427, 6128, 36287, 31862, 7828, IWA4302, IWA7922, 57739, 65010, 57895, IWA3884, 36224, IWA6887, IWA7879, 57676, IWA8520, 9258, 44145, 73513, 39261, IWA4301, IWA7505, 34735, 59957, 35639, 3618, 46887, IWA3499, IWA3882, IWA8394, 9345, 25829, 25830, 58586* |  |
| 37 | 1A | *IWB8898* | 44.7 | ns | *11660* |  |
| 38 | 1A | *IWB13492* | 45.0 | ns | *64861, IWA3536, 3507, 4472, 35080, 31366, IWA6985, 56828, 35436, 45374, 59094, IWA4852, IWA3538, 60215, 65705, 59768, 65314, 20955, 31652, 31690, 32307, IWA8198* |  |
| 39 | 1A | *IWB15064* | 45.2 | ns | *27064, 15302, 57405, 14005, 57483, 45811, IWA3665, IWA8026, 27889, 12984, IWA3806, 14772, 57546, IWA498, 57547.1, 60796* |  |
| 40 | 1A | *IWB71889* | 45.5 | ns |  |  |
| 41 | 1A | *IWB8273* | 46.3 | ns | *66135, IWA4576, IWA697,111893, 10292, 12595, IWA5268, IWA3528, 10807.1, 12529, 65452, 17431, 9465, 16658, IWA3695, IWA7945, IWA4265, IWA6972, 35410, 72825, 10918, 46963, 35945, 3707, 25621, IWA7104, 56573, 26574, 35883, 31760, IWA42* |  |
| 42 | 1A | *IWB61236* | 46.6 | ns |  |  |
| 43 | 1A | *IWB66378* | 46.6 | ns | *IWA490, IWA3957, 72418, 6779, 11190, 31640, 57290, IWA3955, IWA3956, 35364, 47404* |  |
| 44 | 1A | *IWB64464* | 48.6 | ns |  |  |
| 45 | 1A | *IWB20695* | 50.0 | ns | *60037, 72053, 2927, 35117, 52241, 20671, 35842* |  |
| 46 | 1A | *IWB35308* | 52.4 | ns | *IWA4326, IWA4328* |  |
| 47 | 1A | *IWA8070* | 54.7 | ns | *34341* |  |
| 48 | 1A | *IWB58903* | 55.0 | ns |  |  |
| 49 | 1A | *IWB11515* | 55.8 | ns | *11516* |  |
| 50 | 1A | *IWB5406* | 56.4 | ns | *34656, 35023, 40078, IWA7018, 7921, 46302, IWA3820, 70213* |  |
| 51 | 1A | *IWB25487* | 57.5 | ns | *6733* |  |
| 52 | 1A | *IWB35691* | 57.7 | ns |  |  |
| 53 | 1A | *IWB31863* | 58.0 | ns |  |  |
| 54 | 1A | *IWB34337* | 59.1 | ns | *35039, 35897* |  |
| 55 | 1A | *IWB11628* | 59.4 | ns | *70352, 6769, IWA4283* |  |
| 56 | 1A | *IWB43838* | 59.6 | ns |  |  |
| 57 | 1A | *IWB65763* | 60.2 | ns | *12119* |  |
| 58 | 1A | *IWA7871* | 61.0 | ns | *7079, 11063* |  |
| 59 | 1A | *IWB46385* | 61.3 | ns | *IWA3496, 25493, 9588* |  |
| 60 | 1A | *IWB75761* | 61.6 | ns |  |  |
| 61 | 1A | *Xwmc312* | 62.4 | ns |  |  |
| 62 | 1A | *IWB11543* | 62.9 | ns | *IWA3406, 65398, 46448* |  |
| 63 | 1A | *Xcfa2129* | 63.8 | ns |  |  |
| 64 | 1A | *IWB60627* | 64.0 | ns |  |  |
| 65 | 1A | *IWB58118* | 66.0 | ns | *28402, 27332, 58119, 34331* |  |
| 66 | 1A | *IWB27132* | 66.5 | ns |  |  |
| 67 | 1A | *IWB44683.1* | 67.4 | ns |  |  |
| 68 | 1A | *IWB8167* | 68.8 | ns | *7965, 8166, 9545, 13379, 6354, 47390, 60784, IWA3405, 9631, IWA5493, IWA7144, 64795, IWA5534, 31849, 29087, IWA3859* |  |
| 69 | 1A | *IWB35531* | 72.9 | ns | *47990* |  |
| 70 | 1A | *IWB6517* | 74.3 | ns | *45538* |  |
| 71 | 1A | *IWB12579* | 76.3 | * | *20654* |  |
| 72 | 1A | *IWB29580* | 77.1 | * | *29579* |  |
| 73 | 1A | *IWB4075* | 77.4 | * | *68917* |  |
| 74 | 1A | *IWB35025* | 77.7 | * | *4042, IWA145, IWA3805, IWA8482, 65422, IWA6042, IWA3434, 36087 IWA3435* |  |
| 75 | 1A | *IWB11406* | 78.8 | ns | *72493, 36711.1, 50094* |  |
| 76 | 1A | *IWB7472* | 80.4 | ns |  |  |
| 77 | 1A | *IWB64554* | 87.5 | * | *64553* |  |
| 78 | 1A | *IWB9746* | 88.6 | * |  |  |
| 79 | 1A | *IWB52732* | 90.6 | ns |  |  |
| 80 | 1A | *IWB52033* | 90.9 | ns |  |  |
| 81 | 1A | *IWB31208.1* | 93.5 | ns |  |  |
| 82 | 1A | *IWB66930* | 95.5 | ns | *66931* |  |
| 83 | 1A | *IWB31445* | 96.9 | ns | *60380* |  |
| 84 | 1A | *IWB3536* | 102.3 | ns |  |  |
| 85 | 1A | *IWB3034* | 112.0 | ** |  |  |
| 86 | 1A | *IWB67875* | 113.2 | * |  |  |
| 87 | 1A | *IWB62751* | 114.0 | * | *IWA6253, 11057, 74107, 28698, 65681, IWA4713, 26250, 31696, 59427* |  |
| 88 | 1A | *IWB13639* | 114.5 | * | *58474, 13551, 73690, IWA6191* |  |
| 89 | 1A | *IWB4244* | 115.1 | * | *IWA3409, 73557, 13385, 35045, 47703, 2902* |  |
| 90 | 1A | *IWB61091* | 115.9 | ns | *64453, 33223, IWA7893, 29244* |  |
| 91 | 1A | *IWB12293* | 120.2 | ns | *7275, 7430* |  |
| 92 | 1A | *IWA4898* | 120.8 | ns | *58979, IWA4897* |  |
| 93 | 1A | *IWB6426* | 121.0 | ns | *60174* |  |
| 94 | 1A | *IWB43929* | 121.6 | ns | *12340.1, 70021, 58886* |  |
| 95 | 1A | *IWB30871* | 124.0 | ns | *67027, 45183, 59763.1, 31959, 31956, 31955, 31958.1, 26766.1, 26765, 45182, 58726* |  |
| 96 | 1A | *IWB3413* | 124.3 | ns | *7489, 7490, 36094, 8643, 37412, 27546, 34683, 34872, 7532, 35476, IWA8523, 65748, 9191.1, IWA7289* |  |
| 97 | 1A | *IWB65294* | 124.5 | ns | *73764, 71744, 63140, 16568, 60807, 12954.1, 44295, 45352, 60808, 28413.1, 7260, 52266, 39912, IWA5734, 11979* |  |
| 98 | 1A | *Xbarc17* | 125.8 | ns |  |  |
| 99 | 1B | *IWB15041* | 0.0 | ns | *10856* |  |
| 100 | 1B | *IWB10856* | 0.0 | ns |  |  |
| 101 | 1B | *IWB65421* | 1.1 | ns |  |  |
| 102 | 1B | *IWB44730* | 1.4 | ns | *25090, 13538, 27585, 11290* |  |
| 103 | 1B | *IWB43880* | 1.7 | ns |  |  |
| 104 | 1B | *IWB44816* | 2.8 | ns | *52019, 47985, 47978, 7419.1, 44448.1, 40116, 66045, 12256, 73733, 12354, 59696* |  |
| 105 | 1B | *IWB52018* | 4.0 | ns |  |  |
| 106 | 1B | *IWB11925.1* | 6.9 | ns |  |  |
| 107 | 1B | *IWB27496* | 8.3 | ns |  |  |
| 108 | 1B | *IWB57219* | 8.6 | ns | *66722, 39279, 39278* |  |
| 109 | 1B | *IWB12208* | 10.7 | ns |  |  |
| 110 | 1B | *IWB12603* | 11.3 | ns | *71754, 10492, 29450.1, 26731, 68307, 31615, 6008, 12284, 52473, 73112, 10312, 65827, 35936, 59785, 17979, 72908, 12602* |  |
| 111 | 1B | *IWB29507* | 11.9 | ns |  |  |
| 112 | 1B | *IWB70626* | 13.6 | ns | *8902, 6024, 9956, 9378* |  |
| 113 | 1B | *IWB14010* | 15.6 | * | *12346, 47908.1, 72085, 65667, 73358, 72083, 12347, 64131, 9270* |  |
| 114 | 1B | *IWB31845* | 16.2 | * |  |  |
| 115 | 1B | *IWB65685* | 16.7 | * | *65159, 65156, 65155, 2622.1* |  |
| 116 | 1B | *IWB9714.1* | 18.4 | ns |  |  |
| 117 | 1B | *IWB14060* | 19.0 | ns |  |  |
| 118 | 1B | *IWB12048* | 24.3 | ns |  |  |
| 119 | 1B | *IWB72109* | 24.6 | ns |  |  |
| 120 | 1B | *IWB13124.1* | 32.6 | * | *59981* |  |
| 121 | 1B | *IWB58765* | 33.2 | * | *32639* |  |
| 122 | 1B | *IWB62647.1* | 33.7 | * | *57679, 25424, 34093, 28467* |  |
| 123 | 1B | *IWB8310.1* | 34.5 | ns | *IWA63, 71166, 67669, 67670, 71165, 9977, 73612, 18010, 9664, 69778, 17273* |  |
| 124 | 1B | *IWB64708* | 34.8 | * |  |  |
| 125 | 1B | *IWB72704* | 35.4 | ns | *35211, 33689, 7923, 7922* |  |
| 126 | 1B | *IWB9932* | 35.7 | ns | *12279* |  |
| 127 | 1B | *IWB9627* | 36.5 | ns | *31874, 47951, 10080, 31875, 72466, 6522, 34356* |  |
| 128 | 1B | *IWB29515* | 36.8 | ns | *46853* |  |
| 129 | 1B | *IWB8781* | 37.1 | ns |  |  |
| 130 | 1B | *IWB72487* | 37.1 | ns | *9109, 9236, 73942.1, 63847, 60993, 33204, 9942, 9421, 11695* |  |
| 131 | 1B | *IWB16228* | 37.6 | ns |  |  |
| 132 | 1B | *IWB69962* | 37.9 | ns | *7840, 35982, 9645* |  |
| 133 | 1B | *IWB68665* | 38.2 | ns | *68666* |  |
| 134 | 1B | *IWB10753* | 38.5 | ns | *73550, 69254, 8845, 69668, 10754, 7660, 45383, 63556, 72419, 60713, 60711, 67163, 35406, 12252, 73332, 35128, 35375* |  |
| 135 | 1B | *IWB63352* | 38.8 | ns | *60710, 28269* |  |
| 136 | 1B | *IWB60559* | 42.6 | ns |  |  |
| 137 | 1B | *IWB67741* | 43.5 | ns | *48048, 7314, 46756* |  |
| 138 | 1B | *IWB67742* | 44.0 | ns |  |  |
| 139 | 1B | *IWB10361* | 49.3 | ns |  |  |
| 140 | 1B | *IWB15647* | 49.9 | ns |  |  |
| 141 | 1B | *IWB14738* | 50.4 | ns | *58936, 10660, 8585, 47264, 8473, 8041, 58569, 28335, 8588, 10923, 9464, 63258, 9700, 7485, 36303, 11553, 11720, 7228* |  |
| 142 | 1B | *IWB66125* | 51.0 | ns |  |  |
| 143 | 1B | *IWB39745* | 51.9 | ns | *31722, 63613, 47026, 51784, 67218, 63614, 35740, 58640, 72968, 34998, 71062, IWA580, 65882, 47024, 31723, 47025, 70190, 71413, 70189, 49800, 35515, 68271, 72967, 67968, 74187, 68771, 20536, 26169, 39145, 4322, IWA7219, 72756, 65426, 8100, 63062, 27146, 21152, 17346, 73650, 70284, 5596, 71064, 72741, 65799, 64056, 59775, 72966, 72742, 67237, 47585, 60596, 2664, 74186, 73256, IWA5592, 72755, 71887, 69022, 60665, 63945, 8141, 31721, 9573, 60860, 60859, 45552, 27242, 69882, 47788* |  |
| 144 | 1B | *IWB9998* | 52.5 | ns |  |  |
| 145 | 1B | *IWB63944* | 53.8 | ns |  |  |
| 146 | 1B | *IWB7205* | 55.8 | ns | *45947* |  |
| 147 | 1B | *IWB35618* | 56.1 | ns |  |  |
| 148 | 1B | *IWA6965* | 56.4 | ns | *57824* |  |
| 149 | 1B | *IWB73157* | 58.1 | ns | *72174, 6409 ,72789, 7114, 73504, 9281* |  |
| 150 | 1B | *IWB34334* | 58.4 | ns |  |  |
| 151 | 1B | *IWB67338* | 58.9 | ns | *67336, 50463, 25098, 31677, 14452, 50530, IWA7234, 73015, 32306, 67337, 51878* |  |
| 152 | 1B | *IWB40109* | 59.2 | ns | *70974, 46989, 50050, 71800, 58817, IWA3295, IWA5546, IWA5673,72330 69041, 6103, 8612, 8257, 72329, IWA139, 73662, 73373, 71717, 5843, 71369, 68893* |  |
| 153 | 1B | *IWB69550* | 64.7 | ns |  |  |
| 154 | 1B | *IWB65744* | 65.0 | ns | *8296, 71872, 50261, 6519, IWA4849* |  |
| 155 | 1B | *IWA5348* | 65.6 | ns |  |  |
| 156 | 1B | *IWB40146* | 65.8 | ns | *56493, 9703, IWA8081, 74145, 65091, IWA6063, IWA792, 10646, IWA7119, 6921* |  |
| 157 | 1B | *Xbarc302* | 67.5 | ns | *51889* |  |
| 158 | 1B | *IWB5590* | 68.9 | ns | *IWA128* |  |
| 159 | 1B | *IWB2337* | 70.3 | ns |  |  |
| 160 | 1B | *IWB5511* | 71.4 | ns | *58266, 72533* |  |
| 161 | 1B | *IWB10551* | 71.7 | ns | *68429, IWA4197, 12316, IWA188 ,7443, 71512, 65360, 31711, 47412, 69496, 69495, 7992, 59187, IWA189, 47330, 60937, 31478, IWA890, 69494, 14999, 14021, IWA4198, 72532, 27169* |  |
| 162 | 1B | *IWB39521* | 73.1 | ns |  |  |
| 163 | 1B | *IWB35922* | 76.3 | ns |  |  |
| 164 | 1B | *IWB2788* | 78.9 | ns | *35028, 35412, 47134, 47132, 52443, 27767, 47135, 61058, 72645, 27097, 47136, 5590, 28554, 11607, IWA3307, IWA5278, IWA4987, 44100, 71511, 69973, 35581, 67838, 59128, 45235,10445, 17725, 30875, 34661, 69772 11284, 11168, 62738, 29292, 35864, 27095, IWA7017, 60913* |  |
| 165 | 1B | *IWB46080* | 79.7 | ns | *63932* |  |
| 166 | 1B | *Xwmc694* | 80.0 | ns |  |  |
| 167 | 1B | *IWB7183* | 80.0 | ns | *51971, 34504, 35239, IWA3945, 7324, 35577, 73484, 37331, 65731, 27300 IWA491* |  |
| 168 | 1B | *IWB65754* | 80.3 | ns | *27762, 46465, 7180* |  |
| 169 | 1B | *IWA270* | 82.5 | ns | *IWA554, IWA4316, 72738, 59481, IWA460* |  |
| 170 | 1B | *IWB66204* | 83.1 | ns | *7286, 72498* |  |
| 171 | 1B | *IWB68866* | 83.3 | ns | *72962, 68864, 35635, 68865, 72619, 6521, 70777, 68867, 70519* |  |
| 172 | 1B | *IWA4203* | 83.6 | ns | *IWA7982, 72078, IWA6917* |  |
| 173 | 1B | *IWB9661* | 85.6 | ns |  |  |
| 174 | 1B | *IWB26948* | 86.7 | ns |  |  |
| 175 | 1B | *IWB6215* | 86.7 | ns | *26949, 52353, 64848, 44429* |  |
| 176 | 1B | *IWA1231* | 87.5 | ns | *IWA4823* |  |
| 177 | 1B | *IWA775* | 87.5 | ns |  |  |
| 178 | 1B | *IWB13393* | 87.5 | ns |  |  |
| 179 | 1B | *IWB14729* | 87.5 | ns |  |  |
| 180 | 1B | *IWB31909* | 87.8 | ns | *29159, IWA540* |  |
| 181 | 1B | *IWB8507* | 88.1 | ns |  |  |
| 182 | 1B | *IWB39291* | 89.2 | ns | *34737, 39290, IWA6018, 10066, 32500* |  |
| 183 | 1B | *IWB73745* | 89.4 | ns | *35192, 71351, 40176, 20648, 26560, 73004, 72079, 34917, 71355, IWA7275, 39932, 68968, 40124, 73005* |  |
| 184 | 1B | *IWB57391* | 89.4 | ns | *10226 71530 3191 35474 39431 40125 71352 11968 31680* |  |
| 185 | 1B | *IWB73964* | 90.5 | ns | *71350, 3677, 71348, 71349* |  |
| 186 | 1B | *IWB28518* | 91.0 | ns | *56758, 11261, 4734, 31653, 73024, IWA8474, 5184, 73016, 11372, IWA7002.1, 12327, 31807* |  |
| 187 | 1B | *IWB57368* | 91.3 | ns |  |  |
| 188 | 1B | *IWB71902* | 91.6 | ns | *45575, 71596, 57213, 27998, 9175, 71904, 66244, 47923.1, IWA7317* |  |
| 189 | 1B | *IWB8999* | 91.9 | ns | *10880* |  |
| 190 | 1B | *IWB73514* | 92.7 | ns |  |  |
| 191 | 1B | *IWB58168* | 93.0 | ns |  |  |
| 192 | 1B | *IWB72552* | 93.2 | ns | *59152, 72551, 72549, 72550* |  |
| 193 | 1B | *IWB8187* | 93.5 | ns |  |  |
| 194 | 1B | *IWB71822* | 94.6 | ns | *75197, 73694, 35689, 47726, 72730* |  |
| 195 | 1B | *IWB64623* | 95.1 | ns |  |  |
| 196 | 1B | *IWB8944* | 95.4 | ns |  |  |
| 197 | 1B | *IWB70038* | 95.7 | ns | *70037, 70039, 29348, 29347* |  |
| 198 | 1B | *IWB61202* | 95.9 | ns | *64025, IWA4703, IWA4939, 50027, 34848, 40167, 2681, 67259* |  |
| 199 | 1B | *IWB7534* | 95.9 | ns | *9016, 64027, 11437, IWA4090, 65886, 7846, 48254, 48255, IWA4091, 7533 39199, 51921* |  |
| 200 | 1B | *IWB60916* | 96.7 | ns | *63960, 11589, 10104, 67865* |  |
| 201 | 1B | *IWB67348* | 97.0 | ns | *73156, 48469* |  |
| 202 | 1B | *IWB7151* | 97.3 | ns |  |  |
| 203 | 1B | *IWB8066* | 97.5 | ns | *46336, 10982* |  |
| 204 | 1B | *IWB35875* | 98.1 | ns | *IWA5382, 5761, IWA5383* |  |
| 205 | 1B | *IWB59663* | 98.9 | ns | *56771, 7446, 9781, 12322, 45213* |  |
| 206 | 1B | *IWB3067* | 99.2 | ns | *IWA4488, 40197, IWA8313, 44036, 8245, 7579* |  |
| 207 | 1B | *Xwmc134* | 99.8 | ns |  |  |
| 208 | 1B | *IWB12504* | 101.0 | ns |  |  |
| 209 | 1B | *IWB69351* | 101.3 | ns |  |  |
| 210 | 1B | *IWB63855* | 101.8 | ns | *63853, 7410, 9208, 73681, 63852, 26591, 58887* |  |
| 211 | 1B | *IWB72499* | 103.5 | ns |  |  |
| 212 | 1B | *IWB59602* | 104.9 | ns |  |  |
| 213 | 1B | *IWB60525* | 105.2 | ns | *66483* |  |
| 214 | 1B | *IWB9764* | 105.7 | ns | *29430* |  |
| 215 | 1B | *IWB9256* | 106.5 | ns |  |  |
| 216 | 1B | *IWB64093* | 107.6 | ns | *12356, 2667, 64037, 64094, 35479, IWA3341, 59930, 10243, 64091, 64092, 35726, 7290, 6709, IWA255* |  |
| 217 | 1B | *IWB31201* | 108.8 | * | *IWA5186, 10050, 11283, 11211, 12497, 26166, 45740, 58378, 3260, 26168, 26167, 31454, 65180, 58356, 4407, 58355* |  |
| 218 | 1B | *IWB72580* | 109.8 | * |  |  |
| 219 | 1B | *IWB72607* | 112.4 | ** | *7976, 73033, 73032, 6989, 10621* |  |
| 220 | 1B | *IWB35890* | 113.3 | ** | *11958* |  |
| 221 | 1B | *IWB5074* | 113.5 | ** | *3330, 35958* |  |
| 222 | 1B | *IWB29141* | 114.1 | ** | *13447, 57827, 74776, 27691, 56525* |  |
| 223 | 1B | *IWB37326* | 114.3 | ** | *13458, 10068* |  |
| 224 | 1B | *IWB11512* | 114.9 | ** |  |  |
| 225 | 1B | *IWB66919* | 115.7 | * | *5855, 66920* |  |
| 226 | 1B | *IWB12325* | 116.0 | ** | *60344, 12536, 46553.1, 6947, 46187* |  |
| 227 | 1B | *Xbarc188* | 122.2 | ns |  |  |
| 228 | 1B | *IWB66189* | 124.0 | ** | *29144, 34335, 43818, IWA5448, 31486, 36598, 70380, 73882, 34402, 66196, IWA3497, 66195, 66192, 25498, 25499, 66198, 44899, 66191.1, 70151, 66185, 65905, 66186* |  |
| 229 | 1B | *IWB35108* | 124.3 | ** |  |  |
| 230 | 1B | *IWB72240* | 124.6 | ** |  |  |
| 231 | 1B | *IWB60663* | 124.9 | *** | *IWA4031, IWA7141, 9116, 7872, 36125, 6213, 7871, 11390, 13106, 35340, 65867, 6777* |  |
| 232 | 1B | *IWB72142* | 125.7 | ** |  |  |
| 233 | 1B | *IWB66973* | 126.0 | * | *63739, 36563, 29151, 29150, 12399* |  |
| 234 | 1B | *IWB6805* | 127.4 | * | *5810, 10454, 66900* |  |
| 235 | 1B | *IWB57211* | 127.6 | * | *69649* |  |
| 236 | 1B | *IWB73698* | 127.9 | * | *46397* |  |
| 237 | 1B | *IWB8005* | 131.1 | * | *64154* |  |
| 238 | 1B | *IWB36293* | 131.3 | * | *11855, 15640, 73738, 73736, 73737, 8840, IWA8542* |  |
| 239 | 1B | *IWB7686* | 131.9 | * | *7459, 8409, 73299, 9182* |  |
| 240 | 1B | *IWB10962* | 132.7 | ** |  |  |
| 241 | 1B | *Xwmc44* | 139.7 | ** |  |  |
| 242 | 1B | *IWB31871* | 140.0 | * | *IWA7992, IWA8332, 73284, 11006, 11007, 72444, 35847, 72443, 36371, 27960, 69702* |  |
| 243 | 1B | *IWB27085* | 140.0 | * | *69701, 13473, 31174, 35119, 36268, 39347, 44883, 72561, 6507, 6619, 7869, 7870, 28819, IWA3783, 7868, 50473, IWA2558, IWA3998* |  |
| 244 | 1B | *IWB57628* | 140.2 | * | *7694, 31676, 43860, 59155, 71943, 57627, 29684* |  |
| 245 | 1B | *IWB4454* | 141.3 | * | *8096, 72031, 72029, 72030, 8097, 65512, 71885, 20729, 72033, 47986, 34935, IWA848, 14703, 71884, 71886, IWA3892, IWA846, 72032* |  |
| 246 | 1B | *IWB5732* | 146.0 | ** |  |  |
| 247 | 1B | *IWB9706* | 147.1 | ** | *8053, 4839, 5678, 7427, 11634, 34471* |  |
| 248 | 1B | *IWB35087* | 150.1 | ns |  |  |
| 249 | 1B | *IWB9772* | 150.7 | ns | *6488* |  |
| 250 | 1B | *IWB64238* | 151.2 | * |  |  |
| 251 | 1B | *IWA5758.1* | 152.0 | ns | *2673, 9040* |  |
| 252 | 1B | *IWB13057* | 154.6 | * | *70716, 7523, 36253, 72244, 74944, 72243, 9931, 72247, 72245, 70717* |  |
| 253 | 1B | *IWB68096* | 154.8 | ns | *71657, 15244, 46804.1, 9077, 27845, 46805, 9079, 9110, 35151, 68093.1, 27844, 65272, 6700, 46800, 26024, 68094.1, 6721.1, 3405* |  |
| 254 | 1B | *IWB31066* | 156.2 | * | *35036, 11265, 70859, 71898, 20993, 71971.1* |  |
| 255 | 1B | *IWB34435* | 156.7 | * |  |  |
| 256 | 1B | *Xwmc728* | 157.3 | ns |  |  |
| 257 | 1B | *IWB73657* | 158.1 | * | *73820, 7641* |  |
| 258 | 1B | *IWB12221* | 158.4 | * | *72218, 63988, 72626, 72625* |  |
| 259 | 2A | *IWB5945* | 0.0 | ns |  |  |
| 260 | 2A | *IWB72720* | 2.1 | ns | *73487, 14009, 73151* |  |
| 261 | 2A | *IWB7792* | 2.7 | ns | *11669* |  |
| 262 | 2A | *IWB12342.1* | 3.2 | ns |  |  |
| 263 | 2A | *IWB68947* | 4.1 | ns | *16988.1* |  |
| 264 | 2A | *IWB65498* | 4.6 | ns | *52199, IWA3468, 7056, 65522, IWA3469* |  |
| 265 | 2A | *IWB7398* | 4.9 | ns | *25480, 30883, 72492.1, 25949, 72490, 27886, 6671.1, 27885, 72491, IWA5340, 30801.1, 30800.1, IWA5423, 31328.1, 64569* |  |
| 266 | 2A | *Xbarc124* | 4.9 | ns |  |  |
| 267 | 2A | *Xgwm497* | 6.2 | ns |  |  |
| 268 | 2A | *IWA8091* | 9.0 | ns |  |  |
| 269 | 2A | *IWB6542* | 9.6 | ns | *35651, 10983, 64927.1* |  |
| 270 | 2A | *IWB60729* | 10.8 | ns | *60728.1, 7706.1, IWA965* |  |
| 271 | 2A | *IWB15066* | 27.8 | ns | *4006, 5525, 13159, 17580.1, IWA1242* |  |
| 272 | 2A | *IWB6086.1* | 29.0 | ns | *31703, 7112, 10238, 36297, 7053.,1 44249, 31705, 10837, 6164, 4418, 48508, 44250* |  |
| 273 | 2A | *IWB67305* | 37.3 | ns | *67308, 67304, 67306, 67307* |  |
| 274 | 2A | *IWB70098* | 40.0 | ns |  |  |
| 275 | 2A | *IWB67791* | 45.0 | ns | *67790* |  |
| 276 | 2A | *IWB72462* | 45.5 | ns | *72463, 10811* |  |
| 277 | 2A | *IWB8363* | 46.1 | ns | *8362* |  |
| 278 | 2A | *IWB28073* | 49.6 | ns |  |  |
| 279 | 2A | *Xwmc453* | 59.8 | ns |  |  |
| 280 | 2A | *IWB73038* | 60.0 | * |  |  |
| 281 | 2A | *IWB51951* | 60.9 | * |  |  |
| 282 | 2A | *IWB10490* | 63.2 | * |  |  |
| 283 | 2A | *IWB56992* | 65.5 | * |  |  |
| 284 | 2A | *IWA294* | 66.6 | * | *45777, IWA293, 73758, 25061, 9926, 71526, 73981.1, 27013, IWA5495, 50486, 44985, 71880, 71879, 8160.1, 8937* |  |
| 285 | 2A | *IWB10760* | 68.9 | * | *27190, IWA3569, 8331.1, 20811, 26960, 34544* |  |
| 286 | 2A | *IWB32310* | 69.1 | * | *4047.1, 12320, 28709, 68419, 68420, 45265, 36149, IWA8491, 65847* |  |
| 287 | 2A | *IWA994* | 75.2 | ** | *45445* |  |
| 288 | 2A | *IWB27678.1* | 75.7 | ** | *45406* |  |
| 289 | 2A | *IWA581* | 76.2 | ** | *69369* |  |
| 290 | 2A | *IWB11613* | 76.5 | ** |  |  |
| 291 | 2A | *IWB59332* | 80.4 | ns |  |  |
| 292 | 2A | *IWA3431* | 80.7 | ns |  |  |
| 293 | 2A | *Xgwm372* | 80.7 | ns |  |  |
| 294 | 2A | *IWA820* | 81.4 | ns | *2990, IWA336, 34501, 67490, IWA5305, 28385, 39681, 68556, 34366, 46662, 61262, 69340, 30726, IWA5273, 46663, 72435, IWA3368, 14023, 34874, 52548, IWA588, IWA5744, IWA4793, 51841, 65453, IWA3653, 34999, IWA5303, 35580, 57773, 72292, IWA5219, 45843, IWA5307, 73280, IWA5188, 74893, IWA3294, 57685, IWA5550, IWA7969, 35068, 46970, IWA309, IWA7464, 65363, 32405, 21103, IWA5272* |  |
| 295 | 2A | *IWB68288* | 81.9 | ns |  |  |
| 296 | 2A | *IWB7897* | 82.8 | ns |  |  |
| 297 | 2A | *Xbarc15* | 83.6 | ns |  |  |
| 298 | 2A | *IWA5293* | 84.5 | ns |  |  |
| 299 | 2A | *IWB72154* | 102.5 | ns |  |  |
| 300 | 2A | *IWB57080* | 108.5 | ns | *62635, IWA5449, 11139, 7051, 10432, 32664, 64479, 11175* |  |
| 301 | 2A | *IWA200* | 118.6 | ns |  |  |
| 302 | 2A | *IWA544* | 120.6 | ns |  |  |
| 303 | 2A | *IWB43829.1* | 122.7 | ns |  |  |
| 304 | 2A | *IWB58832* | 123.5 | ns | *IWA542, 35296.1* |  |
| 305 | 2A | *IWB11614* | 124.0 | ns |  |  |
| 306 | 2A | *IWA8377* | 124.6 | ns | *61299* |  |
| 307 | 2A | *IWB12036* | 126.8 | ns | *33700, 68084* |  |
| 308 | 2A | *IWB26154* | 130.4 | ns |  |  |
| 309 | 2A | *IWB70002* | 130.6 | ns |  |  |
| 310 | 2A | *IWB7714* | 130.9 | ns |  |  |
| 311 | 2A | *IWB11193* | 139.5 | * | *11796, 2843, 8088, 48026, 30740, 28504, 63995, 60759.1* |  |
| 312 | 2A | *IWA8385* | 145.5 | ns |  |  |
| 313 | 2A | *IWB10182* | 146.7 | ns | *8707, 8706, 32717* |  |
| 314 | 2A | *IWB46244* | 150.8 | ns | *IWA3752, 62594, IWA6931, 62592* |  |
| 315 | 2A | *IWB6121* | 151.7 | ns |  |  |
| 316 | 2A | *IWB71626* | 153.6 | ns |  |  |
| 317 | 2A | *IWB12586* | 154.7 | ns |  |  |
| 318 | 2A | *IWB10957* | 155.0 | ns | *72716, 72723, 73878, IWA3920, 10956, 72722, 6997, 44731, 72718, IWA3919, 73879, 44394.1, 11146* |  |
| 319 | 2A | *IWB5861* | 156.9 | ns | *36028* |  |
| 320 | 2A | *IWB34772* | 158.0 | ns | *71497, 26553, 64480* |  |
| 321 | 2A | *IWB70188* | 158.2 | ns |  |  |
| 322 | 2A | *IWB65315* | 159.1 | ns | *35910, 5740, 73568, 73663* |  |
| 323 | 2A | *IWB61119* | 160.4 | ns |  |  |
| 324 | 2A | *IWB45464* | 161.0 | ns | *3601, 39577, 36400* |  |
| 325 | 2A | *IWB3440* | 161.2 | ns | *7597* |  |
| 326 | 2A | *IWB56728* | 169.1 | ns |  |  |
| 327 | 2A | *IWB72840* | 170.2 | ns |  |  |
| 328 | 2A | *IWB59980* | 170.5 | ns |  |  |
| 329 | 2A | *IWB69132* | 172.5 | ns |  |  |
| 330 | 2A | *IWB9488* | 172.8 | ns | *29388, 35139, 39958, 26483* |  |
| 331 | 2A | *IWB6431* | 173.1 | ns | *34766* |  |
| 332 | 2A | *IWB72117* | 176.9 | ns |  |  |
| 333 | 2A | *IWB68598* | 179.4 | ns |  |  |
| 334 | 2A | *Xgwm526* | 179.4 | ns |  |  |
| 335 | 2A | *IWB14668* | 180.4 | ns |  |  |
| 336 | 2A | *IWB32878* | 180.6 | ns | *5752* |  |
| 337 | 2A | *IWB8941* | 180.9 | ns |  |  |
| 338 | 2A | *IWB45239* | 181.2 | ns | *35319* |  |
| 339 | 2A | *IWB46343* | 181.5 | ns | *46050* |  |
| 340 | 2A | *IWB72126* | 181.7 | ns | *72976, 6807, 44629, 72125, 10627* |  |
| 341 | 2A | *IWB46290.1* | 186.8 | ns | *60386.1* |  |
| 342 | 2A | *Xbarc122* | 187.7 | ns |  |  |
| 343 | 2A | *IWB39805.1* | 188.8 | ns | *7757, 6685.1, 34582.1* |  |
| 344 | 2A | *IWA4493* | 190.1 | ns | *IWA4491* |  |
| 345 | 2A | *IWB33959* | 190.4 | ns | *75252, 70684.1, 13647.1, 62573.1, 32029, 71298.1, IWA7327, 27789.1, 10465.1, IWA6963, 57850.1, 70340.1, 14834, 44619, 25355.1, IWA4463, 44005, 70730, 44454.1, 9316, 32909* |  |
| 346 | 2A | *IWB52585.1* | 192.1 | * |  |  |
| 347 | 2B | *IWB47158* | 0.0 | *** |  |  |
| 348 | 2B | *IWB34415* | 0.8 | *** |  |  |
| 349 | 2B | *IWB29273* | 1.3 | *** |  |  |
| 350 | 2B | *IWB9207* | 1.3 | **** |  |  |
| 351 | 2B | *IWB57906* | 1.6 | *** |  |  |
| 352 | 2B | *IWB6886* | 3.6 | ***** |  |  |
| 353 | 2B | *IWA7916* | 4.4 | ***** |  |  |
| 354 | 2B | *IWB46469* | 5.2 | ***** | *71940, IWA3868, IWA546, 37705, 73253, 44373, 2702, IWA1093, 26451* |  |
| 355 | 2B | *IWB59353* | 5.5 | ***** | *44381* |  |
| 356 | 2B | *IWB69396* | 6.6 | ***** |  |  |
| 357 | 2B | *IWB21237* | 7.2 | ***** |  |  |
| 358 | 2B | *IWB32008* | 8.1 | ***** | *70087, 71313, 39654* |  |
| 359 | 2B | *IWB71587* | 8.4 | ***** | *49945, 21394, 59021, 71586, 59678, 35359* |  |
| 360 | 2B | *IWB73904* | 8.7 | ***** |  |  |
| 361 | 2B | *IWA8243* | 9.6 | ***** | *IWA4652, 6159, 36310* |  |
| 362 | 2B | *IWA4554* | 9.8 | ***** | *71895, 45339, 35392, 60907, 69854, 28588, 45338, 67532, 13632, 13631, 68807* |  |
| 363 | 2B | *IWA6893* | 10.1 | **** | *65417, 28589, IWA6943, 16695, 73263, 73250, 72776, 74618, 47405, 68761, 73251, 35421, 31983, 66020, 69852, 27354, 67029, 31982, 69853, 27355, IWA6026, 73252, 36769, 68808, 45337* |  |
| 364 | 2B | *IWB46988* | 11.0 | ***** | *60107, 45403, 45402, 63381, 47187, 65311* |  |
| 365 | 2B | *IWB7072* | 11.6 | ***** | *45731, 50337, 31002, 39832, 70041, 36124, 7781, 72307, 31001, 34324, 7346, 65326, 71775* |  |
| 366 | 2B | *IWB60831* | 11.9 | **** |  |  |
| 367 | 2B | *IWB72894* | 12.7 | **** | *7481, 72760, 44618, 57346, 2380, 10024, 36550, 2338, 3877* |  |
| 368 | 2B | *IWB72685* | 13.0 | **** |  |  |
| 369 | 2B | *IWB74844* | 13.3 | **** | *8125, 74841, IWA4673, 7738, 8126, 8332* |  |
| 370 | 2B | *IWB26449* | 13.6 | **** |  |  |
| 371 | 2B | *IWB60118* | 13.9 | **** | *28282, 70581, 70580* |  |
| 372 | 2B | *IWB5684* | 14.4 | **** | *50438, 6075, 58252* |  |
| 373 | 2B | *IWB48240* | 15.0 | ** |  |  |
| 374 | 2B | *IWB62688* | 16.1 | *** | *58252, 65460, IWA7030.1, 39369, 35350, 36727, 10430, IWA7029, 4951, 73426, IWA5560* |  |
| 375 | 2B | *IWB11285* | 16.4 | *** |  |  |
| 376 | 2B | *Xbarc55* | 21.1 | * |  |  |
| 377 | 2B | *IWB16217* | 21.9 | ns | *32006, IWA8221, 32007, 60585, 32005, 11568* |  |
| 378 | 2B | *IWB3454* | 23.1 | ns |  |  |
| 379 | 2B | *IWB44515* | 23.9 | * | *44605, 28514, 34871, 12056, 2851,5 63624, IWA7076, 46789, 74180, 25795, 34613, 27957, 73197, 60825, 56586, 33921, 39220, 74064, 26224, 35295, 47398, 44975, 7335, 65565, 17890, 58343, 48351, 73834, 63655, 47512, 56587, 34771, 34545, 63625, 32296, IWA1204, 47399, 7331, 48352, 11527, 10670, 74443, 56514, 63682, 11184, 45082, 33642, 7069, 64803, 35158, 9540, 7825, 73030, 35020, 12063, 57695, 12041, 10408, 7334, 46325* |  |
| 380 | 2B | *IWB26631* | 26.4 | * | *29391* |  |
| 381 | 2B | *IWA905* | 28.8 | ns | *IWA6308, 74209* |  |
| 382 | 2B | *IWA3428* | 31.5 | ns | *IWA6075, 65378, 45219, 26859, 35009, 4614, 3996, IWA4102* |  |
| 383 | 2B | *IWB7715* | 34.2 | ns |  |  |
| 384 | 2B | *IWB36818* | 34.8 | ns | *14689* |  |
| 385 | 2B | *IWB64964* | 35.1 | ns |  |  |
| 386 | 2B | *IWB58274* | 35.4 | ns | *9584, IWA169, 65623, 8102, IWA4606, 59913, IWA4605, 4425* |  |
| 387 | 2B | *IWA5436* | 36.3 | ns | *IWA4100, 4546* |  |
| 388 | 2B | *IWB2692* | 37.2 | ns | *IWA7520, 2691, 34732, 3357, 65990* |  |
| 389 | 2B | *IWB31492* | 37.4 | ns |  |  |
| 390 | 2B | *IWB59779* | 39.5 | ns |  |  |
| 391 | 2B | *IWB65533* | 40.0 | ns |  |  |
| 392 | 2B | *IWB45931* | 40.6 | ns |  |  |
| 393 | 2B | *IWA3817* | 40.9 | ns | *43921, 74656.1, 25870, 64603.1* |  |
| 394 | 2B | *Xbarc18* | 41.5 | ns |  |  |
| 395 | 2B | *IWB34576* | 42.5 | ns | *49966, IWA3995* |  |
| 396 | 2B | *IWB57577* | 43.1 | ns |  |  |
| 397 | 2B | *IWB74647* | 43.4 | ns | *60896, 47381, 39777, 21312, 21074* |  |
| 398 | 2B | *IWB74518* | 43.9 | ns | *59508, IWA5256* |  |
| 399 | 2B | *Xgwm129* | 45.1 | ns |  |  |
| 400 | 2B | *IWA5600* | 46.0 | ns | *71012, 61024, IWA7146, IWA4106, IWA6000, IWA5659, IWA7524, IWA4107, IWA4136, IWA7499, IWA5653* |  |
| 401 | 2B | *IWB56465* | 47.4 | ns |  |  |
| 402 | 2B | *IWB62579* | 47.7 | ns | *IWA6016, 48056, 48057, IWA586, IWA587, IWA1215, IWA772, 2679, 9833, 69314, IWA1216* |  |
| 403 | 2B | *IWB2507* | 48.0 | ns | *30844, IWA6921, 65488, IWA6240, IWA6948, IWA7015, 2458, IWA4517, 57693, 3491, IWA7195, IWA3840, 39200, 11319, 4321, 34486, 62651* |  |
| 404 | 2B | *IWB67747* | 52.1 | * | *34793* |  |
| 405 | 2B | *IWB69363* | 52.4 | * | *IWA7019, 72986, 47895, 69329, 69362, 71212* |  |
| 406 | 2B | *IWB26325* | 52.6 | * | *51844, 68278, 39834, 32588* |  |
| 407 | 2B | *IWB63790* | 54.9 | ** |  |  |
| 408 | 2B | *IWB12137* | 57.3 | ** |  |  |
| 409 | 2B | *IWB48071* | 61.3 | ** | *52584, 50067* |  |
| 410 | 2B | *IWB43954* | 61.6 | *** |  |  |
| 411 | 2B | *IWB60374* | 63.9 | ** | *21139, 48280, 49862, 21199, 21140, IWA4256, 28486, 30854, 47243, 35867, IWA5397, 48279, 46616, 70825, 34696, 73022, IWA5513, 30933, 48278, 27165, 60373, 27166, 49783, 47244, 45631, 59086, 73040, 62675, 28487, IWA5512, 25132, 70826, 60039* |  |
| 412 | 2B | *IWA5461* | 64.5 | ** |  |  |
| 413 | 2B | *IWB50645* | 64.8 | ** |  |  |
| 414 | 2B | *IWB28721* | 65.1 | ** | *21466* |  |
| 415 | 2B | *IWB66390* | 65.6 | *** | *6859* |  |
| 416 | 2B | *IWB11563* | 65.9 | *** | *12239, 72407, 32250, 9310, 64322, 10411, 65589, 72411, 32140, 14419, 45046, 64321, 52020* |  |
| 417 | 2B | *IWB10410* | 66.2 | ** |  |  |
| 418 | 2B | *IWB6515* | 66.5 | *** | *70307* |  |
| 419 | 2B | *IWB52433* | 66.7 | *** | *65526, 6383, 32028, 67744, 62910, 32027* |  |
| 420 | 2B | *IWA5413* | 67.0 | **** | *28591* |  |
| 421 | 2B | *IWA470* | 67.9 | ** |  |  |
| 422 | 2B | *IWB3537* | 69.0 | *** |  |  |
| 423 | 2B | *IWB68982* | 69.8 | **** | *69070, 62876, 44765* |  |
| 424 | 2B | *IWB11092* | 70.1 | **** | *IWA8478* |  |
| 425 | 2B | *IWB35393* | 70.4 | **** |  |  |
| 426 | 2B | *IWB12045* | 73.0 | ***** |  |  |
| 427 | 2B | *IWB3891* | 75.3 | **** | *47664, IWA3395, IWA4358* |  |
| 428 | 2B | *IWB73114* | 75.9 | *** |  |  |
| 429 | 2B | *Xwmc175* | 77.8 | *** |  |  |
| 430 | 2B | *IWB3192* | 79.8 | **** | *73172, 25798* |  |
| 431 | 2B | *IWB72911* | 79.8 | ***** |  |  |
| 432 | 2B | *IWB67207* | 81.2 | ***** | *31274, 67208* |  |
| 433 | 2B | *IWB4571* | 81.8 | ***** | *12298, 11177, 26189, 26191, 2982, 4443, 9247, 10915, 11451, 36062, 6270, 6631, 12230, 63030, 32165, 60670, 48248, 5436, IWA6453,15068, IWA4294, 2985, 6150, 8789, 34949, 63235, 74910, 49117, IWA3742, 28807, 68671, IWA3741* |  |
| 434 | 2B | *IWA6122* | 83.8 | **** | *5627, 56896, 64172, 65989, 73178, 73960, 36126, 36140, 60406, 6567, 57723, IWA7371, IWA4095, IWA4096, 9873, 35840, 68283, 35839, 32189, 43934, 46242, 40156, 52451, 6424,5 64246, 4424, 43935, 48012, 28363, 32190, 26742, IWA8295, 66366, 73961, 66367, IWA4098, IWA4097* |  |
| 435 | 2B | *IWB57922* | 85.3 | *** |  |  |
| 436 | 2B | *IWB28722* | 86.2 | **** |  |  |
| 437 | 2B | *IWB8420* | 86.8 | **** | *34633, 8419, 25695* |  |
| 438 | 2B | *IWB3588* | 87.9 | ***** | *34658, 58059, 58061, 11942, 63970, 58060, 47959, 3648* |  |
| 439 | 2B | *IWB68952* | 89.6 | **** |  |  |
| 440 | 2B | *IWB36072* | 90.1 | **** | *67760* |  |
| 441 | 2B | *IWB73211* | 90.7 | *** |  |  |
| 442 | 2B | *IWB37873* | 91.0 | *** |  |  |
| 443 | 2B | *IWB67728* | 93.0 | *** | *46174.1, 9044.1, 27775 ,73441, 34377, 67729, 8054, 72385, 70765, 61305, 60671, IWA7955* |  |
| 444 | 2B | *IWB71878* | 93.3 | *** | *IWA7909, 56915, IWA3564* |  |
| 445 | 2B | *IWB39394* | 94.4 | *** | *68692* |  |
| 446 | 2B | *IWB9560* | 94.7 | *** |  |  |
| 447 | 2B | *IWB5957* | 95.0 | *** | *49828, 73567, 49827.1, 69430, 73566, 69431.1, 49829* |  |
| 448 | 2B | *IWB44694* | 95.5 | *** | *48386.1, 58646.1, 29532, 9328* |  |
| 449 | 2B | *IWB69630* | 104.1 | *** | *57664, 57663, 69628, 49829, 69631, 4648 ,7050, IWA8534, 25868, 25869, 58747.1, IWA8449, 63201.1* |  |
| 450 | 2B | *IWB6167* | 104.3 | *** | *72654* |  |
| 451 | 2B | *IWB60041* | 105.2 | *** |  |  |
| 452 | 2B | *IWB68445* | 105.4 | *** |  |  |
| 453 | 2B | *IWB57292* | 106.3 | ** | *59956.1, 35179* |  |
| 454 | 2B | *IWB6323* | 106.6 | ** | *29332, 11888, 14959, 57313, 14677.1, 32119, 26011* |  |
| 455 | 2B | *IWB45435* | 107.7 | *** |  |  |
| 456 | 2B | *IWB11174.1* | 114.1 | ** |  |  |
| 457 | 2B | *IWB10162* | 114.4 | ** |  |  |
| 458 | 2B | *IWB66266* | 114.7 | ** | *12574* |  |
| 459 | 2B | *IWB2341* | 115.5 | ** | *2482, 7671* |  |
| 460 | 2B | *IWB52447* | 116.6 | ** | *71976, 4865, 71975* |  |
| 461 | 2B | *IWB11123* | 116.9 | ** | *28651* |  |
| 462 | 2B | *IWB59226* | 117.1 | ** | *35156, 36286, 39104* |  |
| 463 | 2B | *IWB58126* | 117.4 | ** | *8813, 36279, 10706, 5864, 56526, 6113, 46445.1* |  |
| 464 | 2B | *IWB2628* | 117.9 | ** | *13680* |  |
| 465 | 2B | *IWB9088* | 119.1 | ** |  |  |
| 466 | 2B | *IWB60161* | 119.3 | ** |  |  |
| 467 | 2B | *IWB34773* | 119.6 | ** | *26482, 32262, 50096, 47010, 46560* |  |
| 468 | 2B | *IWB8894* | 119.9 | ** | *5976, 33668, 33669, 24984* |  |
| 469 | 2B | *IWB69000* | 123.4 | * | *68998, 68997* |  |
| 470 | 2B | *IWB64039* | 126.7 | *** |  |  |
| 471 | 2B | *IWA3257* | 128.8 | ** |  |  |
| 472 | 2B | *IWB39800* | 129.0 | ** |  |  |
| 473 | 2B | *IWB70683* | 129.3 | ** |  |  |
| 474 | 2B | *IWB32211* | 130.8 | ** | *4318, 66206, 72277, 4319, 72278, 66207* |  |
| 475 | 2B | *IWB7819* | 131.6 | ** | *IWA8055, IWA4422* |  |
| 476 | 2B | *IWB6618* | 132.2 | ** | *IWA3982* |  |
| 477 | 2B | *Xwmc317* | 134.4 | ns |  |  |
| 478 | 2B | *IWB8157* | 136.4 | ** | *10173, 62759, 65625, 56582, 5978, 7106 ,21105, 32143, 36136, 73565, 62757, 62763, 11295* |  |
| 479 | 2B | *IWB36313* | 136.4 | ** |  |  |
| 480 | 2B | *IWB58205* | 138.2 | *** |  |  |
| 481 | 2B | *IWB7569* | 140.6 | ** | *7625, 12117, 48388, 32245, 12415, 66438, 59762, 69343, IWA4658, 7580, 29461, 34450, 46858, 31257, 7624, 14135, 70778, 65734* |  |
| 482 | 2B | *IWA5442* | 140.6 | *** |  |  |
| 483 | 2B | *IWB25347* | 143.0 | ***** |  |  |
| 484 | 2B | *IWB44797* | 146.0 | *** |  |  |
| 485 | 2B | *IWB9733* | 146.9 | ** | *28691* |  |
| 486 | 2B | *IWB8698* | 147.2 | ** | *7605, IWA3315, 74736, 11333, 24927.1, 65020, IWA988* |  |
| 487 | 2B | *IWB45312* | 147.4 | ** | *68447, 6341, 15636* |  |
| 488 | 2B | *IWB52360* | 147.7 | *** |  |  |
| 489 | 2B | *IWB58090* | 151.7 | ** |  |  |
| 490 | 2B | *IWB7893* | 152.0 | * |  |  |
| 491 | 2B | *IWB12598* | 152.5 | * | *48199, 35507, 35005* |  |
| 492 | 2B | *IWB8650* | 152.8 | * | *11314, 11856.1, 13375.1, 6563, 57127, IWA5192.1, 60398, 10841, 11171, 63909, 46025.1, 8633* |  |
| 493 | 3A | *Xbarc321* | 0.0 | ns | *74975, 60480, 11846, 29214, 11362, 3127.1, 10398.1, 67382, 28209, 27973, 8502, IWA447, 75018, 73895, 60417, 35638, 10820, 8501, 60283, 3850, 7136.1, 7261, 4691, 10973.1, 18431, 49840, 4690 ,3128, 2820* |  |
| 494 | 3A | *IWB36703.1* | 1.3 | ns | *11852* |  |
| 495 | 3A | *IWB12111.1* | 2.7 | ns |  |  |
| 496 | 3A | *IWB5950* | 3.3 | ns | *48009.1, 29389, 65500, 50684, 50683, 50548, 12993, 60531, IWA8280, 46438, 28726, 44955, 11084, 7037, 7003, 12606, IWA8587, 12994, 12170, 5952, 34333, 11377, 8658, 12968, 12607, 10638, 60530, 12967, 12225* |  |
| 497 | 3A | *IWB7786.1* | 3.6 | ns |  |  |
| 498 | 3A | *IWB9278* | 4.5 | ns | *29131* |  |
| 499 | 3A | *IWB5333* | 5.0 | ns |  |  |
| 500 | 3A | *IWB65471* | 11.0 | ns |  |  |
| 501 | 3A | *IWB72257* | 11.8 | ns |  |  |
| 502 | 3A | *IWB26667* | 14.1 | ns | *IWA8106, 26668, IWA3939, 34361, IWA8105* |  |
| 503 | 3A | *IWA4804* | 17.6 | ns |  |  |
| 504 | 3A | *IWB8714* | 17.9 | ns | *8715, 14876, IWA4781.1, 29612* |  |
| 505 | 3A | *IWB25484* | 18.7 | ns | *58561* |  |
| 506 | 3A | *IWA288.1* | 20.2 | ns |  |  |
| 507 | 3A | *IWA6928* | 31.6 | ns |  |  |
| 508 | 3A | *IWB45729.1* | 33.9 | ns |  |  |
| 509 | 3A | *IWB70095* | 34.2 | ns | *25465, 70096, 44091, 44089* |  |
| 510 | 3A | *IWB67571* | 41.3 | ns |  |  |
| 511 | 3A | *IWB73247* | 41.6 | ns | *72638* |  |
| 512 | 3A | *IWB32591* | 42.1 | ns | *44601, 15006, 47000* |  |
| 513 | 3A | *IWB34845* | 42.4 | ns | *44372* |  |
| 514 | 3A | *IWB14340* | 42.9 | ns |  |  |
| 515 | 3A | *IWB6837* | 50.6 | ns | *IWA4675, 73640* |  |
| 516 | 3A | *IWB71479* | 54.2 | ns | *63578, 71480, 71477* |  |
| 517 | 3A | *IWB9991* | 55.8 | ns | *9990, 73757, 50038* |  |
| 518 | 3A | *IWB72544* | 57.0 | ns |  |  |
| 519 | 3A | *IWB46342* | 57.2 | ns | *73868* |  |
| 520 | 3A | *IWB71974* | 58.4 | ns |  |  |
| 521 | 3A | *Xgwm5* | 61.1 | ns |  |  |
| 522 | 3A | *IWB67595* | 64.7 | ns |  |  |
| 523 | 3A | *IWB72484* | 65.2 | ns | *9076, 68071* |  |
| 524 | 3A | *IWB39542* | 65.8 | ns | *IWA5616, 20642, 67553* |  |
| 525 | 3A | *IWB39272* | 66.6 | ns | *67857, 39273* |  |
| 526 | 3A | *IWB72529* | 67.2 | ns |  |  |
| 527 | 3A | *IWB37891* | 67.4 | ns | *29267, IWA6204, IWA6229, 48393, 14697* |  |
| 528 | 3A | *IWB65628* | 67.7 | ns |  |  |
| 529 | 3A | *IWB4947* | 68.0 | ns | *6891.1, 48477* |  |
| 530 | 3A | *IWA538* | 70.6 | ns | *8262, 16112, IWA8590, 31065, IWA5617, 35157, 27368, IWA6108, 71668, 58686, IWA143, IWA4912, IWA8283, 29336, 37650, 2801, IWA4172, IWA3498, IWA3535, IWA4913, 20753* |  |
| 531 | 3A | *IWB27964* | 70.8 | ns | *58191, 71028* |  |
| 532 | 3A | *IWA7012* | 71.1 | ns |  |  |
| 533 | 3A | *IWA536* | 71.4 | ns | *66604, 45727, 68422* |  |
| 534 | 3A | *IWB5680* | 74.6 | ns |  |  |
| 535 | 3A | *IWB69157* | 75.1 | ns | *67654* |  |
| 536 | 3A | *IWB72777* | 75.9 | ns | *74968, 74967, IWA3376* |  |
| 537 | 3A | *IWB72687* | 76.5 | ns | *9725, 3645, 65798, IWA5632, 57248, IWA7355, IWA1279, 14015, 35097,, 60253, 72560, IWA7476, IWA4917, 70313, 73850, IWA1019, 50023, 50071, IWA4883, 36660, IWA3929, 35463, 3210, 20961, 69595, 62575, IWA8061, IWA3836, 72686, 2704, 72075, IWA6170, 63064, 72621, 65564, 9982, 72652, IWA4075, 72763, IWA234, 56927, 67049, 25115, 8196, 9012, 72653, 73647, 65513, 10592, 33677, 47447* |  |
| 538 | 3A | *IWB45790* | 77.6 | ns |  |  |
| 539 | 3A | *IWA8465* | 77.9 | ns | *62796.1, 70411, 72139, 70165, 10508, 32801* |  |
| 540 | 3A | *IWB72223* | 79.3 | ns | *72224, IWA8272* |  |
| 541 | 3A | *IWB66938* | 79.6 | ns | *66939* |  |
| 542 | 3A | *IWB26728* | 80.2 | ns | *11027, 7381, 52157.1, 34172, 12173, IWA6914, IWA6913, 35534, 21475, 34552, 35184, 33344* |  |
| 543 | 3A | *IWA7159* | 81.0 | ns | *IWA7877, 12317, IWA5286, 52500* |  |
| 544 | 3A | *IWB72822* | 83.0 | ns | *35578* |  |
| 545 | 3A | *IWB67254* | 84.7 | ns | *IWA8435* |  |
| 546 | 3A | *Xwmc428* | 88.1 | ns |  |  |
| 547 | 3A | *IWB39719* | 90.4 | ns | *52753, 6177, 5446, 39720* |  |
| 548 | 3A | *IWB72995* | 93.6 | ns | *72994, 72993* |  |
| 549 | 3A | *IWB67653* | 94.7 | ns |  |  |
| 550 | 3A | *IWB9778* | 95.3 | ns |  |  |
| 551 | 3A | *Xbarc1040* | 96.7 | ns |  |  |
| 552 | 3A | *IWB65532* | 100.3 | ns |  |  |
| 553 | 3A | *IWB73344* | 100.3 | ns | *73345* |  |
| 554 | 3A | *IWB28080* | 113.6 | ns | *IWA4298, 36101, IWA4296* |  |
| 555 | 3A | *IWB26279* | 115.9 | ns | *13444, 27647, 49970, 65934, 27648* |  |
| 556 | 3A | *IWA5456* | 116.1 | ns |  |  |
| 557 | 3A | *IWA4810* | 116.4 | ns |  |  |
| 558 | 3A | *IWB58368* | 116.7 | ns |  |  |
| 559 | 3A | *IWB10607* | 117.3 | ns | *8806* |  |
| 560 | 3A | *IWA7324* | 118.8 | ns |  |  |
| 561 | 3A | *IWB17683* | 119.0 | ns | *26056, 35794, IWA5596, IWA7169, IWA5419, 6655, 12852, 60875, 57435, 63185, 74979, 36056, 47989, IWA623, 35440, 35470* |  |
| 562 | 3A | *IWB4406* | 122.9 | ns |  |  |
| 563 | 3A | *IWB8584* | 124.9 | ns | *58868, 35917* |  |
| 564 | 3A | *IWB7486* | 128.7 | ns | *68483, 2973* |  |
| 565 | 3A | *IWB11581* | 129.3 | ns | *11580, 73103, 72897, 18010, 73102, 35125, 63055, 73101, 3798* |  |
| 566 | 3A | *IWB6047* | 130.2 | ns |  |  |
| 567 | 3A | *IWB72394* | 136.7 | ns |  |  |
| 568 | 3A | *IWB10433* | 139.4 | ns | *6993, 12566* |  |
| 569 | 3A | *IWB8816* | 140.3 | ns |  |  |
| 570 | 3A | *IWB25213* | 140.6 | ns | *5927, 11738, 8645, 20737, 4110, 26085, 46039, 31324, 39922.1, 47680, 20736, 36105, 57733* |  |
| 571 | 3A | *IWB31321* | 144.7 | ns | *63323, 52569, 7623* |  |
| 572 | 3A | *IWB50481* | 145.2 | ns |  |  |
| 573 | 3A | *IWB7073* | 145.8 | ns | *39724, 7543, 39723, 49995* |  |
| 574 | 3A | *Xwmc153* | 146.1 | ns |  |  |
| 575 | 3A | *IWB71198* | 154.3 | ns |  |  |
| 576 | 3A | *IWB73551* | 154.8 | ns |  |  |
| 577 | 3A | *IWB72058* | 156.0 | ns | *58806* |  |
| 578 | 3A | *IWB72476.1* | 172.3 | ns | *70483, 33546* |  |
| 579 | 3A | *IWB52089* | 174.3 | ns |  |  |
| 580 | 3A | *Xcfa2076* | 174.3 | ns |  |  |
| 581 | 3A | *IWB9606* | 175.2 | ns |  |  |
| 582 | 3A | *IWA391* | 178.0 | ns |  |  |
| 583 | 3A | *IWA95* | 186.1 | ns |  |  |
| 584 | 3A | *IWB8784.1* | 196.0 | ns | *12017* |  |
| 585 | 3A | *IWB44666.1* | 196.3 | ns | *29489, 35582, 35035, 66063.1* |  |
| 586 | 3A | *IWB4391* | 199.4 | ns | *14312, 67246.1, 28841* |  |
| 587 | 3A | *IWB72092* | 199.9 | ns | *67819, 2753, 11436, IWA5755, 70196, 12268, 35872, 11435, 10770, 65706, 28780, 67817* |  |
| 588 | 3A | *IWB73076* | 200.5 | ns | *72045, 73079, 73080, 7722.1, 73078, 73081.1, 73077.1, 73075.1, 72043, 72044* |  |
| 589 | 3A | *IWA7158* | 202.1 | ns |  |  |
| 590 | 3A | *IWB73267* | 202.7 | ns | *IWA4407, 73266, 3584, 73716, 72446, 9186, 63934, 66168, 69167, 73715, 73851, 69399, 72216, 72217, 63935* |  |
| 591 | 3A | *Xgwm162* | 203.8 | * |  |  |
| 592 | 3A | *IWB63588* | 204.9 | ns |  |  |
| 593 | 3A | *IWB36083* | 206.0 | ns |  |  |
| 594 | 3B | *IWB29059* | 0.0 | ns |  |  |
| 595 | 3B | *IWB46537* | 1.7 | ns | *63795, 8962* |  |
| 596 | 3B | *IWB4668* | 2.7 | ns |  |  |
| 597 | 3B | *Xbarc12* | 4.0 | ns |  |  |
| 598 | 3B | *IWB58161.1* | 5.2 | ns |  |  |
| 599 | 3B | *IWB5600* | 6.2 | ns |  |  |
| 600 | 3B | *IWB20761* | 7.6 | ns |  |  |
| 601 | 3B | *IWA6038* | 8.4 | ns | *14081.1, 58562.1, 66968* |  |
| 602 | 3B | *IWB66471* | 27.7 | ***** | *11617, 7204, 6979* |  |
| 603 | 3B | *IWB7693.1* | 37.6 | * |  |  |
| 604 | 3B | *IWB58514* | 41.6 | ns | *74350, 70524, 47239.1, 28481, 71522.1, 26544.1, 13387.1, 72064,11873, 65347, 73423* |  |
| 605 | 3B | *Xgwm389* | 41.6 | * |  |  |
| 606 | 3B | *IWB69147* | 41.9 | ns | *32711, 47165.1, 69146, 35950, 6062, 7355, 73424, 34636, 56684.1, 71225, 35226, 8756, 72020, 5790, 3843, 67769, 14676, 12193, 6573, 8745, 8755, 71813, 71814, 64176, 64989, IWA4796, 67388, 56857, 12194, 50465, 3027, 24980, 25867, 63733, IWA4654, 2828, 73916, 26152, 62893, 68698, 3026, 11923, 75112, 74599, 6491* |  |
| 607 | 3B | *IWB8528.1* | 43.0 | ns | *31170.1, 67389, 67771.1, 36021, 6145* |  |
| 608 | 3B | *IWB6475* | 43.6 | * |  |  |
| 609 | 3B | *IWB4343* | 43.9 | ns | *64968* |  |
| 610 | 3B | *IWB74288* | 44.2 | ns |  |  |
| 611 | 3B | *IWB58878* | 44.5 | ns | *34624, 72959* |  |
| 612 | 3B | *IWA5347* | 44.7 | ns | *50547, 70625, 67376, 60533, 19201, 18164, 6054, 12253, 7919, 24979* |  |
| 613 | 3B | *IWB45836* | 51.7 | * | *64166, 52743, 57156, 74456* |  |
| 614 | 3B | *IWB6299* | 52.2 | ns | *6673* |  |
| 615 | 3B | *IWB9212* | 55.1 | ns | *9415* |  |
| 616 | 3B | *IWB39782* | 55.4 | ns | *IWA3260* |  |
| 617 | 3B | *IWB8163* | 59.7 | ns |  |  |
| 618 | 3B | *IWB7464* | 60.0 | ns | *8751, 52159* |  |
| 619 | 3B | *Xcfd79* | 60.9 | ns | *74008, 74007, 45078* |  |
| 620 | 3B | *IWB8426* | 61.2 | ns |  |  |
| 621 | 3B | *IWB34803* | 62.0 | ns |  |  |
| 622 | 3B | *IWB17577* | 62.3 | ns | *67002* |  |
| 623 | 3B | *IWB12216* | 62.9 | ns |  |  |
| 624 | 3B | *Xbarc131* | 64.4 | ns |  |  |
| 625 | 3B | *IWB10462* | 68.9 | ns |  |  |
| 626 | 3B | *IWB39127* | 72.2 | ns |  |  |
| 627 | 3B | *IWB56689* | 97.3 | **** |  |  |
| 628 | 3B | *IWB25180* | 98.1 | **** | *62638, IWA7860.1, 2481, 7068 ,37541, IWA6201, 48161, 64951, IWA6202, 29321, 59419, IWA8326, 64517, 25051, IWA6200, 3565, 13605, 60996, 26756, 47168, IWA186, 44919, IWA6185, 48116, 46612, 64601, IWA1266, 50676, 58793, 66203* |  |
| 629 | 3B | *Xgwm566* | 98.8 | *** |  |  |
| 630 | 3B | *Xbarc68* | 98.8 | **** |  |  |
| 631 | 3B | *IWB56601* | 99.9 | *** |  |  |
| 632 | 3B | *IWA3390* | 101.0 | *** | *52841, 52842, 26447, 52840, 29116, 6813* |  |
| 633 | 3B | *IWB7234* | 101.9 | *** | *72222, 8803, 10783, 57061, 8986, IWA4838, 8802, 4907, 45902, 48217, 59313, 73520, 57066, 57065, 48218, 45903* |  |
| 634 | 3B | *IWB44056* | 101.9 | *** | *47348* |  |
| 635 | 3B | *IWA747* | 103.3 | *** |  |  |
| 636 | 3B | *IWB26916* | 104.4 | *** | *71972, 60598* |  |
| 637 | 3B | *IWB34239* | 104.7 | ** | *34238.1, 35437, 35458, 39511, 5120, 6626* |  |
| 638 | 3B | *IWB46180* | 105.3 | *** |  |  |
| 639 | 3B | *IWA3426* | 106.1 | **** |  |  |
| 640 | 3B | *IWB16995.1* | 107.2 | *** | *58218* |  |
| 641 | 3B | *IWB32713* | 108.9 | *** |  |  |
| 642 | 3B | *IWB26261* | 109.5 | **** | *28825* |  |
| 643 | 3B | *IWB27781* | 109.5 | *** | *6079, 34153, 62905, 7439, 65864, 65924, IWA6238, 63252, 35832, IWA6920* |  |
| 644 | 3B | *IWB7518* | 111.5 | *** | *12551, 60844, 67915, 67918, 9544, 67916* |  |
| 645 | 3B | *IWB57107* | 112.9 | *** |  |  |
| 646 | 3B | *IWB50286* | 113.2 | *** | *34161, IWA4054, 71531, 25874, 47020, 34160, 69999, IWA3788* |  |
| 647 | 3B | *IWB34974* | 114.1 | **** | *35616, IWA4755, IWA6165, 9734, 14946, 71405, 8111, IWA4310, 7412, 36039, 11463, IWA7294, 71608, IWA4040, IWA4226, IWA1496, 66513, 43862, 65260* |  |
| 648 | 3B | *IWB25473* | 115.8 | ** |  |  |
| 649 | 3B | *IWB66842* | 119.3 | ** |  |  |
| 650 | 3B | *IWB72168* | 120.7 | ** |  |  |
| 651 | 3B | *IWB14775* | 121.6 | *** |  |  |
| 652 | 3B | *Xwmc625* | 123.5 | ** |  |  |
| 653 | 3B | *IWA628* | 125.7 | *** | *73922, IWA629, 27206, 56471, 73051, 37878* |  |
| 654 | 3B | *IWB35464* | 126.6 | ** | *IWA211, IWA210, 65362* |  |
| 655 | 3B | *IWB72454* | 127.4 | * | *72057, 72456, 72453* |  |
| 656 | 3B | *IWB66740* | 128.0 | ** | *35493* |  |
| 657 | 3B | *IWB64191* | 128.8 | *** |  |  |
| 658 | 3B | *IWB3505* | 129.7 | ** |  |  |
| 659 | 3B | *IWB11389* | 130.0 | *** | *74433, 74434* |  |
| 660 | 3B | *IWB67963* | 130.2 | *** | *11913* |  |
| 661 | 3B | *IWB7782* | 130.8 | *** | *45103, 59103, 45058, 11270, 35074, 66016, 35272, 35331, 45059, 58687, 36387, 40103, 45112, 60017* |  |
| 662 | 3B | *IWB2766* | 131.6 | **** |  |  |
| 663 | 3B | *IWA4630* | 132.2 | *** |  |  |
| 664 | 3B | *IWB63191* | 132.5 | *** | *34827, 3264* |  |
| 665 | 3B | *IWB10691* | 132.7 | **** | *65116, 67789, IWA537, 58223* |  |
| 666 | 3B | *IWB65234* | 133.3 | **** | *26723* |  |
| 667 | 3B | *IWB69709* | 133.6 | *** |  |  |
| 668 | 3B | *IWB61144* | 134.4 | **** | *65080, 8616* |  |
| 669 | 3B | *IWB4885* | 134.7 | **** | *IWA898, 32129* |  |
| 670 | 3B | *IWB27586* | 135.5 | ***** | *12061, 9888, 12062, 56431, 52028* |  |
| 671 | 3B | *IWB5754* | 136.6 | **** | *44228* |  |
| 672 | 3B | *IWB73183* | 137.2 | **** | *73591, 67009, 3106, 56881, 73357, 73181, 73182, 72928* |  |
| 673 | 3B | *IWB72421* | 137.5 | **** |  |  |
| 674 | 3B | *IWB8522* | 138.0 | **** |  |  |
| 675 | 3B | *IWB3526* | 138.5 | **** | *60623, 3723, 26579, 11632, 35908, 11633, 60906, 10992, 8797, 9815, 44593, 8347, 27395, 25775.1* |  |
| 676 | 3B | *IWB9814* | 139.1 | ***** |  |  |
| 677 | 3B | *IWB65401* | 140.5 | ***** | *IWA7035* |  |
| 678 | 3B | *IWA7353* | 140.8 | ***** |  |  |
| 679 | 3B | *IWA8196* | 141.1 | ***** | *IWA7519, 32489* |  |
| 680 | 3B | *IWB12493* | 142.2 | ***** | *28943, 27863, 59348, 27759* |  |
| 681 | 3B | *IWB65220* | 143.2 | ***** |  |  |
| 682 | 3B | *IWA239* | 143.7 | ***** | *3480, 27905, 27904, 36172, 27903, 7903, 29033, 57595, IWA5626, 36679, 65344, 27902, 27903, 59708* |  |
| 683 | 3B | *IWB10905* | 144.0 | ***** | *28150* |  |
| 684 | 3B | *IWB71946* | 144.9 | ***** | *71968, 72987, 73084, 7999, 34786, 36059, 14043, 36652, 46341, 71969, 72040, IWA3306, 60466, 25044, IWA3305, 6001, 28853, IWA4721, 58666, 47499, 31045, 71945, 28184, 69438, 64938, 25739* |  |
| 685 | 3B | *IWB71475* | 145.4 | ***** | *66519, 71476* |  |
| 686 | 3B | *IWB32558* | 145.7 | ***** |  |  |
| 687 | 3B | *IWB46996* | 149.4 | ***** | *73162* |  |
| 688 | 3B | *IWB45792* | 149.7 | ***** | *58358, 73553, 74409, IWA1196, 57136, 73554, IWA7225, 58359, 72037* |  |
| 689 | 3B | *IWB58033* | 150.5 | ***** | *IWA773* |  |
| 690 | 3B | *IWB57562* | 154.1 | ***** | *11501* |  |
| 691 | 3B | *IWB12466* | 155.5 | **** | *IWA3601, IWA4457, 6101, 58890, IWA3402* |  |
| 692 | 3B | *IWB7145* | 155.8 | **** | *51803, 74795* |  |
| 693 | 3B | *IWB25495* | 156.3 | **** |  |  |
| 694 | 3B | *IWB11838* | 158.3 | *** | *34730, 25787, 69866, 7838, 65507* |  |
| 695 | 3B | *IWB8515* | 158.6 | ** |  |  |
| 696 | 3B | *IWB50187* | 159.1 | *** |  |  |
| 697 | 3B | *IWB63008* | 159.9 | ** | *39488, 46503, 8984, IWA81, 11298, IWA5209, 65948, 65726* |  |
| 698 | 3B | *IWB35427* | 160.5 | ** |  |  |
| 699 | 3B | *IWA8287* | 160.8 | ** | *9522, 9529, 35497, 12430, 16140, 59356* |  |
| 700 | 3B | *IWB5635* | 161.3 | * | *5637, 29288* |  |
| 701 | 3B | *IWB27956* | 162.1 | ** |  |  |
| 702 | 3B | *IWA3669* | 162.4 | ** | *5030, 18842, 74683, 12192, 63106, 68946, 34833* |  |
| 703 | 3B | *IWB45133* | 162.6 | ** | *25336* |  |
| 704 | 3B | *IWB64869* | 165.4 | * |  |  |
| 705 | 3B | *IWB39561* | 165.7 | * |  |  |
| 706 | 3B | *IWB57606* | 166.0 | * |  |  |
| 707 | 3B | *IWB60995* | 166.5 | * |  |  |
| 708 | 3B | *IWB30796* | 167.1 | ns |  |  |
| 709 | 3B | *IWB57820* | 167.3 | ns | *IWA3332, 35213, IWA8053, 60235, 60236, IWA8054, 52444* |  |
| 710 | 3B | *IWB67102* | 167.6 | ns | *73060, 73061, 25321* |  |
| 711 | 3B | *Xgwm108* | 167.9 | * |  |  |
| 712 | 3B | *IWB65244* | 168.9 | ns | *65245* |  |
| 713 | 3B | *IWB8243* | 169.7 | ns | *3832, 73140, IWA8354, 51903, 62764, IWA6057, 72472, 71427, 72473* |  |
| 714 | 3B | *IWB27126* | 172.3 | ns | *8291, 9005* |  |
| 715 | 3B | *IWB31149* | 172.5 | ns |  |  |
| 716 | 3B | *IWB63048* | 174.8 | ns |  |  |
| 717 | 3B | *IWB59671* | 175.3 | ns | *71096, 71098, 71097, 73555, 11997, 7789* |  |
| 718 | 3B | *IWB58482* | 189.5 | ns |  |  |
| 719 | 3B | *IWB6845* | 195.8 | ns |  |  |
| 720 | 3B | *IWB10030* | 196.0 | ns |  |  |
| 721 | 3B | *IWB31790* | 206.9 | * | *9502, 20636* |  |
| 722 | 3B | *Xwmc326* | 213.0 | ns |  |  |
| 723 | 3B | *IWB12975* | 213.3 | ns | *IWA785, 35557, 14720, 63997, IWA787, IWA786, 48304, IWA3454, 8700, IWA784* |  |
| 724 | 3B | *IWB9228* | 215.6 | ns | *34424, 10390* |  |
| 725 | 3B | *IWB26933* | 221.7 | ns |  |  |
| 726 | 3B | *IWB59521* | 222.0 | ns |  |  |
| 727 | 3B | *IWB10755* | 222.3 | ns |  |  |
| 728 | 3B | *IWB2741* | 224.3 | ns |  |  |
| 729 | 3B | *IWB73987* | 228.8 | ns | *72945, 72294* |  |
| 730 | 3B | *IWB15780* | 229.7 | ns |  |  |
| 731 | 3B | *IWB8511.1* | 229.9 | ns |  |  |
| 732 | 3B | *IWB67339* | 230.5 | ns |  |  |
| 733 | 3B | *IWB62853* | 230.8 | ns |  |  |
| 734 | 3B | *IWB6446* | 231.1 | ns | *2932* |  |
| 735 | 3B | *IWB44760.1* | 232.2 | ns |  |  |
| 736 | 3B | *IWB27815* | 233.0 | ns | *44195, 48528, 66222* |  |
| 737 | 3B | *IWB51875* | 235.9 | ns |  |  |
| 738 | 3B | *IWB44970* | 236.5 | ns | *28240, 45539, 48183, 9387, 9386, 10672, 29374, 67268, 32795, 28241, 44969, 45212, 36533* |  |
| 739 | 3B | *IWB46869* | 239.8 | ns | *10839* |  |
| 740 | 3B | *IWB3434* | 240.3 | ns | *66535, 19046* |  |
| 741 | 3B | *IWB50423* | 242.7 | ns |  |  |
| 742 | 3B | *IWB11701* | 244.1 | ns |  |  |
| 743 | 3B | *Xgwm181* | 244.7 | ns |  |  |
| 744 | 3B | *IWB7537* | 245.3 | ns | *10304, 10530, 74099, IWA8203, 9011, 10667, 34409, 65776, 74101, 35514, 44061, 5917, 10528, 20447, 28522, 45892, 58481, 64375.1, 8058, 4890.1* |  |
| 745 | 3B | *IWB50139* | 245.9 | ns |  |  |
| 746 | 3B | *IWB33098* | 248.0 | ns |  |  |
| 747 | 4A | *IWB9651* | 0.0 | ns |  |  |
| 748 | 4A | *IWA54* | 0.3 | ns | *IWA8389* |  |
| 749 | 4A | *IWB21310* | 5.2 | ns | *21402, 40004, 52625, 21309, IWA3993, 72314* |  |
| 750 | 4A | *IWB8056* | 5.7 | ns |  |  |
| 751 | 4A | *IWB67355* | 8.3 | ns | *26155* |  |
| 752 | 4A | *IWA4232* | 16.4 | ns |  |  |
| 753 | 4A | *IWB47589* | 18.4 | ns |  |  |
| 754 | 4A | *IWA603* | 18.7 | ns |  |  |
| 755 | 4A | *IWB71863* | 19.0 | ns | *IWA5363, 3922, 5591, 71865, 34759* |  |
| 756 | 4A | *IWB4310* | 19.8 | ns | *4316, IWA4260, IWA4261, 6362, 47864* |  |
| 757 | 4A | *IWB52170* | 20.1 | ns | *5953, 39302* |  |
| 758 | 4A | *IWB32882* | 20.9 | ns |  |  |
| 759 | 4A | *IWB5392* | 28.1 | ** | *IWA115, 35812, 37681, 62767, 2614, IWA172, 35055, 14818, 25927, IWA3326, IWA109, IWA5652, 65690, IWA110, 50522, IWA3565* |  |
| 760 | 4A | *IWA7522* | 28.4 | ** | *45447, IWA3582, 35538, 74553, IWA5498, 32815, 32816, IWA5729, 56922, IWA3581* |  |
| 761 | 4A | *IWB47937* | 29.5 | ** |  |  |
| 762 | 4A | *IWB62589* | 30.1 | *** | *25952, IWA232, 75118, 64521, 70360, IWA126, IWA8416* |  |
| 763 | 4A | *IWB3495* | 30.4 | ** | *72073* |  |
| 764 | 4A | *IWB21081.1* | 32.1 | * |  |  |
| 765 | 4A | *IWA3302* | 32.9 | ** | *IWA4359, IWA4253, 51756, IWA4254, IWA4768, 34384, 58994, 31312, IWA4921, IWA7107* |  |
| 766 | 4A | *IWB18200* | 33.8 | * |  |  |
| 767 | 4A | *IWA4698* | 36.3 | ns | *IWA4867, 7960, IWA7448, 31143* |  |
| 768 | 4A | *IWB6710* | 36.6 | ns | *IWA402* |  |
| 769 | 4A | *IWB5380* | 36.9 | * | *5379* |  |
| 770 | 4A | *IWB73430* | 38.3 | ns | *71275, 73431, 71278, 71279* |  |
| 771 | 4A | *IWB48193* | 38.6 | ns |  |  |
| 772 | 4A | *IWA568* | 39.2 | ns | *IWA4431 IWA4657 IWA483* |  |
| 773 | 4A | *IWA4785* | 39.7 | ns | *3706, IWA4512, IWA4787.1, IWA4786, 36333, 3123, 21367, IWA4784, IWA8269.1, 3142, 6208, 28352, 3705* |  |
| 774 | 4A | *IWB10618* | 40.6 | ns | *6880* |  |
| 775 | 4A | *IWB65999* | 43.0 | ns | *59982, 35567, 58950, IWA7077* |  |
| 776 | 4A | *IWB6369* | 43.5 | ns | *IWA6193, 63874, 6533, 44533* |  |
| 777 | 4A | *IWA4199* | 49.9 | ns | *58408* |  |
| 778 | 4A | *IWB32858* | 50.2 | ns | *58650, 5911, 20842, 48433, 34445, 20843* |  |
| 779 | 4A | *IWB63835.1* | 53.0 | ns | *9799* |  |
| 780 | 4A | *IWB69809* | 53.3 | ns | *11902, 40107.1, 7283* |  |
| 781 | 4A | *IWA7058* | 58.7 | ns |  |  |
| 782 | 4A | *IWB36596* | 59.0 | ns |  |  |
| 783 | 4A | *IWB18775.1* | 61.6 | * |  |  |
| 784 | 4A | *IWB68318* | 62.4 | ns | *34721, 29177, 26115, 34669, 7798* |  |
| 785 | 4A | *IWA7118* | 65.4 | ns |  |  |
| 786 | 4A | *IWB26773* | 66.5 | ns | *74450.1* |  |
| 787 | 4A | *IWB10788.1* | 67.1 | ns | *72724, 2776, 25931* |  |
| 788 | 4A | *IWB32864* | 67.6 | ns |  |  |
| 789 | 4A | *IWB64452* | 67.9 | ns | *35289, 15484, 5865* |  |
| 790 | 4A | *IWA6035* | 69.3 | ns | *IWA4425* |  |
| 791 | 4A | *IWB12388* | 72.3 | * |  |  |
| 792 | 4A | *IWB20951* | 74.9 | ns | *47072, 46985* |  |
| 793 | 4A | *IWB68972* | 76.0 | ns |  |  |
| 794 | 4A | *IWB27644* | 79.1 | ns | *59205 ,74494, IWA7066.1, 44327, 52747.1, 70060, 44077.1, 27645, 27643 60773* |  |
| 795 | 4A | *IWA3774* | 80.9 | ns |  |  |
| 796 | 4A | *IWB2658* | 81.7 | * | *28717* |  |
| 797 | 4A | *IWA485* | 84.6 | * |  |  |
| 798 | 4A | *IWB8782* | 85.7 | ns |  |  |
| 799 | 4A | *IWB27701* | 93.4 | * | *60582, 51902, 28330, 48305, 48306, 27429, 73262, 60503, IWA811, 44902, 60583* |  |
| 800 | 4A | *IWB65650* | 93.7 | ** | *31476, 57074* |  |
| 801 | 4A | *IWA4527* | 94.0 | ns | *66725, IWA3698, 26405, 3509, 74680, 46033, 35078* |  |
| 802 | 4A | *IWB34056* | 95.8 | ns | *11890, 18371, 27365, 27364, 8697, 34057, 61041, 28552, 9927, 68839, 34058, 65979, 11891, 10035* |  |
| 803 | 4A | *IWB73029* | 99.1 | ns | *57119* |  |
| 804 | 4A | *IWB34374* | 99.9 | * |  |  |
| 805 | 4A | *IWB28114.1* | 106.3 | ns |  |  |
| 806 | 4A | *IWB44927* | 106.6 | ns | *65221, 66033, 65900* |  |
| 807 | 4A | *IWB6821* | 109.2 | ns |  |  |
| 808 | 4A | *IWB8059* | 109.8 | ns | *60459, 19112, 7057, 44391* |  |
| 809 | 4A | *IWA559* | 111.5 | ns |  |  |
| 810 | 4A | *Xwmc232* | 113.4 | ns |  |  |
| 811 | 4A | *IWB36257* | 116.7 | ns | *11041, 69901, 6276, 28894, 45076, 73461, 7327, 73459, 73460* |  |
| 812 | 4A | *IWB52321* | 117.6 | ns | *46927, 52323, 10131* |  |
| 813 | 4A | *IWB73853* | 117.9 | ns | *8487, 60429, 73854, 10456* |  |
| 814 | 4A | *IWB68597* | 119.1 | ns |  |  |
| 815 | 4A | *IWB71467* | 119.3 | ns |  |  |
| 816 | 4A | *IWB9701* | 120.2 | ns |  |  |
| 817 | 4A | *IWB71844* | 122.3 | ns |  |  |
| 818 | 4A | *IWB36617* | 123.4 | ns | *64352, 73328* |  |
| 819 | 4A | *IWB25556* | 123.7 | ns | *64511, 64509, 11654.1, 73322, 64508, 73321, 64510, 73320, 73329, 34547, 64513, 73323, 73324, 8406, 8408, 7445, 58337, 37657, 11799, 10781, 66546, 64507, 10488, 71843, 8407, 16921, 11714, 73845, 73842, 70641, 6439, 32051, 73843.1, 4506.1, 59715, 11715, 73844, 11713, 5706, 4507, 9122* |  |
| 820 | 4A | *IWB2554* | 126.0 | ns |  |  |
| 821 | 4A | *IWB14801* | 126.0 | ns | *34028, 25400, 60304, 34029, 68682* |  |
| 822 | 4A | *Xbarc78* | 126.0 | ns |  |  |
| 823 | 4A | *IWB70620* | 126.3 | ns | *63966, 6832, 9431, 66663, 68352, 63757, 13152, 57765, 7370, 73505, 59018, 5798* |  |
| 824 | 4A | *IWB18869.1* | 126.6 | ns |  |  |
| 825 | 4A | *IWB3267* | 127.5 | ns | *16109, 73660, 69029, 69030, 48471, 12490, 62597, 3268, 12459, 10945* |  |
| 826 | 4A | *IWB3265* | 129.9 | ns |  |  |
| 827 | 4A | *IWB34579* | 132.3 | ns |  |  |
| 828 | 4A | *IWB57683* | 132.9 | ns | *63976, 27971, 47401, 63975, 10519, 52207, 63979, 34733, 3571, 26020, 3570, IWA7305, 3572, 3569* |  |
| 829 | 4A | *IWB8842* | 135.3 | ns |  |  |
| 830 | 4A | *IWB52795* | 138.3 | ns |  |  |
| 831 | 4A | *IWB8552* | 138.8 | ns | *74014, 36777, 58087, 8778* |  |
| 832 | 4A | *IWB39717* | 139.1 | ns | *71808, 39716, 71809, 60692, 12593, 5608, 71810* |  |
| 833 | 4A | *IWB65913* | 139.4 | ns | *73721, 70769, 45417, 69888.1, 73203, 71700, 71978, 71764, 58574, 14910, 71319, IWA178.1, 57367, 71699, 59774.1, 65772, 21471, 26822, 11466, 57392, 60881, 34249, 34226, 59345, 71765, 31447, 31040, 71768, 27194, 12146, 71979, 31039, 68322, 34224, 27358, 6928, 31512, 34246, 58396, 8062, 69889.1, 71767, 34594, 4517, 26246, 66436, 71697, 73723, IWA4946, 4478, 71698, 59346* |  |
| 834 | 4A | *IWB64680* | 146.3 | ns |  |  |
| 835 | 4A | *IWB18740* | 146.6 | ns | *68148, 59099, 57528, 19424, 25415* |  |
| 836 | 4A | *IWB26452* | 146.9 | ns |  |  |
| 837 | 4A | *IWB39523* | 148.6 | ns |  |  |
| 838 | 4B | *IWB70791* | 0.0 | ns | *70795, 74960, 70793, 70794, 74226, 74227, 70790* |  |
| 839 | 4B | *IWB8794* | 0.6 | ns |  |  |
| 840 | 4B | *IWB71989* | 0.9 | ns |  |  |
| 841 | 4B | *IWB73905* | 4.3 | ns |  |  |
| 842 | 4B | *IWA8107* | 6.6 | ns | *32911* |  |
| 843 | 4B | *IWA8108* | 7.1 | ns |  |  |
| 844 | 4B | *IWB63893* | 8.9 | ns | *63894, 71830* |  |
| 845 | 4B | *IWB35920* | 9.2 | ns |  |  |
| 846 | 4B | *IWB25162* | 9.2 | ns |  |  |
| 847 | 4B | *IWB31302* | 9.5 | ns |  |  |
| 848 | 4B | *IWA8178* | 9.8 | ns | *62755, 28772, 39916, 28771, 73384* |  |
| 849 | 4B | *IWB73117* | 13.5 | ns | *73118, 2333* |  |
| 850 | 4B | *IWB72527* | 15.8 | ns | *44350, 69708, 30977, 46249* |  |
| 851 | 4B | *IWB59992* | 19.1 | ns |  |  |
| 852 | 4B | *IWB59993* | 19.9 | ns |  |  |
| 853 | 4B | *IWB73302* | 23.2 | ns |  |  |
| 854 | 4B | *IWB72764* | 23.4 | ns | *72765, 69587, 73835* |  |
| 855 | 4B | *IWB66414* | 23.7 | ns | *72203, 66413* |  |
| 856 | 4B | *IWB35969* | 24.0 | ns | *58052, 33162, 12150, 7508, 12149* |  |
| 857 | 4B | *IWB69815* | 24.9 | ns | *66138, IWA566, 14853, 44602* |  |
| 858 | 4B | *IWB73258* | 25.1 | ns | *71276, 71281, 65937, 72949, 73919, 4719,71024, 70449, 73563, 11340, 47162, 7255, IWA4916, 70450, 73564, 66679, 71022.1, 71280, 70187, 4721, 73918, 73561, 47988, 5676, 4720* |  |
| 859 | 4B | *IWB73832* | 25.4 | ns | *73831, 6877, 12982, 7934, 73830* |  |
| 860 | 4B | *IWB16360* | 26.0 | ns |  |  |
| 861 | 4B | *IWB11928* | 26.9 | ns |  |  |
| 862 | 4B | *IWB9610* | 28.3 | ns | *72801* |  |
| 863 | 4B | *IWB73411* | 28.9 | ns | *73589, 73588, 71634, 72936, 71407* |  |
| 864 | 4B | *IWB47531* | 29.5 | ns |  |  |
| 865 | 4B | *IWB67487* | 30.3 | ns | *73915, 67483, 67485, 72792, 67486* |  |
| 866 | 4B | *IWB68116* | 30.6 | ns | *6658, 35533, 17788, 65865, 30893, 73026, 46581.1, 73027, 66308, 72187* |  |
| 867 | 4B | *IWB71418* | 30.9 | ns | *67854, 33116, 67738, 57527, 33115, 73701, 28112.1, 69165, 73300, 10265, 10821, 70250, 74042, 45275, 9492, 73207, 72884, 74043* |  |
| 868 | 4B | *IWB71386.1* | 32.7 | ns |  |  |
| 869 | 4B | *Xbarc1133* | 33.6 | ns |  |  |
| 870 | 4B | *IWA8564* | 35.8 | ns |  |  |
| 871 | 4B | *IWB36089* | 37.5 | ns | *10740, 11611, 58174* |  |
| 872 | 4B | *IWB25268* | 38.7 | ns |  |  |
| 873 | 4B | *IWB31724* | 40.1 | ns | *69182, 69236, 67330, 59727, 34149, 70047, 33140, 35802, 69559, 69560, 58216, 47287, 70063, 45500, 57889, 68468, 35870, 47113, 32941, 68259, 69181, 31053, 69705, 61136, 69938, 65875, 58977, 44029, 71186, 45221, 50249, 56796, 43967, 27331, 26492, 3197, 68348, 12519.1, 44467, 73930, 72225, 47430, 5508, 46169, 70484, 36179, 72265, 72288, 33131, 66082, 58952, 45794, 72287, 58933, 63293* |  |
| 874 | 4B | *IWB36179* | 41.0 | ns | *72265, 72288, 33131, 66082, 58952, 45794, 72287, 58933, 63293* |  |
| 875 | 4B | *Xgwm540* | 41.3 | ns |  |  |
| 876 | 4B | *IWB17848* | 43.4 | ns | *9392, 21302* |  |
| 877 | 4B | *IWB35513* | 44.0 | ns | *61000, 15003* |  |
| 878 | 4B | *IWB35380* | 45.9 | ns | *33082, 34629, 74037, 28989, 34873, 47207, IWA5679, 35352, 3636* |  |
| 879 | 4B | *IWB72399* | 47.1 | ns |  |  |
| 880 | 4B | *IWB70599* | 52.2 | ns | *73146* |  |
| 881 | 4B | *IWB44068* | 59.2 | ns |  |  |
| 882 | 4B | *IWB9672.1* | 60.7 | ns |  |  |
| 883 | 4B | *IWB7195* | 61.2 | ns |  |  |
| 884 | 4B | *IWB28344* | 62.6 | ns | *64316* |  |
| 885 | 4B | *IWB34520* | 64.6 | * | *36336, 7850, 9697, 27710, IWA3846, 7849, 20947, 6952* |  |
| 886 | 4B | *IWB39624* | 67.4 | * |  |  |
| 887 | 4B | *IWB73144* | 69.5 | * | *9230, 73143, 6948, 73462* |  |
| 888 | 4B | *IWB69664* | 70.3 | ns | *69695, 71669* |  |
| 889 | 4B | *IWA3396* | 70.6 | ns | *70299, 72778, 61304* |  |
| 890 | 4B | *IWB70855* | 70.9 | ns | *70853, 60841, 73855, 70856, 70858* |  |
| 891 | 4B | *IWB32927* | 71.4 | ns | *IWA4640, IWA3609, IWA3608, IWA3611, 71858, 71859, 60954* |  |
| 892 | 4B | *IWB73466* | 72.2 | ns | *73473* |  |
| 893 | 4B | *IWB17082* | 72.8 | ns |  |  |
| 894 | 4B | *IWB44538* | 75.2 | ** |  |  |
| 895 | 4B | *IWB28273* | 75.5 | ** | *12276, 8168* |  |
| 896 | 4B | *IWB12434* | 75.5 | ** |  |  |
| 897 | 4B | *IWB71804* | 75.7 | ** | *68421* |  |
| 898 | 4B | *IWB66294* | 76.3 | ** | *69711, 35851, 32903, 71402, 47175* |  |
| 899 | 4B | *IWB7973* | 78.3 | ** | *3117, 25056, 73999* |  |
| 900 | 4B | *IWB45462* | 78.3 | * |  |  |
| 901 | 4B | *IWB48267* | 78.6 | * | *65375* |  |
| 902 | 4B | *IWB66538* | 78.9 | * | *73113, 7278, 72310, 36159, 66539, 47105* |  |
| 903 | 4B | *IWB60004* | 79.2 | * | *70938, 71666, 71667* |  |
| 904 | 4B | *IWB44585* | 79.4 | * |  |  |
| 905 | 4B | *IWB73383* | 80.3 | * | *73629, 17020, 73630* |  |
| 906 | 4B | *IWB71553* | 82.7 | * |  |  |
| 907 | 4B | *IWB44750* | 83.2 | *** | *56768, 66095* |  |
| 908 | 4B | *IWB7389* | 83.8 | *** | *44155, 39351* |  |
| 909 | 4B | *IWB47537* | 84.3 | *** | *6506, 72121, 72120, 71883, 32976, 45303* |  |
| 910 | 4B | *IWB75280* | 87.0 | ** |  |  |
| 911 | 4B | *IWB7462* | 89.6 | *** |  |  |
| 912 | 4B | *IWB60053* | 89.6 | *** |  |  |
| 913 | 4B | *IWB20754* | 89.9 | *** |  |  |
| 914 | 4B | *IWB11229* | 91.0 | *** | *72883, 29648, 34604, 6828.1, 29649, 4426* |  |
| 915 | 4B | *IWB75098* | 98.9 | * | *5989* |  |
| 916 | 4B | *IWB74054* | 99.1 | ns | *11859, 58739, 72184, IWA5408, 7266, 35505* |  |
| 917 | 4B | *Xwmc125* | 100.1 | ns |  |  |
| 918 | 4B | *IWB48353* | 100.7 | ns |  |  |
| 919 | 4B | *IWB3641* | 103.6 | ns | *8229, 57254, IWA5358, 50138* |  |
| 920 | 4B | *IWB74794* | 104.1 | ns | *4448 ,7044, 4447, 12222, IWA3781, 3246, IWA564, 17522* |  |
| 921 | 4B | *IWA27* | 105.6 | ns | *35335* |  |
| 922 | 4B | *IWB6896* | 109.6 | ns | *73485, 12144, 73486* |  |
| 923 | 4B | *IWB35942.1* | 123.5 | ns | *3256, 5799, 6596, 37519, 6635, 9483* |  |
| 924 | 4B | *IWB67499* | 125.2 | ns |  |  |
| 925 | 5A | *IWB70789* | 0.0 | ns |  |  |
| 926 | 5A | *IWB71134* | 0.3 | ns | *71133, 71135, 25755* |  |
| 927 | 5A | *IWB64323* | 1.1 | ns | *IWA4445* |  |
| 928 | 5A | *IWA3567* | 1.4 | ns | *IWA3566* |  |
| 929 | 5A | *IWB64781* | 3.4 | ns |  |  |
| 930 | 5A | *IWA7361* | 15.5 | ns | *IWA7360 ,7817* |  |
| 931 | 5A | *IWB74361.1* | 17.9 | ns | *50392, IWA8268, IWA6226.1, 11440, 5245, 4171, IWA6227, 29632* |  |
| 932 | 5A | *IWB29633* | 18.8 | ns |  |  |
| 933 | 5A | *IWB64310* | 20.0 | ns |  |  |
| 934 | 5A | *IWB11068* | 21.8 | ns | *IWA4970* |  |
| 935 | 5A | *IWA5368* | 25.3 | ns | *35357* |  |
| 936 | 5A | *IWB12627* | 28.5 | ns |  |  |
| 937 | 5A | *IWB72119* | 28.8 | ns |  |  |
| 938 | 5A | *IWB48152* | 29.1 | ns | *48151, 40035, 12085, IWA5615, IWA4069, IWA3811, IWA3365* |  |
| 939 | 5A | *IWB6728* | 31.8 | ns |  |  |
| 940 | 5A | *IWB68312* | 33.6 | ns | *9025* |  |
| 941 | 5A | *IWB3091* | 35.0 | ns |  |  |
| 942 | 5A | *IWB66553* | 37.9 | ns |  |  |
| 943 | 5A | *IWA8155* | 38.2 | ns | *IWA6287, 7826, IWA8154* |  |
| 944 | 5A | *IWB3903* | 39.3 | ns |  |  |
| 945 | 5A | *Xbarc186* | 41.3 | ** |  |  |
| 946 | 5A | *IWA5395* | 42.3 | ns | *47210* |  |
| 947 | 5A | *IWB48524* | 44.1 | ns | *35024, IWA5295* |  |
| 948 | 5A | *IWB31546* | 44.7 | ns | *17850 ,73385, IWA3349* |  |
| 949 | 5A | *IWB33251* | 44.9 | ns | *49889, 46277* |  |
| 950 | 5A | *IWA8582* | 46.7 | ns |  |  |
| 951 | 5A | *IWA3975* | 50.5 | ns | *65837, 9723, IWA5539, IWA5538, 65885, 74436, 45248, 9139, 9138* |  |
| 952 | 5A | *IWB66579* | 51.1 | ns | *27439, 51874, 7967, 68868, 12830, 11474, 10513, 44194* |  |
| 953 | 5A | *IWB61122* | 53.5 | ns | *47676, 34727, 35587* |  |
| 954 | 5A | *IWB71919* | 55.5 | ns |  |  |
| 955 | 5A | *IWB66385* | 55.8 | ns |  |  |
| 956 | 5A | *IWB12396* | 56.9 | ns |  |  |
| 957 | 5A | *IWB34674* | 57.5 | ns |  |  |
| 958 | 5A | *IWB33272* | 58.0 | ns |  |  |
| 959 | 5A | *IWB21005* | 58.3 | ns |  |  |
| 960 | 5A | *IWA6036* | 65.7 | ns | *33227, IWA850, 71455, 73141 52403, 60328, 26857, IWA8012, 72698, IWA8013, 69498, 64699, 35711, IWA5326, 12120, 63769, 11110, 71628, 71630, 49769, 7558, 2714, 11700, 11586, 71627, 2713, 10451, 73545, 27684, 71631, IWA5327, 46440, 60329, 36666, 10452 ,31223, IWA5330, 69450, 19015, 5955, 51790, 12121, 6585, 7040, 46074* |  |
| 961 | 5A | *IWB37510* | 66.0 | ns |  |  |
| 962 | 5A | *Xgwm186* | 66.6 | ns |  |  |
| 963 | 5A | *IWB10677* | 66.6 | ns | *46680* |  |
| 964 | 5A | *IWB63767* | 67.7 | ns |  |  |
| 965 | 5A | *IWB48382* | 68.0 | ns | *9533, IWA6126, 33393, 7271, 74982, 46350 ,60075, 52429, 20824, IWA4050, 36569, 36130, 5443, 75149, 3965, 4699, 59045, 3199, 69484, 39228, 6622, 75151, 5479, 44586* |  |
| 966 | 5A | *IWB26864* | 73.8 | ns | *5849, 33444, IWA4667, IWA3313, 12115, IWA4669, 34540, 12116* |  |
| 967 | 5A | *IWB10384* | 74.1 | ns | *36264, 36245, 6074* |  |
| 968 | 5A | *IWB52863* | 74.4 | ns | *52864* |  |
| 969 | 5A | *IWB73963* | 77.0 | ns |  |  |
| 970 | 5A | *IWB44434* | 79.3 | ns | *33346, IWA6949, 11865, 35355, 33345* |  |
| 971 | 5A | *IWB34371* | 81.1 | ns |  |  |
| 972 | 5A | *IWB17918* | 83.7 | ns | *35000, 9118, 8807, 65371, 39532, 66091, 50640 ,75097, 58598, 3848* |  |
| 973 | 5A | *IWB50162* | 84.9 | ns | *IWA7529, IWA4299* |  |
| 974 | 5A | *IWA8588* | 88.5 | * | *IWA7135* |  |
| 975 | 5A | *IWB29626* | 89.1 | * |  |  |
| 976 | 5A | *IWB61032* | 90.9 | ** | *6762, 73631* |  |
| 977 | 5A | *Xwmc415* | 90.9 | * |  |  |
| 978 | 5A | *IWB75269* | 93.7 | ns | *72978, 72977* |  |
| 979 | 5A | *IWB26027* | 94.0 | ns | *6273* |  |
| 980 | 5A | *IWB46709.1* | 94.3 | ns |  |  |
| 981 | 5A | *IWB47646* | 94.8 | ns | *33329, 36671, 10250* |  |
| 982 | 5A | *IWB34570.1* | 103.4 | ns |  |  |
| 983 | 5A | *IWB3340* | 103.9 | ns |  |  |
| 984 | 5A | *IWB27298* | 104.5 | ns | *6702, 26710, 6708, 12467* |  |
| 985 | 5A | *IWB9503* | 107.6 | ns |  |  |
| 986 | 5A | *IWA5668* | 107.9 | ns | *IWA3996, IWA12* |  |
| 987 | 5A | *IWB63299* | 108.1 | ns | *72387, 72386* |  |
| 988 | 5A | *IWB10966* | 108.4 | ns | *10965, 6184* |  |
| 989 | 5A | *IWB59600* | 108.7 | ns | *62557, 35938, 9988* |  |
| 990 | 5A | *IWB72998* | 109.0 | ns | *71983, 71984, 73294, 69159, 73295* |  |
| 991 | 5A | *IWB71642* | 109.3 | ns |  |  |
| 992 | 5A | *IWB59852* | 109.3 | ns | *50059, 71643, 59853, 71641* |  |
| 993 | 5A | *IWB8905* | 110.7 | ns |  |  |
| 994 | 5A | *IWA4205* | 117.6 | ** | *66355* |  |
| 995 | 5A | *IWA5624* | 119.4 | ** | *IWA5623, 52187, 10766, 7746, 26647, 73200, 70567* |  |
| 996 | 5A | *IWB73552* | 120.2 | * |  |  |
| 997 | 5A | *IWB6049* | 123.2 | * | *72992, 73058, 66717* |  |
| 998 | 5A | *IWB58257* | 126.1 | ** |  |  |
| 999 | 5A | *IWB33488* | 127.2 | ** | *72283, 72327* |  |
| 1000 | 5A | *IWB73502* | 128.1 | *** | *36340, 72425, 56489* |  |
| 1001 | 5A | *IWA7256* | 130.1 | ** | *IWA7255, 11585* |  |
| 1002 | 5A | *IWB9808* | 131.0 | ** |  |  |
| 1003 | 5A | *IWB59813* | 132.4 | ns |  |  |
| 1004 | 5A | *IWB8258* | 133.8 | * |  |  |
| 1005 | 5A | *IWB27510* | 134.1 | ** | *26265* |  |
| 1006 | 5A | *IWA4805* | 134.6 | * |  |  |
| 1007 | 5A | *IWB52008* | 135.5 | * |  |  |
| 1008 | 5A | *IWB27060.1* | 136.3 | ns |  |  |
| 1009 | 5A | *IWB72363* | 137.7 | * | *72362, 72361* |  |
| 1010 | 5A | *IWA576* | 140.9 | ** |  |  |
| 1011 | 5A | *IWB44564* | 145.3 | * |  |  |
| 1012 | 5A | *IWB8292* | 146.2 | * |  |  |
| 1013 | 5A | *IWB5457* | 147.0 | ns |  |  |
| 1014 | 5A | *IWB35422* | 147.3 | ns |  |  |
| 1015 | 5A | *IWA582* | 147.8 | ns |  |  |
| 1016 | 5A | *IWB72888* | 157.5 | ns |  |  |
| 1017 | 5A | *IWB73761* | 157.8 | ns |  |  |
| 1018 | 5A | *IWB7685* | 174.3 | ns |  |  |
| 1019 | 5A | *IWB33102.1* | 174.5 | ns |  |  |
| 1020 | 5A | *IWB61152* | 179.7 | ns |  |  |
| 1021 | 5A | *IWB3232* | 181.1 | ns | *67141 ,8349, 66080, 65128, 11676, 10909, 28898* |  |
| 1022 | 5A | *IWB69883* | 182.6 | ns |  |  |
| 1023 | 5A | *IWB69884* | 184.4 | ns |  |  |
| 1024 | 5A | *IWB29171* | 185.6 | ns |  |  |
| 1025 | 5A | *IWB9130* | 187.3 | ns |  |  |
| 1026 | 5A | *IWA7162* | 188.7 | ns |  |  |
| 1027 | 5A | *IWB71656* | 188.9 | ns |  |  |
| 1028 | 5A | *IWB14661* | 189.2 | ns | *45943, 6827, 5368* |  |
| 1029 | 5A | *IWB7282* | 189.5 | ns |  |  |
| 1030 | 5A | *IWB14680* | 190.6 | ns | *4543, 71385, 4836* |  |
| 1031 | 5A | *IWB34800* | 191.1 | ns |  |  |
| 1032 | 5A | *IWB18172* | 191.4 | ns |  |  |
| 1033 | 5A | *IWB14077* | 196.2 | ns | *9855* |  |
| 1034 | 5A | *IWB6716* | 197.7 | ns |  |  |
| 1035 | 5A | *IWB72152* | 197.9 | ns | *72151* |  |
| 1036 | 5A | *IWB60850* | 198.5 | ns | *73574, 60644* |  |
| 1037 | 5A | *IWB19304* | 198.8 | ns |  |  |
| 1038 | 5A | *IWB9800* | 202.2 | ns | *6768* |  |
| 1039 | 5A | *IWB3188* | 202.2 | ns |  |  |
| 1040 | 5B | *XCfb306* | 0.0 | *** |  |  |
| 1041 | 5B | *IWB47425* | 1.7 | **** | *3560, 33430, 46248* |  |
| 1042 | 5B | *IWB11710* | 2.3 | **** | *IWA3436* |  |
| 1043 | 5B | *IWB64829* | 5.9 | **** | *IWA5486 ,65483, IWA7507, IWA8343, 28346, 44555, IWA5485, IWA5621* |  |
| 1044 | 5B | *IWB11140* | 7.0 | **** | *3561* |  |
| 1045 | 5B | *IWB73718* | 7.3 | **** | *34844, IWA5331* |  |
| 1046 | 5B | *IWB64286* | 7.5 | ***** | *56529, 34553* |  |
| 1047 | 5B | *IWB37583* | 8.4 | ***** | *72268* |  |
| 1048 | 5B | *IWB73786* | 14.0 | *** | *33287* |  |
| 1049 | 5B | *IWB33289* | 14.3 | **** |  |  |
| 1050 | 5B | *IWB26051* | 18.6 | *** | *46856, IWA3514* |  |
| 1051 | 5B | *IWB4841* | 20.3 | ** | *4020, 8869, IWA421, IWA332, 33515, IWA420, 66134* |  |
| 1052 | 5B | *IWB26568* | 20.5 | ** |  |  |
| 1053 | 5B | *IWB25449* | 21.1 | ** |  |  |
| 1054 | 5B | *IWA7181* | 26.9 | ns |  |  |
| 1055 | 5B | *IWB14163* | 27.2 | ns | *14332* |  |
| 1056 | 5B | *IWB21416* | 28.9 | ns | *IWA4856, 58056, 5907* |  |
| 1057 | 5B | *IWB58311* | 29.5 | ns | *7932, IWA6947, 47615, IWA6946* |  |
| 1058 | 5B | *IWB26650* | 30.6 | ns |  |  |
| 1059 | 5B | *IWB57734* | 37.3 | ns |  |  |
| 1060 | 5B | *IWB8792* | 39.1 | ns | *10350* |  |
| 1061 | 5B | *IWB6617* | 48.9 | ns | *8358, 27923, 33409, 34691, 49864, 67262, IWA6987, 3389, 4368, 46762, IWA4400, IWA6967, 10779, 34442, 74739, 29582, 64498, 66057, IWA5281, IWA7307, 34843, 35059, IWA271, IWA4547, IWA5604, 8594, 11231, 35390, IWA4839, IWA5517, 20683, 48001, 44376, 59222, IWA4127, 60973, 34808, 45165, IWA4540, IWA7186, 9030, IWA7096, 36654, 48052, 65246, 65912, IWA7515, 5537, 33490, 8593, IWA7079, 9094, 60145, 20682, IWA7882, IWA5730, 34924, IWA6968, 39139, IWA5519, 39138, 25908, 39140, IWA5412, IWA4060, 45167, IWA5603, IWA4756, 15223* |  |
| 1062 | 5B | *IWB29025* | 49.5 | ns | *58992* |  |
| 1063 | 5B | *IWB6012* | 50.0 | ns | *IWA8604, 33248, 58528, IWA5439 ,3400, 39846, 65840, 47295, IWA5440, 52869, IWA8378, IWA8569, 43890, IWA4016, 21107, 12549, 44326, 66116, 49857, IWA5438* |  |
| 1064 | 5B | *IWB10925* | 50.3 | ns |  |  |
| 1065 | 5B | *IWB8397* | 50.6 | ns | *9536, 64551, IWA396* |  |
| 1066 | 5B | *IWB68299* | 50.8 | ns | *71274* |  |
| 1067 | 5B | *IWB7200* | 51.1 | ns | *35584, IWA3707, 58307, 4516, IWA7272, 26887, 6899, 34781, 44691, IWA3706, 10495, 20936, 20644* |  |
| 1068 | 5B | *IWA4158* | 51.4 | ns | *IWA4414, 70531, 47892, 21305, 26138, IWA8005, 27987, 29478, 33377* |  |
| 1069 | 5B | *IWB39718* | 52.4 | ns | *73671, 65517, 7194, 9881, 33255, 5705, 33437, 35796, 5806, 24957, 27185* |  |
| 1070 | 5B | *IWB36090* | 54.1 | ns | *36619, IWA5280, 64495, 70100, IWA5279, 69519, 74026* |  |
| 1071 | 5B | *Xbarc74* | 55.0 | ns |  |  |
| 1072 | 5B | *IWB11100* | 56.1 | ns | *67284, IWA5289, IWA4422, 71914, 11638, 62611, 71912, 45956, IWA123, 33454* |  |
| 1073 | 5B | *IWB70171* | 56.4 | ns |  |  |
| 1074 | 5B | *Xgwm213* | 56.7 | ns |  |  |
| 1075 | 5B | *IWB64262* | 58.0 | ns | *33231, IWA8603* |  |
| 1076 | 5B | *IWA5742* | 60.9 | ns | *35030* |  |
| 1077 | 5B | *IWB25222* | 61.1 | ns |  |  |
| 1078 | 5B | *IWB36811* | 64.9 | ns | *9324* |  |
| 1079 | 5B | *IWB67658* | 68.0 | ns |  |  |
| 1080 | 5B | *IWB3560* | 69.7 | ns | *33430, 46248, IWA3436, IWA5486, 65483, IWA7507, IWA8343, 28346, 44555, IWA5485, IWA5621, 3561, 34844, IWA5331, 56529, 34553, 72268* |  |
| 1081 | 5B | *IWB65958* | 70.5 | ns |  |  |
| 1082 | 5B | *IWB11879* | 73.6 | ns | *63206, 47848* |  |
| 1083 | 5B | *IWB3607* | 74.4 | ns |  |  |
| 1084 | 5B | *IWB70658* | 75.5 | ns | *65972, 33293, 65268, 70492* |  |
| 1085 | 5B | *IWB5390* | 76.1 | ns |  |  |
| 1086 | 5B | *IWB45894* | 76.3 | ns | *32971, 73106, 57415, 70679.1, 35878, 52780, 65086, IWA3985, IWA4074, 37690, IWA5283, 69455* |  |
| 1087 | 5B | *IWB5713* | 77.4 | ns |  |  |
| 1088 | 5B | *IWB6817* | 77.7 | ns | *57484, 72579, 45663, 66469, 73111, IWA265, 28823, 44183, 65241, 45559, 73110, 57882, 72578, 9767, 45662* |  |
| 1089 | 5B | *IWB46986* | 78.0 | ns | *IWA4222, 13598, 48223, 48132, 9038, IWA4571, 73416, 6346, 8758, 4727, 66311, 39314, 28625, 46563, 6188* |  |
| 1090 | 5B | *IWB33391* | 78.3 | ns | *33316, 66934, 36579, 33390* |  |
| 1091 | 5B | *IWB10247* | 81.5 | ns | *27412* |  |
| 1092 | 5B | *IWB5882* | 81.7 | ns | *29181, IWA4641* |  |
| 1093 | 5B | *IWB12924* | 89.5 | ns | *11179, 35598, 4569, 33263, 52612, IWA5672, IWA8187, 44588* |  |
| 1094 | 5B | *IWB72334* | 89.8 | ns |  |  |
| 1095 | 5B | *IWB20925* | 90.3 | ns | *27613, 7132, 20967, 6302, 10415, 6211, 66391, 10904, 58357, 10903, 64820, 7809, 35898, 58072, 59471, 65583, 8298, 33814, 11306, 6612, 9523, 34284, 16701, 28901, 48375, 34489, 43973, 33476, 65697, 60951, 61075, 26666, 31745, 69283, 20927* |  |
| 1096 | 5B | *IWB6464* | 91.2 | ns |  |  |
| 1097 | 5B | *IWA8252* | 92.0 | ns | *63264, 63265, 63263* |  |
| 1098 | 5B | *IWA5478* | 105.2 | ns | *11817, 30911, 35467, 3593, 25540* |  |
| 1099 | 5B | *IWB34313* | 106.4 | ns | *IWA6905, 33507, 36661, 33509* |  |
| 1100 | 5B | *IWB72095* | 116.9 | ns |  |  |
| 1101 | 5B | *IWB72094* | 117.2 | ns |  |  |
| 1102 | 5B | *IWB48396* | 117.8 | ns | *57214* |  |
| 1103 | 5B | *IWA8097* | 118.1 | ns | *46363* |  |
| 1104 | 5B | *IWB6687* | 118.3 | ns | *56720, 70159, 5758, 11074, IWA6915, 11477, 13523, 66356, 8368, 11522, 27688, 44719, 35873, 33371, 61049, 60182, 12092, 57669, 36772, 27651, 33372* |  |
| 1105 | 5B | *IWB43876* | 119.5 | ns |  |  |
| 1106 | 5B | *IWB10635* | 119.8 | ns | *61245, 71533, 9783* |  |
| 1107 | 5B | *IWB18958* | 121.8 | ns | *10018, 46125* |  |
| 1108 | 5B | *IWB6063* | 122.1 | ns | *6407, 70387, 35127, 6852, 8052, 29205, 34530, 34918, IWA3479, 35621, 36098, 11813, 39412, 70720, IWA6024, 48019, 47298, 34332, 6023, 25936, 52446* |  |
| 1109 | 5B | *IWB56412* | 122.3 | ns |  |  |
| 1110 | 5B | *IWB10135* | 123.2 | ns | *8906* |  |
| 1111 | 5B | *IWB69317* | 123.7 | ns | *75248* |  |
| 1112 | 5B | *IWB6424* | 124.0 | ns | *IWA8395* |  |
| 1113 | 5B | *IWB52474* | 124.3 | ns |  |  |
| 1114 | 5B | *Xwmc75* | 125.2 | ns |  |  |
| 1115 | 5B | *IWB32852* | 126.0 | ns | *21097, 66067, 56628, 11341, 36002, 58682, 35480, 34518, 2787, 34511, 2786* |  |
| 1116 | 5B | *IWB29442* | 126.3 | ns |  |  |
| 1117 | 5B | *IWB7415* | 126.6 | ns | *65012, 31717* |  |
| 1118 | 5B | *IWB27379* | 126.9 | ns |  |  |
| 1119 | 5B | *IWB33063* | 127.7 | ns | *IWA5552, 60268, 46235, 11984, 9459, IWA5551* |  |
| 1120 | 5B | *IWB7206* | 128.0 | ns | *7441* |  |
| 1121 | 5B | *IWB75021* | 128.3 | ns | *33023* |  |
| 1122 | 5B | *IWB67069* | 128.6 | ns |  |  |
| 1123 | 5B | *IWB2976* | 130.0 | ns |  |  |
| 1124 | 5B | *IWB74046* | 131.4 | ns | *74045* |  |
| 1125 | 5B | *IWB6641* | 133.7 | ns | *7213* |  |
| 1126 | 5B | *IWB10535* | 134.6 | ns |  |  |
| 1127 | 5B | *IWB68076* | 134.8 | ns | *7405, 45473* |  |
| 1128 | 5B | *IWA4539* | 135.1 | ns | *7930, 48107, 59550, 36107, 59549, 47857, 21390, 7634, 7633, 34578, 25600, 44945, 7931, 73702, 72281, 8537, IWA7478* |  |
| 1129 | 5B | *IWB29397* | 135.4 | ns | *50044, IWA7872, 36568* |  |
| 1130 | 5B | *IWB71390* | 135.7 | ns |  |  |
| 1131 | 5B | *IWB71849* | 136.3 | ns |  |  |
| 1132 | 5B | *IWB73175* | 136.8 | ns |  |  |
| 1133 | 5B | *Xbarc142* | 137.4 | ns |  |  |
| 1134 | 5B | *IWB69475* | 138.0 | ns | *8035, 64502, 64503, 68202, 29637, 69510, 64501, 28146, 47520* |  |
| 1135 | 5B | *IWB9438* | 138.8 | ns |  |  |
| 1136 | 5B | *IWB72592* | 139.6 | ns | *73678, 73572* |  |
| 1137 | 5B | *IWB63298* | 140.5 | ns |  |  |
| 1138 | 5B | *IWA4185* | 140.7 | ns | *3699, IWA4184, IWA4182, 69025, IWA4183, 8581* |  |
| 1139 | 5B | *IWB72048* | 141.9 | ns | *72049, 34592* |  |
| 1140 | 5B | *IWB12143* | 142.5 | ns | *5765, 50686, 74826, 63422, 12142, IWA3394, 7080, 37513, 7359, 7593, 70782* |  |
| 1141 | 5B | *IWB71831* | 144.5 | ns |  |  |
| 1142 | 5B | *IWB64707* | 144.8 | ns | *35993, 71938, 72667, 7744, 73816, 72681, 73814, 72666, 72503, 71437, 47069, 7105, 72680, 71439, 71438, 73815, 69972* |  |
| 1143 | 5B | *IWB71387* | 145.1 | ns | *75132, 6980, 64600, 73499, 5199* |  |
| 1144 | 5B | *IWB71821* | 154.4 | ns | *71819, 32973, 65531, 71818, 71820* |  |
| 1145 | 5B | *IWB44136* | 154.7 | ns | *33119, 44993, 7672, 12043, 71749, 45090, 50470, 26748, 46501, 10888.1, 34447, IWA3972, 8173, 2823, 46655, 34550, 9382, 30997, 71748, 11032, 61247, 58120, 36006, 73180, 5837* |  |
| 1146 | 5B | *IWB68167* | 155.5 | ns | *64466, 11919* |  |
| 1147 | 5B | *IWB63067* | 156.1 | ns | *9424, 9901.1, 63068, 48112, 34960, 12106, 12107* |  |
| 1148 | 5B | *IWB74041* | 156.7 | ns | *72617, 72615, 72618, 72616* |  |
| 1149 | 5B | *IWB27453* | 156.9 | ns | *26458, 27369, 4834, 63462, 29239, 5599, 4441, 5663* |  |
| 1150 | 5B | *IWB29437* | 158.4 | ns |  |  |
| 1151 | 5B | *IWB26828* | 158.7 | ns | *26827* |  |
| 1152 | 5B | *IWA6211* | 158.9 | * | *IWA4748, 29675, 34210, IWA5454, IWA4635, 7719, 11035, 25892* |  |
| 1153 | 5B | *IWB74035* | 160.0 | ns |  |  |
| 1154 | 5B | *IWA7903* | 160.9 | ns |  |  |
| 1155 | 5B | *IWB12232* | 162.3 | ns | *IWA22, 36204, 34704, 13050, 18951, 9279* |  |
| 1156 | 5B | *IWB26719* | 165.9 | ns |  |  |
| 1157 | 5B | *IWB73021* | 166.1 | ns |  |  |
| 1158 | 5B | *Xbarc243* | 166.4 | ns |  |  |
| 1159 | 5B | *IWB26951* | 166.4 | * |  |  |
| 1160 | 5B | *IWB70634* | 166.7 | ns |  |  |
| 1161 | 5B | *IWB29509* | 167.0 | ns |  |  |
| 1162 | 5B | *IWB67752* | 167.2 | * | *65591* |  |
| 1163 | 5B | *IWB63871* | 170.1 | * |  |  |
| 1164 | 5B | *IWB7739* | 175.9 | ns | *65055, 73941* |  |
| 1165 | 5B | *IWB46237* | 176.2 | ns |  |  |
| 1166 | 5B | *IWB70408* | 176.5 | ns | *34482, 11661, IWA3358* |  |
| 1167 | 5B | *IWB7836* | 177.3 | ns | *74021, 7835, 74020* |  |
| 1168 | 5B | *Xgwm118* | 177.6 | ns |  |  |
| 1169 | 5B | *IWB65485* | 177.6 | ns | *8808, 7302.1, 68170.1, 5688, 59928, 52723, 72711, 65484, 36771, 73046, 48153, 65802, 72714, 36621, 28207, 27150, 72712, 10360, 10359, 18224* |  |
| 1170 | 5B | *IWB72674* | 178.5 | ns |  |  |
| 1171 | 5B | *IWB66258* | 178.7 | ns | *66257* |  |
| 1172 | 5B | *IWB71272* | 179.3 | ns | *IWA8006, 63348, 47639, 64061, 4410, IWA8391, 65926, 47640, 25477, 73352, 39994, 28158, 71271, IWA4329, 4412* |  |
| 1173 | 5B | *IWA7374* | 181.9 | ns |  |  |
| 1174 | 5B | *IWB69552* | 183.1 | ns | *73829, 69551, 4803* |  |
| 1175 | 5B | *IWB24987* | 187.5 | ns | *47090, 74919, 28598, 47087, 20541, 26850, 46556, 29548, 45643, 28597, 44680, 44679, 29164, 59314* |  |
| 1176 | 5B | *IWB47086* | 187.8 | ns |  |  |
| 1177 | 6A | *IWB10105* | 0.0 | ns |  |  |
| 1178 | 6A | *IWB7762* | 1.1 | ns | *10814, 39292, 11785, 7353, 10458, 59591, 71875, 10558, 47842, 10557, 60891* |  |
| 1179 | 6A | *IWA5416* | 1.4 | ns |  |  |
| 1180 | 6A | *IWB12447* | 2.0 | ns | *11910, 26592, 33855, 5029, 12448, 44965, 65570, 2598* |  |
| 1181 | 6A | *IWB60233* | 2.8 | ns |  |  |
| 1182 | 6A | *IWB11316.1* | 3.3 | ns |  |  |
| 1183 | 6A | *IWA7913* | 4.2 | ns |  |  |
| 1184 | 6A | *IWB9075* | 4.3 | ns | *72428, 6902.1, 37515* |  |
| 1185 | 6A | *IWB35466* | 6.8 | ns | *11242* |  |
| 1186 | 6A | *IWB72510* | 7.1 | ns | *8452* |  |
| 1187 | 6A | *IWA4552* | 8.9 | ns | *72905, 10743, 65474, 31824, 9288, 20946, 14410, 74738, 29603, 9150, IWA6999.2, 34573, 9287, 65918, 17902, 48174, 25242, 65379, 47076, 9439, 5854, 47077, 13112, 65350, 8061, 36781, 6871.1, 9985, IWA4962, 11711, IWA7287, 63698, 9898, 59995.1, 50019, IWA4551, 25243.1* |  |
| 1188 | 6A | *IWB28201* | 14.0 | ns | *44960, 7337, 67933, 29014, 28559, IWA51, 13400, IWA770, 28199* |  |
| 1189 | 6A | *IWB33750* | 14.3 | ns | *61273, 10958, 75134, 71485, 33749, 27480, IWA4961, 10710* |  |
| 1190 | 6A | *IWB26040* | 15.2 | ns | *58142, 36543, 66218, 29622, 66602, IWA8608, 73285, 44659, 11433, 10776, 50402, 59681, 10775, 66603, 36242, 6044, 6401, 66606, 66601, 66600, 10227* |  |
| 1191 | 6A | *IWB9567* | 15.7 | ns |  |  |
| 1192 | 6A | *IWB12224* | 18.4 | ns | *72039* |  |
| 1193 | 6A | *Xgpw3087* | 20.1 | ns |  |  |
| 1194 | 6A | *IWB67078* | 21.4 | ns | *63758, 67079, 58031* |  |
| 1195 | 6A | *IWB73265* | 25.2 | ns | *IWA3320 ,IWA3319, IWA7444, 32523, 25326, 7442, 64837, 32524, 65633, IWA3322, 56969, IWA621, 66393, 71378, 73939, 73937, 32522, 72866, 72868, 44471, 72869, 65434, 65923, 72867, 65862, 35338, 32525, IWA5402, 70421, 73938, 39835, 10887, IWA5401* |  |
| 1196 |  | IWB70731 | 25.2 | ns |  |  |
| 1197 | 6A | *IWB66392* | 25.2 | ns |  |  |
| 1198 | 6A | *IWB12439* | 25.8 | ns |  |  |
| 1199 | 6A | *IWB4117* | 28.2 | ns |  |  |
| 1200 | 6A | *IWB50336* | 29.1 | ns |  |  |
| 1201 | 6A | *IWB7500* | 30.6 | ns |  |  |
| 1202 | 6A | *IWB73817* | 33.5 | ns |  |  |
| 1203 | 6A | *IWB3692* | 33.5 | ns | *34957* |  |
| 1204 | 6A | *IWB9036* | 35.0 | ns | *10644, 13025* |  |
| 1205 | 6A | *IWB26966* | 35.3 | ns |  |  |
| 1206 | 6A | *IWB10234* | 37.0 | ns | *IWA6311* |  |
| 1207 | 6A | *IWA902* | 37.0 | ns |  |  |
| 1208 | 6A | *IWB6293* | 40.4 | ns | *72208* |  |
| 1209 | 6A | *IWB17589* | 47.4 | ns | *52347, 59351, 52665, 35130, 34635, 52666, 35228* |  |
| 1210 | 6A | *IWA7438* | 47.7 | ns | *52712, 39921, 21378, 29660, 35121, 25812, 51879, 49990, 52050, 39414, IWA6095, IWA5656, IWA7492, 65577, 28056, IWA3782, 20844, 52222, IWA5421, 26704, IWA7847, 9334, 9221, 10857, IWA6928, 43905, 63144, 10774, IWA7354, 65773, 9705, IWA5376, 11029, 35256, 11434, 44856, 9704, 8871, 27029, 12060, 74895, 7152, 78518, 7175, IWA3879, 9144, IWA7349, 11484, IWA28, IWA7940, IWA4371, 59409, 10629, 7319, 59349, 52786, 36276, IWA3356, 27992, 52105, 7951, 26877, 9444, 59543, 21080, 39584, 35212, 34757, IWA4029, 63073, 59402, 37679, 34375, 6555, 65393, 35915, 65689, 35453, 35040, 34522, 21483, 8045, 34811, 35565, 7420, 6351, 11026, 10321, 9507, 5437, 3897, IWA6084, 7526, 4299, IWA5441, IWA3408, 39455, 31491* |  |
| 1211 | 6A | *IWA6927* | 48.0 | * |  |  |
| 1212 | 6A | *IWA8341* | 48.9 | ns |  |  |
| 1213 | 6A | *IWA3526* | 50.1 | * | *IWA3527, 3738* |  |
| 1214 | 6A | *IWB10038* | 50.7 | ns | *IWA428, 74380, 60499, 62878, 35526, 57264, 65965, IWA8348, 25702* |  |
| 1215 | 6A | *IWB6717* | 50.9 | ns |  |  |
| 1216 | 6A | *IWB73642* | 53.0 | ns |  |  |
| 1217 | 6A | *IWB69886* | 53.6 | ns | *30925, 67403, 31234* |  |
| 1218 | 6A | *IWA6962* | 53.9 | ns | *IWA3463, 33567, 39171, 74244, 52504, IWA3482, IWA8306, IWA3483, 35169, 31050* |  |
| 1219 | 6A | *IWB36081* | 55.1 | ns | *75285, 62830, 6191, 5848, 36195, 36012, 8206* |  |
| 1220 | 6A | *IWA6033* | 56.1 | ns | *IWA6012, IWA4842, 8036, 34964, 62858* |  |
| 1221 | 6A | *IWB39473* | 56.4 | ns | *5996* |  |
| 1222 | 6A | *IWB9844* | 56.7 | ns | *33751, 33872, 35485* |  |
| 1223 | 6A | *IWB60244* | 56.9 | ns | *27445, 6511, 6286* |  |
| 1224 | 6A | *IWB7281* | 57.2 | ns | *10739* |  |
| 1225 | 6A | *IWA38* | 57.8 | ns | *36100, 35929, 65994, 36365* |  |
| 1226 | 6A | *IWB40151* | 58.4 | ns |  |  |
| 1227 | 6A | *IWB5338* | 59.0 | ns |  |  |
| 1228 | 6A | *IWB75144* | 59.3 | ns |  |  |
| 1229 | 6A | *IWB9445* | 60.8 | ns |  |  |
| 1230 | 6A | *IWB73569* | 67.1 | ns |  |  |
| 1231 | 6A | *IWB73413* | 69.5 | ns | *73414, 13073, 58127* |  |
| 1232 | 6A | *IWB9616* | 71.2 | ns |  |  |
| 1233 | 6A | *IWB9468* | 72.7 | ns | *60449* |  |
| 1234 | 6A | *IWB64974* | 74.1 | ns |  |  |
| 1235 | 6A | *IWB33879* | 74.4 | ns | *35971, 14335, 69370* |  |
| 1236 | 6A | *IWB69770* | 87.4 | ns | *64875* |  |
| 1237 | 6A | *IWB61092* | 87.7 | ns | *45292, 71441* |  |
| 1238 | 6A | *Xgwm169* | 88.7 | ns |  |  |
| 1239 | 6A | *IWB6825* | 88.7 | ns |  |  |
| 1240 | 6A | *IWB31101* | 89.0 | ns |  |  |
| 1241 | 6A | *IWB12203* | 94.4 | ns | *65198, 65197, 69637, 63000, 50538* |  |
| 1242 | 6A | *IWB46146* | 94.7 | ns |  |  |
| 1243 | 6A | *IWB7048* | 95.0 | ns |  |  |
| 1244 | 6A | *IWB73296.1* | 99.3 | ns |  |  |
| 1245 | 6A | *IWB11953* | 99.6 | ns |  |  |
| 1246 | 6A | *IWB3818* | 99.9 | ns | *IWA504, 28546, 35685, IWA4603, IWA4602* |  |
| 1247 | 6A | *IWB73927* | 100.4 | ns | *58641, IWA6116, 57644, 37899, 35343, 29652* |  |
| 1248 | 6A | *IWB5971* | 100.4 | ns | *5419* |  |
| 1249 | 6A | *IWB58802* | 100.4 | ns |  |  |
| 1250 | 6A | *Xgwm617* | 100.4 | * |  |  |
| 1251 | 6A | *Xgwm427* | 100.7 | ns |  |  |
| 1252 | 6A | *IWB56463* | 100.7 | ns |  |  |
| 1253 | 6A | *IWB14865* | 100.7 | ns | *64255* |  |
| 1254 | 6A | *IWB71122* | 100.7 | ns | *35245, 57413, 71119, 10261* |  |
| 1255 | 6A | *IWB57893* | 102.2 | ns | *75127, 31096, IWA4691, 35122* |  |
| 1256 | 6A | *IWA8595* | 102.8 | ns |  |  |
| 1257 | 6A | *IWB71956* | 102.8 | ns | *6085, 72197, 47393.1, 36618, IWA4918.1, 8923.1, 69846, 33717.1, 69845* |  |
| 1258 | 6A | *IWB21359* | 102.8 | ns |  |  |
| 1259 | 6A | IWB34398 | 103.4 | ns | *73398, 32665.1* |  |
| 1260 | 6A | IWB30724.1 | 103.4 | ns |  |  |
| 1261 | 6A | IWB33694 | 103.4 | ** |  |  |
| 1262 | 6A | IWB5885.1 | 103.4 | ns |  |  |
| 1263 | 6A | IWB56916 | 103.4 | ns |  |  |
| 1264 | 6A | IWB64114 | 103.7 | ns |  |  |
| 1265 | 6A | IWB73410 | 103.7 | ns | *73398, 32665.1* |  |
| 1266 | 6A | CK207347 | 104 | ns |  |  |
| 1267 | 6A | KASPSr13 | 104 | ns |  |  |
| 1268 |  | BE403950 | 104 | ns |  |  |
| 1269 |  | Xrwgsnp6 | 104 | * |  |  |
| 1270 |  | Xrwgsnp7 | 104.8 | ** |  |  |
| 1271 | 6B | *IWB3172* | 0.0 | ns | *10604, 45119, 45121.1* |  |
| 1272 | 6B | *IWB7728* | 1.1 | ns |  |  |
| 1273 | 6B | *IWB67429* | 2.8 | ns |  |  |
| 1274 | 6B | *IWB6572* | 3.9 | * | *36361, 6463, 39540, 8078, 39541, 73020, 56606, 68662, 56604* |  |
| 1275 | 6B | *IWB6212* | 3.9 | * | *25142.1, 3282, 56605, 63423, 71552, 14443, 71436, 2946, 57350, 73489, 3564, 2693, 10358, 65546, 63938, 57351, 59320, 71434, IWA3297, 29542, 71433, 67427, 29541, 3563, 71431, 71432, IWA3298, 71435, 67428* |  |
| 1276 | 6B | *IWB59377* | 4.4 | * | *21062, 58008, 60101, 58009, IWA7070, 59378, IWA8477* |  |
| 1277 | 6B | *IWB9057* | 5.3 | * | *46708* |  |
| 1278 | 6B | *IWB71329* | 6.1 | * | *71331, 65148, 18280, 72322, 4662, 25035* |  |
| 1279 | 6B | *IWB74087* | 6.4 | * | *28262, 13056* |  |
| 1280 | 6B | *IWB28633* | 10.2 | ns | *28634* |  |
| 1281 | 6B | *IWB47396* | 10.4 | ns |  |  |
| 1282 | 6B | *IWB65787* | 11.6 | * | *52738, 7119, 57421, 57334, 52739, IWA4610, 6216, 6353, 69722, 45870* |  |
| 1283 | 6B | *IWB64815* | 19.2 | ns |  |  |
| 1284 | 6B | *IWB26083* | 19.5 | ns | *10163* |  |
| 1285 | 6B | *IWB60019* | 20.4 | * |  |  |
| 1286 | 6B | *IWB7937* | 20.7 | ns | *60219, 65635, IWA4290* |  |
| 1287 | 6B | *IWB7935* | 21.5 | ns |  |  |
| 1288 | 6B | *IWB73576* | 21.8 | ns |  |  |
| 1289 | 6B | *IWB33652* | 23.5 | ns | *IWA4010, IWA4011, 34432, IWA52, 32767, 32769, 63285, 7723, 59637.1, 59107* |  |
| 1290 | 6B | *Xwmc487* | 27.8 | ns |  |  |
| 1291 | 6B | *IWB11642* | 28.4 | ns | *11641, 11643* |  |
| 1292 | 6B | *IWB26775* | 28.6 | ns | *37846, 65084, 36341, 73955* |  |
| 1293 | 6B | *IWB58963* | 29.2 | ns |  |  |
| 1294 | 6B | *IWB62877* | 29.5 | ns | *36095, 46957* |  |
| 1295 | 6B | *IWB59118* | 31.2 | ns | *3182, 12568, 52064, 57839, 7074, 11192, 59119, 57840, 75076, 72594, 44180, 59120, 10742* |  |
| 1296 | 6B | *IWA8228* | 32.7 | ns |  |  |
| 1297 | 6B | *IWB69190* | 37.2 | ns | *10716, 25627, 10351* |  |
| 1298 | 6B | *Xwmc494* | 40.7 | * |  |  |
| 1299 | 6B | *IWB63807* | 42.3 | ns | *43931, 44083, 33797, 65961, 33796, 10087, 33795, 63808* |  |
| 1300 | 6B | *IWB9609* | 42.6 | ns | *9354* |  |
| 1301 | 6B | *IWB72401* | 43.4 | ns |  |  |
| 1302 | 6B | *IWB71937* | 43.7 | ns |  |  |
| 1303 | 6B | *IWA862* | 45.7 | ns |  |  |
| 1304 | 6B | *IWA7239* | 45.7 | ns | *IWA1243, 31535, 5533, IWA842, 60404, IWA861* |  |
| 1305 | 6B | *IWB45612* | 46.6 | ns | *IWA4408, 35555, 39117, 44230* |  |
| 1306 | 6B | *IWB45887* | 47.2 | ns | *69518* |  |
| 1307 | 6B | *IWB3473* | 48.3 | ns | *IWA3300, 34422, 11556, 10625* |  |
| 1308 | 6B | *IWB71635* | 49.5 | ns | *43853, 43852, 7680, 71636, 73728, 73303, 68621* |  |
| 1309 | 6B | *IWB26716* | 50.6 | ns | *68256, 68254, 68255* |  |
| 1310 | 6B | *IWB71284* | 52.1 | * | *25823, 10704, 33273, 74027, 26976, 35654* |  |
| 1311 | 6B | *IWB64914* | 52.4 | * |  |  |
| 1312 | 6B | *IWB33858* | 52.9 | * | *IWA4730, 28975* |  |
| 1313 | 6B | *IWA6032* | 53.2 | ** |  |  |
| 1314 | 6B | *IWB73968* | 53.5 | * | *33618, 70635, 68130, IWA7937, IWA3501* |  |
| 1315 | 6B | *IWB65137* | 53.8 | ** |  |  |
| 1316 | 6B | *IWB72416* | 55.2 | ** | *72202, 58313* |  |
| 1317 | 6B | *IWA3676* | 56.6 | ** | *25217, 56415* |  |
| 1318 | 6B | *IWB29373* | 57.7 | ** | *8809, 29372, 3465, 35384* |  |
| 1319 | 6B | *IWB59925* | 58.0 | *** | *59055, 5941, 59737, 3608, 6452, IWA6064* |  |
| 1320 | 6B | *IWB47211* | 58.3 | *** |  |  |
| 1321 | 6B | *IWB10393* | 59.4 | ** |  |  |
| 1322 | 6B | *IWB34946* | 62.1 | *** | *14722, 70530, 57022, 66131, 63057, 60265, 47313* |  |
| 1323 | 6B | *Xwmc397* | 63.4 | **** |  |  |
| 1324 | 6B | *IWA6153* | 63.7 | *** | *28873, 70979, 36648, 56968, IWA5504, 48185, 66246, 31348, 52110.1,18183, 48400, 46486, 60984, IWA4170, 60288, 45266, 34780, IWA4848, IWA5225, IWA3917, 50447, 56703* |  |
| 1325 | 6B | *IWB47049* | 64.3 | ***** | *28268, 33747, 67439, 25027, 65914, 69753, 33826, 71605, 32014, 71603* |  |
| 1326 | 6B | *IWB11783* | 64.6 | **** |  |  |
| 1327 | 6B | *IWA3459* | 65.7 | ***** |  |  |
| 1328 | 6B | *IWB35737* | 66.0 | ***** | *47634, IWA8165, IWA5346, IWA5345, 31411, 6932, 68769, 73415* |  |
| 1329 | 6B | *IWB31833* | 66.3 | ***** | *12289, 58440, 35826* |  |
| 1330 | 6B | *IWA1251* | 67.2 | ***** | *36146* |  |
| 1331 | 6B | *IWB68061* | 67.7 | ***** | *21249, IWA971, IWA4502, IWA4501, 46344, 8492, IWA755, 12855, IWA7962, IWA4500* |  |
| 1332 | 6B | *IWA5625* | 68.3 | ***** | *65680, 31076, IWA4435, 65195, 74159, 34321* |  |
| 1333 | 6B | *IWB35946* | 69.5 | ***** | *17986, IWA3289, 13603* |  |
| 1334 | 6B | *IWB45959* | 69.7 | ***** | *IWA7084, IWA3679, 35399, 57949, IWA3354, 47281, 56906, 34839, 20733, 45958, 34510, 30825, IWA1017, 35401, 68902* |  |
| 1335 | 6B | *IWB72677* | 72.7 | ***** |  |  |
| 1336 | 6B | *IWB57801* | 73.0 | ***** |  |  |
| 1337 | 6B | *IWB50214* | 73.3 | ***** | *72870, 25440, 14861, 57917, 64884, 44917* |  |
| 1338 | 6B | *IWB73860* | 73.6 | ***** |  |  |
| 1339 | 6B | *IWB75290* | 77.1 | ***** | *71501, 73456* |  |
| 1340 | 6B | *IWB63539* | 77.4 | ***** | *63538, 13090, 73501, 74611* |  |
| 1341 | 6B | *IWB26622* | 77.6 | ***** | *73599, 70007* |  |
| 1342 | 6B | *IWB73387* | 78.2 | ***** | *73386* |  |
| 1343 | 6B | *Xbarc79* | 79.4 | ***** |  |  |
| 1344 | 6B | *IWB71734* | 80.4 | ***** |  |  |
| 1345 | 6B | *IWB71780* | 80.6 | ***** | *73374, 61094, IWA283* |  |
| 1346 | 6B | *IWB72209* | 80.9 | ***** | *71113* |  |
| 1347 | 6B | *IWA1263* | 81.2 | ***** | *25654* |  |
| 1348 | 6B | *IWB61166* | 82.6 | ***** |  |  |
| 1349 | 6B | *IWB33834* | 82.9 | ***** | *71546, 35377, IWA3735* |  |
| 1350 | 6B | *IWB72305* | 83.2 | ***** | *73224, 73225* |  |
| 1351 | 6B | *IWB14152* | 85.1 | ***** | *32217* |  |
| 1352 | 6B | *IWB65825* | 87.9 | ***** | *43845* |  |
| 1353 | 6B | *IWB60027* | 88.5 | ***** |  |  |
| 1354 | 6B | *IWB9394* | 90.4 | ***** |  |  |
| 1355 | 6B | *IWB9393* | 91.5 | ***** |  |  |
| 1356 | 6B | *IWA8383* | 93.5 | ***** | *46624* |  |
| 1357 | 6B | *Xbarc24* | 96.1 | ***** |  |  |
| 1358 | 6B | *IWB58636* | 102.1 | ***** | *25254, 59482, 58733* |  |
| 1359 | 6B | *IWB71618* | 103.6 | ***** | *70643* |  |
| 1360 | 6B | *IWA404* | 104.7 | ***** | *71722, 58494, 29386, 72325, IWA405* |  |
| 1361 | 6B | *IWB46771* | 105.0 | ***** |  |  |
| 1362 | 6B | *IWA8064* | 110.2 | ** | *36530* |  |
| 1363 | 6B | *IWB45036* | 114.9 | * |  |  |
| 1364 | 6B | *IWB26890* | 115.2 | ns | *39172, 47632* |  |
| 1365 | 6B | *IWB48236* | 115.5 | * |  |  |
| 1366 | 6B | *IWB73204* | 117.8 | * | *60708, 52227, 50537, 65276* |  |
| 1367 | 6B | *IWB34340* | 118.9 | ns | *7417, 68655* |  |
| 1368 | 6B | *IWB27763* | 133.5 | ns | *63554, 69466, 56963, 50278, 45524, IWA7116, 27200* |  |
| 1369 | 6B | *Xbarc134* | 144.1 | ns |  |  |
| 1370 | 6B | *IWB74863* | 152.3 | *** |  |  |
| 1371 | 6B | *IWB70316* | 152.6 | ** | *72471* |  |
| 1372 | 6B | *IWB26627* | 152.9 | ** | *31443, 67504, 3121, 59005, 57728, IWA3880, 73836, 20749, 26626, 26624, 69375, 57727, IWA4244, 59116, 6219, 45263, 59006* |  |
| 1373 | 6B | *IWA7098* | 155.2 | ** |  |  |
| 1374 | 6B | *IWB31091* | 158.4 | ns |  |  |
| 1375 | 6B | *IWB8341* | 158.6 | ns | *68750, 10824* |  |
| 1376 | 6B | *IWB65437* | 160.4 | ns |  |  |
| 1377 | 6B | *IWB47825* | 161.3 | ns | *64004, 19435* |  |
| 1378 | 6B | *IWB66055* | 161.9 | ns |  |  |
| 1379 | 6B | *IWA3947* | 164.5 | * |  |  |
| 1380 | 6B | *IWB72523* | 165.4 | ** | *58199, 34443* |  |
| 1381 | 6B | *IWB34745* | 166.8 | *** |  |  |
| 1382 | 6B | *IWB74864* | 167.7 | ** | *4183, 3696, 68721, 34507, 44253, 74861, 44252, 13511, 74865, 74862, 13510* |  |
| 1383 | 6B | *IWA4919* | 168.5 | ** |  |  |
| 1384 | 6B | *IWA4920* | 169.3 | *** |  |  |
| 1385 | 6B | *IWB8425* | 169.8 | *** | *71412, 70379* |  |
| 1386 | 6B | *IWB70734* | 170.4 | ** |  |  |
| 1387 | 7A | *IWB12610* | 0.0 | ns | *26552* |  |
| 1388 | 7A | *IWB25834* | 0.6 | ns | *7458, 75056, 73433, 12369* |  |
| 1389 | 7A | *IWB20752* | 1.1 | ns |  |  |
| 1390 | 7A | *IWB73683* | 2.6 | ns |  |  |
| 1391 | 7A | *IWB11001* | 6.7 | ns |  |  |
| 1392 | 7A | *IWB3124* | 11.5 | ns |  |  |
| 1393 | 7A | *IWB29333* | 13.8 | ns |  |  |
| 1394 | 7A | *IWB31010* | 16.9 | ns |  |  |
| 1395 | 7A | *IWB7400* | 17.2 | ns |  |  |
| 1396 | 7A | *IWB74024* | 18.3 | ns | *9904, 9397, 67721, 9078, 65110, 19485, 71683, 65111, 58667, 74161.1, 18185* |  |
| 1397 | 7A | *IWA954* | 18.6 | ns | *74238* |  |
| 1398 | 7A | *IWB27944* | 22.3 | ns |  |  |
| 1399 | 7A | *IWB73570* | 22.3 | ns | *73571, 9383* |  |
| 1400 | 7A | *IWB59817* | 23.8 | ns |  |  |
| 1401 | 7A | *Xbarc127* | 27.6 | ns |  |  |
| 1402 | 7A | *IWB44791* | 29.3 | ns |  |  |
| 1403 | 7A | *IWB47321* | 29.9 | ns | *8374* |  |
| 1404 | 7A | *IWB12197* | 30.1 | ns | *12020, 73688, 12198, 6718, 68545, 68544* |  |
| 1405 | 7A | *IWB34499* | 33.3 | ns |  |  |
| 1406 | 7A | *IWB59294* | 34.1 | ns | *57059, 64911* |  |
| 1407 | 7A | *IWB39676* | 34.7 | ns | *57737, 6198, 7484* |  |
| 1408 | 7A | *IWB33919* | 35.0 | ns |  |  |
| 1409 | 7A | *IWA3760* | 35.2 | ns | *56953, 52818* |  |
| 1410 | 7A | *IWB73665* | 39.4 | ns | *11441, 62921* |  |
| 1411 | 7A | *IWB8251* | 40.0 | ns |  |  |
| 1412 | 7A | *IWB11091* | 40.3 | ns |  |  |
| 1413 | 7A | *IWB26398* | 42.3 | ns | *74061, 36370* |  |
| 1414 | 7A | *IWB48426* | 43.1 | ns | *IWA4386* |  |
| 1415 | 7A | *Xwmc83* | 45.5 | ns |  |  |
| 1416 | 7A | *IWB25760* | 47.1 | ns | *32666* |  |
| 1417 | 7A | *IWB63867* | 47.3 | ns | *21011, 17515.1, 72710, 8241, 28082, 58671* |  |
| 1418 | 7A | *IWB60588* | 47.6 | ns | *34025, 7985* |  |
| 1419 | 7A | *IWA8492* | 48.4 | ns | *66777* |  |
| 1420 | 7A | *IWA4181* | 50.4 | ns | *IWA4180, 6339, 14901* |  |
| 1421 | 7A | *IWB70597* | 50.7 | ns |  |  |
| 1422 | 7A | *IWB60067* | 51.0 | ns | *60068, IWA7205* |  |
| 1423 | 7A | *IWB4724.1* | 52.7 | ns |  |  |
| 1424 | 7A | *Xbarc174* | 54.2 | ns |  |  |
| 1425 | 7A | *IWB12628* | 55.4 | ns |  |  |
| 1426 | 7A | *IWA5258* | 56.0 | ns | *3129, 62609, 34932, 45866, 46130, 7104, 8471* |  |
| 1427 | 7A | *IWB27867* | 56.6 | ns |  |  |
| 1428 | 7A | *IWB11841* | 57.1 | ns |  |  |
| 1429 | 7A | *IWB8372* | 57.4 | ns |  |  |
| 1430 | 7A | *IWB8703* | 57.7 | ns |  |  |
| 1431 | 7A | *IWB28629* | 58.8 | ns |  |  |
| 1432 | 7A | *IWB12533* | 59.0 | ns | *29169, 65530* |  |
| 1433 | 7A | *IWB7752* | 59.6 | ns | *3676* |  |
| 1434 | 7A | *IWB11234* | 60.1 | ns |  |  |
| 1435 | 7A | *IWB60270* | 60.9 | ns | *34318, 43994, 60651, 43995, 34967, 34718, 11693, 60652, 43993, 50318, IWA7500, 50319, 34968, 26683, 43996* |  |
| 1436 | 7A | *IWB72739* | 61.4 | ns |  |  |
| 1437 | 7A | *IWB9633* | 63.7 | ns |  |  |
| 1438 | 7A | *IWB45830* | 65.1 | ns |  |  |
| 1439 | 7A | *IWB8555* | 65.4 | ns | *72148* |  |
| 1440 | 7A | *IWA305* | 65.9 | ns | *70592* |  |
| 1441 | 7A | *IWB48549* | 67.0 | ns | *32122* |  |
| 1442 | 7A | *IWB35738* | 67.3 | ns | *48383, 46718, 49814, 66115* |  |
| 1443 | 7A | *IWB34110* | 69.0 | ns |  |  |
| 1444 | 7A | *IWB33920* | 69.2 | ns |  |  |
| 1445 | 7A | *IWB8620* | 69.2 | ns | *14782, IWA796, 35092, 50013, 35799, IWA797* |  |
| 1446 | 7A | *IWB72694* | 69.5 | ns | *4178, 36190* |  |
| 1447 | 7A | *IWB35503* | 71.7 | ns | *56070.1, 9983, 9702, 59583, 62936, IWA6183, 34519, 45583, 39122, 34519, 45583, 39122, IWA7472, 69646, 9049, 10939, 28701, 50244, 8022, 52438, 9602, 52549, 69937, 6806, 51943, 60298, 20679, 52367, 68081, 9156, IWA788, 72880, 39180, 65015, 59874, 11089, IWA8171* |  |
| 1448 | 7A | *IWA208* | 73.4 | ns | *6006, 8231, 11537, 36528, 12101, 35659, 12129, 50557, IWA4277, 35894, 21459* |  |
| 1449 | 7A | *IWB59123* | 74.3 | ns | *IWA7293, 34725, 14967, 10876* |  |
| 1450 | 7A | *IWA4638* | 74.5 | ns | *IWA4639, IWA4637, 11698, 61040* |  |
| 1451 | 7A | *IWB11124* | 74.8 | ns | *36186* |  |
| 1452 | 7A | *IWB9558* | 76.2 | ns | *4194, 8563, 7607* |  |
| 1453 | 7A | *IWB8935* | 76.8 | ns | *9490* |  |
| 1454 | 7A | *IWA4601* | 78.8 | ns |  |  |
| 1455 | 7A | *IWA8073.1* | 81.7 | ns |  |  |
| 1456 | 7A | *IWB46770* | 82.9 | ns | *71702, 71703, 72620, 52135, 58341, IWA4996, 72486, IWA448, 27404* |  |
| 1457 | 7A | *IWB45735* | 83.1 | ns | *IWA4062, 51810, IWA4672, 5811, 11559, 34535, 8790, IWA3662, 65986, IWA7917, IWA8248, 35881, 21281, IWA4411, 29543, IWA5526, 33880, 45736, 21282, 34290, IWA4735, IWA3925, 26548.1, 31301, IWA4817, IWA4037, 6695* |  |
| 1458 | 7A | *IWB29555* | 84.2 | ns | *29556, 7146, 7238, IWA808* |  |
| 1459 | 7A | *IWB68969* | 84.8 | ns | *72890, 71633, IWA6940, 68970, 34700, 68971* |  |
| 1460 | 7A | *IWB10959* | 85.1 | ns |  |  |
| 1461 | 7A | *IWB44085* | 88.2 | ns |  |  |
| 1462 | 7A | *IWB14692* | 89.4 | ns | *34090, 64111, 49799* |  |
| 1463 | 7A | *Xwmc607* | 93.5 | ns |  |  |
| 1464 | 7A | *IWB4809* | 96.4 | ns | *8359* |  |
| 1465 | 7A | *IWB72494* | 96.7 | ns |  |  |
| 1466 | 7A | *IWB9063* | 98.1 | ns | *9062, 14235, 3470, 4764* |  |
| 1467 | 7A | *IWB25280* | 99.2 | ns |  |  |
| 1468 | 7A | *IWB8305* | 99.7 | ns | *69294, 52737* |  |
| 1469 | 7A | *IWB47653* | 100.0 | ns |  |  |
| 1470 | 7A | *IWB6963* | 100.2 | ns |  |  |
| 1471 | 7A | *IWB3803* | 100.8 | ns | *73653, 10968* |  |
| 1472 | 7A | *IWB70469* | 101.6 | ns |  |  |
| 1473 | 7A | *IWB73704* | 103.5 | ns |  |  |
| 1474 | 7A | *IWB52378* | 108.3 | ns | *74326, 11584, 57762, 59224, 10093, 10552* |  |
| 1475 | 7A | *IWB57246* | 108.6 | ns |  |  |
| 1476 | 7A | *IWB46635* | 108.9 | ns |  |  |
| 1477 | 7A | *IWB46703* | 113.3 | ns |  |  |
| 1478 | 7A | *IWB7632* | 114.2 | ns |  |  |
| 1479 | 7A | *IWB74845* | 115.3 | ns | *61074* |  |
| 1480 | 7A | *IWB45179* | 115.6 | ns | *58299* |  |
| 1481 | 7A | *IWB58863* | 115.8 | ns |  |  |
| 1482 | 7A | *IWB36250* | 116.4 | ns |  |  |
| 1483 | 7A | *IWB35048* | 117.8 | ns |  |  |
| 1484 | 7A | *IWB69898* | 118.4 | ns | *35597* |  |
| 1485 | 7A | *IWA4621* | 118.6 | ns | *IWA4620* |  |
| 1486 | 7A | *IWB36680* | 119.4 | ns |  |  |
| 1487 | 7A | *IWB6983* | 126.8 | ns | *25011, 25012.1, 9574, 17825* |  |
| 1488 | 7A | *IWB44281* | 127.0 | ns |  |  |
| 1489 | 7A | *IWB57321* | 127.3 | ns |  |  |
| 1490 | 7A | *IWB26081* | 128.1 | ns | *47016* |  |
| 1491 | 7A | *Xcfa2019* | 134.8 | * |  |  |
| 1492 | 7A | *IWB36108* | 144.1 | ns |  |  |
| 1493 | 7A | *IWB12038* | 144.7 | ns |  |  |
| 1494 | 7A | *IWB71398.1* | 146.3 | ns | *72397* |  |
| 1495 | 7A | *IWA6115* | 157.3 | ns | *2539, 12588, 35278, 7189, 14178.1, 12587, IWA4594, 48035, 65614, 46622, IWA7185, IWA4595, 35137, 48036, 12618* |  |
| 1496 | 7A | *IWA866* | 158.1 | ns | *IWA865, 8800, 34754, 14331, 14426* |  |
| 1497 | 7A | *IWB33997.1* | 158.4 | ns | *9343* |  |
| 1498 | 7A | *IWB63209* | 158.9 | ns | *72691* |  |
| 1499 | 7A | *IWB12246* | 161.5 | ns | *IWA4173* |  |
| 1500 | 7A | *IWA4434* | 161.5 | ns |  |  |
| 1501 | 7A | *IWB27807* | 162.0 | ns |  |  |
| 1502 | 7A | *IWB73997* | 162.3 | ns |  |  |
| 1503 | 7A | *IWB11121* | 162.9 | * |  |  |
| 1504 | 7A | *IWB73864* | 167.0 | ns | *6268, 72673, 26780* |  |
| 1505 | 7A | *IWB25307* | 168.3 | * |  |  |
| 1506 | 7A | *IWB64099* | 168.9 | * |  |  |
| 1507 | 7A | *IWB34223* | 169.5 | * | *IWA179, 5828* |  |
| 1508 | 7A | *IWA501* | 169.7 | * | *3406, IWA6923, 10682, 7367, 29001, 60813, 58668, 58344, 9146* |  |
| 1509 | 7A | *IWB6675* | 170.0 | * | *IWA7005* |  |
| 1510 | 7A | *IWB36793* | 171.7 | * | *36289, 6132, 8915, 65289, 17924, 67930, 10183.1, 7006, 65291, 71625, 25497, 57412.1, IWA7904, 60246* |  |
| 1511 | 7A | *IWB6037* | 171.7 | * | *52831* |  |
| 1512 | 7B | *IWB33121* | 0.0 | ns | *33120* |  |
| 1513 | 7B | *IWB46416* | 0.3 | ns | *65211, 65212* |  |
| 1514 | 7B | *IWB6919* | 10.0 | ns | *29117, 29118* |  |
| 1515 | 7B | *IWB10879* | 10.3 | ns | *66787, 17787, 65277, 72316, 45102, 69416, 69202, 74022* |  |
| 1516 | 7B | *IWB27107* | 11.4 | ns | *68516, 57207, 73685, 27109, 27108* |  |
| 1517 | 7B | *Xwmc323* | 11.4 | ns |  |  |
| 1518 | 7B | *IWA1181* | 11.7 | ns | *25434, 70085* |  |
| 1519 | 7B | *IWB6455* | 14.3 | ns |  |  |
| 1520 | 7B | *IWB3164* | 14.9 | ns |  |  |
| 1521 | 7B | *IWB40019* | 19.5 | ns |  |  |
| 1522 | 7B | *IWB74056* | 23.0 | ns |  |  |
| 1523 | 7B | *Xgwm537* | 31.7 | ns |  |  |
| 1524 | 7B | *IWA1220* | 35.0 | ns |  |  |
| 1525 | 7B | *IWA4977* | 36.4 | ns | *11322, 58277, 11323* |  |
| 1526 | 7B | *IWA4967* | 39.8 | ns | *IWA4966* |  |
| 1527 | 7B | *IWB44432* | 40.6 | ns |  |  |
| 1528 | 7B | *IWB39492* | 41.7 | ns |  |  |
| 1529 | 7B | *IWA3572* | 42.0 | ns | *3402, 70551, 27459, 71980, IWA7232, IWA7233, 49859, 72146, IWA3508, IWA3507, 3050* |  |
| 1530 | 7B | *IWB34204* | 42.5 | ns | *65888, 34206, 34205, 26214, 34035, 60479, IWA518, 3251, 34207* |  |
| 1531 | 7B | *IWB7646* | 43.1 | ns | *66826, 8603, 33909, 56817* |  |
| 1532 | 7B | *IWB11703* | 44.5 | ns | *31792* |  |
| 1533 | 7B | *IWB5875* | 44.8 | ns | *36566, 65114, 65115* |  |
| 1534 | 7B | *IWB68850* | 46.1 | ns | *34211* |  |
| 1535 | 7B | *IWB67435* | 46.4 | ns | *71513, 71514, 70399* |  |
| 1536 | 7B | *IWB35361* | 48.3 | ns | *7118* |  |
| 1537 | 7B | *IWB25649* | 49.7 | ns | *3173, IWA8456, 57670, 27525* |  |
| 1538 | 7B | *IWB5739* | 50.3 | ns | *67979* |  |
| 1539 | 7B | *IWB59024* | 51.4 | ns |  |  |
| 1540 | 7B | *IWB3531* | 53.1 | ns | *73107, IWA4873, 59235, 45667* |  |
| 1541 | 7B | *IWB72147* | 53.4 | ns | *5043, 46912* |  |
| 1542 | 7B | *IWB63035* | 56.3 | ns | *57007* |  |
| 1543 | 7B | *IWA7846* | 56.8 | ns | *IWA5210, IWA5663, 33982, 33983, IWA5662, IWA3663, IWA5661.1* |  |
| 1544 | 7B | *IWA5210* | 56.8 | ns |  |  |
| 1545 | 7B | *IWB59735* | 58.3 | ns | *IWA8233* |  |
| 1546 | 7B | *IWB51978* | 58.9 | ns | *31169, 21151, 57449* |  |
| 1547 | 7B | *IWB34369* | 59.1 | ns | *62994, 34748* |  |
| 1548 | 7B | *IWB4857* | 62.6 | ns | *5264, IWA881, 69619, 69305, 68814, 28367, 65104, 30874, 65103, 12878, 73957, 73956* |  |
| 1549 | 7B | *Xgwm46* | 63.2 | ns |  |  |
| 1550 | 7B | *IWB34169* | 64.7 | ns |  |  |
| 1551 | 7B | *IWB56734* | 65.0 | ns | *63670, 74638, 61270, 63688, 34131, IWA8418, 63369, 35064, IWA3886, 5634, 5632* |  |
| 1552 | 7B | *IWB68177* | 65.8 | ns | *45035, 8566, 73858, 65764, 71523, 63985, 71357, 71359, 60430, 58211, 66868, 12526, 14836, 7824, 62732, IWA8300, 34768, IWA7105, 73123, 10628, 7697, 4395* |  |
| 1553 | 7B | *IWB33246* | 66.4 | ns |  |  |
| 1554 | 7B | *IWB16218.1* | 67.5 | ns | *48336, 59968, 65369, IWA632, 26248, 69152, IWA8345, 14262* |  |
| 1555 | 7B | *IWB35172* | 72.0 | ns |  |  |
| 1556 | 7B | *IWB67917* | 76.9 | ns |  |  |
| 1557 | 7B | *IWB21203* | 77.5 | ns | *33961, 3003, 46739, 46738, 4352, 45752, 31460, 34033, 45718, 25156* |  |
| 1558 | 7B | *IWB69818* | 78.9 | ns |  |  |
| 1559 | 7B | *IWB69817* | 79.5 | ns |  |  |
| 1560 | 7B | *IWB69816* | 80.1 | ns |  |  |
| 1561 | 7B | *IWB60403* | 82.0 | ns | *71916* |  |
| 1562 | 7B | *IWB71925* | 82.3 | ns | *69052, 75309, 71838, 51895, 73909, 71924, 71926, 68015, 26171, 69050, 58270* |  |
| 1563 | 7B | *IWB34102* | 83.2 | * | *45912, 3274, 4812, 4816* |  |
| 1564 | 7B | *IWB56488* | 83.5 | * | *26679, 62673, 72331, 2834, 62671, 71496, 36651, 71733, 71582, 26677, 57075, 72641, 73338, 73339, 73340, 73341, 72960, 72961* |  |
| 1565 | 7B | *IWB35736* | 83.8 | * | *69881, IWA354, 13066, 33945, 61022, 13479, IWA355* |  |
| 1566 | 7B | *IWB33623* | 84.4 | * | *73892, 33622, 73891, 73890, 73395, 59338* |  |
| 1567 | 7B | *IWB66304* | 84.7 | ** | *73602, 69212, 60181, 68768, 68767, 73104, 69063, 71963, 26274, 71964, 73105, 64761* |  |
| 1568 | 7B | *IWB72925* | 85.3 | ** |  |  |
| 1569 | 7B | *IWB69740* | 85.8 | ns | *8765, 3161, 72254, 72504, 8766.1, 72505, 69741, IWA3987, 72979, 7521, 44319, 69449, 73035* |  |
| 1570 | 7B | *IWB73840* | 87.3 | * | *8805, 12371, 11767, 71827, 5830* |  |
| 1571 | 7B | *IWB43821* | 89.0 | ** | *70890, 44978, 68837, 47614, 57668, 47700, 26969, IWA8030, 45709, 68838, 26968, 68451* |  |
| 1572 | 7B | *IWB69447* | 92.1 | * | *69446, 69758, 69445, 59199, 59197* |  |
| 1573 | 7B | *IWB60324* | 92.4 | ** | *28473, 56832, 35732, 45276, 34138, 45277, 59506, 75085, 48219, 34139, 59887, IWA594, 34141, 48044, 34202* |  |
| 1574 | 7B | *IWB46260* | 94.8 | * | *27692, 27833, 66025, 27696* |  |
| 1575 | 7B | *IWA436* | 96.5 | ns | *IWA437, 5025, 58428, 58429* |  |
| 1576 | 7B | *IWB69178* | 100.7 | ns | *69177* |  |
| 1577 | 7B | *IWB75195* | 101.0 | ns | *34042, 46134* |  |
| 1578 | 7B | *IWB46046* | 101.3 | ns | *57705, 2717* |  |
| 1579 | 7B | *IWB9018* | 103.0 | ns | *7572, 7573* |  |
| 1580 | 7B | *IWB47178* | 104.4 | ns | *33975, 7710* |  |
| 1581 | 7B | *IWB7711* | 104.7 | ns |  |  |
| 1582 | 7B | *IWB9687* | 105.8 | ns |  |  |
| 1583 | 7B | *IWB25633* | 106.6 | ns |  |  |
| 1584 | 7B | *IWB28760* | 107.2 | ns | *36066, 28758, 6881, 15590, 28757, 28759, 57362, 9899* |  |
| 1585 | 7B | *IWA3928* | 110.2 | ns | *27012, IWA3927, 29268, 2638, 9120* |  |
| 1586 | 7B | *IWB69542* | 110.8 | ns | *56849, 56847, 56848, 66049, 57298, 58468, 73542, 28513, 63754* |  |
| 1587 | 7B | *IWB64750* | 112.8 | ns |  |  |
| 1588 | 7B | *IWB31227* | 115.6 | ns | *7642* |  |
| 1589 | 7B | *IWB10895* | 115.9 | ns |  |  |
| 1590 | 7B | *IWB3092* | 116.2 | ns | *58112, 17756.1* |  |
| 1591 | 7B | *IWB6608* | 116.2 | ns |  |  |
| 1592 | 7B | *Xwmc517* | 119.4 | ns |  |  |
| 1593 | 7B | *IWA836* | 122.7 | ns | *13136, 9204* |  |
| 1594 | 7B | *IWB8151* | 123.2 | ns |  |  |
| 1595 | 7B | *IWB25319* | 126.8 | ns | *73350, 73349, 72357* |  |
| 1596 | 7B | *IWB9384* | 128.5 | ns |  |  |
| 1597 | 7B | *IWB26359* | 129.1 | ns |  |  |
| 1598 | 7B | *IWB9261* | 129.6 | ns | *12159* |  |
| 1599 | 7B | *IWB72319* | 130.2 | ns | *60960, 72317, 72320, 72318, 35230.1, 20616, 69205* |  |
| 1600 | 7B | *IWB72939* | 131.1 | ns |  |  |
| 1601 | 7B | *IWA4306* | 131.7 | ns | *12171, 9373, 7845* |  |
| 1602 | 7B | *IWB13010.1* | 132.5 | ns | *8451* |  |
| 1603 | 7B | *IWB60959* | 134.2 | ns |  |  |
| 1604 | 7B | *IWB59225* | 139.8 | ns |  |  |
| 1605 | 7B | *IWA4309* | 142.4 | ns |  |  |
| 1606 | 7B | *IWB34900.1* | 143.9 | ns | *7099.1* |  |
| 1607 | 7B | *IWB68676* | 147.8 | ns |  |  |
| 1608 | 7B | *IWB34981* | 148.1 | ns | *31241, 6594, 59453, 68494, 52637, 31273, 63339, 60654, 43871, 27142, 34928, 59454, 68493, 35300, 27294, 37730, 71573, 59455, 48453, 35376, 48454, IWA130, 71571, 71575, 71574, 71572, 37731,* |  |
| 1609 | 7B | *IWB47992* | 148.4 | ns | *36230.1, IWA432.1, 35813, 35787, 9818, IWA431.1, 17743, 49960, 72479, 47991* |  |
| 1610 | 7B | *IWB7456* | 148.7 | ns | *68171, 47495, 58295, 7329.1, 63121, 71465, 20849, IWA4750, 57797, 36376, 34699, IWA4864, 68612, 71622, 35653, 2670, 28666, 5869, 6544, 75191, 7134, 35704* |  |
| 1611 | 7B | *IWB60879* | 149.9 | ns |  |  |
| 1612 | 7B | *IWA3387* | 152.9 | ns | *IWA439,3 56681, IWA3386* |  |
| 1613 | 7B | *IWB72683* | 153.5 | ns |  |  |
| 1614 | 7B | *IWB72830* | 153.8 | ns | *51787, IWA1091, 10844, 72829, 73725, 52641, 20596, 52567, 12168, 39684, 10477, 3163, 65152, IWA3513, 71559, 27699, 65154, 65153, 34837, 72828, 5626, IWA7260, 73726, 73399, 10476, 5034, IWA598, 60676, 9002, 6176, 11254, 60677* |  |
| 1615 | 7B | *IWB68215* | 154.1 | ns | *57979.1, 72834, 7268, 74566, 47563, 8911, 8215, 27056, 27055, 73659, 45400, 71977, 33998, 73658, 35547, 33999, 12068, 6574, 68214, 5105, 3581* |  |
| 1616 | 7B | *IWB74670* | 155.0 | ns | *73261, 31089, 63220, 56659, 47876, 26595, IWA3675, 74818, 57016, 68321, 64301, 64172, 71996, IWA4522, IWA4897, 13534, 64162, 5954, 73667, 44608, 58997, 31410, 14033, 64859, 8761, 57013, 60900, 27877, 69905, 11144, 6321, 58998, 11145, 8571, 27818, 13417, 7408, 3236, 12377.1, 5969, 64173, 64077, 44744, 64015, 75239, 74817, 57443, 27881, 58601, 50374, 11951, 11225, 49806, 29008, 71288, 59570, 9064, 27876, 75237, 39904, 59150, 48518, 66158, 34196, 47549, 28483, 45629, 62917, 8587, 31292, 7586, 68521, 11950, 45871, 57444, 12006, 8724, 64303, 10816, 10676, 70127, 64302, 58460, 13462, 65255, 8824, 10533, 58600, 71506, 31408, 12638, 34193, 13463, 74819, 71671, 56661, 29360, 25320, 69562, 46381, 14072, IWA4888, 6736, 56660, 56658, 56657, 47461, 44297, 34230, 27414, 9405, 69904, 44298, 28121, IWA7003, 56656, 65365, 10818, 9782, 57859, 50136, 6089, 7512, 34231, 13363, 13384, 4835, 66980, 59072, 58836, 26682, 3312, 12007, 34886, 10815, 63221, 48282, 9137, 8586, 8613, 58861.1, 8725, 64414, 13461, 8762, 39301, 21268, 28543, 31485, IWA8312, 10917, 8469, IWA7190, 50373, 25962* |  |
| 1617 | 7B | *IWB60899* | 155.5 | ns |  |  |
| 1618 | 7B | *IWB8577* | 156.7 | ns | *34117, 12557, 10240* |  |
| 1619 | 7B | *IWB25083* | 157.6 | ns |  |  |
| 1620 | 7B | *IWA7072* | 159.4 | ns |  |  |
| 1621 | 7B | *IWB47204* | 162.2 | ns |  |  |
| *, **, ***, ****, ***** indicate significant at P < 0.05, 0.01, 0.001, 0.00001, respectively; ns indicates non-significant markers. | | | | | |  |
| † The “IWB” was removed from the marker names. | | | | | |  |
