# Supplementary material for: Function and evolution of allelic variations of Sr13 conferring resistance to stem rust in tetraploid wheat (Triticum turgidum L.)
Source: Plant J. 2021 May 29;106(6):1674–91. doi: 10.1111/tpj.15263 (PMC8362117; doi:10.1111/tpj.15263)
Supplement: Supplementary file 10 — Table S4. Details of markers mapped in each chromosome/genome in the Rusty × PI 387696 recombinant inbred line population. Table S5. Coordinate positions (bp) of the markers associated with Sr13 on chromosome 6A linkage map in the reference genome RefSeq v1.0 of common wheat (IWGSC, 2018). Table S6. List of 67 simple sequence repeat markers used in bulked segregant analysis of a stem rust resistance gene derived from Triticum turgidum subsp. polonicum and size (bp) of the amplicon from each parent. [file TPJ-106-1674-s001.docx]

| **Table S4.** Details of markers mapped in each chromosome/genome in the Rusty × PI 387696 recombinant inbred line population. | | | | | | | | | |
| --- | --- | --- | --- | --- | --- | --- | --- | --- | --- |
|  | No of markers † | | | | | |  | Marker | No. of |
| Chromosome | SSR | EST- | 90K- | KASP | STARP | Total | Length ‡ | density | distorted |
|  |  | STS | SNP |  |  |  | (cM) | (cM) § | markers |
| 1A | 5 | 0 | 93 | 0 | 0 | 98 | 126.3 | 1.3 | 11 |
| 1B | 6 | 0 | 154 | 0 | 0 | 160 | 158.4 | 1.0 | 43 |
| 2A | 7 | 0 | 81 | 0 | 0 | 88 | 192.1 | 2.2 | 13 |
| 2B | 5 | 0 | 141 | 0 | 0 | 146 | 152.8 | 1.1 | 119 |
| 3A | 7 | 0 | 94 | 0 | 0 | 101 | 206.0 | 2.0 | 1 |
| 3B | 10 | 0 | 143 | 0 | 0 | 153 | 248.0 | 1.6 | 89 |
| 4A | 2 | 0 | 89 | 0 | 0 | 91 | 148.6 | 1.6 | 16 |
| 4B | 3 | 0 | 84 | 0 | 0 | 87 | 125.2 | 1.4 | 25 |
| 5A | 3 | 0 | 112 | 0 | 0 | 115 | 202.2 | 1.8 | 22 |
| 5B | 7 | 0 | 130 | 0 | 0 | 137 | 187.8 | 1.4 | 18 |
| 6A | 4 | 2 | 85 | 1 | 2 | 94 | 104.8 | 1.1 | 4 |
| 6B | 6 | 0 | 110 | 0 | 0 | 116 | 170.4 | 1.5 | 77 |
| 7A | 5 | 0 | 120 | 0 | 0 | 125 | 171.7 | 1.4 | 9 |
| 7B | 4 | 0 | 106 | 0 | 0 | 110 | 162.2 | 1.5 | 11 |
|  |  |  |  |  |  |  |  |  |  |
| A genome | 33 | 2 | 674 | 1 | 2 | 712 | 1,151.7 | 1.6 | 76 |
|  |  |  |  |  |  |  |  |  |  |
| B genome | 41 | 0 | 868 | 0 | 0 |  | 1,204.8 | 1.3 | 382 |
| Total | 74 | 2 | 1,542 | 1 | 2 | 1,621 | 2,356.5 | 1.5 | 458 |
| † Marker types are SSR = simple sequence repeat, EST-STS = expressed sequence tag derived sequence-tagged site, KASP = Kompetitive allele specific PCR, STARP = semi-thermal asymmetric reverse PCR, 90K-SNP = 90K iSelect SNP (single nucleotide polymorphism).  ‡ cM is length in centiMorgans.  § Marker density is genetic distance between markers (cM/marker). | | | | | | | | | |

| **Table S5**. Coordinate positions (bp) of the markers associated with *Sr13* on chromosome 6A linkage map in the reference genome RefSeq v1.0 of common wheat (IWGSC, 2018). | | | | |
| --- | --- | --- | --- | --- |
| 90 K Index | Marker | SNP Name | PI387696 map  Position (cM) | IWGSC_Ref Seq v1_position_coordinates (bp) |
| 10105 | *IWB10105* | BS00067630_51 | 0.0 | 5221776 – 5221676 |
| 67078 | *IWB67078* | Tdurum_contig11414_310 | 21.4 | 25635018 – 25635118 |
| 73817 | *IWB73817* | Tdurum_contig92819_647 | 33.5 | 51408290 – 51408190 |
| 6293 | *IWB6293* | BS00010811_51 | 40.4 | 77513390 – 77513490 |
| 17589 | *IWB17589* | D_F5XZDLF01EPDYG_190 | 47.4 | 214481663 – 214481443 |
| 7281 | *IWB7281* | BS00022836_51 | 57.2 | 534962109 – 534962009 |
| *73413* | *IWB73413* | Tdurum_contig77175_150 | 69.5 | 578514204 – 578514104 |
| *33879* | *IWB33879* | GENE-4268_101 | 74.4 | 585192054 – 585192154 |
| *56463* | *IWB56463* | RAC875_c3038_2741 | 100.7 | 605232192 – 605232292 |
| 71956 | *rwgsnp6* | Tdurum_contig49206_350 | 104.0 |  |
| 34398 | *rwgsnp7* | IAAV1529 | 104.8 | 615457318 – 615457118 |

| **Table S6.** List of 67 SSR markers used in bulked segregant analysis of a stem rust resistance gene derived from *T. turgidum* subsp. *polonicum* and size (bp) of the amplicon from each parent. | | | | |
| --- | --- | --- | --- | --- |
|  | Chromosome region | Marker | Rusty (bp) | *T. polonicum* (bp) |
| 1 | 1AL sub-metacentric | wmc9 | 188 | 195 |
| 2 | 1AL telomeric | barc158 | 262 | 273 |
| 3 | 1AS sub-metacentric | wmc24 | 141 | 172 |
| 4 | 1AS telomeric | gwm11 | 237 | 213 |
|  |  |  |  |  |
| 5 | 1BL sub-metacentric | cfd48 | 247 | 263 |
| 6 | 1BL telomeric | wmc830 | 302 | 308 |
| 7 | 1BS sub-metacentric | barc60 | 266 | 253 |
| 8 | 1BS telomeric | wmc818 | 180 | 216 |
|  |  |  |  |  |
| 9 | 2AL sub-metacentric | gwm372 | 338 | 361 |
| 10 | 2AL telomeric | gwm311 | 165 | 183 |
| 11 | 2AL telomeric | gwm382 | 134 | 152 |
| 12 | 2AS sub-metacentric | wmc602 | 188 | 142 |
| 13 | 2AS telomeric | gwm512 | 210 | 202 |
|  |  |  |  |  |
| 14 | 2BL sub-metacentric | cfd73 | 280 | 286 |
| 15 | 2BL telomeric | barc159 | 229 | 255 |
| 16 | 2BS sub-metacentric | wmc770 | 156 | 172 |
| 17 | 2BS sub-metacentric | barc124 | 263 | 268 |
| 18 | 2BS sub-metacentric | wmc597 | 264 | 260 |
| 19 | 2BS telomeric | wmc661 | 190 | 209 |
| 20 | 2BS telomeric | wmc489 | 255 | 282 |
| 21 | 2BS telomeric | gwm614 | 169, null | 144, 172 |
|  |  |  |  |  |
| 22 | 3AL sub-metacentric | wmc428 | 290 | 275 |
| 23 | 3AL telomeric | wmc594 | 164 | 176 |
| 24 | 3AS sub-metacentric | gwm674 | 186 | 184 |
| 25 | 3AS telomeric | gwm369 | 212 | 176 |
| 26 | 3AS telomeric | wmc532 | 195 | 180 |
|  |  |  |  |  |
| 27 | 3BL sub-metacentric | wmc787 | 180 | 162 |
| 28 | 3BL telomeric | gwm247 | 159 | 169 |
| 29 | 3BS sub-metacentric | gwm264 | 240 | 249 |
| 30 | 3BS telomeric | gwm493 | 168 | 181 |
|  |  |  |  |  |
| 31 | 4AL sub-metacentric | barc170 | 209 | 187 |
| 32 | 4AL telomeric | wmc313 | 219 | 213 |
| 33 | 4AS sub-metacentric | wmc173 | 263 | 272, 281 |
| 34 | 4AS telomeric | gwm397 | 205, 220 | 196, 210 |
| 35 | 4AS telomeric | gwm494 | 198, 206 | 198, 204 |
|  |  |  |  |  |
| 36 | 4BL sub-metacentric | wmc254 | 189 | 224 |
| 37 | 4BL telomeric | wmc617 | 242 | 236 |
| 38 | 4BS sub-metacentric | wmc652 | 186 | 180 |
| 39 | 4BS telomeric | barc109 | 266 | 260 |
|  |  |  |  |  |
| 40 | 5AL sub-metacentric | cfa2141 | 265 | 260 |
| 41 | 5AL sub-metacentric | cfa2155 | 265 | 233 |
| 42 | 5AL telomeric | wmc524 | 225 | 227 |
| 43 | 5AL telomeric | wmc727 | 105 | 103 |
| 44 | 5AS sub-metacentric | barc56 | 125 | 136 |
| 45 | 5AS telomeric | gwm443 | 205 | 188 |
|  |  |  |  |  |
| 46 | 5BL sub-metacentric | wmc537 | 205 | 193 |
| 47 | 5BL telomeric | wmc783 | 250 | 227 |
| 48 | 5BS sub-metacentric | wmc740 | 168 | 207, 213 |
| 49 | 5BS telomeric | barc240 | 277 | 291 |
|  |  |  |  |  |
| 50 | 6AL sub-metacentric | gwm169 | 208 | 218 |
| 51 | 6AL telomeric | wmc621 | 162 | 149 |
| 52 | 6AL telomeric | barc104 | null | 222 |
| 53 | 6AL telomeric | dupw167 | 248 | 266 |
| 54 | 6AS sub-metacentric | wmc753 | 298 | 309 |
| 55 | 6AS telomeric | gwm459 | 151 | 170 |
|  |  |  |  |  |
| 56 | 6BL sub-metacentric | wmc539 | 204 | 194 |
| 57 | 6BL telomeric | barc178 | 303 | 297 |
| 58 | 6BS sub-metacentric | gwm193 | 190 | 194 |
| 59 | 6BS telomeric | gwm132 | 131, 148 | 119, 143 |
|  |  |  |  |  |
| 60 | 7AL sub-metacentric | barc49 | 225 | 245 |
| 61 | 7AL telomeric | cfa2040 | 263 | 255 |
| 62 | 7AS sub-metacentric | barc154 | 247 | 255 |
| 63 | 7AS telomeric | gwm635 | 228 | 119 |
|  |  |  |  |  |
| 64 | 7BL sub-metacentric | wmc517 | 203 | 207 |
| 65 | 7BL telomeric | barc182 | 120 | 114 |
| 66 | 7BS sub-metacentric | gwm400 | 163 | 169 |
| 67 | 7BS telomeric | gwm569 | 154 | 170 |
