# Supplementary material for: Function and evolution of allelic variations of Sr13 conferring resistance to stem rust in tetraploid wheat (Triticum turgidum L.)
Source: Plant J. 2021 May 29;106(6):1674–91. doi: 10.1111/tpj.15263 (PMC8362117; doi:10.1111/tpj.15263)
Supplement: Supplementary file 11 — Table S7. Sequences of the Sr13 coding regions in eight lines tested (sequence of Langdon was downloaded from Zhang et al. (2017)). [file TPJ-106-1674-s006.docx]

**Table S7**. Sequences of the *Sr13* coding regions in eight lines tested (sequence of Langdon was downloaded from Zhang et al. (2017)).

1. Rusty-KL-B (Identical to Rusty-KL-C)

CTAGACTTGGCGCAAACTTTCCTGTTCGATCACTGGCCGTGCATTCGAGCTTTTAGGCCATGGAGGCAGCTCTGGTGACTGTGGCCACGGGGGTCCTCAAACCTGTCCTGGGGAAGCTGGCCACCCTGCTCGGCGACGAGTACAAGCGTTTTAAGGGTGTGCGCAAGGAGATCAGGTCTCTCACTCATGAACTCGCCGCCATGGAGGCTTTTCTCCTCAAGATGTCGGAGGAGGAGGAGGATCTCAATGTGCAGGATAAAGTTTGGATGAATGAGGTGCGGGAATTGTCCTATGACATGGAGGATGCCATCGATGACTTCATGCAAAGCGTTGGTGACAAAGAAGAAAAGCCGGATGGCTTCATTGACAAGATCAAGAGCTCGCTAGGAAAGTTGGGAAATATGAAGGCTCGTCATCGAATTGGCAAGGAGATACAGGATCTGAAGAAACAAATCATTGAGGTGGGCGACAGGAATGCAAGGTACAAGGGACGTGAGATCTTCTCCAAGGCCGTTAATGTGACCGTTGACCCTAGAGCTCTTGCTATCTTTGAGCATGCCTCAAAGCTCGTCGGAATTGATGAGCCCAAGGCTGAGCTGATCAAGTTGTTAACTGACAAGGATGGAGTTGCATCAACACAACAACAAGTGAAGATGGTCTCCATTGTTGGATCGGGAGGAATGGGCAAAACAACTCTTGCAAACCAAGTGTATCAAGAGCTGAAAGAGAAATTCAAGTGTAAGGCTTTCATTTCAGTGTCACGAAATCCAGATATGACAAATATCTTGAGAACCCTCCTTAGTGAAGTTGGGTGTCAAGATTATGCTGACACTGAAGCAGGGAGCATACAACAACTAATAAGAAAGATTACCGACTACCTAGCAGAAAAAAGGTACTATTATATTTCTTTAAATTTACTTCTCACCCATAGAAAGTTACATCAAGAATTCTCACATAGAAAAATACACTCCTAATTAAGAATCAAAATAAGCAATTATATACTTTTTAGGAGAAAATTAATTGCCAAATGTATGGAAGCACTTATTTGCATTACTTTACTAGTTAACTAGGTTGTTGCATTTGTAGGGAAAATAAGTCTTATATAAGTATGCATATAGACTAAAAGACAGCCTTTTCCGCAATAAAGAAATAGCATCAATCTTCAATCAAGCAAGTATGCTACACGATATGTACCACTCCAAGTGCTTAGAGCTCTTTTGCTCTTATATAGCTTATCTAGGAAAACATATTTATTTGATAAGCACATGTTTATATGAGTAGAAACAGTATATAGGTGTTTTCTGGCCATGTGGCCCTGTTTAAGTTGCATAGTACCCTAGAGCCGATACATTTATCTTTTGCATGTTGCCAATGAGAACACAGAAATTTCTCTTTCTTCTTATTTTGCTTGTACGCTTCGTTTTAACACATCACACTAACTAATACTACTAAAAAAATCATGTGCAGGTATATTATAGTGATTGACGACATATGGGATGTTAAAACATGGGACGTTATTAAGTGCGCATTCCCCATGACCAGATGCGGTGGTGTAATAATCACCACTACACGGCTGAGTGATGTTGCACGTTCGTGTCATTCATCAATCGGTGGCCATATTTATAATATAAGGCCTCTTAATATGGAGCACTCAAGACAACTATTCCATAGAAGATTATTCAGCTCCGAAGAAGATTGCCCTTCATCGCTCGTGAAAGTTTCTAATCAAATCTTGGAAAAATGTGATGGGTTGCCTTTGGCAATCATTGCTATAGCTGGTTTGTTGGCTAACACAGGAAGATCAGAGCATCTATGGAACCAAGTGAAAGATTCAATTGGTCGTGCACTTGAAAGGAATCCTAATGTCGAAGTAATGATAAAGATATTGTCACTTAGTTACTTTGATCTTCCTCCTCATCTGAAAACATGTCTCTTGTATCTCAGTATATTTCCGGAAGATTCTATTATTGAGAAGAAAACACTAATATCAAGATGGATTGCCGAAGGATTCATTCAGAAAGAAGGTATATATACTGCATATGAGGTAGGAGTGAGGTGTTTTAATGAGCTCATCAATAGGAGTTTGATCCAACCTGTGAAGAAAGACGATTATAGGGGGAAGAGTTGTCGAGTTCACGACATAATTCTTGATTTCATAGTATCCAAGTCCATTGAAGAGAACTTTGTTACTTTTGCTGGTGTCCCCAGTTTAACTACCGTGACACAAGGCAAAGTCCGCCGTCTCTCCATGCAAGTTGAAGGGAAAGGGGATTCTATCTTGCCAATGAGCCCGATATTGTCTCATGTCCGATCATTTAATGTGTTCAGGAATAGGGTGAATATCCATTCGACGATGGAGTTCAGACATTTGCGTGTTGTGGACTTTAATGACAGTCTACTGGAAAACCACCATCTTGCAAATGTAGGGAGGCTGCTTCAGCTAAGGTACCTCAGCATTTACATGACAGCAGTAAGCGAGCTCCCGGAACAAATCGGACACCTACAATGCTTAGAGATGTTGGACATCAGGTATACAATGGTGTCCGAGTTGCCAGCCAGTATTGTCAATCTTGGCAAACTGGCACACTTACTTCTT**C**GCTCAGAAGACACATGTGTTAAGTTTCCCGATGGAATTGCGAAGATGCAAGCACTAGAGACTTTGGATGAGGTTGACGCCAGCAAGCAGTCATATAACTTTCTGCAAGGGCTTGGTCGGCTAAAGAATCTGAGGAAGCTGCACATTGATTATCATGATGTTGCCCAGGAAGACAAGGAAGTTATTGCTTCTTCTCTCGGTAAACTATGCACACAAAACCTTTGTTCTCTAACTATGCGGGGGAATGATGATGATGACTTCTTGCTGAATACATGGTGCACTTCTCCGCCGCTTAACCTCCGAAAACTT**C**TCATATGGGGTTGTATATTCCCAAAGGTTCCGCATTGGGTAGGATCACTCGTCAACCTACAGAAGTTACGCTTGCATGTGGGGAAAGAAATCCGGCATGAAGATATCTGCATCCTTGGAGCCTTACCCGCTCTGCTCACTCTGGGTCTAAAAGGAATGCAAAAACAGCCTTCTTGTGAAGATGGAAGGCTGGCAGTTAGTGGTGAAGCTGGGTTCCGATGCCTGAGGAAGTTTAAATACTG**G**AGGTGGGGAGATAGGATGGATCTTATGTTTACGGCGAAATGTATGCCCAAGCTAGAAAAACTGAAGATTATATTTTACCGGCATGCCCAAGATGAGGCTCCCATCATTCCTGCTTTCGATTTCGGGATCGAAAACCTGTCCCGCCTCACTACTTTCAAATGTCACCTAGGTTGTAGGCCTATGGCAACGAGAACTTTTGATGCTGTAAAGGCTTCTCTGGACAGAGTAGTCAGAGCACATCCCAACCACCTTACTGTAATCTTCAGTTATCCTCTGCGTACGTGATTACCTTCTCAAGATTTATTATCTATACGACTAGTACAAACGTACTGACAACCTCTCTTAATTCCCTTGCCCTGTATTTTCAGGTA**C**GTCGGACATGACATATACATTCCATGATTGCTACATGCGGTCTCAAGACTAATCACTGCACTCCTGAGATCGGTTCGATCAGCATGTAAATTCCTCGACGAATGCGCCCAGGTTCTTGTTGACTTGTTTGTACGAAGGACGAATCCTGCCTCCTGCCTGCAGGTACATACCCAACTTTGTATTCGTCCAGATTTATTGTTTCTTTTACATTGCTCGCAGCGTCCTGCAGGTACATACCCAACTTTGTATTCGTCCAGATTTATTGTTTCTTTTACATTGCTCGCAGCGTCCTTAGATAGATAGCTGCTCTTTCAGAGAGTATG

1. CAT-A1 (**New resistance haplotype**)

CTAGACTTGGCGCAAACTTTCCTGTTCGATCACTGGCCGTGCATTCGAGCTTTTAGGCCATGGAGGCAGCTCTGGTGACTGTGGCCACGGGGGTCCTCAAACCTGTCCTGGGGAAGCTGGCCACCCTGCTCGGCGACGAGTACAAGCGTTTTAAGGGTGTGCGCAAGGAGATCAGGTCTCTCACTCATGAACTCGCCGCCATGGAGGCTTTTCTCCTCAAGATGTCGGAGGAGGAGGAGGATCTCAATGTGCAGGATAAAGTTTGGATGAATGAGGTGCGGGAATTGTCCTATGACATGGAGGATGCCATCGATGACTTCATGCAAAGCGTTGGTGACAAAGAAGAAAAGCCGGATGGCTTCATTGACAAGATCAAGAGCTCGCTAGGAAAGTTGGGAAATATGAAGGCTCGTCATCGAATTGGCAAGGAGATACAGGATCTGAAGAAACAAATCATTGAGGTGGGCGACAGGAATGCAAGGTACAAGGGACGTGAGATCTTCTCCAAGGCCGTTAATGTGACCGTTGACCCTAGAGCTCTTGCTATCTTTGAGCATGCCTCAAAGCTCGTCGGAATTGATGAGCCCAAGGCTGAGCTGATCAAGTTGTTAACTGACAAGGATGGAGTTGCATCAACACAACAACAAGTGAAGATGGTCTCCATTGTTGGATCGGGAGGAATGGGCAAAACAACTCTTGCAAACCAAGTGTATCAAGAGCTGAAAGAGAAATTCAAGTGTAAGGCTTTCATTTCAGTGTCACGAAATCCAGATATGACAAATATCTTGAGAACCCTCCTTAGTGAAGTTGGGTGTCAAGATTATGCTGACACTGAAGCAGGGAGCATACAACAACTAATAAGAAAGATTACCGACTACCTAGCAGAAAAAAGGTACTATTATATTTCTTTAAATTTACTTCTCACCCATAGAAAGTTACATCAAGAATTCTCACATAGAAAAATACACTCCTAATTAAGAATCAAAATAAGCAATTATATACTTTTTAGGAGAAAATTAATTGCCAAATGTATGGAAGCACTTATTTGCATTACTTTACTAGTTAACTAGGTTGTTGCATTTGTAGGGAAAATAAGTCTTATATAAGTATGCATATAGACTAAAAGACAGCCTTTTCCGCAATAAAGAAATAGCATCAATCTTCAATCAAGCAAGTATGCTACACGATATGTACCACTCCAAGTGCTTAGAGCTCTTTTGCTCTTATATAGCTTATCTAGGAAAACATATTTATTTGATAAGCACATGTTTATATGAGTAGAAACAGTATATAGGTGTTTTCTGGCCATGTGGCCCTGTTTAAGTTGCATAGTACCCTAGAGCCGATACATTTATCTTTTGCATGTTGCCAATGAGAACACAGAAATTTCTCTTTCTTCTTATTTTGCTTGTACGCTTCGTTTTAACACATCACACTAACTAATACTACTAAAAAAATCATGTGCAGGTATATTATAGTGATTGACGACATATGGGATGTTAAAACATGGGACGTTATTAAGTGCGCATTCCCCATGACCAGATGCGGTGGTGTAATAATCACCACTACACGGCTGAGTGATGTTGCACGTTCGTGTCATTCATCAATCGGTGGCCATATTTATAATATAAGGCCTCTTAATATGGAGCACTCAAGACAACTATTCCATAGAAGATTATTCAGCTCCGAAGAAGATTGCCCTTCATCGCTCGTGAAAGTTTCTAATCAAATCTTGGAAAAATGTGATGGGTTGCCTTTGGCAATCATTGCTATAGCTGGTTTGTTGGCTAACACAGGAAGATCAGAGCATCTATGGAACCAAGTGAAAGATTCAATTGGTCGTGCACTTGAAAGGAATCCTAATGTCGAAGTAATGATAAAGATATTGTCACTTAGTTACTTTGATCTTCCTCCTCATCTGAAAACATGTCTCTTGTATCTCAGTATATTTCCGGAAGATTCTATTATTGAGAAGAAAACACTAATATCAAGATGGATTGCCGAAGGATTCATTCAGAAAGAAGGTATATATACTGCATATGAGGTAGGAGTGAGGTGTTTTAATGAGCTCATCAATAGGAGTTTGATCCAACCTGTGAAGAAAGACGATTATAGGGGGAAGAGTTGTCGAGTTCACGACATAATTCTTGATTTCATAGTATCCAAGTCCATTGAAGAGAACTTTGTTACTTTTGCTGGTGTCCCCAGTTTAACTACCGTGACACAAGGCAAAGTCCGCCGTCTCTCCATGCAAGTTGAAGGGAAAGGGGATTCTATCTTGCCAATGAGCCCGATATTGTCTCATGTCCGATCATTTAATGTGTTCAGGAATAGGGTGAATATCCATTCGACGATGGAGTTCAGACATTTGCGTGTTGTGGACTTTAATGACAGTCTACTGGAAAACCACCATCTTGCAAATGTAGGGAGGCTGCTTCAGCTAAGGTACCTCAGCATTTACATGACAGCAGTAAGCGAGCTCCCGGAACAAATCGGACACCTACAATGCTTAGAGATGTTGGACATCAGGTATACAATGGTGTCCGAGTTGCCAGCCAGTATTGTCAATCTTGGCAAACTGGCACACTTACTTCTT**G**GCTCAGAAGACACATGTGTTAAGTTTCCCGATGGAATTGCGAAGATGCAAGCACTAGAGACTTTGGATGAGGTTGACGCCAGCAAGCAGTCATATAACTTTCTGCAAGGGCTTGGTCGGCTAAAGAATCTGAGGAAGCTGCACATTGATTATCATGATGTTGCCCAGGAAGACAAGGAAGTTATTGCTTCTTCTCTCGGTAAACTATGCACACAAAACCTTTGTTCTCTAACTATGCGGGGGAATGATGATGATGACTTCTTGCTGAATACATGGTGCACTTCTCCGCCGCTTAACCTCCGAAAACTT**G**TCATATGGGGTTGTATATTCCCAAAGGTTCCGCATTGGGTAGGATCACTCGTCAACCTACAGAAGTTACGCTTGCATGTGGGGAAAGAAATCCGGCATGAAGATATCTGCATCCTTGGAGCCTTACCCGCTCTGCTCACTCTGGGTCTAAAAGGAATGCAAAAACAGCCTTCTTGTGAAGATGGAAGGCTGGCAGTTAGTGGTGAAGCTGGGTTCCGATGCCTGAGGAAGTTTAAATACTG**G**AGGTGGGGAGATAGGATGGATCTTATGTTTACGGCGAAATGTATGCCCAAGCTAGAAAAACTGAAGATTATATTTTACCGGCATGCCCAAGATGAGGCTCCCATCATTCCTGCTTTCGATTTCGGGATCGAAAACCTGTCCCGCCTCACTACTTTCAAATGTCACCTAGGTTGTAGGCCTATGGCAACGAGAACTTTTGATGCTGTAAAGGCTTCTCTGGACAGAGTAGTCAGAGCACATCCCAACCACCTTACTGTAATCTTCAGTTATCCTCTGCGTACGTGATTACCTTCTCAAGATTTATTATCTATACGACTAGTACAAACGTACTGACAACCTCTCTTAATTCCCTTGCCCTGTATTTTCAGGTA**C**GTCGGACATGACATATACATTCCATGATTGCTACATGCGGTCTCAAGACTAATCACTGCACTCCTGAGATCGGTTCGATCAGCATGTAAATTCCTCGACGAATGCGCCCAGGTTCTTGTTGACTTGTTTGTACGAAGGACGAATCCTGCCTCCTGCCTGCAGGTACATACCCAACTTTGTATTCGTCCAGATTTATTGTTTCTTTTACATTGCTCGCAGCGTCCTGCAGGTACATACCCAACTTTGTATTCGTCCAGATTTATTGTTTCTTTTACATTGCTCGCAGCGTCCTTAGATAGATAGCTGCTCTTTCAGAGAGTATG

1. Rusty-14803 (Identical to PI 387696 and Iumillo-C2)

CTAGACTTGGCGCAAACTTTCCTGTTCGATCACTGGCCGTGCATTCGAGCTTTTAGGCCATGGAGGCAGCTCTGGTGACTGTGGCCACGGGGGTCCTCAAACCTGTCCTGGGGAAGCTGGCCACCCTGCTCGGCGACGAGTACAAGCGTTTTAAGGGTGTGCGCAAGGAGATCAGGTCTCTCACTCATGAACTCGCCGCCATGGAGGCTTTTCTCCTCAAGATGTCGGAGGAGGAGGAGGATCTCAATGTGCAGGATAAAGTTTGGATGAATGAGGTGCGGGAATTGTCCTATGACATGGAGGATGCCATCGATGACTTCATGCAAAGCGTTGGTGACAAAGAAGAAAAGCCGGATGGCTTCATTGACAAGATCAAGAGCTCGCTAGGAAAGTTGGGAAATATGAAGGCTCGTCATCGAATTGGCAAGGAGATACAGGATCTGAAGAAACAAATCATTGAGGTGGGCGACAGGAATGCAAGGTACAAGGGACGTGAGATCTTCTCCAAGGCCGTTAATGTGACCGTTGACCCTAGAGCTCTTGCTATCTTTGAGCATGCCTCAAAGCTCGTCGGAATTGATGAGCCCAAGGCTGAGCTGATCAAGTTGTTAACTGACAAGGATGGAGTTGCATCAACACAACAACAAGTGAAGATGGTCTCCATTGTTGGATCGGGAGGAATGGGCAAAACAACTCTTGCAAACCAAGTGTATCAAGAGCTGAAAGAGAAATTCAAGTGTAAGGCTTTCATTTCAGTGTCACGAAATCCAGATATGACAAATATCTTGAGAACCCTCCTTAGTGAAGTTGGGTGTCAAGATTATGCTGACACTGAAGCAGGGAGCATACAACAACTAATAAGAAAGATTACCGACTACCTAGCAGAAAAAAGGTACTATTATATTTCTTTAAATTTACTTCTCACCCATAGAAAGTTACATCAAGAATTCTCACATAGAAAAATACACTCCTAATTAAGAATCAAAATAAGCAATTATATACTTTTTAGGAGAAAATTAATTGCCAAATGTATGGAAGCACTTATTTGCATTACTTTACTAGTTAACTAGGTTGTTGCATTTGTAGGGAAAATAAGTCTTATATAAGTATGCATATAGACTAAAAGACAGCCTTTTCCGCAATAAAGAAATAGCATCAATCTTCAATCAAGCAAGTATGCTACACGATATGTACCACTCCAAGTGCTTAGAGCTCTTTTGCTCTTATATAGCTTATCTAGGAAAACATATTTATTTGATAAGCACATGTTTATATGAGTAGAAACAGTATATAGGTGTTTTCTGGCCATGTGGCCCTGTTTAAGTTGCATAGTACCCTAGAGCCGATACATTTATCTTTTGCATGTTGCCAATGAGAACACAGAAATTTCTCTTTCTTCTTATTTTGCTTGTACGCTTCGTTTTAACACATCACACTAACTAATACTACTAAAAAAATCATGTGCAGGTATATTATAGTGATTGACGACATATGGGATGTTAAAACATGGGACGTTATTAAGTGCGCATTCCCCATGACCAGATGCGGTGGTGTAATAATCACCACTACACGGCTGAGTGATGTTGCACGTTCGTGTCATTCATCAATCGGTGGCCATATTTATAATATAAGGCCTCTTAATATGGAGCACTCAAGACAACTATTCCATAGAAGATTATTCAGCTCCGAAGAAGATTGCCCTTCATCGCTCGTGAAAGTTTCTAATCAAATCTTGGAAAAATGTGATGGGTTGCCTTTGGCAATCATTGCTATAGCTGGTTTGTTGGCTAACACAGGAAGATCAGAGCATCTATGGAACCAAGTGAAAGATTCAATTGGTCGTGCACTTGAAAGGAATCCTAATGTCGAAGTAATGATAAAGATATTGTCACTTAGTTACTTTGATCTTCCTCCTCATCTGAAAACATGTCTCTTGTATCTCAGTATATTTCCGGAAGATTCTATTATTGAGAAGAAAACACTAATATCAAGATGGATTGCCGAAGGATTCATTCAGAAAGAAGGTATATATACTGCATATGAGGTAGGAGTGAGGTGTTTTAATGAGCTCATCAATAGGAGTTTGATCCAACCTGTGAAGAAAGACGATTATAGGGGGAAGAGTTGTCGAGTTCACGACATAATTCTTGATTTCATAGTATCCAAGTCCATTGAAGAGAACTTTGTTACTTTTGCTGGTGTCCCCAGTTTAACTACCGTGACACAAGGCAAAGTCCGCCGTCTCTCCATGCAAGTTGAAGGGAAAGGGGATTCTATCTTGCCAATGAGCCCGATATTGTCTCATGTCCGATCATTTAATGTGTTCAGGAATAGGGTGAATATCCATTCGACGATGGAGTTCAGACATTTGCGTGTTGTGGACTTTAATGACAGTCTACTGGAAAACCACCATCTTGCAAATGTAGGGAGGCTGCTTCAGCTAAGGTACCTCAGCATTTACATGACAGCAGTAAGCGAGCTCCCGGAACAAATCGGACACCTACAATGCTTAGAGATGTTGGACATCAGGTATACAATGGTGTCCGAGTTGCCAGCCAGTATTGTCAATCTTGGCAAACTGGCACACTTACTTCTT**G**GCTCAGAAGACACATGTGTTAAGTTTCCCGATGGAATTGCGAAGATGCAAGCACTAGAGACTTTGGATGAGGTTGACGCCAGCAAGCAGTCATATAACTTTCTGCAAGGGCTTGGTCGGCTAAAGAATCTGAGGAAGCTGCACATTGATTATCATGATGTTGCCCAGGAAGACAAGGAAGTTATTGCTTCTTCTCTCGGTAAACTATGCACACAAAACCTTTGTTCTCTAACTATGCGGGGGAATGATGATGATGACTTCTTGCTGAATACATGGTGCACTTCTCCGCCGCTTAACCTCCGAAAACTT**G**TCATATGGGGTTGTATATTCCCAAAGGTTCCGCATTGGGTAGGATCACTCGTCAACCTACAGAAGTTACGCTTGCATGTGGGGAAAGAAATCCGGCATGAAGATATCTGCATCCTTGGAGCCTTACCCGCTCTGCTCACTCTGGGTCTAAAAGGAATGCAAAAACAGCCTTCTTGTGAAGATGGAAGGCTGGCAGTTAGTGGTGAAGCTGGGTTCCGATGCCTGAGGAAGTTTAAATACTG**G**AGGTGGGGAGATAGGATGGATCTTATGTTTACGGCGAAATGTATGCCCAAGCTAGAAAAACTGAAGATTATATTTTACCGGCATGCCCAAGATGAGGCTCCCATCATTCCTGCTTTCGATTTCGGGATCGAAAACCTGTCCCGCCTCACTACTTTCAAATGTCACCTAGGTTGTAGGCCTATGGCAACGAGAACTTTTGATGCTGTAAAGGCTTCTCTGGACAGAGTAGTCAGAGCACATCCCAACCACCTTACTGTAATCTTCAGTTATCCTCTGCGTACGTGATTACCTTCTCAAGATTTATTATCTATACGACTAGTACAAACGTACTGACAACCTCTCTTAATTCCCTTGCCCTGTATTTTCAGGTA**A**GTCGGACATGACATATACATTCCATGATTGCTACATGCGGTCTCAAGACTAATCACTGCACTCCTGAGATCGGTTCGATCAGCATGTAAATTCCTCGACGAATGCGCCCAGGTTCTTGTTGACTTGTTTGTACGAAGGACGAATCCTGCCTCCTGCCTGCAGGTACATACCCAACTTTGTATTCGTCCAGATTTATTGTTTCTTTTACATTGCTCGCAGCGTCCTGCAGGTACATACCCAACTTTGTATTCGTCCAGATTTATTGTTTCTTTTACATTGCTCGCAGCGTCCTTAGATAGATAGCTGCTCTTTCAGAGAGTATG

1. PI 387696 (Identical to Rusty-14803 and Iumillo-C2))

CTAGACTTGGCGCAAACTTTCCTGTTCGATCACTGGCCGTGCATTCGAGCTTTTAGGCCATGGAGGCAGCTCTGGTGACTGTGGCCACGGGGGTCCTCAAACCTGTCCTGGGGAAGCTGGCCACCCTGCTCGGCGACGAGTACAAGCGTTTTAAGGGTGTGCGCAAGGAGATCAGGTCTCTCACTCATGAACTCGCCGCCATGGAGGCTTTTCTCCTCAAGATGTCGGAGGAGGAGGAGGATCTCAATGTGCAGGATAAAGTTTGGATGAATGAGGTGCGGGAATTGTCCTATGACATGGAGGATGCCATCGATGACTTCATGCAAAGCGTTGGTGACAAAGAAGAAAAGCCGGATGGCTTCATTGACAAGATCAAGAGCTCGCTAGGAAAGTTGGGAAATATGAAGGCTCGTCATCGAATTGGCAAGGAGATACAGGATCTGAAGAAACAAATCATTGAGGTGGGCGACAGGAATGCAAGGTACAAGGGACGTGAGATCTTCTCCAAGGCCGTTAATGTGACCGTTGACCCTAGAGCTCTTGCTATCTTTGAGCATGCCTCAAAGCTCGTCGGAATTGATGAGCCCAAGGCTGAGCTGATCAAGTTGTTAACTGACAAGGATGGAGTTGCATCAACACAACAACAAGTGAAGATGGTCTCCATTGTTGGATCGGGAGGAATGGGCAAAACAACTCTTGCAAACCAAGTGTATCAAGAGCTGAAAGAGAAATTCAAGTGTAAGGCTTTCATTTCAGTGTCACGAAATCCAGATATGACAAATATCTTGAGAACCCTCCTTAGTGAAGTTGGGTGTCAAGATTATGCTGACACTGAAGCAGGGAGCATACAACAACTAATAAGAAAGATTACCGACTACCTAGCAGAAAAAAGGTACTATTATATTTCTTTAAATTTACTTCTCACCCATAGAAAGTTACATCAAGAATTCTCACATAGAAAAATACACTCCTAATTAAGAATCAAAATAAGCAATTATATACTTTTTAGGAGAAAATTAATTGCCAAATGTATGGAAGCACTTATTTGCATTACTTTACTAGTTAACTAGGTTGTTGCATTTGTAGGGAAAATAAGTCTTATATAAGTATGCATATAGACTAAAAGACAGCCTTTTCCGCAATAAAGAAATAGCATCAATCTTCAATCAAGCAAGTATGCTACACGATATGTACCACTCCAAGTGCTTAGAGCTCTTTTGCTCTTATATAGCTTATCTAGGAAAACATATTTATTTGATAAGCACATGTTTATATGAGTAGAAACAGTATATAGGTGTTTTCTGGCCATGTGGCCCTGTTTAAGTTGCATAGTACCCTAGAGCCGATACATTTATCTTTTGCATGTTGCCAATGAGAACACAGAAATTTCTCTTTCTTCTTATTTTGCTTGTACGCTTCGTTTTAACACATCACACTAACTAATACTACTAAAAAAATCATGTGCAGGTATATTATAGTGATTGACGACATATGGGATGTTAAAACATGGGACGTTATTAAGTGCGCATTCCCCATGACCAGATGCGGTGGTGTAATAATCACCACTACACGGCTGAGTGATGTTGCACGTTCGTGTCATTCATCAATCGGTGGCCATATTTATAATATAAGGCCTCTTAATATGGAGCACTCAAGACAACTATTCCATAGAAGATTATTCAGCTCCGAAGAAGATTGCCCTTCATCGCTCGTGAAAGTTTCTAATCAAATCTTGGAAAAATGTGATGGGTTGCCTTTGGCAATCATTGCTATAGCTGGTTTGTTGGCTAACACAGGAAGATCAGAGCATCTATGGAACCAAGTGAAAGATTCAATTGGTCGTGCACTTGAAAGGAATCCTAATGTCGAAGTAATGATAAAGATATTGTCACTTAGTTACTTTGATCTTCCTCCTCATCTGAAAACATGTCTCTTGTATCTCAGTATATTTCCGGAAGATTCTATTATTGAGAAGAAAACACTAATATCAAGATGGATTGCCGAAGGATTCATTCAGAAAGAAGGTATATATACTGCATATGAGGTAGGAGTGAGGTGTTTTAATGAGCTCATCAATAGGAGTTTGATCCAACCTGTGAAGAAAGACGATTATAGGGGGAAGAGTTGTCGAGTTCACGACATAATTCTTGATTTCATAGTATCCAAGTCCATTGAAGAGAACTTTGTTACTTTTGCTGGTGTCCCCAGTTTAACTACCGTGACACAAGGCAAAGTCCGCCGTCTCTCCATGCAAGTTGAAGGGAAAGGGGATTCTATCTTGCCAATGAGCCCGATATTGTCTCATGTCCGATCATTTAATGTGTTCAGGAATAGGGTGAATATCCATTCGACGATGGAGTTCAGACATTTGCGTGTTGTGGACTTTAATGACAGTCTACTGGAAAACCACCATCTTGCAAATGTAGGGAGGCTGCTTCAGCTAAGGTACCTCAGCATTTACATGACAGCAGTAAGCGAGCTCCCGGAACAAATCGGACACCTACAATGCTTAGAGATGTTGGACATCAGGTATACAATGGTGTCCGAGTTGCCAGCCAGTATTGTCAATCTTGGCAAACTGGCACACTTACTTCTT**G**GCTCAGAAGACACATGTGTTAAGTTTCCCGATGGAATTGCGAAGATGCAAGCACTAGAGACTTTGGATGAGGTTGACGCCAGCAAGCAGTCATATAACTTTCTGCAAGGGCTTGGTCGGCTAAAGAATCTGAGGAAGCTGCACATTGATTATCATGATGTTGCCCAGGAAGACAAGGAAGTTATTGCTTCTTCTCTCGGTAAACTATGCACACAAAACCTTTGTTCTCTAACTATGCGGGGGAATGATGATGATGACTTCTTGCTGAATACATGGTGCACTTCTCCGCCGCTTAACCTCCGAAAACTT**G**TCATATGGGGTTGTATATTCCCAAAGGTTCCGCATTGGGTAGGATCACTCGTCAACCTACAGAAGTTACGCTTGCATGTGGGGAAAGAAATCCGGCATGAAGATATCTGCATCCTTGGAGCCTTACCCGCTCTGCTCACTCTGGGTCTAAAAGGAATGCAAAAACAGCCTTCTTGTGAAGATGGAAGGCTGGCAGTTAGTGGTGAAGCTGGGTTCCGATGCCTGAGGAAGTTTAAATACTG**G**AGGTGGGGAGATAGGATGGATCTTATGTTTACGGCGAAATGTATGCCCAAGCTAGAAAAACTGAAGATTATATTTTACCGGCATGCCCAAGATGAGGCTCCCATCATTCCTGCTTTCGATTTCGGGATCGAAAACCTGTCCCGCCTCACTACTTTCAAATGTCACCTAGGTTGTAGGCCTATGGCAACGAGAACTTTTGATGCTGTAAAGGCTTCTCTGGACAGAGTAGTCAGAGCACATCCCAACCACCTTACTGTAATCTTCAGTTATCCTCTGCGTACGTGATTACCTTCTCAAGATTTATTATCTATACGACTAGTACAAACGTACTGACAACCTCTCTTAATTCCCTTGCCCTGTATTTTCAGGTA**A**GTCGGACATGACATATACATTCCATGATTGCTACATGCGGTCTCAAGACTAATCACTGCACTCCTGAGATCGGTTCGATCAGCATGTAAATTCCTCGACGAATGCGCCCAGGTTCTTGTTGACTTGTTTGTACGAAGGACGAATCCTGCCTCCTGCCTGCAGGTACATACCCAACTTTGTATTCGTCCAGATTTATTGTTTCTTTTACATTGCTCGCAGCGTCCTGCAGGTACATACCCAACTTTGTATTCGTCCAGATTTATTGTTTCTTTTACATTGCTCGCAGCGTCCTTAGATAGATAGCTGCTCTTTCAGAGAGTATG

1. Rusty-ST464-C1 (Identical to Langdon and 8155-B2)

**CTAGACTTGGCGCAAACTTTCCTG**TTCGATCACTGGCCGTGCATTCGAGCTTTTAGGCCATGGAGGCAGCTCTGGTGACTGTGGCCACGGGGGTCCTCAAACCTGTCCTGGGGAAGCTGGCCACCCTGCTCGGCGACGAGTACAAGCGTTTTAAGGGTGTGCGCAAGGAGATCAGGTCTCTCACTCATGAACTCGCCGCCATGGAGGCTTTTCTCCTCAAGATGTCGGAGGAGGAGGAGGATCTCAATGTGCAGGATAAAGTTTGGATGAATGAGGTGCGGGAATTGTCCTATGACATGGAGGATGCCATCGATGACTTCATGCAAAGCGTTGGTGACAAAGAAGAAAAGCCGGATGGCTTCATTGACAAGATCAAGAGCTCGCTAGGAAAGTTGGGAAATATGAAGGCTCGTCATCGAATTGGCAAGGAGATACAGGATCTGAAGAAACAAATCATTGAGGTGGGCGACAGGAATGCAAGGTACAAGGGACGTGAGATCTTCTCCAAGGCCGTTAATGTGACCGTTGACCCTAGAGCTCTTGCTATCTTTGAGCATGCCTCAAAGCTCGTCGGAATTGATGAGCCCAAGGCTGAGCTGATCAAGTTGTTAACTGACAAGGATGGAGTTGCATCAACACAACAACAAGTGAAGATGGTCTCCATTGTTGGATCGGGAGGAATGGGCAAAACAACTCTTGCAAACCAAGTGTATCAAGAGCTGAAAGAGAAATTCAAGTGTAAGGCTTTCATTTCAGTGTCACGAAATCCAGATATGACAAATATCTTGAGAACCCTCCTTAGTGAAGTTGGGTGTCAAGATTATGCTGACACTGAAGCAGGGAGCATACAACAACTAATAAGAAAGATTACCGACTACCTAGCAGAAAAAAGGTACTATTATATTTCTTTAAATTTACTTCTCACCCATAGAAAGTTACATCAAGAATTCTCACATAGAAAAATACACTCCTAATTAAGAATCAAAATAAGCAATTATATACTTTTTAGGAGAAAATTAATTGCCAAATGTATGGAAGCACTTATTTGCATTACTTTACTAGTTAACTAGGTTGTTGCATTTGTAGGGAAAATAAGTCTTATATAAGTATGCATATAGACTAAAAGACAGCCTTTTCCGCAATAAAGAAATAGCATCAATCTTCAATCAAGCAAGTATGCTACACGATATGTACCACTCCAAGTGCTTAGAGCTCTTTTGCTCTTATATAGCTTATCTAGGAAAACATATTTATTTGATAAGCACATGTTTATATGAGTAGAAACAGTATATAGGTGTTTTCTGGCCATGTGGCCCTGTTTAAGTTGCATAGTACCCTAGAGCCGATACATTTATCTTTTGCATGTTGCCAATGAGAACACAGAAATTTCTCTTTCTTCTTATTTTGCTTGTACGCTTCGTTTTAACACATCACACTAACTAATACTACTAAAAAAATCATGTGCAGGTATATTATAGTGATTGACGACATATGGGATGTTAAAACATGGGACGTTATTAAGTGCGCATTCCCCATGACCAGATGCGGTGGTGTAATAATCACCACTACACGGCTGAGTGATGTTGCACGTTCGTGTCATTCATCAATCGGTGGCCATATTTATAATATAAGGCCTCTTAATATGGAGCACTCAAGACAACTATTCCATAGAAGATTATTCAGCTCCGAAGAAGATTGCCCTTCATCGCTCGTGAAAGTTTCTAATCAAATCTTGGAAAAATGTGATGGGTTGCCTTTGGCAATCATTGCTATAGCTGGTTTGTTGGCTAACACAGGAAGATCAGAGCATCTATGGAACCAAGTGAAAGATTCAATTGGTCGTGCACTTGAAAGGAATCCTAATGTCGAAGTAATGATAAAGATATTGTCACTTAGTTACTTTGATCTTCCTCCTCATCTGAAAACATGTCTCTTGTATCTCAGTATATTTCCGGAAGATTCTATTATTGAGAAGAAAACACTAATATCAAGATGGATTGCCGAAGGATTCATTCAGAAAGAAGGTATATATACTGCATATGAGGTAGGAGTGAGGTGTTTTAATGAGCTCATCAATAGGAGTTTGATCCAACCTGTGAAGAAAGACGATTATAGGGGGAAGAGTTGTCGAGTTCACGACATAATTCTTGATTTCATAGTATCCAAGTCCATTGAAGAGAACTTTGTTACTTTTGCTGGTGTCCCCAGTTTAACTACCGTGACACAAGGCAAAGTCCGCCGTCTCTCCATGCAAGTTGAAGGGAAAGGGGATTCTATCTTGCCAATGAGCCCGATATTGTCTCATGTCCGATCATTTAATGTGTTCAGGAATAGGGTGAATATCCATTCGACGATGGAGTTCAGACATTTGCGTGTTGTGGACTTTAATGACAGTCTACTGGAAAACCACCATCTTGCAAATGTAGGGAGGCTGCTTCAGCTAAGGTACCTCAGCATTTACATGACAGCAGTAAGCGAGCTCCCGGAACAAATCGGACACCTACAATGCTTAGAGATGTTGGACATCAGGTATACAATGGTGTCCGAGTTGCCAGCCAGTATTGTCAATCTTGGCAAACTGGCACACTTACTTCTT**G**GCTCAGAAGACACATGTGTTAAGTTTCCCGATGGAATTGCGAAGATGCAAGCACTAGAGACTTTGGATGAGGTTGACGCCAGCAAGCAGTCATATAACTTTCTGCAAGGGCTTGGTCGGCTAAAGAATCTGAGGAAGCTGCACATTGATTATCATGATGTTGCCCAGGAAGACAAGGAAGTTATTGCTTCTTCTCTCGGTAAACTATGCACACAAAACCTTTGTTCTCTAACTATGCGGGGGAATGATGATGATGACTTCTTGCTGAATACATGGTGCACTTCTCCGCCGCTTAACCTCCGAAAACTT**G**TCATATGGGGTTGTATATTCCCAAAGGTTCCGCATTGGGTAGGATCACTCGTCAACCTACAGAAGTTACGCTTGCATGTGGGGAAAGAAATCCGGCATGAAGATATCTGCATCCTTGGAGCCTTACCCGCTCTGCTCACTCTGGGTCTAAAAGGAATGCAAAAACAGCCTTCTTGTGAAGATGGAAGGCTGGCAGTTAGTGGTGAAGCTGGGTTCCGATGCCTGAGGAAGTTTAAATACTG**T**AGGTGGGGAGATAGGATGGATCTTATGTTTACGGCGAAATGTATGCCCAAGCTAGAAAAACTGAAGATTATATTTTACCGGCATGCCCAAGATGAGGCTCCCATCATTCCTGCTTTCGATTTCGGGATCGAAAACCTGTCCCGCCTCACTACTTTCAAATGTCACCTAGGTTGTAGGCCTATGGCAACGAGAACTTTTGATGCTGTAAAGGCTTCTCTGGACAGAGTAGTCAGAGCACATCCCAACCACCTTACTGTAATCTTCAGTTATCCTCTGCGTACGTGATTACCTTCTCAAGATTTATTATCTATACGACTAGTACAAACGTACTGACAACCTCTCTTAATTCCCTTGCCCTGTATTTTCAGGTA**C**GTCGGACATGACATATACATTCCATGATTGCTACATGCGGTCTCAAGACTAATCACTGCACTCCTGAGATCGGTTCGATCAGCATGTAAATTCCTCGACGAATGCGCCCAGGTTCTTGTTGACTTGTTTGTACGAAGGACGAATCCTGCCTCCTGCCTGCAGGTACATACCCAACTTTGTATTCGTCCAGATTTATTGTTTCTTTTACATTGCTCGCAGCGTCCTGCAGGTACATACCCAACTTTGTATTCGTCCAGATTTATTGTTTCTTTTACATTGCTCGCAGCGTCCTTAGATAGATAGCTGCTCTTTCAGAGAGTATG

1. Rusty-KL-C (Identical to Rusty-KL-B)

**CTAGACTTGGCGCAAACTTTCCTG**TTCGATCACTGGCCGTGCATTCGAGCTTTTAGGCCATGGAGGCAGCTCTGGTGACTGTGGCCACGGGGGTCCTCAAACCTGTCCTGGGGAAGCTGGCCACCCTGCTCGGCGACGAGTACAAGCGTTTTAAGGGTGTGCGCAAGGAGATCAGGTCTCTCACTCATGAACTCGCCGCCATGGAGGCTTTTCTCCTCAAGATGTCGGAGGAGGAGGAGGATCTCAATGTGCAGGATAAAGTTTGGATGAATGAGGTGCGGGAATTGTCCTATGACATGGAGGATGCCATCGATGACTTCATGCAAAGCGTTGGTGACAAAGAAGAAAAGCCGGATGGCTTCATTGACAAGATCAAGAGCTCGCTAGGAAAGTTGGGAAATATGAAGGCTCGTCATCGAATTGGCAAGGAGATACAGGATCTGAAGAAACAAATCATTGAGGTGGGCGACAGGAATGCAAGGTACAAGGGACGTGAGATCTTCTCCAAGGCCGTTAATGTGACCGTTGACCCTAGAGCTCTTGCTATCTTTGAGCATGCCTCAAAGCTCGTCGGAATTGATGAGCCCAAGGCTGAGCTGATCAAGTTGTTAACTGACAAGGATGGAGTTGCATCAACACAACAACAAGTGAAGATGGTCTCCATTGTTGGATCGGGAGGAATGGGCAAAACAACTCTTGCAAACCAAGTGTATCAAGAGCTGAAAGAGAAATTCAAGTGTAAGGCTTTCATTTCAGTGTCACGAAATCCAGATATGACAAATATCTTGAGAACCCTCCTTAGTGAAGTTGGGTGTCAAGATTATGCTGACACTGAAGCAGGGAGCATACAACAACTAATAAGAAAGATTACCGACTACCTAGCAGAAAAAAGGTACTATTATATTTCTTTAAATTTACTTCTCACCCATAGAAAGTTACATCAAGAATTCTCACATAGAAAAATACACTCCTAATTAAGAATCAAAATAAGCAATTATATACTTTTTAGGAGAAAATTAATTGCCAAATGTATGGAAGCACTTATTTGCATTACTTTACTAGTTAACTAGGTTGTTGCATTTGTAGGGAAAATAAGTCTTATATAAGTATGCATATAGACTAAAAGACAGCCTTTTCCGCAATAAAGAAATAGCATCAATCTTCAATCAAGCAAGTATGCTACACGATATGTACCACTCCAAGTGCTTAGAGCTCTTTTGCTCTTATATAGCTTATCTAGGAAAACATATTTATTTGATAAGCACATGTTTATATGAGTAGAAACAGTATATAGGTGTTTTCTGGCCATGTGGCCCTGTTTAAGTTGCATAGTACCCTAGAGCCGATACATTTATCTTTTGCATGTTGCCAATGAGAACACAGAAATTTCTCTTTCTTCTTATTTTGCTTGTACGCTTCGTTTTAACACATCACACTAACTAATACTACTAAAAAAATCATGTGCAGGTATATTATAGTGATTGACGACATATGGGATGTTAAAACATGGGACGTTATTAAGTGCGCATTCCCCATGACCAGATGCGGTGGTGTAATAATCACCACTACACGGCTGAGTGATGTTGCACGTTCGTGTCATTCATCAATCGGTGGCCATATTTATAATATAAGGCCTCTTAATATGGAGCACTCAAGACAACTATTCCATAGAAGATTATTCAGCTCCGAAGAAGATTGCCCTTCATCGCTCGTGAAAGTTTCTAATCAAATCTTGGAAAAATGTGATGGGTTGCCTTTGGCAATCATTGCTATAGCTGGTTTGTTGGCTAACACAGGAAGATCAGAGCATCTATGGAACCAAGTGAAAGATTCAATTGGTCGTGCACTTGAAAGGAATCCTAATGTCGAAGTAATGATAAAGATATTGTCACTTAGTTACTTTGATCTTCCTCCTCATCTGAAAACATGTCTCTTGTATCTCAGTATATTTCCGGAAGATTCTATTATTGAGAAGAAAACACTAATATCAAGATGGATTGCCGAAGGATTCATTCAGAAAGAAGGTATATATACTGCATATGAGGTAGGAGTGAGGTGTTTTAATGAGCTCATCAATAGGAGTTTGATCCAACCTGTGAAGAAAGACGATTATAGGGGGAAGAGTTGTCGAGTTCACGACATAATTCTTGATTTCATAGTATCCAAGTCCATTGAAGAGAACTTTGTTACTTTTGCTGGTGTCCCCAGTTTAACTACCGTGACACAAGGCAAAGTCCGCCGTCTCTCCATGCAAGTTGAAGGGAAAGGGGATTCTATCTTGCCAATGAGCCCGATATTGTCTCATGTCCGATCATTTAATGTGTTCAGGAATAGGGTGAATATCCATTCGACGATGGAGTTCAGACATTTGCGTGTTGTGGACTTTAATGACAGTCTACTGGAAAACCACCATCTTGCAAATGTAGGGAGGCTGCTTCAGCTAAGGTACCTCAGCATTTACATGACAGCAGTAAGCGAGCTCCCGGAACAAATCGGACACCTACAATGCTTAGAGATGTTGGACATCAGGTATACAATGGTGTCCGAGTTGCCAGCCAGTATTGTCAATCTTGGCAAACTGGCACACTTACTTCTT**C**GCTCAGAAGACACATGTGTTAAGTTTCCCGATGGAATTGCGAAGATGCAAGCACTAGAGACTTTGGATGAGGTTGACGCCAGCAAGCAGTCATATAACTTTCTGCAAGGGCTTGGTCGGCTAAAGAATCTGAGGAAGCTGCACATTGATTATCATGATGTTGCCCAGGAAGACAAGGAAGTTATTGCTTCTTCTCTCGGTAAACTATGCACACAAAACCTTTGTTCTCTAACTATGCGGGGGAATGATGATGATGACTTCTTGCTGAATACATGGTGCACTTCTCCGCCGCTTAACCTCCGAAAACTT**C**TCATATGGGGTTGTATATTCCCAAAGGTTCCGCATTGGGTAGGATCACTCGTCAACCTACAGAAGTTACGCTTGCATGTGGGGAAAGAAATCCGGCATGAAGATATCTGCATCCTTGGAGCCTTACCCGCTCTGCTCACTCTGGGTCTAAAAGGAATGCAAAAACAGCCTTCTTGTGAAGATGGAAGGCTGGCAGTTAGTGGTGAAGCTGGGTTCCGATGCCTGAGGAAGTTTAAATACTG**G**AGGTGGGGAGATAGGATGGATCTTATGTTTACGGCGAAATGTATGCCCAAGCTAGAAAAACTGAAGATTATATTTTACCGGCATGCCCAAGATGAGGCTCCCATCATTCCTGCTTTCGATTTCGGGATCGAAAACCTGTCCCGCCTCACTACTTTCAAATGTCACCTAGGTTGTAGGCCTATGGCAACGAGAACTTTTGATGCTGTAAAGGCTTCTCTGGACAGAGTAGTCAGAGCACATCCCAACCACCTTACTGTAATCTTCAGTTATCCTCTGCGTACGTGATTACCTTCTCAAGATTTATTATCTATACGACTAGTACAAACGTACTGACAACCTCTCTTAATTCCCTTGCCCTGTATTTTCAGGTA**C**GTCGGACATGACATATACATTCCATGATTGCTACATGCGGTCTCAAGACTAATCACTGCACTCCTGAGATCGGTTCGATCAGCATGTAAATTCCTCGACGAATGCGCCCAGGTTCTTGTTGACTTGTTTGTACGAAGGACGAATCCTGCCTCCTGCCTGCAGGTACATACCCAACTTTGTATTCGTCCAGATTTATTGTTTCTTTTACATTGCTCGCAGCGTCCTGCAGGTACATACCCAACTTTGTATTCGTCCAGATTTATTGTTTCTTTTACATTGCTCGCAGCGTCCTTAGATAGATAGCTGCTCTTTCAGAGAGTATG

1. 8155-B2 (Identical to Langdon and Rusty-ST464)

**CTAGACTTGGCGCAAACTTTCCTG**TTCGATCACTGGCCGTGCATTCGAGCTTTTAGGCCATGGAGGCAGCTCTGGTGACTGTGGCCACGGGGGTCCTCAAACCTGTCCTGGGGAAGCTGGCCACCCTGCTCGGCGACGAGTACAAGCGTTTTAAGGGTGTGCGCAAGGAGATCAGGTCTCTCACTCATGAACTCGCCGCCATGGAGGCTTTTCTCCTCAAGATGTCGGAGGAGGAGGAGGATCTCAATGTGCAGGATAAAGTTTGGATGAATGAGGTGCGGGAATTGTCCTATGACATGGAGGATGCCATCGATGACTTCATGCAAAGCGTTGGTGACAAAGAAGAAAAGCCGGATGGCTTCATTGACAAGATCAAGAGCTCGCTAGGAAAGTTGGGAAATATGAAGGCTCGTCATCGAATTGGCAAGGAGATACAGGATCTGAAGAAACAAATCATTGAGGTGGGCGACAGGAATGCAAGGTACAAGGGACGTGAGATCTTCTCCAAGGCCGTTAATGTGACCGTTGACCCTAGAGCTCTTGCTATCTTTGAGCATGCCTCAAAGCTCGTCGGAATTGATGAGCCCAAGGCTGAGCTGATCAAGTTGTTAACTGACAAGGATGGAGTTGCATCAACACAACAACAAGTGAAGATGGTCTCCATTGTTGGATCGGGAGGAATGGGCAAAACAACTCTTGCAAACCAAGTGTATCAAGAGCTGAAAGAGAAATTCAAGTGTAAGGCTTTCATTTCAGTGTCACGAAATCCAGATATGACAAATATCTTGAGAACCCTCCTTAGTGAAGTTGGGTGTCAAGATTATGCTGACACTGAAGCAGGGAGCATACAACAACTAATAAGAAAGATTACCGACTACCTAGCAGAAAAAAGGTACTATTATATTTCTTTAAATTTACTTCTCACCCATAGAAAGTTACATCAAGAATTCTCACATAGAAAAATACACTCCTAATTAAGAATCAAAATAAGCAATTATATACTTTTTAGGAGAAAATTAATTGCCAAATGTATGGAAGCACTTATTTGCATTACTTTACTAGTTAACTAGGTTGTTGCATTTGTAGGGAAAATAAGTCTTATATAAGTATGCATATAGACTAAAAGACAGCCTTTTCCGCAATAAAGAAATAGCATCAATCTTCAATCAAGCAAGTATGCTACACGATATGTACCACTCCAAGTGCTTAGAGCTCTTTTGCTCTTATATAGCTTATCTAGGAAAACATATTTATTTGATAAGCACATGTTTATATGAGTAGAAACAGTATATAGGTGTTTTCTGGCCATGTGGCCCTGTTTAAGTTGCATAGTACCCTAGAGCCGATACATTTATCTTTTGCATGTTGCCAATGAGAACACAGAAATTTCTCTTTCTTCTTATTTTGCTTGTACGCTTCGTTTTAACACATCACACTAACTAATACTACTAAAAAAATCATGTGCAGGTATATTATAGTGATTGACGACATATGGGATGTTAAAACATGGGACGTTATTAAGTGCGCATTCCCCATGACCAGATGCGGTGGTGTAATAATCACCACTACACGGCTGAGTGATGTTGCACGTTCGTGTCATTCATCAATCGGTGGCCATATTTATAATATAAGGCCTCTTAATATGGAGCACTCAAGACAACTATTCCATAGAAGATTATTCAGCTCCGAAGAAGATTGCCCTTCATCGCTCGTGAAAGTTTCTAATCAAATCTTGGAAAAATGTGATGGGTTGCCTTTGGCAATCATTGCTATAGCTGGTTTGTTGGCTAACACAGGAAGATCAGAGCATCTATGGAACCAAGTGAAAGATTCAATTGGTCGTGCACTTGAAAGGAATCCTAATGTCGAAGTAATGATAAAGATATTGTCACTTAGTTACTTTGATCTTCCTCCTCATCTGAAAACATGTCTCTTGTATCTCAGTATATTTCCGGAAGATTCTATTATTGAGAAGAAAACACTAATATCAAGATGGATTGCCGAAGGATTCATTCAGAAAGAAGGTATATATACTGCATATGAGGTAGGAGTGAGGTGTTTTAATGAGCTCATCAATAGGAGTTTGATCCAACCTGTGAAGAAAGACGATTATAGGGGGAAGAGTTGTCGAGTTCACGACATAATTCTTGATTTCATAGTATCCAAGTCCATTGAAGAGAACTTTGTTACTTTTGCTGGTGTCCCCAGTTTAACTACCGTGACACAAGGCAAAGTCCGCCGTCTCTCCATGCAAGTTGAAGGGAAAGGGGATTCTATCTTGCCAATGAGCCCGATATTGTCTCATGTCCGATCATTTAATGTGTTCAGGAATAGGGTGAATATCCATTCGACGATGGAGTTCAGACATTTGCGTGTTGTGGACTTTAATGACAGTCTACTGGAAAACCACCATCTTGCAAATGTAGGGAGGCTGCTTCAGCTAAGGTACCTCAGCATTTACATGACAGCAGTAAGCGAGCTCCCGGAACAAATCGGACACCTACAATGCTTAGAGATGTTGGACATCAGGTATACAATGGTGTCCGAGTTGCCAGCCAGTATTGTCAATCTTGGCAAACTGGCACACTTACTTCTT**G**GCTCAGAAGACACATGTGTTAAGTTTCCCGATGGAATTGCGAAGATGCAAGCACTAGAGACTTTGGATGAGGTTGACGCCAGCAAGCAGTCATATAACTTTCTGCAAGGGCTTGGTCGGCTAAAGAATCTGAGGAAGCTGCACATTGATTATCATGATGTTGCCCAGGAAGACAAGGAAGTTATTGCTTCTTCTCTCGGTAAACTATGCACACAAAACCTTTGTTCTCTAACTATGCGGGGGAATGATGATGATGACTTCTTGCTGAATACATGGTGCACTTCTCCGCCGCTTAACCTCCGAAAACTT**G**TCATATGGGGTTGTATATTCCCAAAGGTTCCGCATTGGGTAGGATCACTCGTCAACCTACAGAAGTTACGCTTGCATGTGGGGAAAGAAATCCGGCATGAAGATATCTGCATCCTTGGAGCCTTACCCGCTCTGCTCACTCTGGGTCTAAAAGGAATGCAAAAACAGCCTTCTTGTGAAGATGGAAGGCTGGCAGTTAGTGGTGAAGCTGGGTTCCGATGCCTGAGGAAGTTTAAATACTG**T**AGGTGGGGAGATAGGATGGATCTTATGTTTACGGCGAAATGTATGCCCAAGCTAGAAAAACTGAAGATTATATTTTACCGGCATGCCCAAGATGAGGCTCCCATCATTCCTGCTTTCGATTTCGGGATCGAAAACCTGTCCCGCCTCACTACTTTCAAATGTCACCTAGGTTGTAGGCCTATGGCAACGAGAACTTTTGATGCTGTAAAGGCTTCTCTGGACAGAGTAGTCAGAGCACATCCCAACCACCTTACTGTAATCTTCAGTTATCCTCTGCGTACGTGATTACCTTCTCAAGATTTATTATCTATACGACTAGTACAAACGTACTGACAACCTCTCTTAATTCCCTTGCCCTGTATTTTCAGGTA**C**GTCGGACATGACATATACATTCCATGATTGCTACATGCGGTCTCAAGACTAATCACTGCACTCCTGAGATCGGTTCGATCAGCATGTAAATTCCTCGACGAATGCGCCCAGGTTCTTGTTGACTTGTTTGTACGAAGGACGAATCCTGCCTCCTGCCTGCAGGTACATACCCAACTTTGTATTCGTCCAGATTTATTGTTTCTTTTACATTGCTCGCAGCGTCCTGCAGGTACATACCCAACTTTGTATTCGTCCAGATTTATTGTTTCTTTTACATTGCTCGCAGCGTCCTTAGATAGATAGCTGCTCTTTCAGAGAGTATG

1. Im-C2 (Iumillo) (Identical to Rusty-14803 and PI 387696)

**CTAGACTTGGCGCAAACTTTCCTG**TTCGATCACTGGCCGTGCATTCGAGCTTTTAGGCCATGGAGGCAGCTCTGGTGACTGTGGCCACGGGGGTCCTCAAACCTGTCCTGGGGAAGCTGGCCACCCTGCTCGGCGACGAGTACAAGCGTTTTAAGGGTGTGCGCAAGGAGATCAGGTCTCTCACTCATGAACTCGCCGCCATGGAGGCTTTTCTCCTCAAGATGTCGGAGGAGGAGGAGGATCTCAATGTGCAGGATAAAGTTTGGATGAATGAGGTGCGGGAATTGTCCTATGACATGGAGGATGCCATCGATGACTTCATGCAAAGCGTTGGTGACAAAGAAGAAAAGCCGGATGGCTTCATTGACAAGATCAAGAGCTCGCTAGGAAAGTTGGGAAATATGAAGGCTCGTCATCGAATTGGCAAGGAGATACAGGATCTGAAGAAACAAATCATTGAGGTGGGCGACAGGAATGCAAGGTACAAGGGACGTGAGATCTTCTCCAAGGCCGTTAATGTGACCGTTGACCCTAGAGCTCTTGCTATCTTTGAGCATGCCTCAAAGCTCGTCGGAATTGATGAGCCCAAGGCTGAGCTGATCAAGTTGTTAACTGACAAGGATGGAGTTGCATCAACACAACAACAAGTGAAGATGGTCTCCATTGTTGGATCGGGAGGAATGGGCAAAACAACTCTTGCAAACCAAGTGTATCAAGAGCTGAAAGAGAAATTCAAGTGTAAGGCTTTCATTTCAGTGTCACGAAATCCAGATATGACAAATATCTTGAGAACCCTCCTTAGTGAAGTTGGGTGTCAAGATTATGCTGACACTGAAGCAGGGAGCATACAACAACTAATAAGAAAGATTACCGACTACCTAGCAGAAAAAAGGTACTATTATATTTCTTTAAATTTACTTCTCACCCATAGAAAGTTACATCAAGAATTCTCACATAGAAAAATACACTCCTAATTAAGAATCAAAATAAGCAATTATATACTTTTTAGGAGAAAATTAATTGCCAAATGTATGGAAGCACTTATTTGCATTACTTTACTAGTTAACTAGGTTGTTGCATTTGTAGGGAAAATAAGTCTTATATAAGTATGCATATAGACTAAAAGACAGCCTTTTCCGCAATAAAGAAATAGCATCAATCTTCAATCAAGCAAGTATGCTACACGATATGTACCACTCCAAGTGCTTAGAGCTCTTTTGCTCTTATATAGCTTATCTAGGAAAACATATTTATTTGATAAGCACATGTTTATATGAGTAGAAACAGTATATAGGTGTTTTCTGGCCATGTGGCCCTGTTTAAGTTGCATAGTACCCTAGAGCCGATACATTTATCTTTTGCATGTTGCCAATGAGAACACAGAAATTTCTCTTTCTTCTTATTTTGCTTGTACGCTTCGTTTTAACACATCACACTAACTAATACTACTAAAAAAATCATGTGCAGGTATATTATAGTGATTGACGACATATGGGATGTTAAAACATGGGACGTTATTAAGTGCGCATTCCCCATGACCAGATGCGGTGGTGTAATAATCACCACTACACGGCTGAGTGATGTTGCACGTTCGTGTCATTCATCAATCGGTGGCCATATTTATAATATAAGGCCTCTTAATATGGAGCACTCAAGACAACTATTCCATAGAAGATTATTCAGCTCCGAAGAAGATTGCCCTTCATCGCTCGTGAAAGTTTCTAATCAAATCTTGGAAAAATGTGATGGGTTGCCTTTGGCAATCATTGCTATAGCTGGTTTGTTGGCTAACACAGGAAGATCAGAGCATCTATGGAACCAAGTGAAAGATTCAATTGGTCGTGCACTTGAAAGGAATCCTAATGTCGAAGTAATGATAAAGATATTGTCACTTAGTTACTTTGATCTTCCTCCTCATCTGAAAACATGTCTCTTGTATCTCAGTATATTTCCGGAAGATTCTATTATTGAGAAGAAAACACTAATATCAAGATGGATTGCCGAAGGATTCATTCAGAAAGAAGGTATATATACTGCATATGAGGTAGGAGTGAGGTGTTTTAATGAGCTCATCAATAGGAGTTTGATCCAACCTGTGAAGAAAGACGATTATAGGGGGAAGAGTTGTCGAGTTCACGACATAATTCTTGATTTCATAGTATCCAAGTCCATTGAAGAGAACTTTGTTACTTTTGCTGGTGTCCCCAGTTTAACTACCGTGACACAAGGCAAAGTCCGCCGTCTCTCCATGCAAGTTGAAGGGAAAGGGGATTCTATCTTGCCAATGAGCCCGATATTGTCTCATGTCCGATCATTTAATGTGTTCAGGAATAGGGTGAATATCCATTCGACGATGGAGTTCAGACATTTGCGTGTTGTGGACTTTAATGACAGTCTACTGGAAAACCACCATCTTGCAAATGTAGGGAGGCTGCTTCAGCTAAGGTACCTCAGCATTTACATGACAGCAGTAAGCGAGCTCCCGGAACAAATCGGACACCTACAATGCTTAGAGATGTTGGACATCAGGTATACAATGGTGTCCGAGTTGCCAGCCAGTATTGTCAATCTTGGCAAACTGGCACACTTACTTCTT**G**GCTCAGAAGACACATGTGTTAAGTTTCCCGATGGAATTGCGAAGATGCAAGCACTAGAGACTTTGGATGAGGTTGACGCCAGCAAGCAGTCATATAACTTTCTGCAAGGGCTTGGTCGGCTAAAGAATCTGAGGAAGCTGCACATTGATTATCATGATGTTGCCCAGGAAGACAAGGAAGTTATTGCTTCTTCTCTCGGTAAACTATGCACACAAAACCTTTGTTCTCTAACTATGCGGGGGAATGATGATGATGACTTCTTGCTGAATACATGGTGCACTTCTCCGCCGCTTAACCTCCGAAAACTT**G**TCATATGGGGTTGTATATTCCCAAAGGTTCCGCATTGGGTAGGATCACTCGTCAACCTACAGAAGTTACGCTTGCATGTGGGGAAAGAAATCCGGCATGAAGATATCTGCATCCTTGGAGCCTTACCCGCTCTGCTCACTCTGGGTCTAAAAGGAATGCAAAAACAGCCTTCTTGTGAAGATGGAAGGCTGGCAGTTAGTGGTGAAGCTGGGTTCCGATGCCTGAGGAAGTTTAAATACTG**G**AGGTGGGGAGATAGGATGGATCTTATGTTTACGGCGAAATGTATGCCCAAGCTAGAAAAACTGAAGATTATATTTTACCGGCATGCCCAAGATGAGGCTCCCATCATTCCTGCTTTCGATTTCGGGATCGAAAACCTGTCCCGCCTCACTACTTTCAAATGTCACCTAGGTTGTAGGCCTATGGCAACGAGAACTTTTGATGCTGTAAAGGCTTCTCTGGACAGAGTAGTCAGAGCACATCCCAACCACCTTACTGTAATCTTCAGTTATCCTCTGCGTACGTGATTACCTTCTCAAGATTTATTATCTATACGACTAGTACAAACGTACTGACAACCTCTCTTAATTCCCTTGCCCTGTATTTTCAGGTA**A**GTCGGACATGACATATACATTCCATGATTGCTACATGCGGTCTCAAGACTAATCACTGCACTCCTGAGATCGGTTCGATCAGCATGTAAATTCCTCGACGAATGCGCCCAGGTTCTTGTTGACTTGTTTGTACGAAGGACGAATCCTGCCTCCTGCCTGCAGGTACATACCCAACTTTGTATTCGTCCAGATTTATTGTTTCTTTTACATTGCTCGCAGCGTCCTGCAGGTACATACCCAACTTTGTATTCGTCCAGATTTATTGTTTCTTTTACATTGCTCGCAGCGTCCTTAGATAGATAGCTGCTCTTTCAGAGAGTATG

Langdon

**CTAGACTTGGCGCAAACTTTCCTG**TTCGATCACTGGCCGTGCATTCGAGCTTTTAGGCCATGGAGGCAGCTCTGGTGACTGTGGCCACGGGGGTCCTCAAACCTGTCCTGGGGAAGCTGGCCACCCTGCTCGGCGACGAGTACAAGCGTTTTAAGGGTGTGCGCAAGGAGATCAGGTCTCTCACTCATGAACTCGCCGCCATGGAGGCTTTTCTCCTCAAGATGTCGGAGGAGGAGGAGGATCTCAATGTGCAGGATAAAGTTTGGATGAATGAGGTGCGGGAATTGTCCTATGACATGGAGGATGCCATCGATGACTTCATGCAAAGCGTTGGTGACAAAGAAGAAAAGCCGGATGGCTTCATTGACAAGATCAAGAGCTCGCTAGGAAAGTTGGGAAATATGAAGGCTCGTCATCGAATTGGCAAGGAGATACAGGATCTGAAGAAACAAATCATTGAGGTGGGCGACAGGAATGCAAGGTACAAGGGACGTGAGATCTTCTCCAAGGCCGTTAATGTGACCGTTGACCCTAGAGCTCTTGCTATCTTTGAGCATGCCTCAAAGCTCGTCGGAATTGATGAGCCCAAGGCTGAGCTGATCAAGTTGTTAACTGACAAGGATGGAGTTGCATCAACACAACAACAAGTGAAGATGGTCTCCATTGTTGGATCGGGAGGAATGGGCAAAACAACTCTTGCAAACCAAGTGTATCAAGAGCTGAAAGAGAAATTCAAGTGTAAGGCTTTCATTTCAGTGTCACGAAATCCAGATATGACAAATATCTTGAGAACCCTCCTTAGTGAAGTTGGGTGTCAAGATTATGCTGACACTGAAGCAGGGAGCATACAACAACTAATAAGAAAGATTACCGACTACCTAGCAGAAAAAAGGTACTATTATATTTCTTTAAATTTACTTCTCACCCATAGAAAGTTACATCAAGAATTCTCACATAGAAAAATACACTCCTAATTAAGAATCAAAATAAGCAATTATATACTTTTTAGGAGAAAATTAATTGCCAAATGTATGGAAGCACTTATTTGCATTACTTTACTAGTTAACTAGGTTGTTGCATTTGTAGGGAAAATAAGTCTTATATAAGTATGCATATAGACTAAAAGACAGCCTTTTCCGCAATAAAGAAATAGCATCAATCTTCAATCAAGCAAGTATGCTACACGATATGTACCACTCCAAGTGCTTAGAGCTCTTTTGCTCTTATATAGCTTATCTAGGAAAACATATTTATTTGATAAGCACATGTTTATATGAGTAGAAACAGTATATAGGTGTTTTCTGGCCATGTGGCCCTGTTTAAGTTGCATAGTACCCTAGAGCCGATACATTTATCTTTTGCATGTTGCCAATGAGAACACAGAAATTTCTCTTTCTTCTTATTTTGCTTGT**ACGCTTCGTTTTAACACATCAC**ACTAACTAATACTACTAAAAAAATCATGTGCAGGTATATTATAGTGATTGACGACATATGGGATGTTAAAACATGGGACGTTATTAAGTGCGCATTCCCCATGACCAGATGCGGTGGTGTAATAATCACCACTACACGGCTGAGTGATGTTGCACGTTCGTGTCATTCATCAATCGGTGGCCATATTTATAATATAAGGCCTCTTAATATGGAGCACTCAAGACAACTATTCCATAGAAGATTATTCAGCTCCGAAGAAGATTGCCCTTCATCGCTCGTGAAAGTTTCTAATCAAATCTTGGAAAAATGTGATGGGTTGCCTTTGGCAATCATTGCTATAGCTGGTTTGTTGGCTAACACAGGAAGATCAGAGCATCTATGGAACCAAGTGAAAGATTCAATTGGTCGTGCACTTGAAAGGAATCCTAATGTCGAAGTAATGATAAAGATATTGTCACTTAGTTACTTTGATCTTCCTCCTCATCTGAAAACATGTCTCTTGTATCTCAGTATATTTCCGGAAGATTCTATTATTGAGAAGAAAACACTAATATCAAGATGGATTGCCGAAGGATTCATTCAGAAAGAAGGTATATATACTGCATATGAGGTAGGAGTGAGGTGTTTTAATGAGCTCATCAATAGGAGTTTGATCCAACCTGTGAAGAAAGACGATTATAGGGGGAAGAGTTGTCGAGTTCACGACATAATTCTTGATTTCATAGTATCCAAGTCCATTGAAGAGAACTTTGTTACTTTTGCTGGTGTCCCCAGTTTAACTACCGTGACACAAGGCAAAGTCCGCCGTCTCTCCATGCAAGTTGAAGGGAAAGGGGATTCTATCTTGCCAATGAGCCCGATATTGTCTCATGTCCGATCATTTAATGTGTTCAGGAATAGGGTGAATATCCATTCGACGATGGAGTTCAGACATTTGCGTGTTGTGGACTTTAAT**GACAGTCTACTGGAAAACCACCATCTTG**CAAATGTAGGGAGGCTGCTTCAGCTAAGGTACCTCAGCATTTACATGACAGCAGTAAGCGAGCTCCCGGAACAAATCGGACACCTACAATGCTTAGAGATGTTGGACATCAGGTATACAATGGTGTCCGAGTTGCCAGCCAGTATTGTCAATCTTGGCAAACTGGCACACTTACTTCTT**G**GCTCAGAAGACACATGTGTTAAGTTTCCCGATGGAATTGCGAAGATGCAAGCACTAGAGACTTTGGATGAGGTTGACGCCAGCAAGCAGTCATATAACTTTCTGCAAGGGCTTGGTCGGCTAAAGAATCTGAGGAAGCTGCACATTGATTATCATGATGTTGCCCAGGAAGACAAGGAAGTTATTGCTTCTTCTCTCGGTAAACTATGCACACAAAACCTTTGTTCTCTAACTATGCGGGGGAATGATGATGATGACTTCTTGCTGAATACATGGTGCACTTCTCCGCCGCTTAACCTCCGAAAACTT**G**TCATATGGGGTTGTATATTCCCAAAGGTTCCGCATTGGGTAGGATCACTCGTCAACCTACAGAAGTTACGCTTGCATGTGGGGAAAGAAATCCGGCATGAAGATATCTGCATCCTTGGAGCCTTACCCGCTCTGCTCACTCTGGGTCTAAAAGGAATGCAAAAACAGCCTTCTTGTGAAGATGGAAGGCTGGCAGTTAGTGGTGAAGCTGGGTTCCGATGCCTGAGGAAGTTTAAATACTG**T**AGGTGGGGAGATAGGATGGATCTTATGTTTACGGCGAAATGTATGCCCAAGCTAGAAAAACTGAAGATTATATTTTACCGGCATGCCCAAGATGAGGCTCCCATCATTCCTGCTTTCGATTTCGGGATCGAAAACCTGTCCCGCCTCACTACTTTCAAATGTCACCTAGGTTGTAGGCCTATGGCAACGAGAACTTTTGATGCTGTAAAGGCTTCTCTGGACAGAGTAGTCAGAGCACATCCCAACCACCTTACTGTAATCTTCAGTTATCCTCTGCGTACGTGATTACCTTCTCAAGATTTATTATCTATACGACTAGTACAAACGTACTGACAACCTCTCTTAATTCCCTTGCCCTGTATTTTCAGGTA**C**GTCGGACATGACATATACATTCCATGATTGCTACATGCGGTCTCAAGACTAATCACTGCACTCCTGAGATCGGTTCGATCAGCATGTAAATTCCTCGACGAATGCGCCCAGGTTCTTGTTGACTT**GTTTGTA****CGAAGGACGAATCCTGCCTC**CTGCCTGCAGGTACATACCCAACTTTGTATTCGTCCAGATTTATTGTTTCTTTTACATTGCTCGCAGCGTCCTGCAGGTACATACCCAACTTTGTATTCGTCCAGATTTATTGTTTCTTTTACATTGCTCGCAGCGTCCTTAGATAGATAGCTGCTCTTTCAGAGAGTATG
