# Supplementary material for: Function and evolution of allelic variations of Sr13 conferring resistance to stem rust in tetraploid wheat (Triticum turgidum L.)
Source: Plant J. 2021 May 29;106(6):1674–91. doi: 10.1111/tpj.15263 (PMC8362117; doi:10.1111/tpj.15263)
Supplement: Supplementary file 12 — Table S8. Segregation and haplotyping for Sr13 in historical landraces of tetraploid wheat. Table S9. Analysis of pustule size when four genotypes where inoculated with three races of Puccinia graminis f. sp. tritici at St Paul, MN, USA. Table S10. Stem rust infection types recorded on nine monogenic tetraploid wheat lines developed by N. D. Williams and tested on 21 stem rust races by Miller, Williams and Klindworth at Fargo, ND, in 1995 and on five races by Yue Jin at St Paul, MN in 2008. Table S11. Infection types (ITs) observed when CAT‐A1 and 10 other genotypes were tested with nine races of Puccinia graminis f. sp. tritici at 25°C and 20°C at Fargo, ND. Table S12. Infection types observed when CAT‐A1 and 10 other genotypes were tested with races JRCQC and TTKSK of Puccinia graminis f. sp. tritici at 25°C and 20°C at St Paul, MN. Table S13. Analysis of durum and common wheat cultivars or lines with six STARP markers linked to the Sr13 locus. Table S14. Pedigrees and Sr13 haplotypes of North Dakota durum cultivars. Table S15. Avirulence/virulence formula for Puccinia graminis f. sp. tritici races used or mentioned in this study. [file TPJ-106-1674-s011.docx]

| **Table S8.**  Segregation and haplotyping for *Sr13* in historical landraces of tetraploid wheat. | | | | | |
| --- | --- | --- | --- | --- | --- |
| Accession name |  | Year  cataloged | Total tested | No *Sr13* positive | Haplotype of *Sr13* pos. |
| Iumillo | PI 5996 | 1901 | 39 | 0 | - |
|  | PI 174662 | 1949 | 38 | 0 | - |
|  | PI 210973 | 1953 | 8 | 0 | - |
|  | PI 377886 | 1973 | 8 | 0 | - |
|  | PI 519716 | 1987 | 8 | 0 | - |
|  | CDL Bulk | unknown | 37 | 0 | - |
|  | Total |  | 138 | 0 | - |
| Im-B7 |  |  | 1 | 1 | R2 |
| Im-C2 |  |  | 1 | 1 | R2 |
|  |  |  |  |  |  |
| Khapli | CItr 4013 | 1908 | 28 | 14 | R1 |
|  | PI 101971 | 1933 | 8 | 8 | R1 |
|  | CDL Bulk | unknown | 8 | 8 | R1 |
|  |  |  |  |  |  |
| ST464 | PI 191365 | 1950 | 29 | 13 | R3 |
| CItr 8155 | CItr 8155 | 1924 | 27 | 22 | R3 |
| Camadi Abdu tipo #103 | PI 192168 | 1950 | 15 | 6 | R4 |
| Khapstein | PI 210125 | 1953 | 5 | 5 | R1 |
| Langdon (Fargo) | PI 13165 | 1954 | 1 | 1 | R3 |
| Langdon (Aberdeen) | PI 13165 | 1954 | 2 | 0 | - |
| Leeds | CItr 13768 | 1963 | 2 | 2 | R2 |
|  | | | | | |

| **Table S9**. Analysis of pustule size when four genotypes where inoculated with three races of *Puccinia graminis* f. sp. *tritici* at St. Paul, Minnesota, USA. | | | | | |
| --- | --- | --- | --- | --- | --- |
| Race | Line | Gene | Pustule number | Average pustule size (mm^2^) | p-value |
| TCMJC | Fielder | - | 15 | 5.68 |  |
|  | T_2_-Sr13 | *Sr13-R3* | 15 | 3.92 | 0.049 |
|  | Kronos | *Sr13-R1* | 54 | 3.16 |  |
|  | T_4_-3102 | - | 48 | 2.75 | 0.107 |
|  |  |  |  |  |  |
| THTSC | Fielder | - | 14 | 3.27 |  |
|  | T_2_-Sr13 | *Sr13-R3* | 18 | 2.13 | 0.015 |
|  | Kronos | *Sr13-R1* | 62 | 0.94 |  |
|  | T_4_-3102 | - | 35 | 2.36 | 1.16 × 10^-7^ |
|  |  |  |  |  |  |
| TTKSK | Fielder | - | 163 | 3.13 |  |
|  | T_2_-Sr13 | *Sr13-R3* | 203 | 1.16 | 4.95 × 10^-15^ |
|  | Kronos | *Sr13-R1* | 68 | 0.66 |  |
|  | T_4_-3102 | - | 245 | 1.47 | 6.05 × 10^-18^ |
| The T_2_-Sr13 transgenic has *Sr13* transformed into a Fielder background and thus carries the R3 haplotype. The T_4_-3102 line is an EMS mutant of Kronos that carries a premature stop codon in *CNL13* (Zhang et al, 2017) and thus loss of function of *Sr13.* | | | | | |

| **Table S10.** Stem rust infection types recorded on nine monogenic tetraploid wheat lines developed by ND Williams and tested on 21 stem rust races by Miller, Williams, and Klindworth at Fargo, North Dakota, USA in 1995 | | | | | | | | | | | | | | | | | | |
| --- | --- | --- | --- | --- | --- | --- | --- | --- | --- | --- | --- | --- | --- | --- | --- | --- | --- | --- |
| and on five races by Yue Jin at St. Paul, Minnesota, USA in 2008. | |  |  |  |  |  |  |  |  |  |  |  |  |  |  |  |  |  |
|  |  |  |  | Race and Isolate Fargo 1995 | | | | | | | | | | | | | |  |
|  |  |  |  | R111 | A-14 | R29M | A-15 | gb 121 | or 9E | A-5 | or R11c | WM-1 | 36-55A | QCC-2 | 64E(1)sp1 | A-1 | 370C |  |
| Material | Pedigree | Haplotype | Allele | LBBLB | HPHJC | HKHJC | HPLGC | JCMNC | KCCJC | MCCFC | RCMJC | RCCDC | RCCNC | QCCJC | QTHJC | QTHJC | QFCSC | haplotype |
| KL-B | Marruecos 9623//Khapli/Marruecos 9623 | R1 | *Sr13a* | 2 | 210; | 21 | 1 | 1 | 12 | 21 | 21 | 2 | 12 | 2 | 210; | 1 | 21 | R1 |
| KL-C | Marruecos 9623//Khapli/Marruecos 9623 | R1 | *Sr13a* | 2 | 210; | 21 | 10; | 1 | 12 | 2 | 21 | 120; | 21 | 23 | 21 | 12 | 10; | R1 |
| Im-B7 (digenic) | Marruecos 9623//Iumillo/Marruecos 9623 | R2 | *Sr13b+* | 0;1 | 123rt0; | 123c^-^ | 10; | 210; | 21/32 | 2 | 21 | 12 | 3 | 32 | 2 | 10;33^+^rt | 32 | R2 |
| Im-C2 | Marruecos 9623//Marruecos 9623/Iumillo | R2 | *Sr13b* | 12^-^ | 23^-^c^-^ | 23c1 | 1 | 12 | 32 | 21 | 23 | 23/32 | 3/2 | 3 | 23 | 33^+^rt2 | 32 | R2 |
| 8155-C1 | Marruecos 9623//Marruecos 9623/CI 8155 | R3 | *Sr13c* | 12 | 10; | 0;1 | 10; | 10; | 10; | 1 | 0;1 | 0;1 | 10; | 1/30; | 10; | 0;1 | 10; | R3 |
| 8155-B2 | Marruecos 9623//Marruecos 9623/CI 8155 | R3 | *Sr13c* | 21 | 10; | 10; | 10; | 10; | 120; | 12 | 0;1 | 12 | 10; | 1 | 120; | 0;1 | 12^-^0; | R3 |
| ST-464-C1 | Marruecos 9623//Marruecos 9623/ST464 | R3 | *Sr13c* | 0;1/120; | 120; | 12 | 10; | 10; | 10; | 12 | 0;1 | 120; | 0;1 | 12 | 120; | 12 | 120; | R3 |
| CAT-A1 | Marruecos 9623//Marruecos 9623/Camadi Abdu tipo #103 | R4 |  | 12/23 | 2 | 213c^-^ | 1 | 1 | 21 | 21 | 12 | 23 | 12 | 21 | 12 | 12 | 12^-^ | R4 |
| CAT-B1 | Marruecos 9623/Camadi Abdu tipo #103 | - | - | 0;1 | 0;1^-^ | 10; | 0;1 | 120; | 120; | 0;1 | 0;1 | 0;1 | 0;1 | 0;n | 0;1 | 10; | 0;1 | - |
|  |  |  |  | c - more chlorosis than normally observed, n - necrosis, rt - ring tendency | | | | | | |  |  |  |  |  |  |  |  |
|  |  |  |  |  |  |  |  |  |  |  |  |  |  |  |  |  |  |  |
|  |  |  |  | Race and Isolate Fargo 1995 | | | | | | |  | Race and Isolate St. Paul 2008 | | | | |  |  |
|  |  |  |  | 72.00 | 72-41sp1 | TNMKsp1 | A-12 | A-21 | or41-21sp-1 | 81AC-46(2) |  |  | | | | | |  |
| Material | Pedigree | Haplotype | Allele | RTQQC | TMLKC | TPMKC | THTSC | TCMJC | TCCJC | TPPKC |  | TTTTF | TRTT | TTKST | TTTSK | TTKSK | haplotype |  |
| KL-B | Marruecos 9623//Khapli/Marruecos 9623 | R1 | *Sr13a* | 21 | 2 | 21 | 3 | 34 | 2 | 21 |  | 4 | 2^+^ | 2^+^ | 2^+^ | 2^+^ | R1 |  |
| KL-C | Marruecos 9623//Khapli/Marruecos 9623 | R1 | *Sr13a* | 21 | 2 | 21 | 32 | 34 | 2 | 21 |  | 4 | 2^++^ | 2^+^ | 2^+^ | 2^+^ | R1 |  |
| Im-B7 (digenic) | Marruecos 9623//Iumillo/Marruecos 9623 | R2 | *Sr13b+* | 21 | 12 | 12 | 1 | 12 | 1 | 1 |  | 2 | 2^-^ | 2^-^ | 2^-^ | 2^-^ | R2 |  |
| Im-C2 | Marruecos 9623//Marruecos 9623/Iumillo | R2 | *Sr13b* | 21 | 12 | 12 | 1 | 13c | 12 | 1 |  | 2^+^ | 2^+^ | 2^+^ | 2^+^ | 2 | R2 |  |
| 8155-C1 | Marruecos 9623//Marruecos 9623/CI 8155 | R3 | *Sr13c* | 21 | 1 | 21 | 1 | 1 | 10; | 0;1 |  | - | - | 2^-^ | 2^-^ | 2^-^; | R3 |  |
| 8155-B2 | Marruecos 9623//Marruecos 9623/CI 8155 | R3 | *Sr13c* | 21 | 12 | 12 | 12 | 12 | 12^-^0; | 10; |  | 2 | 2^+^ | 2^-^ | 2 | 22^+^ | R3 |  |
| ST-464-C1 | Marruecos 9623//Marruecos 9623/ST464 | R3 | *Sr13c* | 21 | 12 | 12 | 1 | 12 | 12 | 0;1 |  | 2^+^ | - | - | 2 | 2 | R3 |  |
| CAT-A1 | Marruecos 9623//Marruecos 9623/Camadi Abdu tipo #103 | R4 |  | 21 | 12 | 2 | 32 | 34 | 34 | 33^+^ |  | - | - | - | - | - | R4 |  |
| CAT-B1 | Marruecos 9623/Camadi Abdu tipo #103 | - | - | 21 | 33^+^2 | 34 | 3 | 310; | 10;3cn | 0;13cn^-^ |  | - | - | - | - | - | - |  |
|  |  |  |  | c - more chlorosis than normally observed, n – necrosis | | | | |  |  |  |  |  |  |  |  |  |  |

| **Table S11**. Infection types (ITs) observed when CAT-A1 and ten other genotypes were tested with nine races of *Puccinia graminis* f. sp. *tritici* at 25℃ and 20℃ at Fargo, North Dakota, USA. | | | | | | | | | | | | |
| --- | --- | --- | --- | --- | --- | --- | --- | --- | --- | --- | --- | --- |
|  |  |  |  | Race | | | | | | | | |
| Genotype | Haplotype | Trial | Temp. | QFCSC | QCCJC | QTHJC | RTQQC | TCCJC | TCMJC | TMLKC | TPMKC | TPPKC |
| Rusty | - | 1 | 25℃ | 34 | 34 | 34 | 34 | 34 | 34/23 | 34 | 34 | 34 |
| KL-B | R1 | 1 | 25℃ | 2^+^ | 23 | 23 | 1 | 23 | 34 | 2^-^c^+^ | 1c^+^ | 12 |
| Rusty-14803 | R2 | 1 | 25℃ | 34 | 34 | 23/23^+^ | 1 | 12 | 21 | 2^-^c | 1c | 21 |
| Im-C2 | R2 | 1 | 25℃ | 34 | 34 | 23 | 1 | 12c | 21 | 2^-^ | 1 | 12 |
| Im-B7 | R2 | 1 | 25℃ | 34 | 34 | 23 | 1 | 12 | 1^+^c | 2^-^ | 1 | 12 |
| 8155-B2 | R3 | 1 | 25℃ | 2c | 1^+^c | 2 | 1^-^ | 2^-^ | 21 | 2^-^c^+^ | 1c^+^ | 1^-^ |
| 8155-C1 | R3 | 1 | 25℃ | 2c | 2^-^/0;1^-^ | 2^-^ | 1^-^ | 2^-^ | 21 | 1^+^c | 1^-^c/0; | 11^-^ |
| ST464-C1 | R3 | 1 | 25℃ | 22^+^c | 1^+^c | 23^-^/23 | 1 | 2/23 | 21 | 2^-^ | 2^-^c | 1^-^ |
| CAT-A1 | R4 | 1 | 25℃ | 23/34 | 2 | 23 | 2 | 34 | 32 | 23^-^ | 23 | 23 |
| CAT-B1 | non-*Sr13* | 1 | 25℃ | 2^-^c | 1cn | 2/23 | 2^-^ | 32 | 23/34 | 32 | 34/23 | 32 |
| Camadi-1 † | R4 | 1 | 25℃ | 1^+^c^+^ | 2 | 1^+^/2 | 1 | 34/23 | 1/34 | 23^+^ | 34/23 | 34 |
|  |  |  |  |  |  |  |  |  |  |  |  |  |
| Rusty | - | 2 | 25℃ | 4 | 4 | 4 | 4 | 4 | 34/4 | 4 | 4 | 4 |
| KL-B | R1 | 2 | 25℃ | 2^+^ | 34 | 2^+^ | 2 | 34 | 34 | 1^+^ | 2^-^ | 2^+^3^-^c |
| Rusty-14803 | R2 | 2 | 25℃ | 34 | 34 | 34 | 2 | 2^-^ | 2/22^+^ | 2^-^ | 2^-^c | 1^+^c |
| Im-C2 | R2 | 2 | 25℃ | 34 | 34 | 34/23 | 2^-^ | 2^-^ | 2 | 2^-^ | 0; | 1^+^/1^+^c |
| Im-B7 | R2 | 2 | 25℃ | 34 | 34 | 34/23 | 2 | 1^+^ | 12/2 | 2^-^ | 1^+^ | 1^+^c |
| 8155-B2 | R3 | 2 | 25℃ | 2 | 2^-^ | 2 | 2/1^-^ | 1^+^ | 2 | 2^-^ | 1 | 1 |
| 8155-C1 | R3 | 2 | 25℃ | 2 | 2^-^ | 2^-^ | 2 | 1^+^ | 2^+^ | 2^-^ | 1 | 1^-^ |
| ST464-C1 | R3 | 2 | 25℃ | 2 | 2^-^ | 2^+^ | 2 | 1^+^c | 2^+^ | 2^-^ | 1^+^c | 1^-^c |
| CAT-A1 | R4 | 2 | 25℃ | 1,3 | 4/2^+^ | 2^+^ | 2 | 34 | 34 | 2^+^ | 2/2^+^ | 2^+^ |
| CAT-B1 | non-*Sr13* | 2 | 25℃ | 1 | 1 | 2^+^ | 2 | 34 | 34 | 4 | 4 | 2^+^3^-^c |
| Camadi-1 † | R4 | 2 | 25℃ | 1 | 0; | 2^+^ | 1 | 22^+^ | 34/2^+^ | 4 | 4 | 2^-^ |
|  |  |  |  |  |  |  |  |  |  |  |  |  |
| Rusty | - | 3 | 20℃ | 4 | 4 | 4 | 4 | 4 | 34 | 4 | 4 | 34 |
| KL-B | R1 | 3 | 20℃ | 32 | 2^+^ | 2^+^3^-^ | 2 | 32 | 34 | 2c | 2c | 2^+^/34 |
| Rusty-14803 | R2 | 3 | 20℃ | 34 | 34 | 3 | 2 | 22^+^ | 2^+^ | 2^-^c^+^ | 2 | 2c |
| Im-C2 | R2 | 3 | 20℃ | 4 | 34 | 2^+^/3 | 2 | 2^-^/2^+^ | 2^+^ | 2^-^ | 2^-^ | 2 |
| Im-B7 | R2 | 3 | 20℃ | 4 | 34 | 3 | 2 | 2^-^ | 2 | 2^-^ | 2^-^ | 2 |
| 8155-B2 | R3 | 3 | 20℃ | 2^-^ | 2^-^ | 2 | 0;1^-^ | 2^-^ | 2^+^ | 2^-^ | 2^-^c | 1 |
| 8155-C1 | R3 | 3 | 20℃ | 2 | 2^-^c | 1^+^ | 2 | 2^-^/2 | 2/2^+^ | 2 | 2^-^ | 2^-^ |
| ST464-C1 | R3 | 3 | 20℃ | 2^+^ | 2-c | 2^+^ | 2 | 22^+^/2 | 2^+^ | 2^-^c^+^ | 2^-^c | 2^-^c/2 |
| CAT-A1 | R4 | 3 | 20℃ | 2^+^ | 2^-^/2^+^ | 2^+^ | 2/2^+^ | 34 | 34/2^+^3 | 2^+^ | 2 | 2^+^ |
| CAT-B1 | non-*Sr13* | 3 | 20℃ | 1^-^ | 0;1/1 | 1,3 | 2 | 34 | 34 | 4 | 4 | 34 |
| Camadi-1 † | R4 | 3 | 20℃ | 0;1^-^ | 0; | 0;1^-^ | 0;1^-^ | 2^-^ | 34/2^+^3 | 2^+^ | 2/2^+^ | 2^-^c |
| † Camadi Abdu tipo #103-1 | | | | | | | | | | | | |

| **Table S12**. Infection types (ITs) observed when CAT-A1 and ten other genotypes were tested with races JRCQC and TTKSK of *Puccinia graminis* f. sp. *tritici* at 25℃ and 20℃ at St Paul, Minnesota, USA. | | | | | | | | |
| --- | --- | --- | --- | --- | --- | --- | --- | --- |
| Genotype | *Sr13* haplotype | Marker *rwgsnp37.2* |  | JRCQC | |  | TTKSK | |
|  |  |  |  | 22℃ | 25℃ |  | 22℃ | 25℃ |
| Rusty | - | - |  | 4 | 4 |  | 4 | 3^+^ |
| KL-B | R1 | + |  | 2^+^ | 2^+^ |  | 22^+^ | 2 |
| Rusty-14803 | R2 | + |  | 3^+^ | 33^+^ |  | 2 | 2 |
| Im-C2 | R2 | + |  | 4 | 4 |  | 2 | 2 |
| Im-B7 | R2 | + |  | 3^+^ | 33^+^ |  | 2 | 2^-^ |
| 8155-B2 | R3 | + |  | 2 | 2 |  | 2 | 22^-^ |
| 8155-C1 | R3 | + |  | 2^-^ | 22^-^ |  | 2^-^ | 2^-^ |
| ST464-C1 | R3 | + |  | 2^+^ | 2^+^ |  | 2^+^ | 2^+^ |
| CAT-A1, plant #1 | R4 | + |  | 2^+^ | 2^+^ |  | 3 | 3 |
| CAT-A1, plant #2 | R4 | + |  | 2^+^3 | 2^+^ |  | 2^+^3 | 2^+^3 |
| CAT-A1, plant #3 | R4 | + |  | 2^+^3 | 3 |  | 3 | 3 |
| CAT-B1 | non-*Sr13* | - |  | 3 | 3^+^ |  | 3^+^ | 3^+^ |
| Camadi-1 † | R4+ | + |  | 33^+^ | 3^+^ |  | 33^+^c | 3 |
| Camadi-2 † | - | - |  | ;11^+^ | 1^+^1;3^-^ |  | 4 | 3^+^ |
| Camadi-3 † | R4+ | + |  | 22^+^ | 2^+^ |  | 22^+^ | 2 |
| Camadi-4 † | - | - |  | 32+ | 32+ |  | 33+ | 3+ |
| † Camadi Abdu tipo #103 | | | | | | | | |

| **Table S13.**  Analysis of durum and common wheat cultivars or lines with six STARP markers linked to the *Sr13* locus. | | | | | | | | | | |
| --- | --- | --- | --- | --- | --- | --- | --- | --- | --- | --- |
| Cultivar/line | Type | Habit | Origin † | *rwgsnp6* | *rwgsnp7*‡ | *rwgsnp37.2* | *rwgsnp38* | *rwgsnp39* | *rwgsnp40* | Allele |
| Rusty | Durum | Spring | ND, USA | A1 | A1 | *Null* | *Null* | *Null* | *Null* |  |
| PI 387696 | *T. carthlicum* | Spring | ID, USA | A2 | A2 | *Sr13* | R2 | R1R2R4 | R2R3R4 | *Sr13b* |
| Khapli, CItr 4013 | Emmer | Spring | India | - | - | *Sr13* | R1R3R4 | R1R2R4 | R1 | *Sr13a* |
| Rusty-KL-B | Durum | Spring | ND, USA | - | - | *Sr13* | R1R3R4 | R1R2R4 | R1 | *Sr13a* |
| Rusty-KL-C | Durum | Spring | ND, USA | - | - | *Sr13* | R1R3R4 | R1R2R4 | R1 | *Sr13a* |
| Strongfield | Durum | Spring | Canada | A1 | A2 | *Sr13* | R1R3R4 | R1R2R4 | R1 | *Sr13a* |
| Transcend | Durum | Spring | Canada | A1 | A2 | *Sr13* | R1R3R4 | R1R2R4 | R1 | *Sr13a* |
| Grenora | Durum | Spring | ND, USA | A2 | A2 | *Sr13* | R1R3R4 | R1R2R4 | R1 | *Sr13a* |
| Wells | Durum | Spring | ND, USA | - | - | *Sr13* | R1R3R4 | R1R2R4 | R1 | *Sr13a* |
| Lakota | Durum | Spring | ND, USA | - | - | *Sr13* | R1R3R4 | R1R2R4 | R1 | *Sr13a* |
| Cando | Durum | Spring | ND, USA | - | - | *Sr13* | R1R3R4 | R1R2R4 | R1 | *Sr13a* |
| Mountrail | Durum | Spring | ND, USA | - | - | *Sr13* | R1R3R4 | R1R2R4 | R1 | *Sr13a* |
| Durox | Durum | Spring | WA, USA | - | - | *Sr13* | R1R3R4 | R1R2R4 | R1 | *Sr13a* |
| Kronos | Durum | Spring | AZ, USA | - | - | *Sr13* | R1R3R4 | R1R2R4 | R1 | *Sr13a* |
| Leeds | Durum | Spring | ND, USA | - | - | *Sr13* | R2 | R1R2R4 | R2R3R4 | *Sr13b* |
| Svevo | Durum | Spring | Italy | A2 | A2 | *Sr13* | R2 | R1R2R4 | R2R3R4 | *Sr13b* |
| D151343 | Durum | Spring | ND, USA | A2 | A2 | *Sr13* | R2 | R1R2R4 | R2R3R4 | *Sr13b* |
| ND Grano | Durum | Spring | ND, USA | A2 | A2 | *Sr13* | R2 | R1R2R4 | R2R3R4 | *Sr13b* |
| ND Riveland | Durum | Spring | ND, USA | A2 | A2 | *Sr13* | R2 | R1R2R4 | R2R3R4 | *Sr13b* |
| Joppa | Durum | Spring | ND, USA | A2 | A2 | *Sr13* | R2 | R1R2R4 | R2R3R4 | *Sr13b* |
| Lebsock | Durum | Spring | ND, USA | A2 | A2 | *Sr13* | R2 | R1R2R4 | R2R3R4 | *Sr13b* |
| Carpio | Durum | Spring | ND, USA | A2 | A2 | *Sr13* | R2 | R1R2R4 | R2R3R4 | *Sr13b* |
| Ben | Durum | Spring | ND, USA | - | - | *Sr13* | R2 | R1R2R4 | R2R3R4 | *Sr13b* |
| Pierce | Durum | Spring | ND, USA | - | - | *Sr13* | R2 | R1R2R4 | R2R3R4 | *Sr13b* |
| Ward | Durum | Spring | ND, USA | - | - | *Sr13* | R2 | R1R2R4 | R2R3R4 | *Sr13b* |
| Rugby | Durum | Spring | ND, USA | - | - | *Sr13* | R2 | R1R2R4 | R2R3R4 | *Sr13b* |
| Botno | Durum | Spring | ND, USA | - | - | *Sr13* | R2 | R1R2R4 | R2R3R4 | *Sr13b* |
| Munich | Durum | Spring | ND, USA | - | - | *Sr13* | R2 | R1R2R4 | R2R3R4 | *Sr13b* |
| Calvin | Durum | Spring | ND, USA | - | - | *Sr13* | R2 | R1R2R4 | R2R3R4 | *Sr13b* |
| Vic | Durum | Spring | ND, USA | - | - | *Sr13* | R2 | R1R2R4 | R2R3R4 | *Sr13b* |
| Plaza | Durum | Spring | ND, USA | - | - | *Sr13* | R2 | R1R2R4 | R2R3R4 | *Sr13b* |
| Lloyd | Durum | Spring | ND, USA | - | - | *Sr13* | R2 | R1R2R4 | R2R3R4 | *Sr13b* |
| Tioga | Durum | Spring | ND, USA | - | - | *Sr13* | R2 | R1R2R4 | R2R3R4 | *Sr13b* |
| Sceptre | Durum | Spring | SK, Canada | - | - | *Sr13* | R2 | R1R2R4 | R2R3R4 | *Sr13b* |
| Medora | Durum | Spring | MB, Canada | - | - | *Sr13* | R2 | R1R2R4 | R2R3R4 | *Sr13b* |
| 340, CItr 7777 | Durum | Spring | Ethiopia | - | - | *Sr13* | R2 | R1R2R4 | R2R3R4 | *Sr13b* |
| Im-B7 | Durum | Spring | ND, USA | - | - | *Sr13* | R2 | R1R2R4 | R2R3R4 | *Sr13b* |
| Im-C2 | Durum | Spring | ND, USA | - | - | *Sr13* | R2 | R1R2R4 | R2R3R4 | *Sr13b* |
| CItr8155 | Poulard | Spring | Ethiopia | - | - | *Sr13* | R1R3R4 | R3 | R2R3R4 | *Sr13c* |
| 8155-B2 | Durum | Spring | ND, USA | - | - | *Sr13* | R1R3R4 | R3 | R2R3R4 | *Sr13c* |
| 8155-C1 | Durum | Spring | ND, USA | - | - | *Sr13* | R1R3R4 | R3 | R2R3R4 | *Sr13c* |
| ST464 | Durum | Spring | Ethiopia | - | - | *Sr13* | R1R3R4 | R3 | R2R3R4 | *Sr13c* |
| Rusty-ST464-C1 | Durum | Spring | ND, USA | - | - | *Sr13* | R1R3R4 | R3 | R2R3R4 | *Sr13c* |
| Altar 84 | Durum | Spring | CIMMYT | - | - | *Sr13* | R1R3R4 | R3 | R2R3R4 | *Sr13c* |
| PI 352548 | Durum |  |  | - | - | *Sr13* | R1R3R4 | R3 | R2R3R4 | *Sr13c* |
| Langdon | Durum | Spring | ND, USA | A2 | A2 | *Sr13* | R1R3R4 | R3 | R2R3R4 | *Sr13c* |
| D101073 | Durum | Spring | ND, USA | A2 | A2 | *Sr13* | R1R3R4 | R3 | R2R3R4 | *Sr13c* |
| Alkabo | Durum | Spring | ND, USA | A2 | A2 | *Sr13* | R1R3R4 | R3 | R2R3R4 | *Sr13c* |
| 340, CItr 7771 | Durum | Spring | Ethiopia | - | - | *Sr13* | R1R3R4 | R3 | R2R3R4 | *Sr13c* |
| Camadi Abdu tipo | Durum | Spring | Ethiopia | - | - | *Sr13* | R1R3R4 | R1R2R4 | R2R3R4 |  |
| CAT-A1 | Durum | Spring | ND, USA | - | - | *Sr13* | R1R3R4 | R1R2R4 | R2R3R4 |  |
| Divide | Durum | Spring | ND, USA | A2 | A2 | *Null* | False pos | False pos | False pos |  |
| Monroe | Durum | Spring | ND, USA | - | - | *Null* | False pos | *Null* | False pos |  |
| Dilse | Durum | Spring | ND, USA | - | - | *Null* | False pos | *Null* | False pos |  |
| Edmore | Durum | Spring | ND, USA | - | - | *Null* | False pos | *Null* | False pos |  |
| Belzer | Durum | Spring | ND, USA | - | - | *Null* | False pos | *Null* | False pos |  |
| Crosby | Durum | Spring | ND, USA | - | - | *Null* | - | - | - |  |
| Carleton | Durum | Spring | ND, USA | - | - | *Null* | *Null* | *Null* | *Null* |  |
| Stewart | Durum | Spring | ND, USA | - | - | *Null* | *Null* | *Null* | *Null* |  |
| Ramsey | Durum | Spring | ND, USA | - | - | *Null* | *Null* | *Null* | *Null* |  |
| Cappelli | Durum | Spring | Italy | A1 | A1 | *Null* | *Null* | *Null* | - |  |
| 340, CItr 7780 | Durum | Spring | Ethiopia | - | - | *Null* | - | - | - |  |
| Iumillo, PI 5996 | Durum | Spring | Italy | - | - | *Null* | *Null* | *Null* | *Null* |  |
|  |  |  |  |  |  |  |  |  |  |  |
| Line E | Common | Spring | Australia | A1 | A2 |  |  |  |  |  |
| BR34 | Common | Spring | Brazil | A2 | A1A2 |  | False pos | False pos |  |  |
| LMPG-6 | Common | Spring | SK, Canada | A2 | A2 |  | False pos | False pos |  |  |
| Chinese Spring | Common | Spring | China | A2 | A1A2 |  |  |  |  |  |
| Sumai 3 | Common | Spring | China | A2 | A1A2 |  |  |  |  |  |
| Zhoumai 27 | Common | Winter | China | A2 | A1A2 |  |  |  |  |  |
| Jimai 22 | Common | Winter | China | A2 | A1A2 |  | False pos | False pos |  |  |
| Jinqiang 5 | Common | Spring | China | A2 | A1A2 |  | False pos | False pos |  |  |
| Yangmai 16 | Common | Spring | China | A2 | A1A2 |  | False pos | False pos |  |  |
| Zhengmai 9023 | Common | Facultative | China | A2 | A1A2 |  | False pos | False pos |  |  |
| Alsen | Common | Spring | ND, USA | A2 | A1A2 |  | False pos | False pos |  |  |
| Barlow | Common | Spring | ND, USA | A2 | A1A2 |  | False pos | False pos |  |  |
| Faller | Common | Spring | ND, USA | A2 | A2 |  |  |  |  |  |
| Grandin | Common | Spring | ND, USA | A2 | A2 |  |  |  |  |  |
| Elgin-ND | Common | Spring | ND, USA | A2 | A1A2 |  |  |  |  |  |
| Glenn | Common | Spring | ND, USA | A2 | A1A2 |  |  |  |  |  |
| ND830 | Common | Spring | ND, USA | A2 | A1A2 |  |  |  |  |  |
| ND833 | Common | Spring | ND, USA | A2 | A1A2 |  |  |  |  |  |
| NDHRS16-12-19 | Common | Spring | ND, USA | A2 | A1A2 |  |  |  |  |  |
| Reeder | Common | Spring | ND, USA | A2 | A1A2 |  |  |  |  |  |
| Steele-ND | Common | Spring | ND, USA | A2 | A1A2 |  |  |  |  |  |
| VitPro-ND | Common | Spring | ND, USA | A2 | A1A2 |  |  |  |  |  |
| IL06-14262 | Common | Winter | IL, USA | A2 | A1A2 |  |  |  |  |  |
| Newton | Common | Winter | KS, USA | A2 | A1A2 |  |  |  |  |  |
| Tom | Common | Spring | MN, USA | A2 | A2 |  |  |  |  |  |
| Linkert | Common | Spring | MN, USA | A2 | A1A2 |  | False Pos | False pos |  |  |
| Ada | Common | Spring | MN, USA | A2 | A1A2 |  |  |  |  |  |
| Bolles | Common | Spring | MN, USA | A2 | A1A2 |  |  |  |  |  |
| Brick | Common | Spring | SD, USA | A2 | A1A2 |  |  |  |  |  |
| Granger | Common | Spring | SD, USA | A2 | A1A2 |  |  |  |  |  |
| † Origin: ND, North Dakota; SD, South Dakota; MN, Minnesota; IL, Illinois; ID, Idaho; KS, Kansas; MB, Manitoba; SK, Saskatchewan.  ‡ Presence of both Rusty and PI 387696 alleles (A1A2) in the 25 common wheat cultivars/lines might be caused by the homoealleles from different chromosomes detected by marker *rwgsnp7.* | | | | | | | | | | |

| **Table S14**. Pedigrees and *Sr13* haplotypes of North Dakota durum cultivars. | | | | | | | | | | | | | | | | |
| --- | --- | --- | --- | --- | --- | --- | --- | --- | --- | --- | --- | --- | --- | --- | --- | --- |
| Cultivar | | Year  Released | | | Cultivar  haplotype | | | Pedigree | Notes | | | | | Source  of *Sr13* | | |
| Carleton | | 1943 | | | S | | | Mindum*^4^/Vernal |  | | | | | none | | |
| Stewart | | 1943 | | | S | | | Vernal/*^3^Mindum |  | | | | | none | | |
| Sentry | | 1954 | | | S | | | Ld308/Nugget |  | | | | | none | | |
| Langdon | | 1956 | | | **R3** | | | Ld194/Khapli//Ld308/3/Stewart/4/Carleton |  | | | | | unknown | | |
| Yuma | | 1956 | | | ? | | | Ld194/Khapli//Ld308 |  | | | | | **?** | | |
| Ramsey | | 1956 | | | S | | | Carleton/PI94701 |  | | | | | none | | |
| Towne | | 1956 | | | S | | | Carleton/PI94701 |  | | | | | none | | |
| Lakota | | 1960 | | | R1 | | | Sentry//Ld379/Ld357 | Wells sib | | | | | LD379 | | |
| Wells | | 1962 | | | R1 | | | Sentry//Ld379/Ld357 | Lakota sib | | | | | LD379 | | |
| Leeds | | 1967 | | | R2 | | | Ld357/ST464//Ld357/3/Wells |  | | | | | unknown | | |
| Rolette | | 1972 | | | R1 | | | Ld393/^2^*Yuma/3/Ld398//Ld357*^2^/ST464 |  | | | | | Ld393 or Ld398 | | |
| Ward | | 1972 | | | R2 | | | Langdon/3/Ld357//CItr7780/Ld362/4/Br180/Wells |  | | | | | unknown | | |
| Rugby | | 1973 | | | R2 | | | Langdon/3/Ld357//CItr7780/Ld362/4/Br180/Wells |  | | | | | unknown | | |
| Botno | | 1973 | | | R2 | | | Langdon/3/Ld357//CItr7780/Ld362/4/Br180/Wells |  | | | | | unknown | | |
| Crosby | | 1973 | | | **S** | | | Langdon*^2^/ST464//Leeds |  | | | | | ST464 † | | |
| Cando | | 1975 | | | R1 | | | Lakota/5/Willet sib//Norin 10/Brevor/3/Langdon/4/Langdon/6/Leeds | Calvin sib | | | | | Lakota | | |
| Edmore | | 1978 | | | **S** | | | D6530/D65114 |  | | | | | Capelli (S4) | | |
| Vic | | 1979 | | | R2 | | | Edmore/Ward |  | | | | | Ward | | |
| Calvin | | 1980 | | | R2 | | | Lakota/5/Willet sib//Norin 10/Brevor/3/Langdon/4/Langdon/6/Leeds | Cando sib | | | | | Leeds | | |
| Lloyd | | 1983 | | | R2 | | | Cando/Edmore |  | | | | | unknown | | |
| Monroe | | 1985 | | | **S** | | | D6771/Rugby//Vic |  | | | | | Stewart | | |
| Renville | | 1988 | | | R1 | | | Rolette/Vic |  | | | | | Rolette | | |
| Munich | | 1995 | | | R2 | | | D65150/Leeds//Ward/3/D62220/D57114//Leeds/4/Ward/Macoun//Ward/3/Vic | |  | | | | multiple R2 | | |
| Ben | | 1996 | | | R2 | | | D68111/Rugby//Ward/3/Vic/4/Monroe |  | | | | | multiple R2 | | |
| Belzer | | 1997 | | | **S** | | | Lakota/DwF4-Ldn//Leeds/3/Ward/4/Edmore/5/DT367 |  | | | | | Edmore | | |
| Mountrail | | 1998 | | | R1 | | | D8479/Renville |  | | | | | Renville | | |
| Maier | | 1998 | | | R1 | | | D68111/Rugby//Crosby/3/Vic/4/Wascana/Rolette//Vic |  | | | | | Rolette | | |
| Plaza | | 1999 | | | R2 | | | Plenty/3/Cando//Edmore/Coulter |  | | | | | Plenty or Coulter | | |
| Lebsock | | 1999 | | | R2 | | | Munich/3/Vic/D7025//Edmore/Wakooma |  | | | | | multiple R2 | | |
| Pierce | | 2001 | | | R2 | | | Monroe/D87209//D88289 | pedigree includes Rugby, Ward, Vic, etc | | | | | multiple R2 | |  |
| Dilse | | 2002 | | | **S2** | | | Maier/D88273 | pedigree includes Edmore etc | | | | | Edmore? | |  |
| Grenora | | 2005 | | | R1 | | | D901260/D901419 | pedigree includes Yuma etc | | | | |  | | |
| Divide | | 2005 | | | **S2** | | | Ben/D901292//Belzer |  | | | | | Belzer | | |
| Alkabo | | 2005 | | | **R3** | | | D901247/D89263 |  | | | | | LDN(Dic-5B) | | |
| Tioga | | 2010 | | | R2 | | | Plaza/Maier |  | | | | | Plaza or Maier | | |
| Carpio | | 2012 | | | R2 | | | D95580/D95595 |  | | | |  | | | |
| Joppa | | 2013 | | | R2 | | | Maier/D97643 |  | | | |  | | | |
| ND Riveland | | 2017 | | | R2 | | | DT764/DH01146 | pedigree includes D95580 | | | |  | | | |
| ND Grano | | 2017 | | | R2 | | | D00752/DH01146 | pedigree includes D95580 | | | |  | | | |
|  |  | |  | | | |  | |  | | |  | | | | |
| Zhang et al (2017) reported the following haplotypes in the following lines | | | | | | | | | | | | | | | | |
| Mindum |  | | | | S4 | |  | |  | | |  | | | | |
| Capelli |  | | | | S4 | |  | |  | | |  | | | | |
| PI94701 |  | | | | S4 | |  | |  | | |  | | | | |
| Kronos |  | | | | R1 | |  | |  | | |  | | | | |
| Sceptre |  | | | | R2 | |  | |  | | |  | | | | |
| Medora |  | | | | R2 | |  | |  | | |  | | | | |
|  |  | |  | | |  | | |  | | |  | | | | |
| Some important parental lines, most of these pedigrees reported by Gough, Lebsock, and Williams ‡. | | | | | | | | | | |  | | | | | |
| Ld308 |  | | | Null (S) | | | | Heiti/Stewart//Mindum/Carleton | Possible Nugget sib | | | | | | | |
| Nugget |  | | | Null (S) | | | | Heiti/Stewart//Mindum/Carleton | Possible Ld308 sib | | | | | | | |
| Ld357 |  | | | Null (S) | | | | Ld308/Nugget | Possible Sentry sib | | | | | | | |
| Ld362 |  | | | Null (S?) | | | | Pedigree not reported, but it is reported in the Registrations of Ward, Rugby and Botno durum wheat that the parentage is similar to Ld357 |  | |  | | | | | |
| Ld379 |  | | | postulate  R1 | | | | Not available for testing, but Khapli is a parent via Ld377, so it probably  carried the *Sr13*-R1 haplotype; plus, Wells and Lakota are both R1 which they could only have gotten from Ld379. |  | | Khapli | | | | | |
| LD393 |  | | | postulate R1 | | | | Sentry/Ld 379//Ld 357 | possible Wells/Lakota sib | | | | | | LD379 | |
| LD398 |  | | | postulate R1 | | | | Sentry/Ld 379//Ld 357 | possible Wells/Lakota sib | | | | | | LD379 | |
| CItr7771 |  | | | R3 | | | | Ethiopian origin, selection from PI 58785, CItr 7780 sib |  | |  | | | | | |
| CItr7777 |  | | | R2 | | | | Ethiopian origin, selection from PI 58785, CItr 7780 sib |  | |  | | | | | |
| CItr7780 |  | | | Null (S) | | | | Ethiopian origin, selection from PI 58785, sib of CItr 7771 and CItr 7777, found in the pedigree of Ward, Rugby, and Botno |  | |  | | | | | |

† Some ST464 plants have been shown to lack *Sr13.*

‡ **Gough, F. J., Lebsock, K. L., and Williams, N. D.** (1964). Culture 15-WL of *Puccinia graminis* f. sp. *tritici* virulent on *Triticum* durum ‘Lakota’ and ‘Wells’.

*Plant Dis. Rep*. **48**, 971-973.

| Table S15. Avirulence/virulence formula for *P. graminis* f. sp. *tritici* races used or mentioned in this study. | | | | |
| --- | --- | --- | --- | --- |
| *Pgt*-code | Isolate | Origin | Avirulent | Virulent |
| LBBLB | 111SS-2 | USA-ND | *Sr6, 7b, 8a, 9b, 9d, 9e, 9g, 10, 11, 17, 21, 24, 30, 31, 36, 38, McN, Tmp* | *Sr5, 9a* |
| HKHJC | R29M | USA-ND | *Sr5, 9a, 9e, 11, 24, 30, 31, 36, 38, Tmp* | *Sr6, 7b, 8a, 9b, 9d, 9g, 10, 17, 21, McN* |
| HPHJC | A-14NW | USA-ND | *Sr5, 6, 9a, 9e, 10, 24, 30, 31, 36, 38, Tmp* | *Sr7b, 8a, 9b, 9d, 9g, 11, 17, 21, McN* |
| HPLGC | A15 | USA-ND | *Sr5, 6, 9a, 9b, 9e, 10, 17, 24, 30, 31, 38, Tmp* | *Sr7b, 8a, 9d, 9g, 11, 21, 36, McN* |
| JCMNC | gb121 | USA-ND | *Sr5, 6, 7b, 8a, 9b, 9d, 11, 24, 30, 31, 38, Tmp* | *Sr9a, 9e, 9g, 10, 17, 21, 36, McN* |
| JRCQC | 08ETH03-1 | Ethiopia | *Sr5, 7b, 8a, 9b, 10, 24, 30, 31, 36, 38, Tmp* | *Sr6, 9a, 9d, 9e, 9g, 11, 17 21, McN* |
| KCCJC | or9e | USA-ND | *Sr5, 6, 8a, 9a, 9b, 11, 24, 30, 31, 36, 38, Tmp* | *Sr7b, 9d, 9e, 9g, 10, 17, 21, McN* |
| MCCFC | A-5 | USA-ND | *Sr6, 8a, 9a, 9b, 9d, 9e, 11, 21,24, 30, 31, 36, 38* | *Sr5, 7b, 9g, 10, 17, McN, Tmp* |
| QFCSC | 370C | USA-ND | *Sr6, 7b, 9b, 9e, 11, 24, 30, 31, 36, 38, Tmp* | *Sr5, 8a, 9a, 9d, 9g, 10, 17, 21, McN* |
| QTHJC | A-1 (151QSH) | USA-ND | *Sr7b, 9a, 9e,24, 30, 31, 36, 38, Tmp* | *Sr5, 6, 8a, 9b, 9d, 9g, 10, 11, 17, 21, McN* |
| QTHJC | 64E(1) | USA-ND | *Sr7b, 9a, 9e,24, 30, 31, 36, 38, Tmp* | *Sr5, 6, 8a, 9b, 9d, 9g, 10, 11, 17, 21, McN* |
| QCCJC | QCC-2 | USA-ND | *Sr6, 7b, 8a, 9a, 9b, 9e, 11, 24, 30, 31, 36, 38, Tmp* | *Sr5, 9d, 9g, 10, 17, 21, McN* |
| RCCDC | WM-1 | USA-ND | *Sr6, 8a, 9a, 9b, 9d, 9e, 11, 24, 30, 31, 36, 38, Tmp* | *Sr5, 7b, 9g, 10, 17, 21, McN* |
| RCCNC | 36-55A | USA-ND | *Sr6, 8a, 9b, 9d, 9e, 11, 24, 30, 31, 36, 38, Tmp* | *Sr5, 7b, 9a, 9g, 10, 17, 21, McN* |
| RCMJC | or11c | USA-ND | *Sr6, 8a, 9a, 9b, 9e, 11, 24, 30, 31, 38, Tmp* | *Sr5, 7b, 9d, 9g, 10, 17, 21, 36, McN* |
| RHFSC | 72.22 | USA-ND | *Sr8a, 9b, 9e, 11, 24, 31, 36, 38, Tmp* | *Sr5, 6, 7b, 9a, 9d, 9g, 10, 17, 21, 30, McN* |
| RKQQC | RKQQ | USA-MN | *Sr9e, 10, 11, 17, 24, 30, 31, 38, Tmp* | *Sr5, 6, 7b, 8a, 9a, 9b, 9d, 9g, 21, 36, McN* |
| RTQQC | 72.00 | USA-ND | *Sr9e, 10, 17, 24, 30, 31, 38, Tmp* | *Sr5, 6, 7b, 8a, 9a, 9b, 9d, 9g, 11, 21, 36, McN* |
| TCCJC | or41-21 | USA-ND | *Sr6, 8a, 9a, 9b, 11, 24, 30, 31, 36, 38, Tmp* | *Sr5, 7b, 9d, 9e, 9g, 10, 17, 21, McN* |
| TCMJC | A-21 | USA-ND | *Sr6, 8a, 9a, 9b, 11, 24, 30, 31, 38, Tmp* | *Sr5, 7b, 9d, 9e, 9g, 10, 17, 21, 36, McN* |
| THTSC | A-12 | USA-ND | *Sr8a, 11, 24, 31, 38, Tmp* | *Sr5, 6, 7b, 9a, 9b, 9a, 9e, 9g, 10, 17, 21, 30, 36, McN* |
| TMLKC | 72-41sp2 (15TLM) | USA-ND | *Sr6, 8a, 9a, 9b, 17, 24, 30, 31, 38* | *Sr5, 7b, 9d, 9e, 9g, 10, 11, 21, 36, McN, Tmp* |
| TPMKC | TNMK (15TNM) | USA-ND | *Sr6, 9a, 9b, 24, 30, 31, 38* | *Sr5, 7b, 8a, 9d, 9e, 9g, 10, 11, 17, 21, 36, McN, Tmp* |
| TPPKC | 81AC 46(2) | USA-ND | *Sr6, 9a, 9b, 24, 31, 38* | *Sr5, 7b, 8a, 9d, 9e, 9g, 10, 11, 17, 21, 30, 36, McN, Tmp* |
| TTTTF | 01MN84A-1-2 | USA-MN | *Sr24, 31* | *Sr5, 6, 7b, 8a, 9a, 9b, 9d, 9e, 9g, 10, 11, 17, 21, 30, 36, 38, McN, Tmp* |
| TRTTF | 06YEM34-1 | Yemen | *Sr8a, 24, 31* | *Sr5, 6, 7b, 9a, 9b, 9d, 9e, 9g, 10, 11, 17, 21, 30, 36, 38, McN, Tmp* |
| TTKST | 06KEN19-V-3 | Kenya | *Sr36, Tmp* | *Sr5, 6, 7b, 8a, 9a, 9b, 9d, 9e, 9g, 10, 11, 17, 21, 24, 30, 31, 38, McN* |
| TTTSK | 07KEN24-4 | Kenya | *Sr24, Tmp* | *Sr5, 6, 7b, 8a, 9a, 9b, 9d, 9e, 9g, 10, 11, 17, 21, 30, 31, 36, 38, McN* |
| TTKSK | 04KEN156/04 | Kenya | *Sr24, 36, Tmp* | *Sr5, 6, 7b, 8a, 9a, 9b, 9d, 9e, 9g, 10, 11, 17, 21, 30, 31, 38, McN* |
| TKTTF(Eth) | † | Ethiopia | *Sr11, 24, 31* | *Sr5, 6, 7b, 8a, 9a, 9b, 9d, 9e, 9g, 10, 17, 21, 30, 36, 38, McN, Tmp* |
| TKTTF(Ger) | † | Germany | *Sr11, 24, 31* | *Sr5, 6, 7b, 8a, 9a, 9b, 9d, 9e, 9g, 10, 17, 21, 30, 36, 38, McN, Tmp*  (+*Sr7a, Sr33, Sr45, SrTt-3)* |
| TTRTF | † | Georgia | *Sr24, 30, 31* | *Sr5, 6, 7b, 8a, 9a, 9b, 9d, 9e, 9g, 10, 11, 17, 21, 36, 38, McN, Tmp* |
| † Not part of this study so isolate numbers are not included.  Roelfs, A.P. and D.V. McVey. 1974. Races of *Puccinia graminis* f. sp. *tritici* in the U.S.A. during 1973. Plant Dis. Rept. 58:608-611.  Roelfs, A.P. and J.W. Martens. 1988. An international system of nomenclature for *Puccinia graminis* f. sp. *tritici*. Phytopathology 78:526-533. | | | | |
